# Supplementary figures and images for: C. elegans genome-wide analysis reveals DNA repair pathways that act cooperatively to preserve genome integrity upon ionizing radiation
Source: PLoS One. 2021 Oct 6;16(10):e0258269. doi: 10.1371/journal.pone.0258269 (PMC8494335; doi:10.1371/journal.pone.0258269)

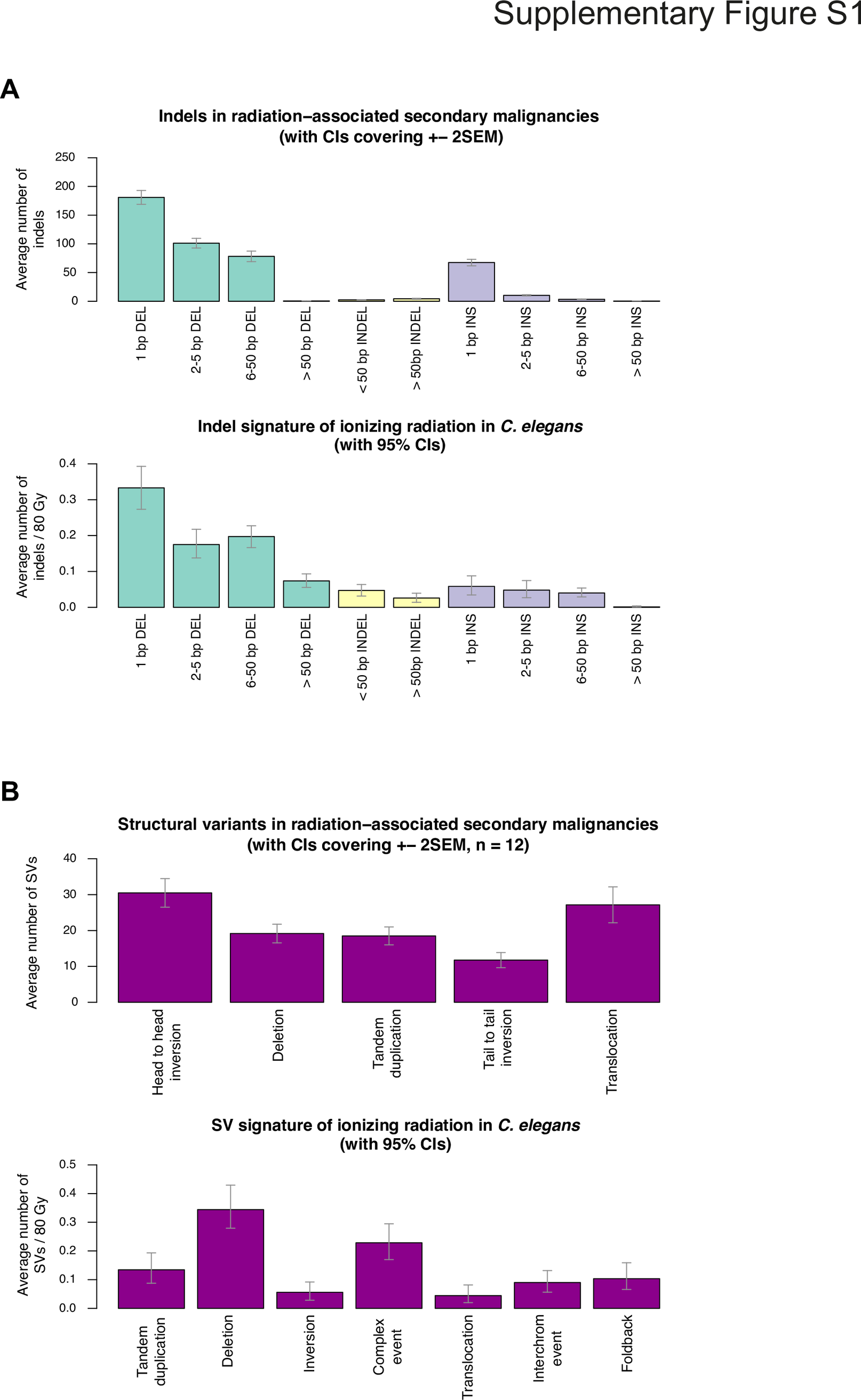

Supplement: S1 Fig — A. Average number of indels observed in secondary malignancies (1) (top panel) and Cs-137 irradiated C. elegans (bottom panel). Indels are classified as deletions (DEL), deletions with insertions (INDEL) and insertions (INS) with indicated size ranges. Error bars represent confidence intervals with 2 standard errors of the mean (SEM) (top panel) and 95% credible intervals (bottom panel). B. Average numbers of structural variants observed in secondary malignancies (1) (top panel) and in Cs-137 irradiated C. elegans (bottom panel) by type. Error bars represent confidence intervals with 2 standard errors of the mean (SEM) (top panel) and 95% credible intervals (bottom panel). (TIFF) [file pone.0258269.s003.tiff]

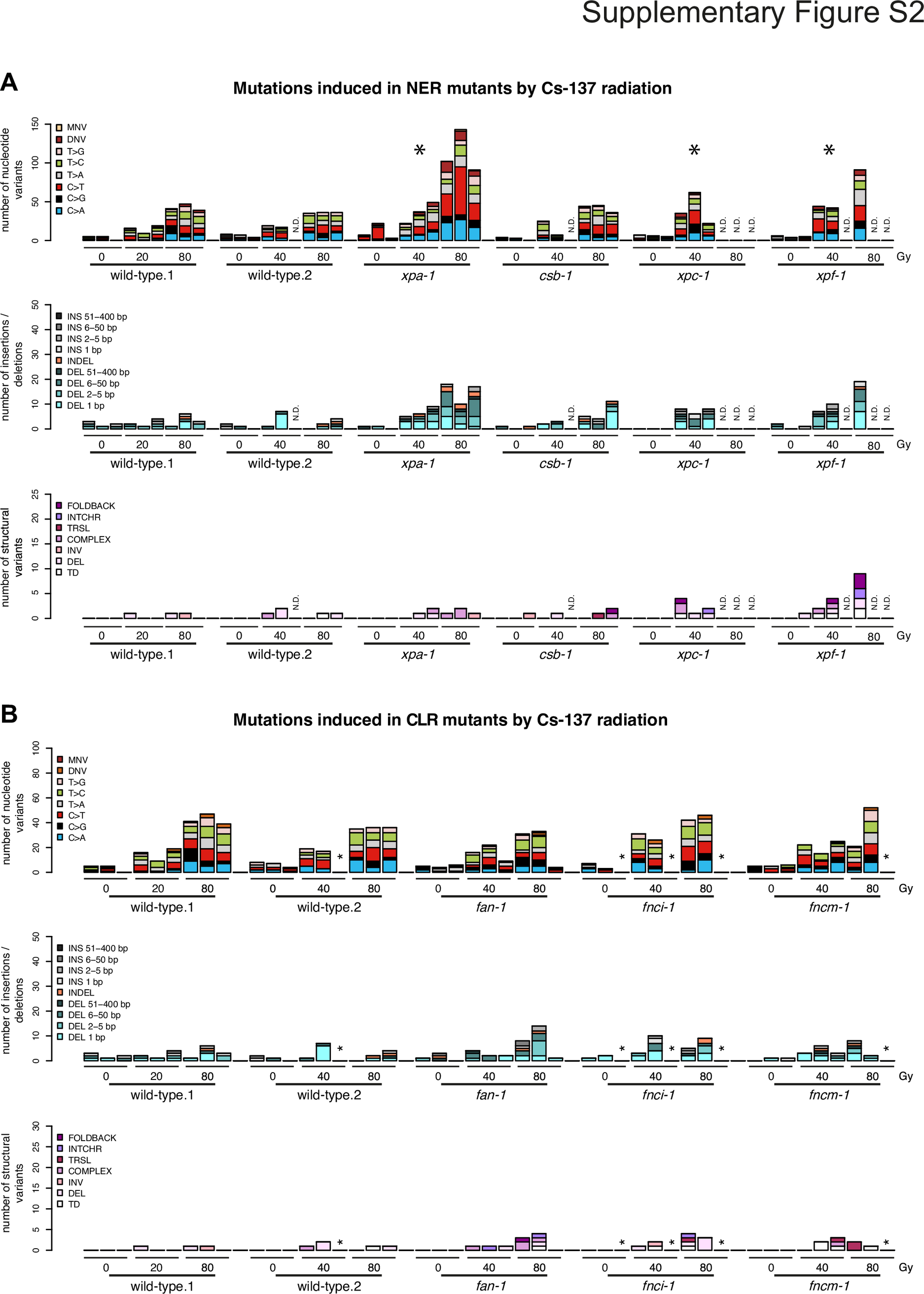

Supplement: S2 Fig — A. Mutation numbers by class and type observed in wild-type and nucleotide excision repair (NER) mutants for indicated radiation doses and 3 irradiated lines per genotype. Mutation types are shown as the 6 possible single nucleotide variants, dinucleotide variants (DNV), multi-nucleotide variants (MNVs) (top panel), insertions (INS) and deletions (DEL) and deletions with insertions (INDEL) (centre panel), and structural variants (SVs) encompassing tandem duplications (TD), large deletions (DEL), inversions (INV), translocations (TRSL), foldback (FOLDBACK), interchromosomal (INTCHR) and complex (COMPLEX) SVs (lower panel). B. Mutation numbers by class and type observed in wild-type and DNA crosslink repair (CLR) mutants for indicated radiation doses and 3 irradiated lines per genotype. Mutation types as described in A. N.D. indicates lines for which no DNA sequencing information could be obtained. Strains with mutational patterns statistically significantly different from wild-type are indicated by an asterisk ‘*’ (P ≤ 0.05). Strains lacking a dose-response and thus were excluded from our detailed analysis are indicated with ∉. (TIFF) [file pone.0258269.s004.tiff]

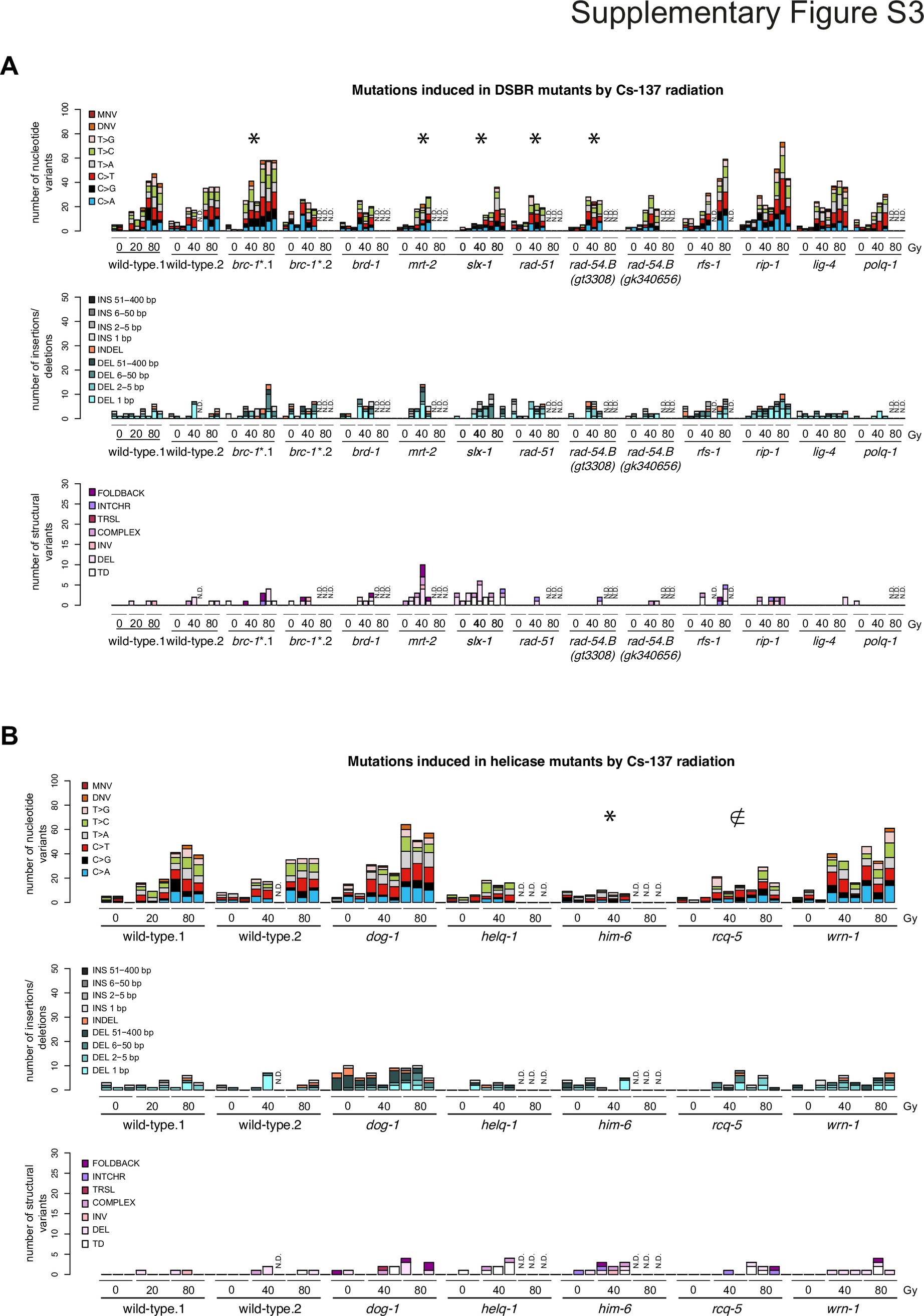

Supplement: S3 Fig — A. Mutation numbers by class and type observed in wild-type and double-strand break repair (DSBR) mutants for 3 irradiated lines per genotype and indicated radiation dose. B. Mutation numbers by class and type observed in wild-type and helicase mutants for indicated radiation doses and 3 irradiated lines per genotype. N.D. indicates lines for which no DNA sequencing information could be obtained. Strains with mutational patterns statistically significantly different from wild-type are indicated by an asterisk ‘*’ (P ≤ 0.05). Strains lacking a dose-response and thus excluded from our detailed analysis are indicated with ∉. (TIFF) [file pone.0258269.s005.tiff]

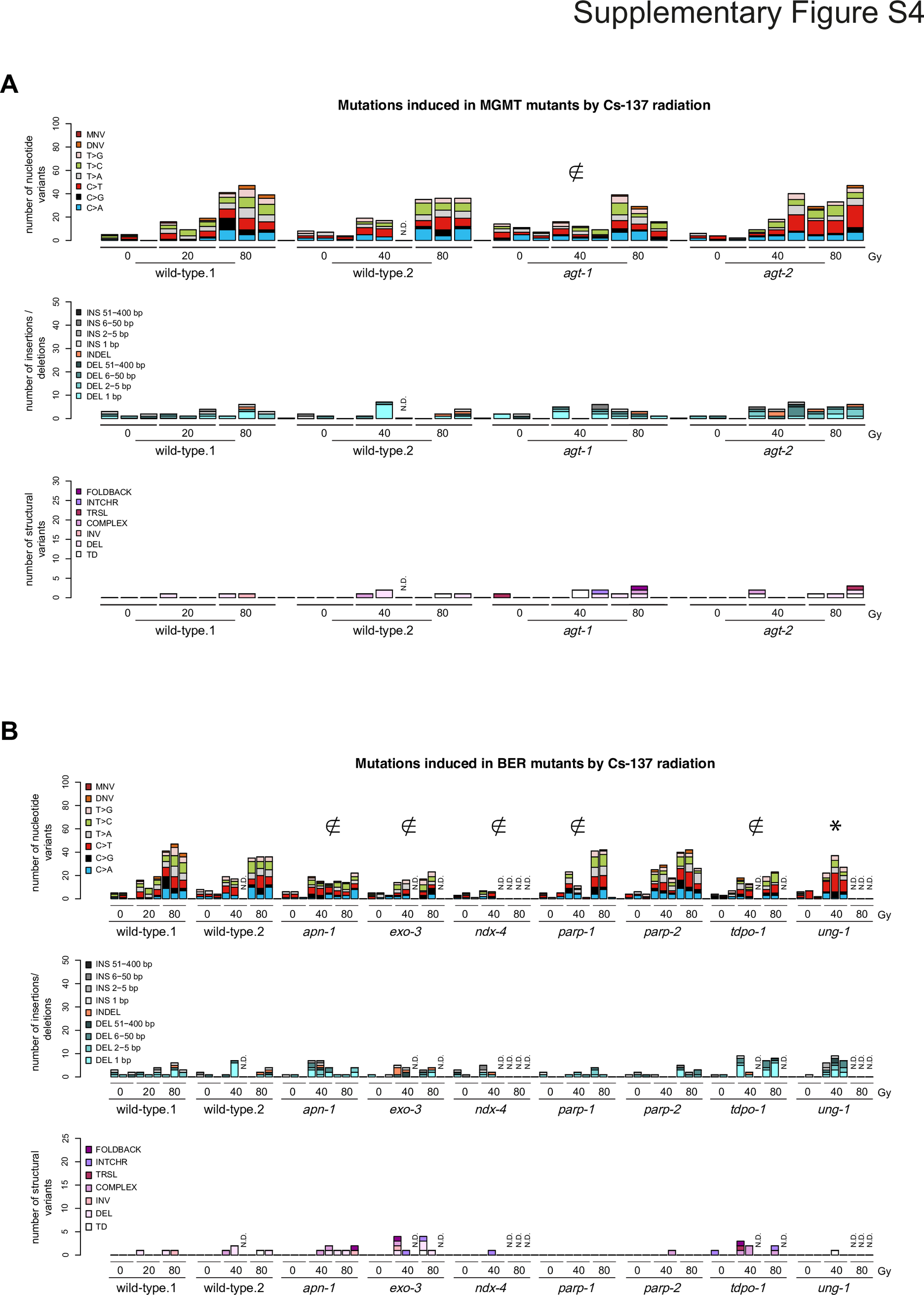

Supplement: S4 Fig — A. Mutation numbers by class and type observed in wild-type and putative direct damage reversal repair 06-Methylguanine methyltransferase (MGMT) mutants for indicated radiation doses and 3 irradiated lines per genotype. B. Mutation numbers by class and type observed in wild-type and base excision repair (BER) mutants for indicated radiation doses and 3 irradiated lines per genotype. N.D. indicates lines for which no DNA sequencing information could be obtained. Strains with mutational patterns statistically significantly different from wild-type are indicated by an asterisk ‘*’ (P ≤ 0.05). Strains lacking a dose-response thus excluded from our detailed analysis are indicated with ∉. (TIFF) [file pone.0258269.s006.tiff]

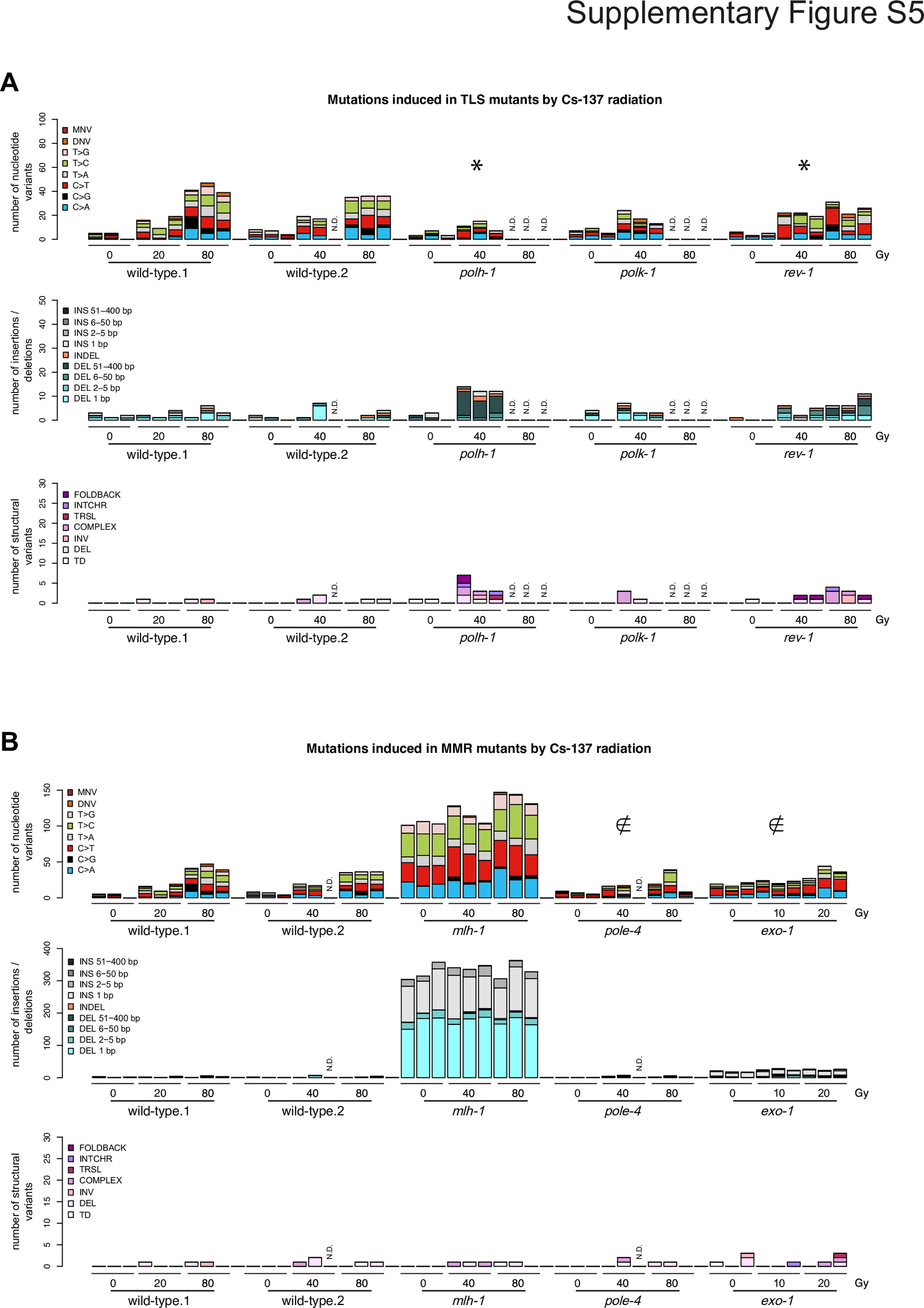

Supplement: S5 Fig — A. Mutation numbers by class and type observed in wild-type and translesion synthesis (TLS) mutants for indicated radiation doses and 3 irradiated lines per genotype. N.D. indicates lines for which no DNA sequencing information could be obtained. Strains with mutational patterns statistically significantly different from wild-type are indicated by an asterisk ‘*’ (P ≤ 0.05). Strains lacking a dose-response thus excluded from our detailed analysis are indicated with ∉. (TIFF) [file pone.0258269.s007.tiff]

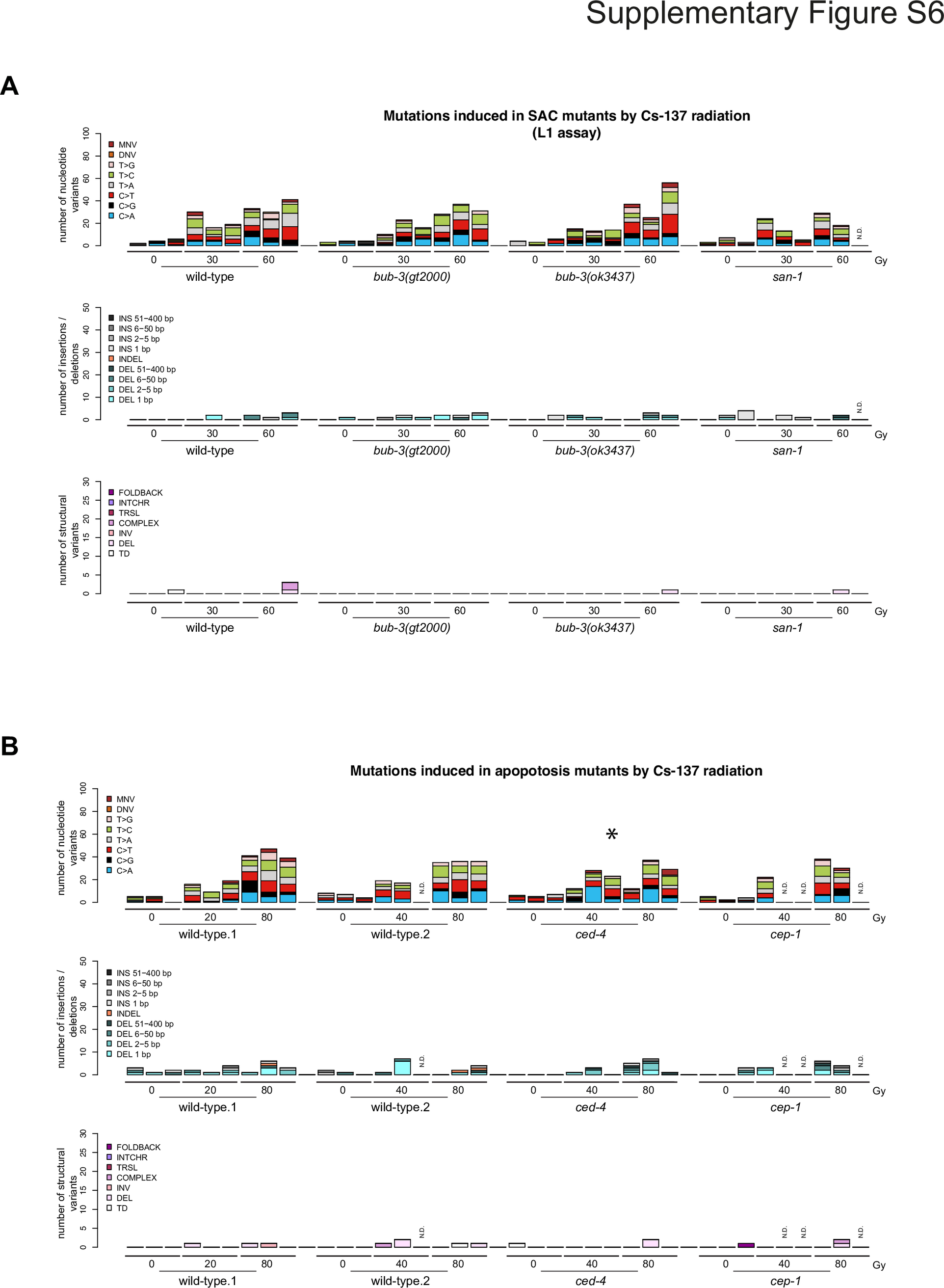

Supplement: S6 Fig — A. Mutation numbers by class and type observed in wild-type and spindle assembly checkpoint (SAC) mutants for indicated radiation doses and 3 irradiated lines per genotype. B. Mutation numbers by class and type observed in wild-type and apoptosis mutants for indicated radiation doses and 3 irradiated lines per genotype. N.D. indicates lines for which no DNA sequencing information could be obtained. Strains with mutational patterns statistically significantly different from wild-type are indicated by an asterisk ‘*’ (P ≤ 0.05). Strains lacking a dose-response thus excluded from our detailed analysis are indicated with ∉. (TIFF) [file pone.0258269.s008.tiff]

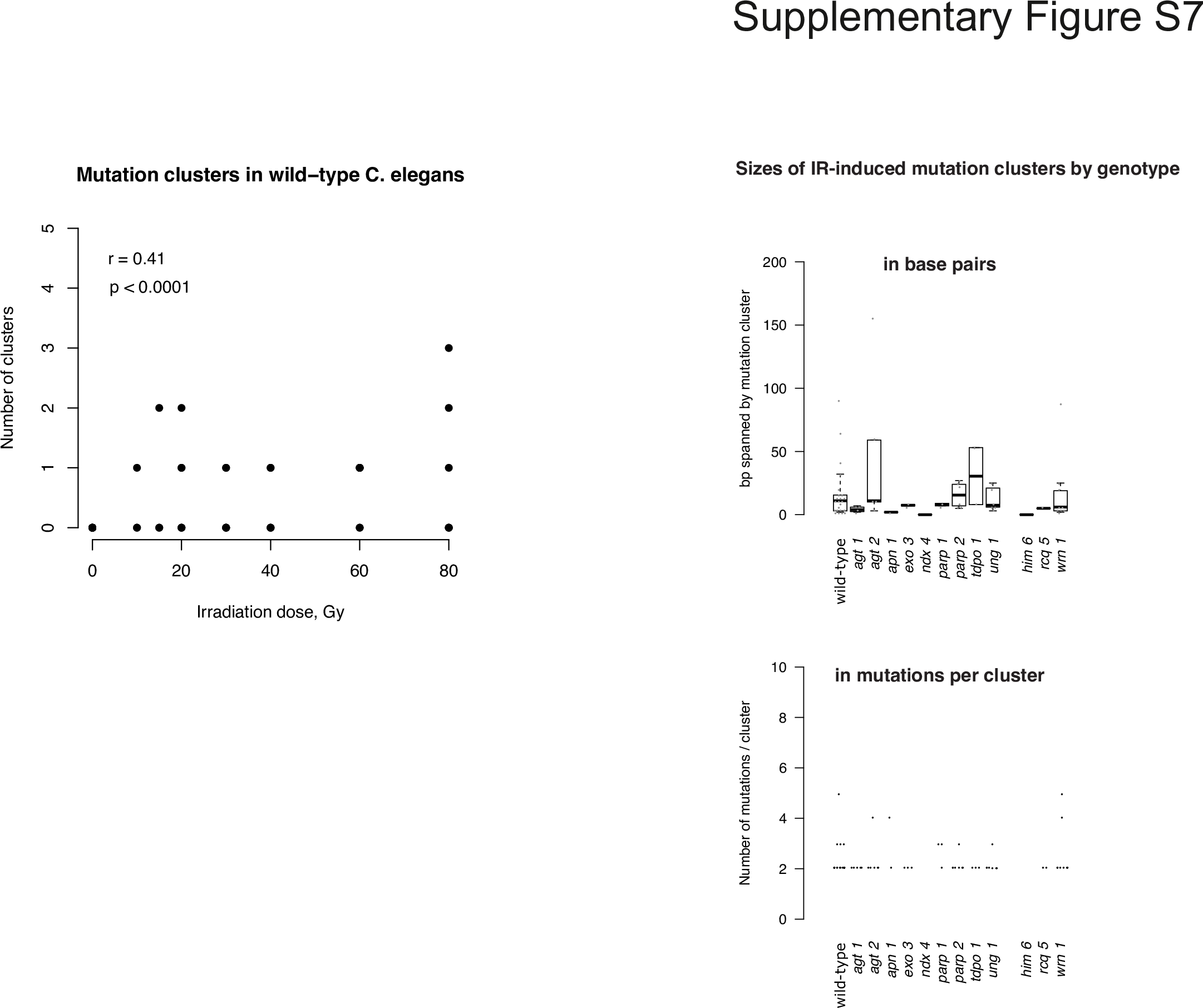

Supplement: S7 Fig — Sizes of IR-induced mutation clusters shown in bp spanned by the mutation cluster (top panel) and as the number of mutations per cluster (bottom panel). Grey or black dots indicate bp spanned or mutations by cluster, respectively, observed in individual lines of the indicated genotypes. Black bars of the boxplot (right top panel) indicate the median mutation cluster size, squares the interquartile range and error bars 1.5*interquartile ranges. (TIFF) [file pone.0258269.s009.tiff]

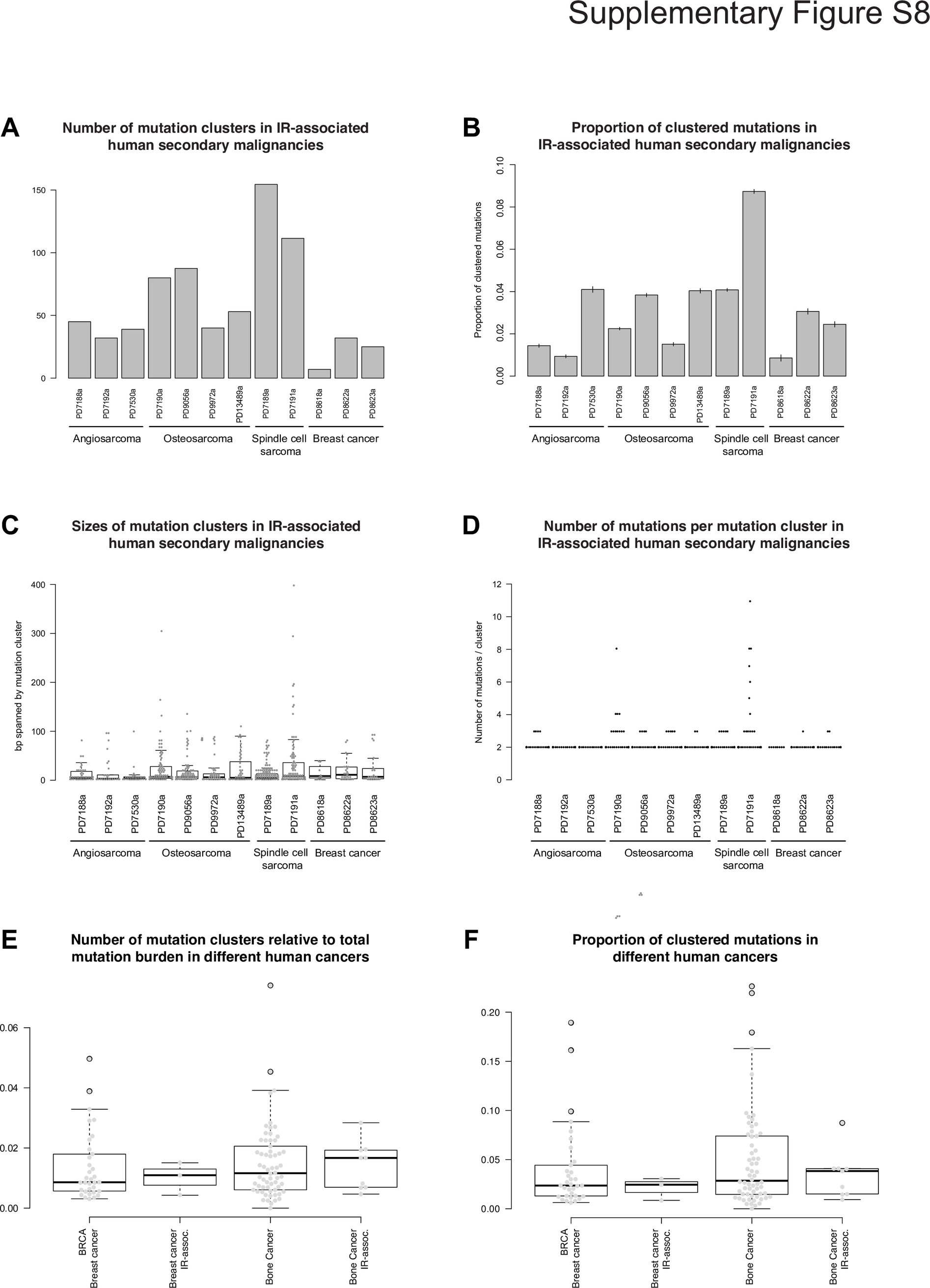

Supplement: S8 Fig — A. Observed number of mutation clusters in 12 IR-associated human secondary malignancies (Material and Methods) [1]. B. Proportion of clustered mutations in 12 IR-associated human secondary malignancies. C. Boxplots depicting the sizes of mutation clusters in 12 IR-associated human secondary malignancies in bp. Black bars indicate the median mutation cluster size, squares the interquartile range, and error bars 1.5* interquartile ranges. Grey dots represent the size of individual mutation clusters observed in the respective tumour. D. Number of mutations per mutation cluster in 12 IR-associated human secondary malignancies. Black dots represent the number of mutations observed in individual clusters of the respective tumour. E. Number of mutation clusters in different human cancers. Mutation clusters are shown as the relative number of mutation clusters to total mutation burden. Grey dots represent the number of mutation clusters in 33 individual non-IR associated BRCA negative breast cancers, 3 IR-associated secondary breast cancers (PD8618a, PD8622a, PD8623a, Panel A-D), 62 non-IR associated bone cancers, and 9 IR-associated bone cancers including angio-, osteo-, and spindle cells sarcomas (Panel A-D). Outliers are highlighted by a black outline. Black bars indicate the median mutation cluster size, squares the interquartile range and error bars 1.5* interquartile ranges. F. Proportion of clustered mutations in different human cancers shown in non-IR and IR-associated human cancers as described in E. (TIFF) [file pone.0258269.s010.tiff]
