# Supplementary material for: C. elegans genome-wide analysis reveals DNA repair pathways that act cooperatively to preserve genome integrity upon ionizing radiation
Source: PLoS One. 2021 Oct 6;16(10):e0258269. doi: 10.1371/journal.pone.0258269 (PMC8494335; doi:10.1371/journal.pone.0258269)
Supplement: S1 File — Details of the observed number of mutations in all samples of C. elegans wild-type and mutant strains across radiation doses. Number of mutations observed across all Cs-137-irradiated samples by dose and mutation type: single nucleotide variants (left panel), indels (center panel) and structural variants (left panel). Black dots represent mutations observed in individual samples at a given dose, red lines represent a best fit linear regression. (PDF) [file pone.0258269.s011.pdf]

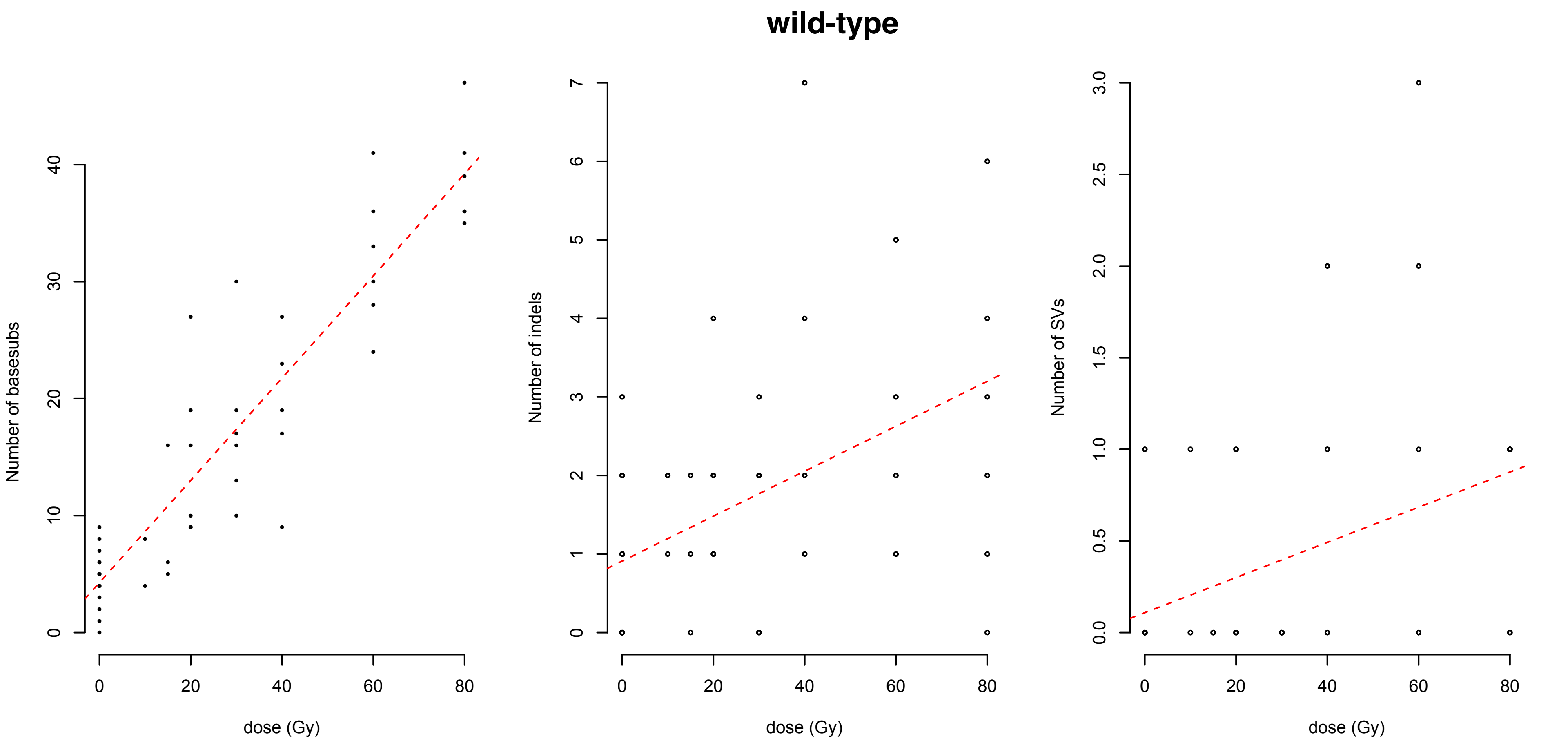

wild-type
Number of basesubs
Number of indels
Number of SVs
dose (Gy)
dose (Gy)
dose (Gy)
0
10
20
30
40
0
1
2
3
4
5
6
7
0.0
0.5
1.0
1.5
2.0
2.5
3.0
0
20
40
60
80

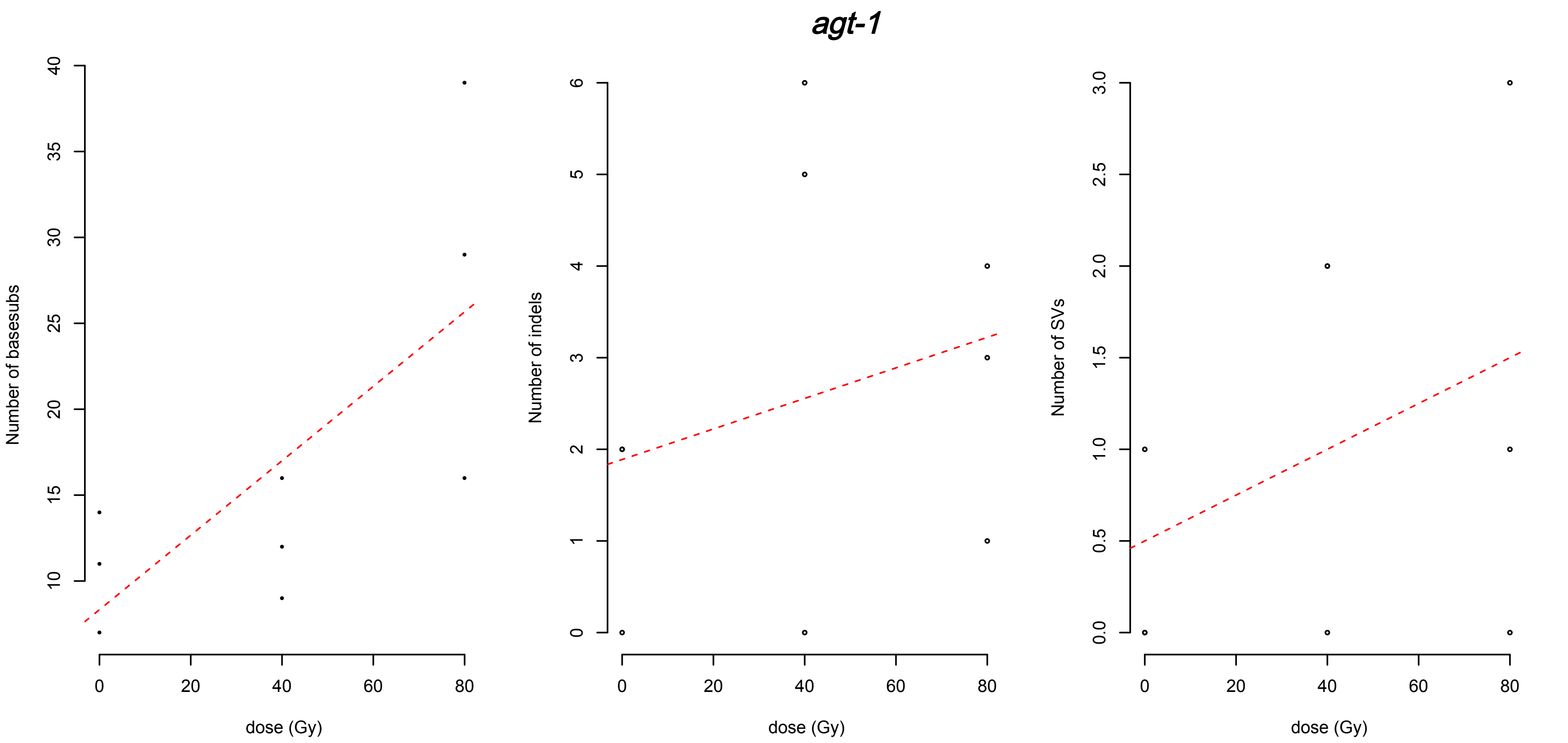
agt-1
Number of basesubs
10
15
20
25
30
35
40
Number of indels
0
1
2
3
4
5
6
Number of SVs
0.0
0.5
1.0
1.5
2.0
2.5
3.0
0
20
40
60
80
dose (Gy)

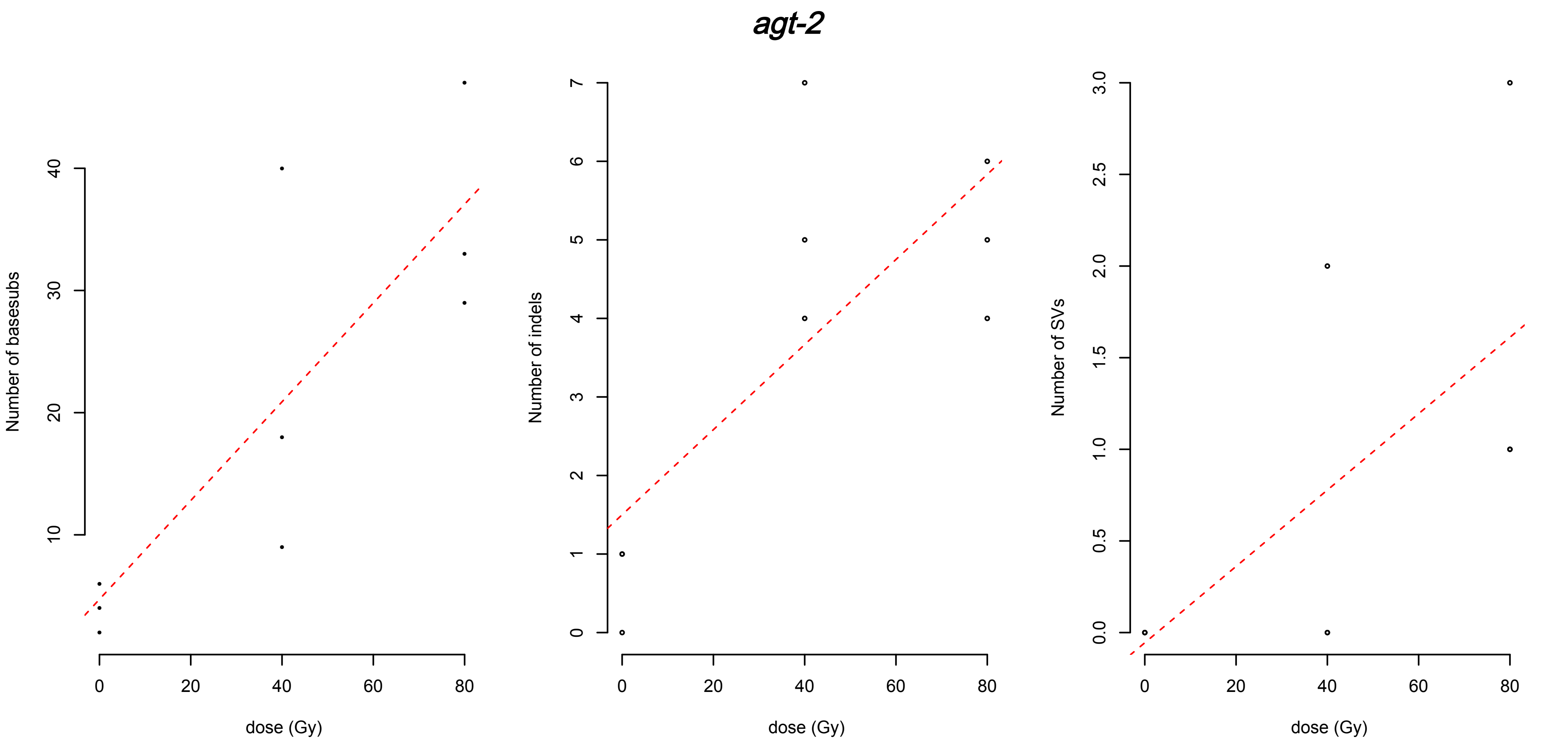

agt-2
Number of basesubs
10
20
30
40
0
20
40
60
80
dose (Gy)
Number of indels
0
1
2
3
4
5
6
7
0
20
40
60
80
dose (Gy)
Number of SVs
0.0
0.5
1.0
1.5
2.0
2.5
3.0
0
20
40
60
80
dose (Gy)

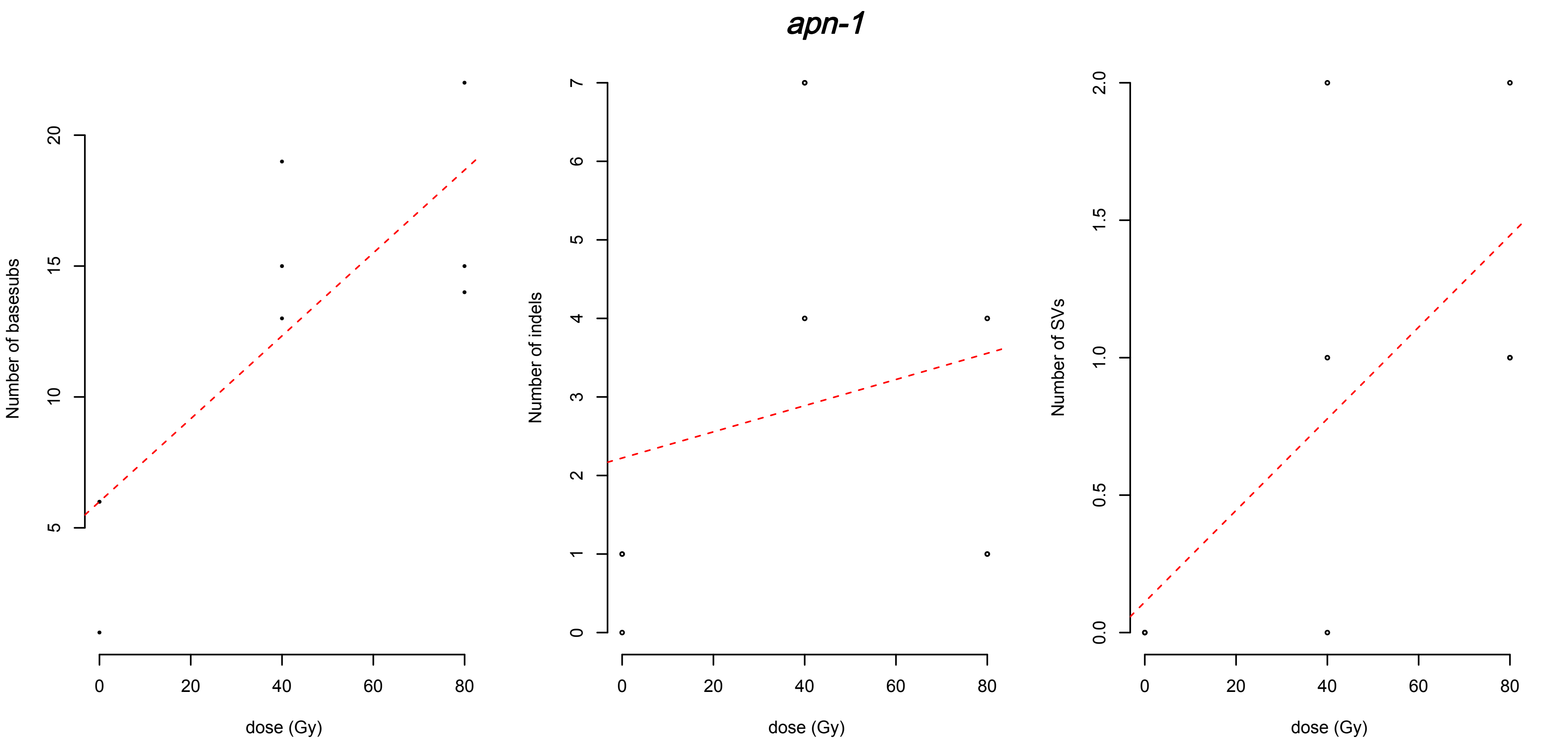

apn-1
Number of basesubs
dose (Gy)
Number of indels
dose (Gy)
Number of SVs
dose (Gy)

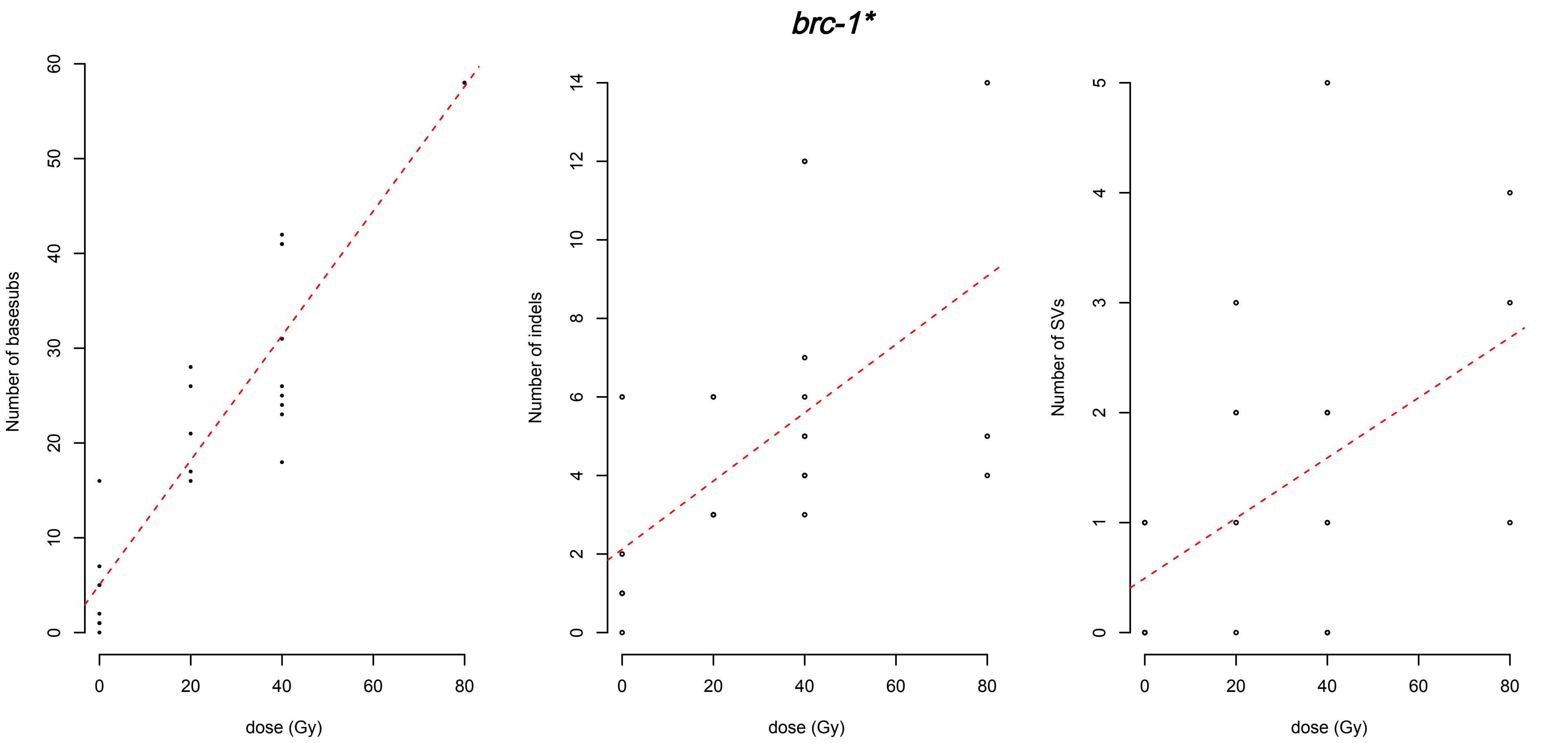

brc-1*
Number of basesubs
Number of indels
Number of SVs
dose (Gy)
dose (Gy)
dose (Gy)

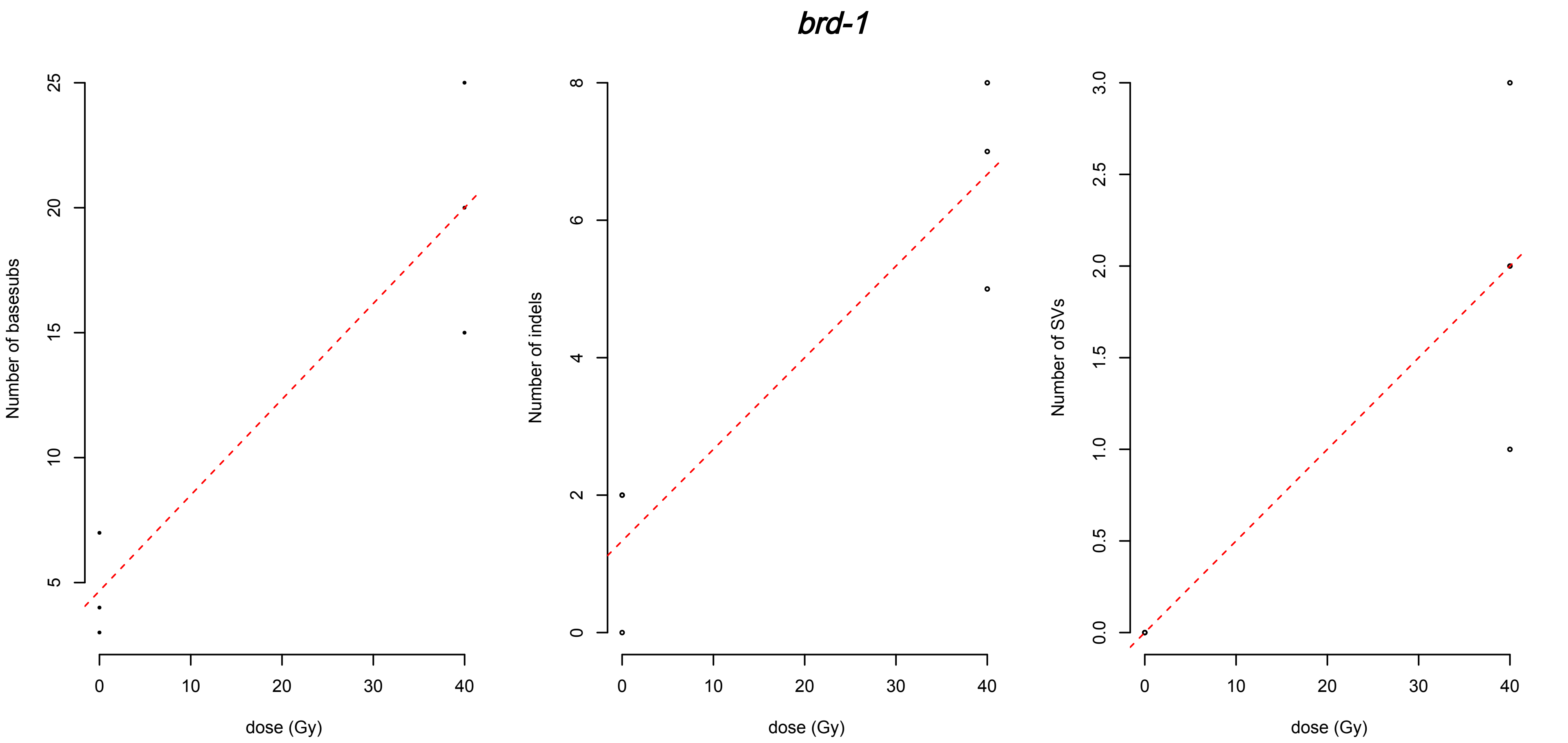

brd-1
Number of basesubs
Number of indels
Number of SVs
dose (Gy)
dose (Gy)
dose (Gy)

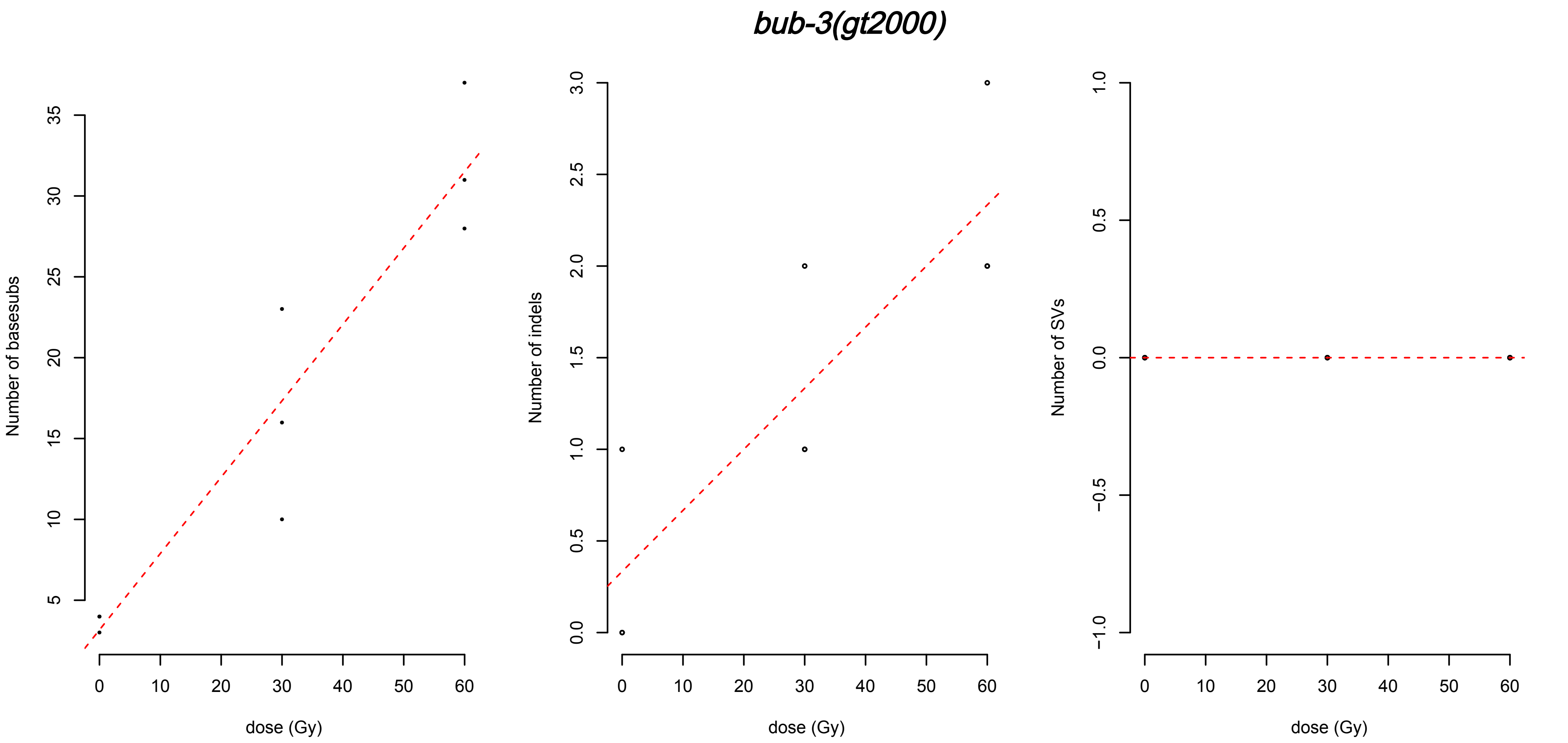

bub-3(gt2000)
Number of basesubs
5
10
15
20
25
30
35
0
10
20
30
40
50
60
dose (Gy)
Number of indels
0.0
0.5
1.0
1.5
2.0
2.5
3.0
0
10
20
30
40
50
60
dose (Gy)
Number of SVs
−1.0
−0.5
0.0
0.5
1.0
0
10
20
30
40
50
60
dose (Gy)

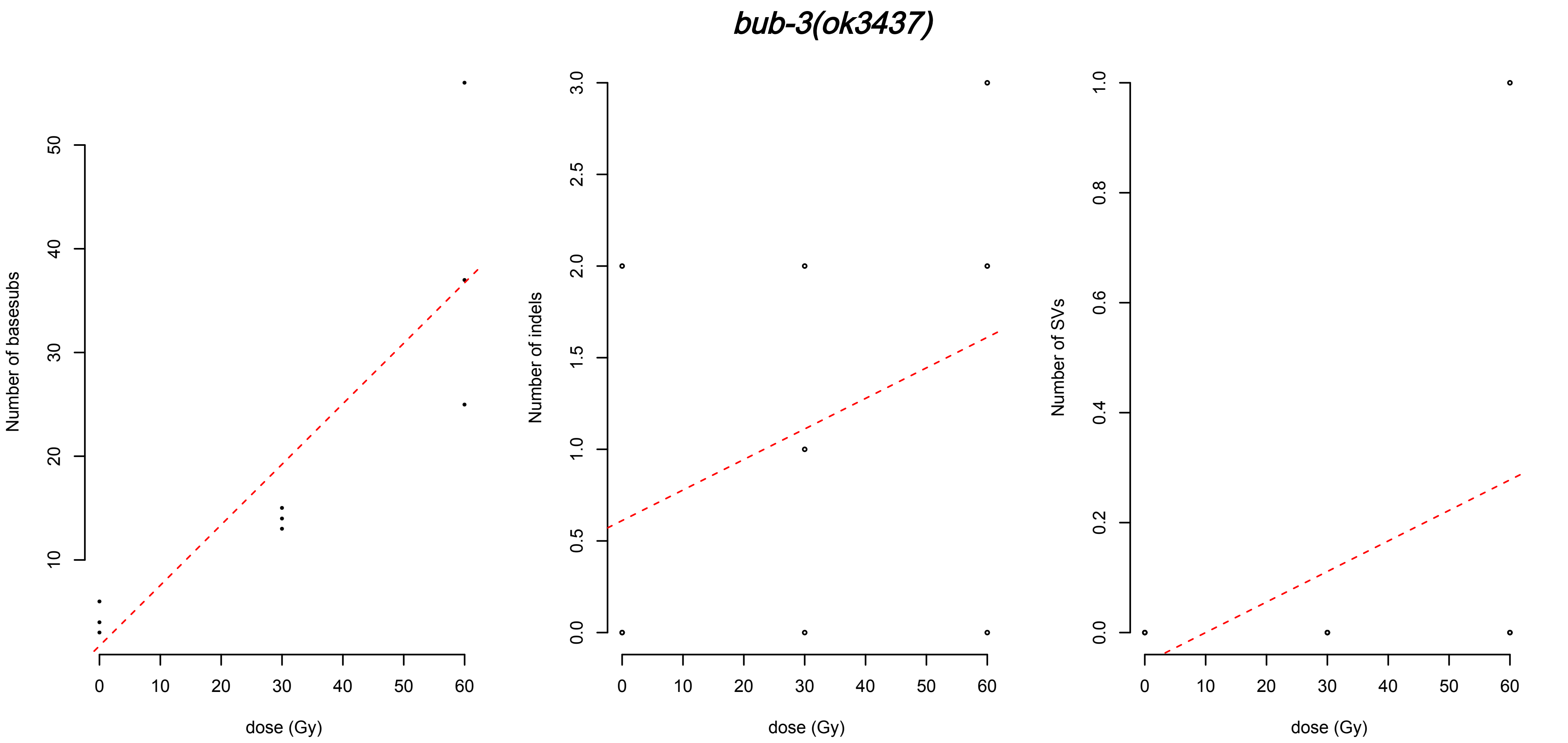

bub-3(ok3437)
Number of basesubs
10
20
30
40
50
0
10
20
30
40
50
60
dose (Gy)
Number of indels
0.0
0.5
1.0
1.5
2.0
2.5
3.0
0
10
20
30
40
50
60
dose (Gy)
Number of SVs
0.0
0.2
0.4
0.6
0.8
1.0
0
10
20
30
40
50
60
dose (Gy)

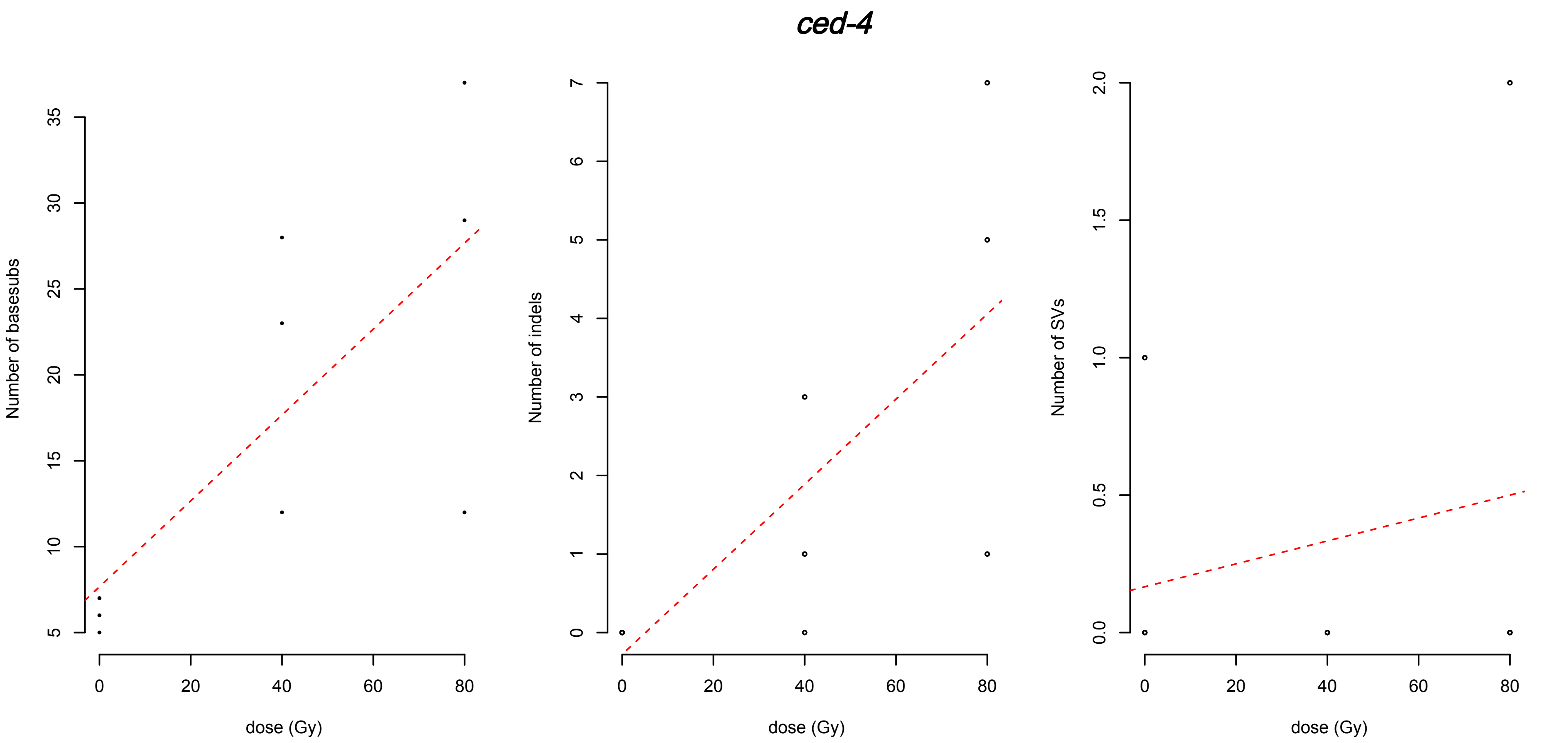

ced-4
Number of basesubs
Number of indels
Number of SVs
dose (Gy)
dose (Gy)
dose (Gy)

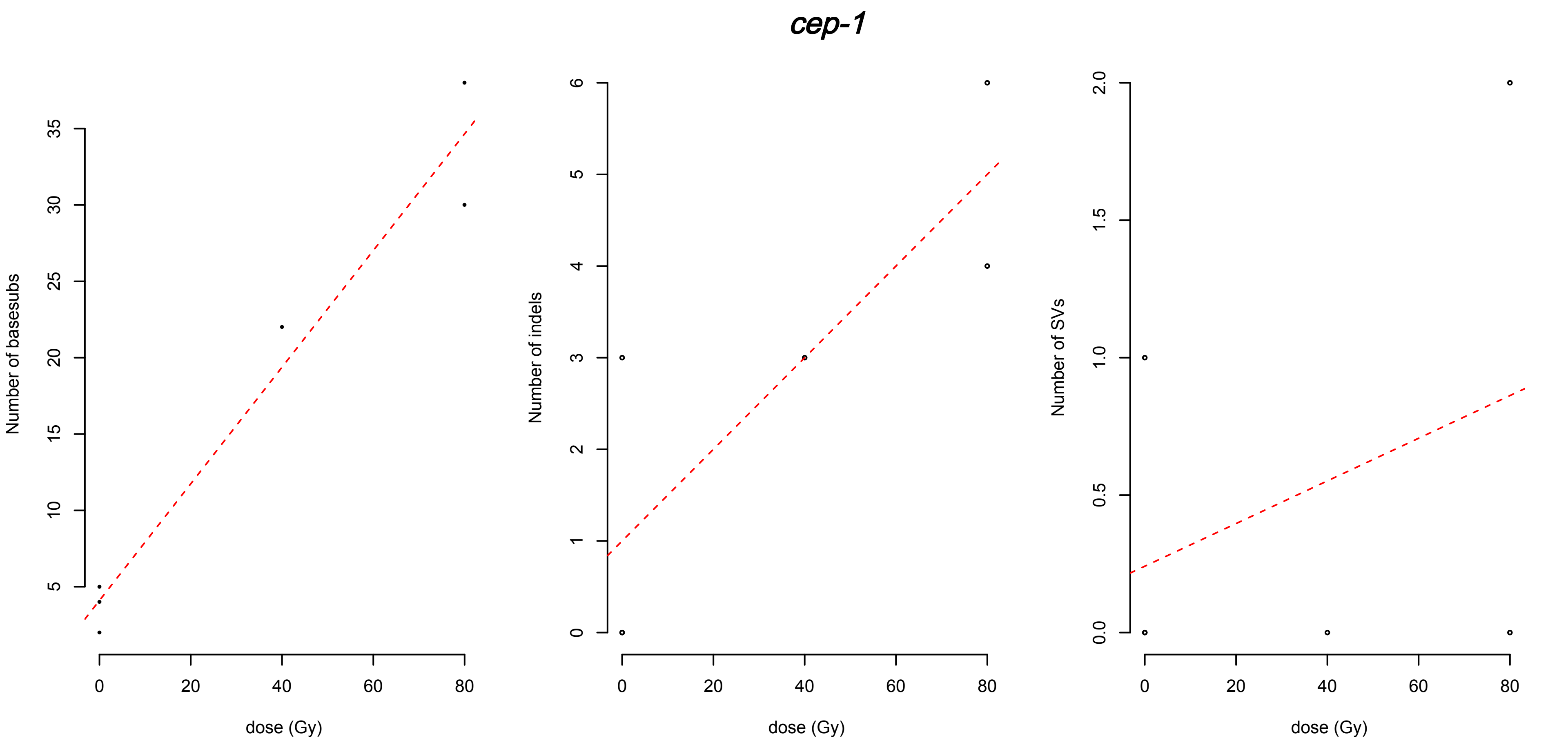

cep-1
Number of basesubs
5
10
15
20
25
30
35
0
20
40
60
80
dose (Gy)
Number of indels
0
1
2
3
4
5
6
0
20
40
60
80
dose (Gy)
Number of SVs
0.0
0.5
1.0
1.5
2.0
0
20
40
60
80
dose (Gy)

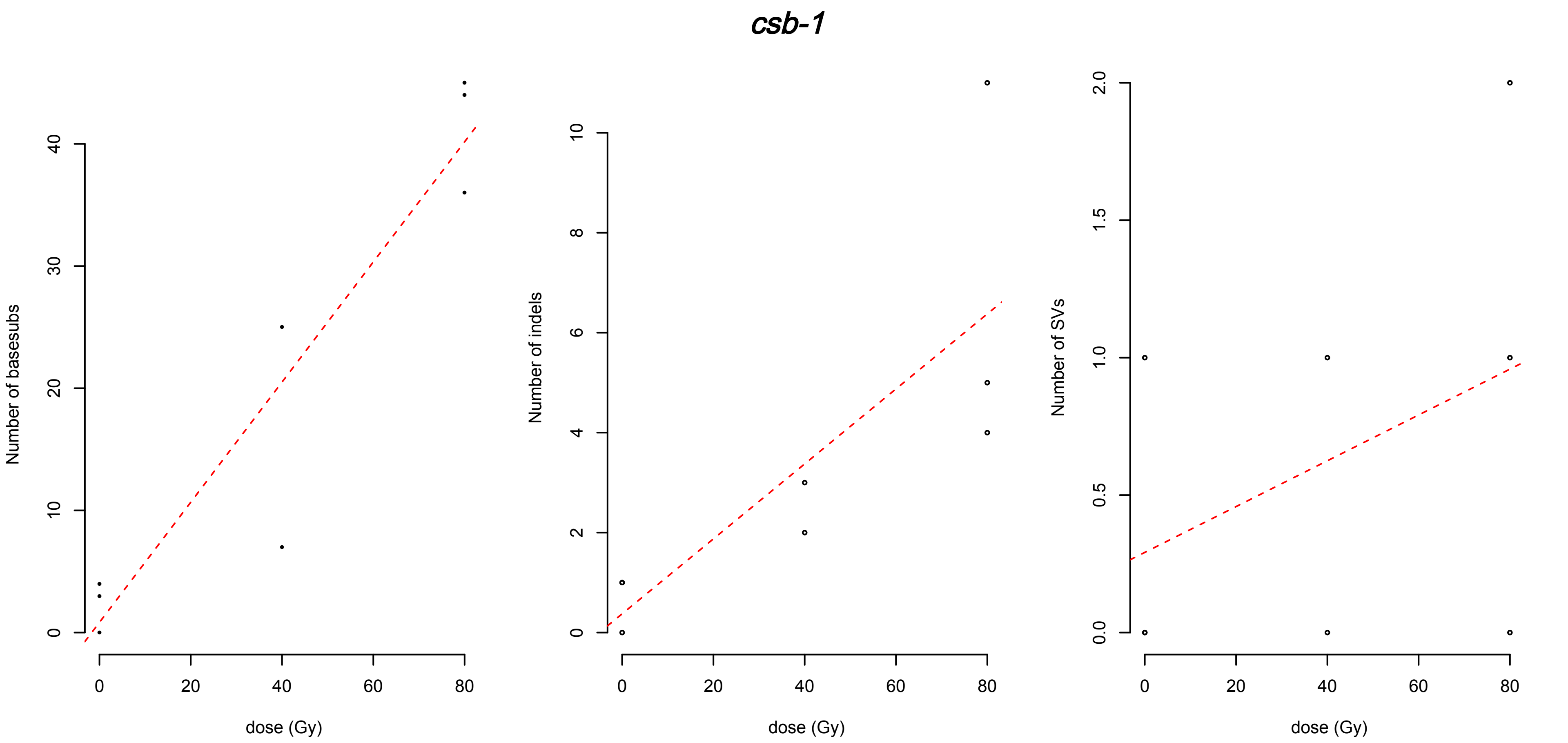

csb-1
Number of basesubs
Number of indels
Number of SVs
dose (Gy)
dose (Gy)
dose (Gy)
0
10
20
30
40
0
2
4
6
8
10
0.0
0.5
1.0
1.5
2.0
0
20
40
60
80

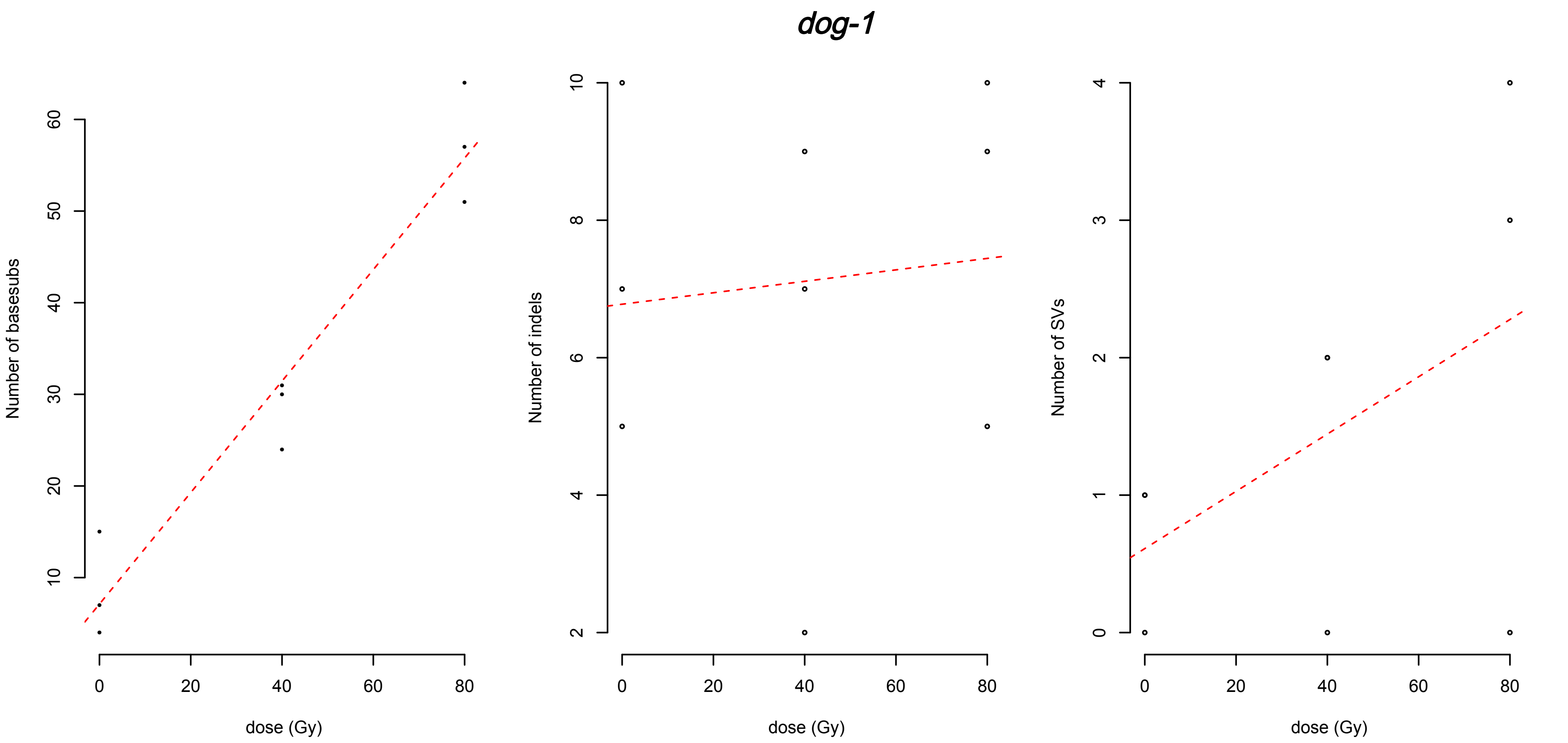
dog-1
Number of basesubs
dose (Gy)
Number of indels
dose (Gy)
Number of SVs
dose (Gy)
0
20
40
60
80
10
20
30
40
50
60
2
4
6
8
10
0
1
2
3
4

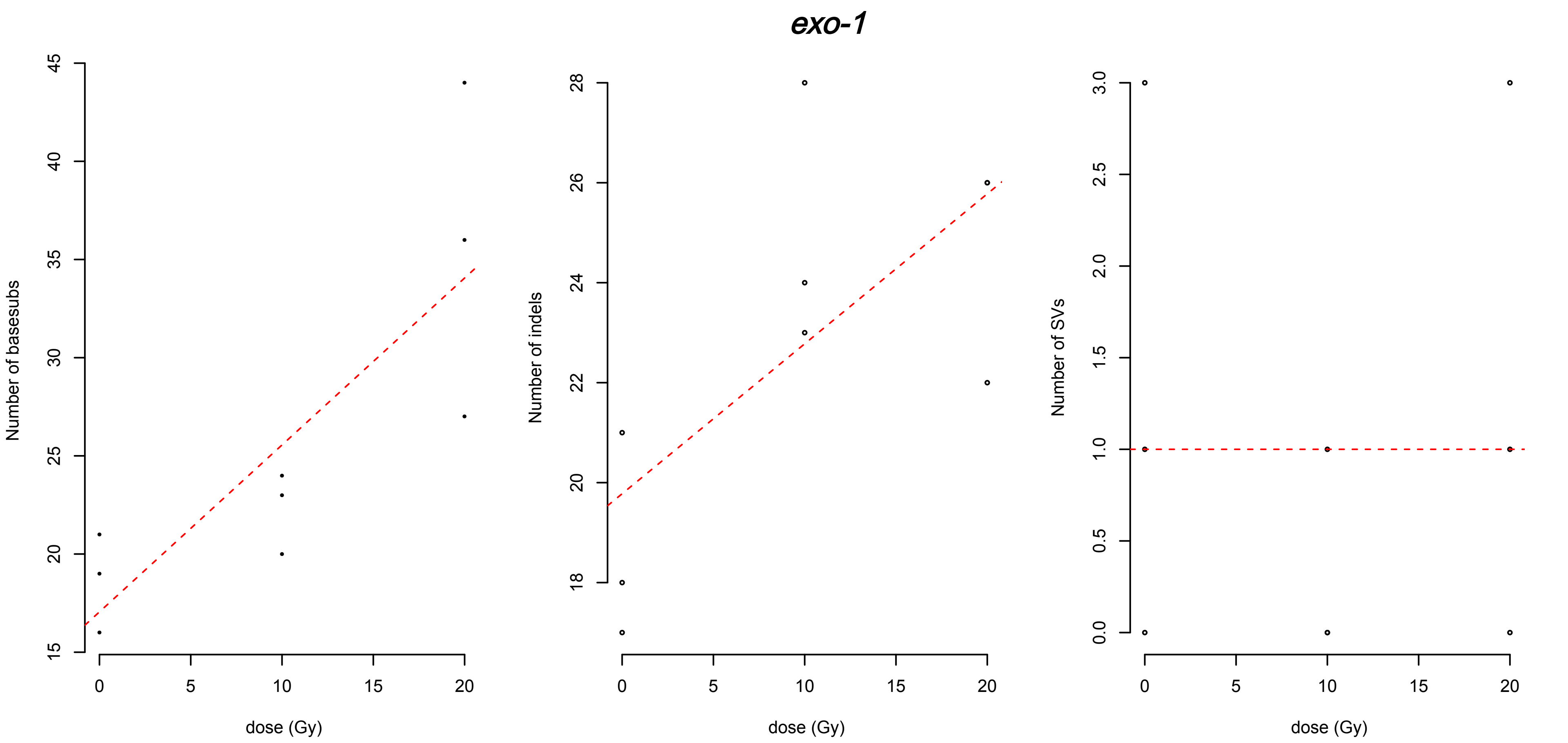

exo-1
Number of basesubs
Number of indels
Number of SVs
dose (Gy)
dose (Gy)
dose (Gy)

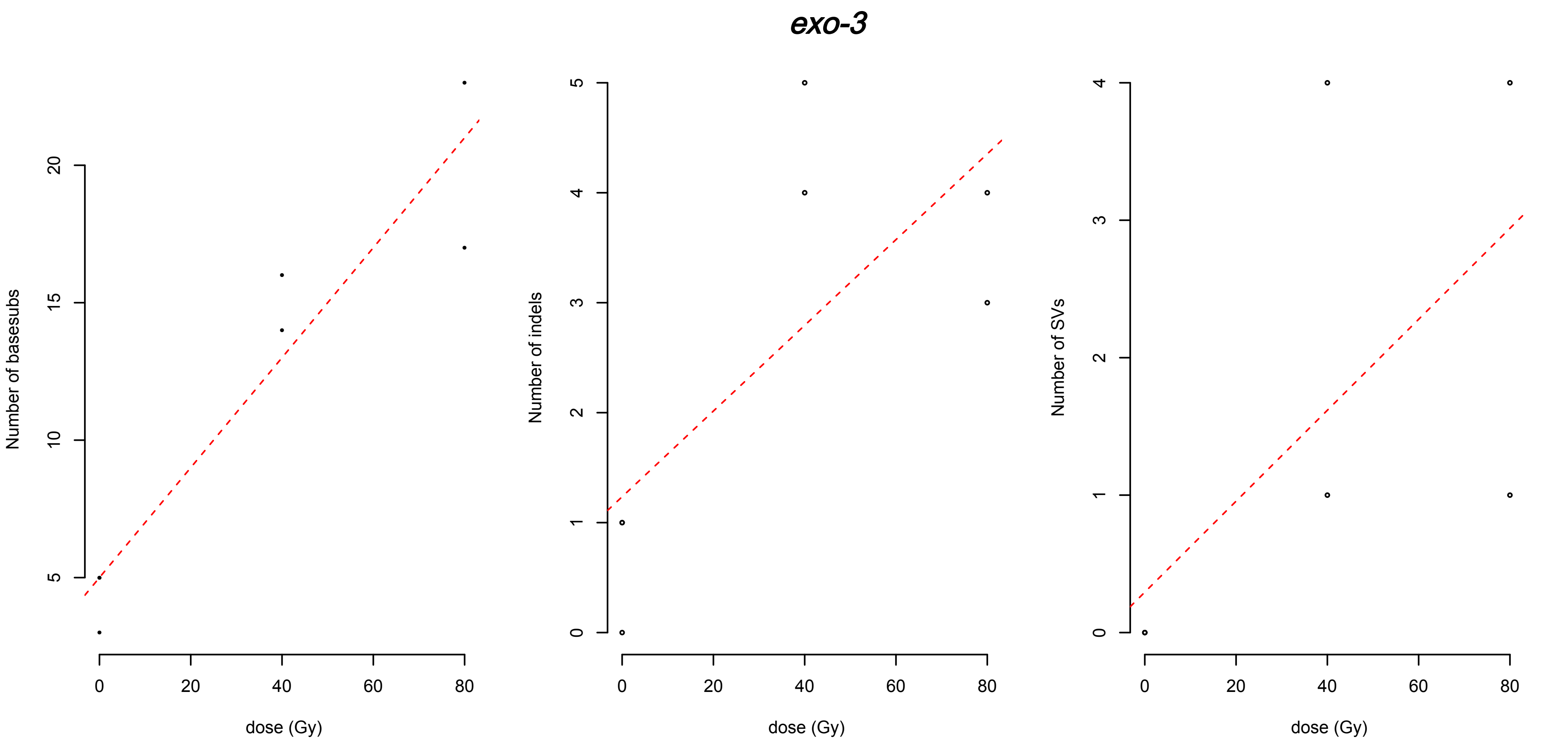
exo-3
Number of basesubs
Number of indels
Number of SVs
dose (Gy)
dose (Gy)
dose (Gy)
0
20
40
60
80
5
10
15
20
0
1
2
3
4
5
0
1
2
3
4

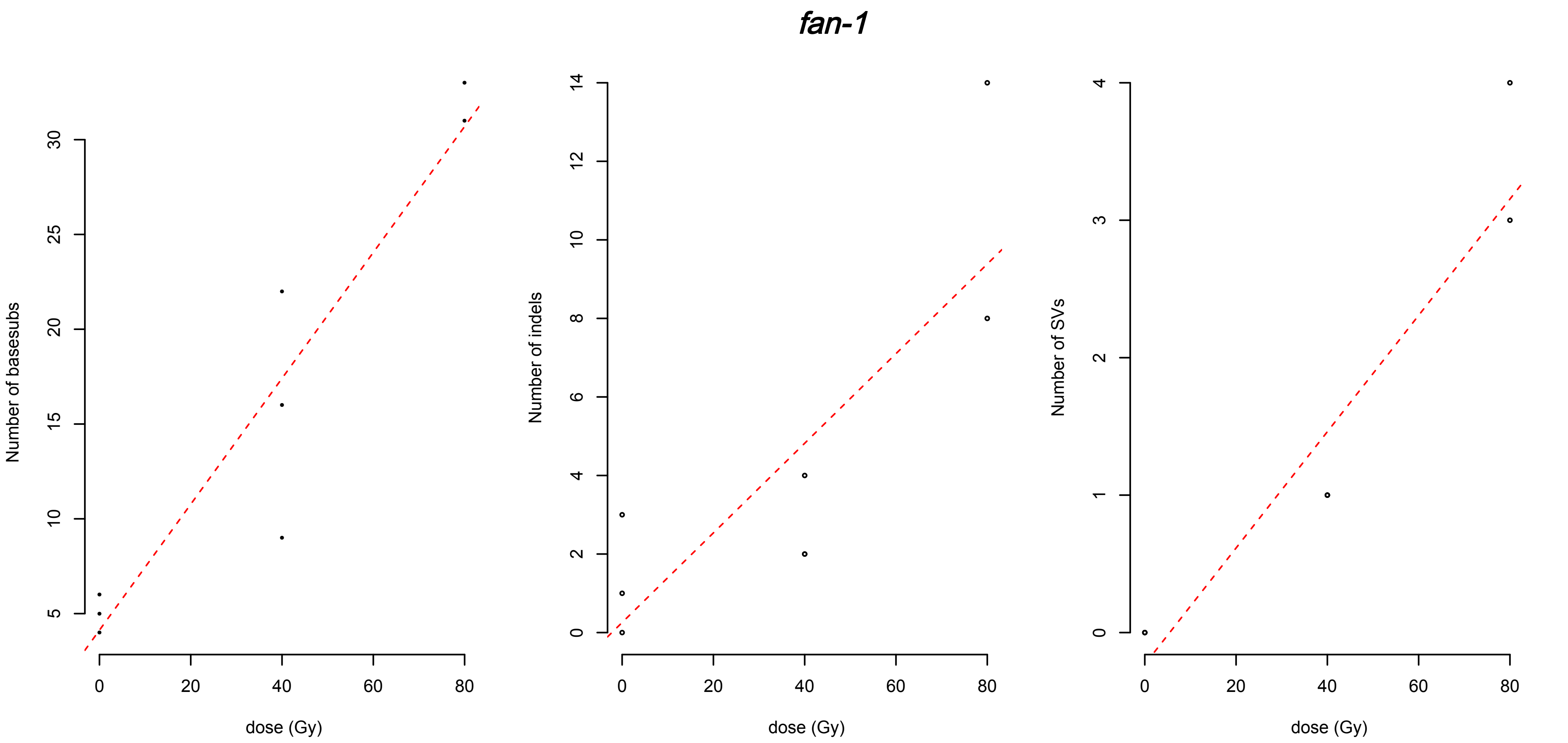

fan-1
Number of basesubs
5
10
15
20
25
30
0
20
40
60
80
dose (Gy)
Number of indels
0
2
4
6
8
10
12
14
0
20
40
60
80
dose (Gy)
Number of SVs
0
1
2
3
4
0
20
40
60
80
dose (Gy)

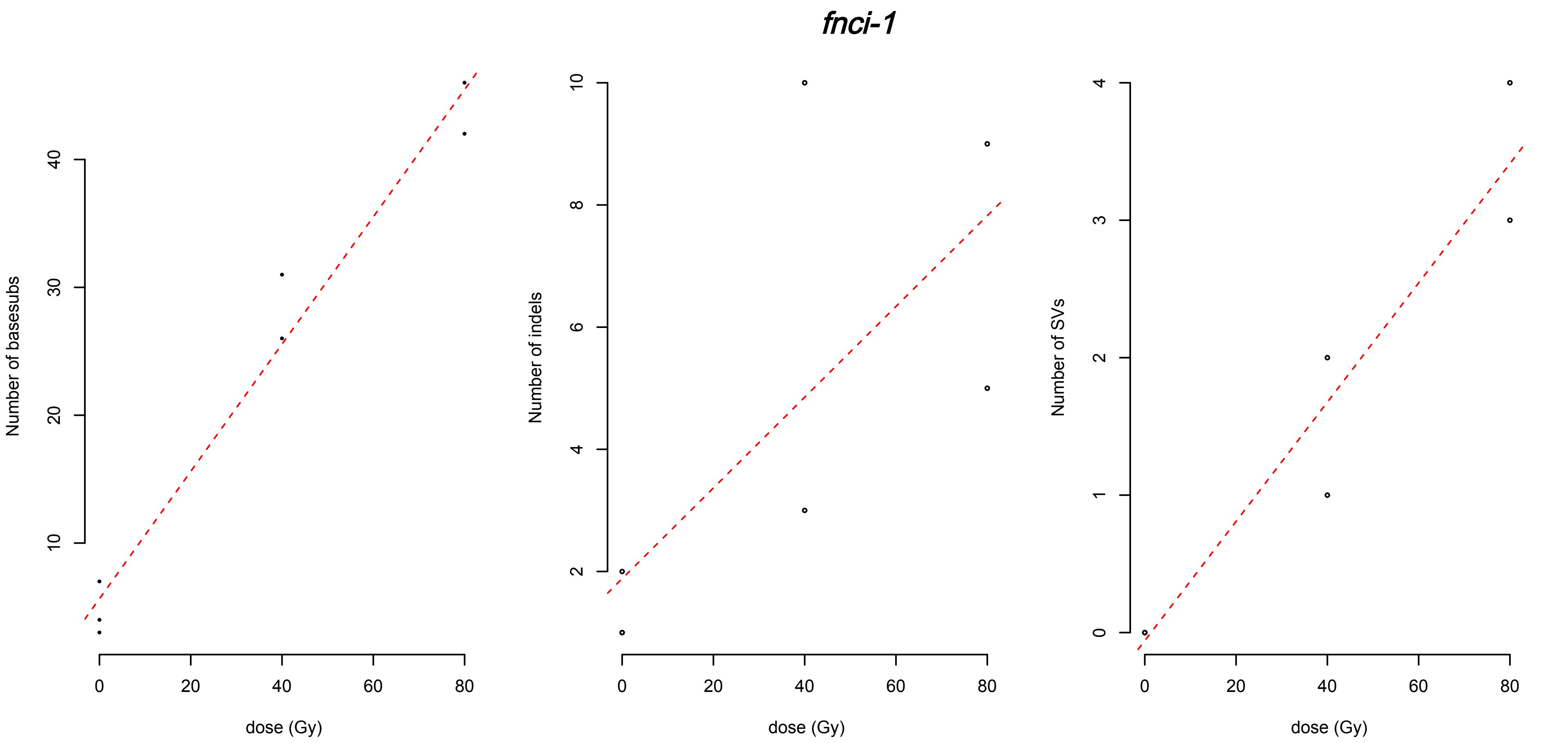

fnci-1
Number of basesubs
dose (Gy)
Number of indels
dose (Gy)
Number of SVs
dose (Gy)

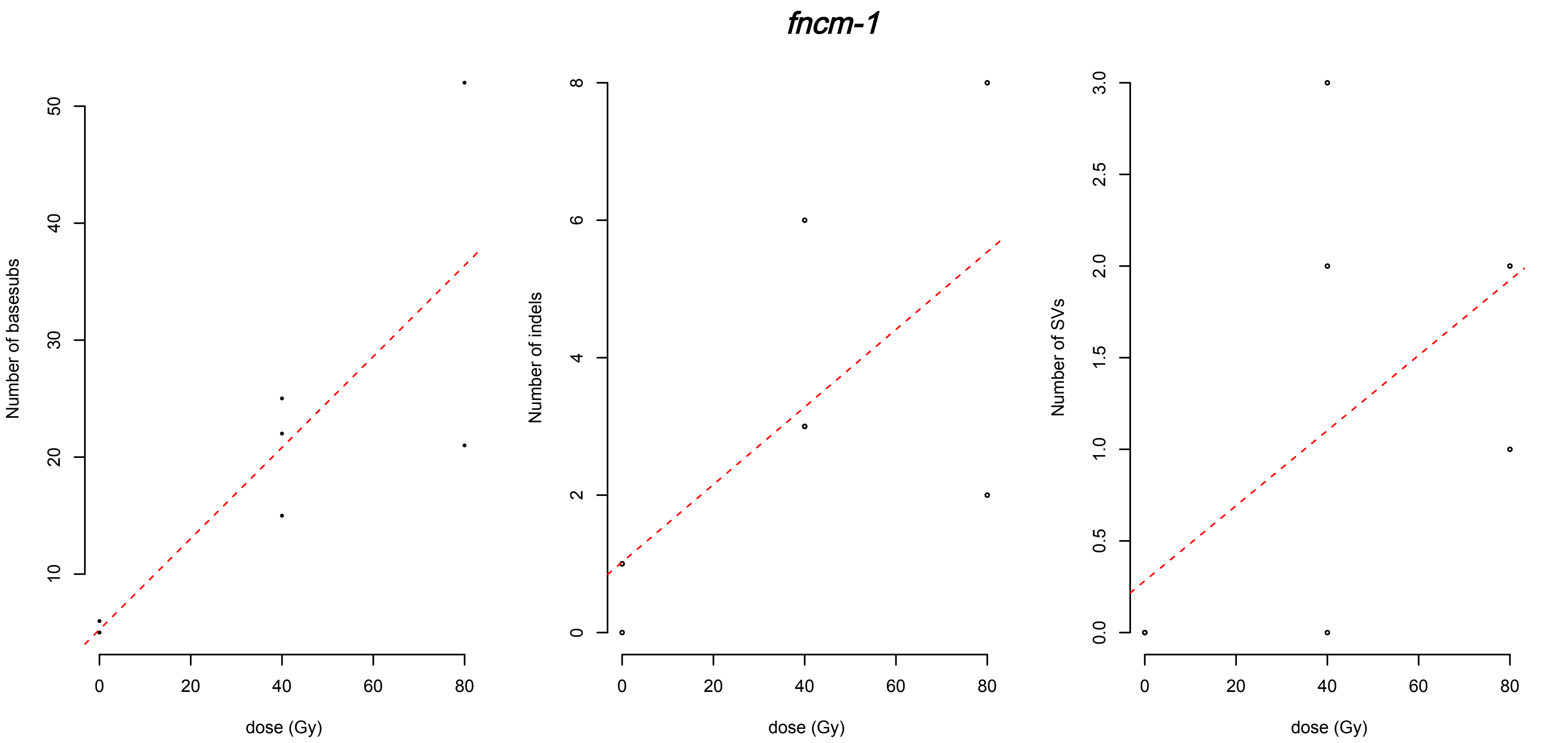

fncm-1
Number of basesubs
Number of indels
Number of SVs
dose (Gy)
dose (Gy)
dose (Gy)

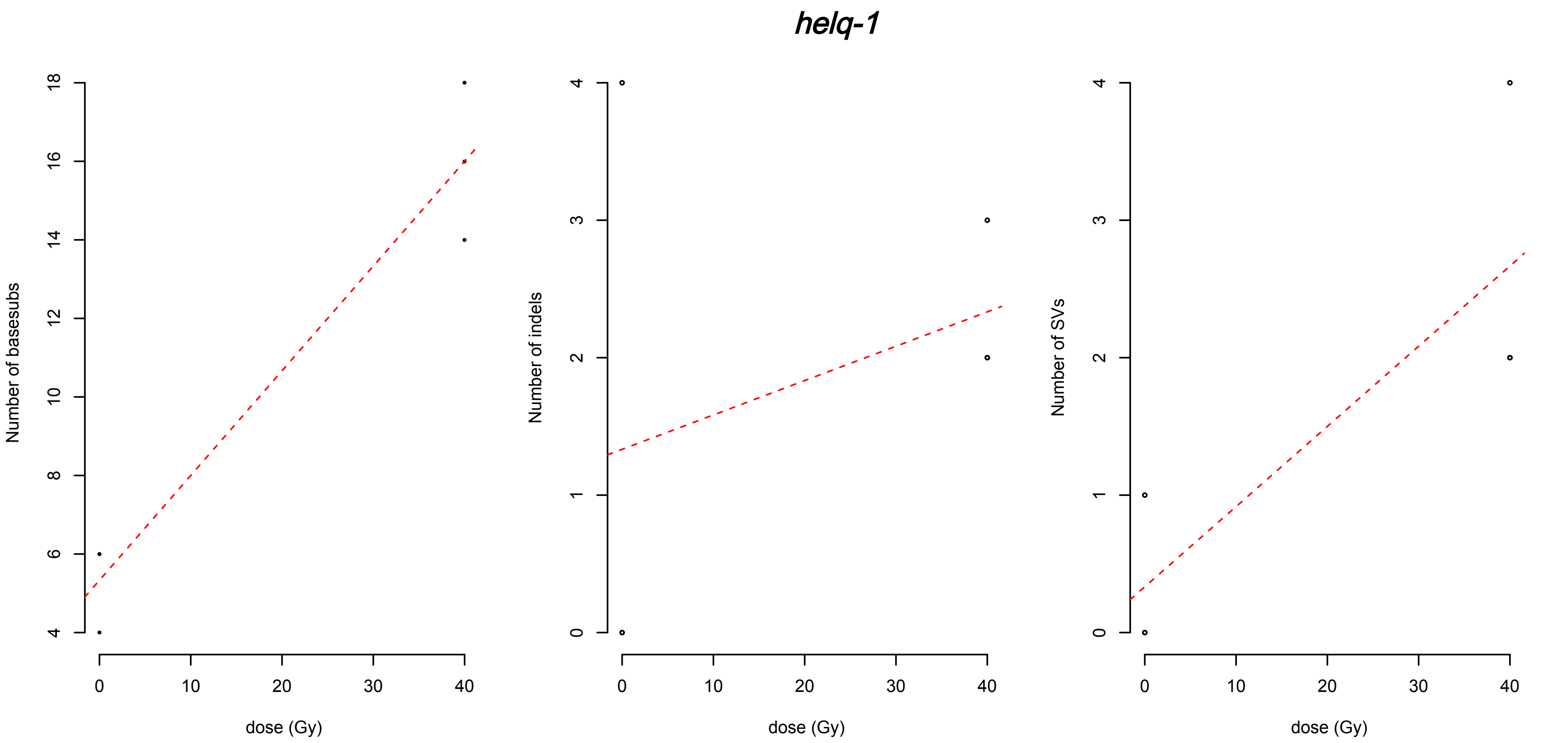

helq-1
Number of basesubs
Number of indels
Number of SVs
dose (Gy)
dose (Gy)
dose (Gy)

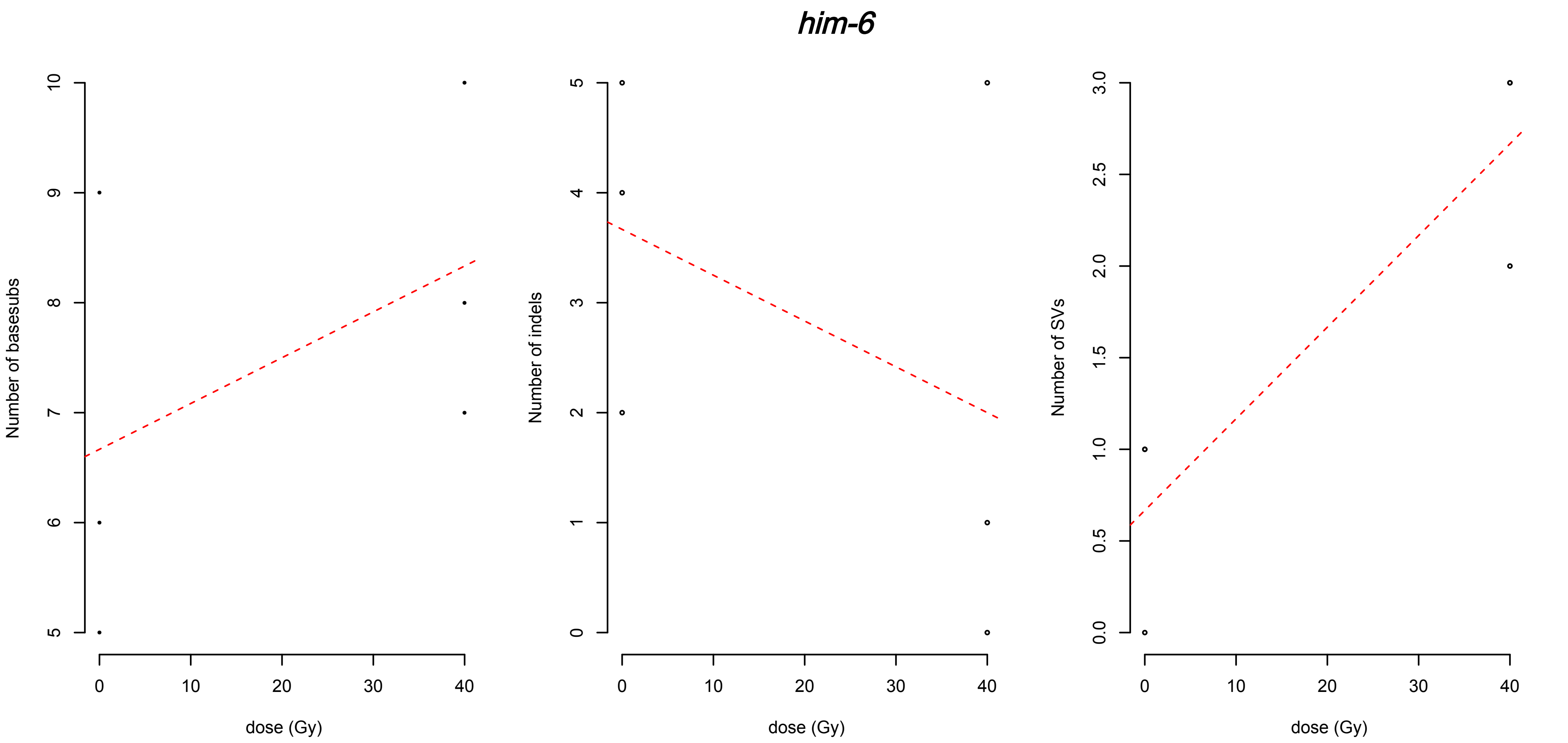

him-6
Number of basesubs
Number of indels
Number of SVs
dose (Gy)
dose (Gy)
dose (Gy)

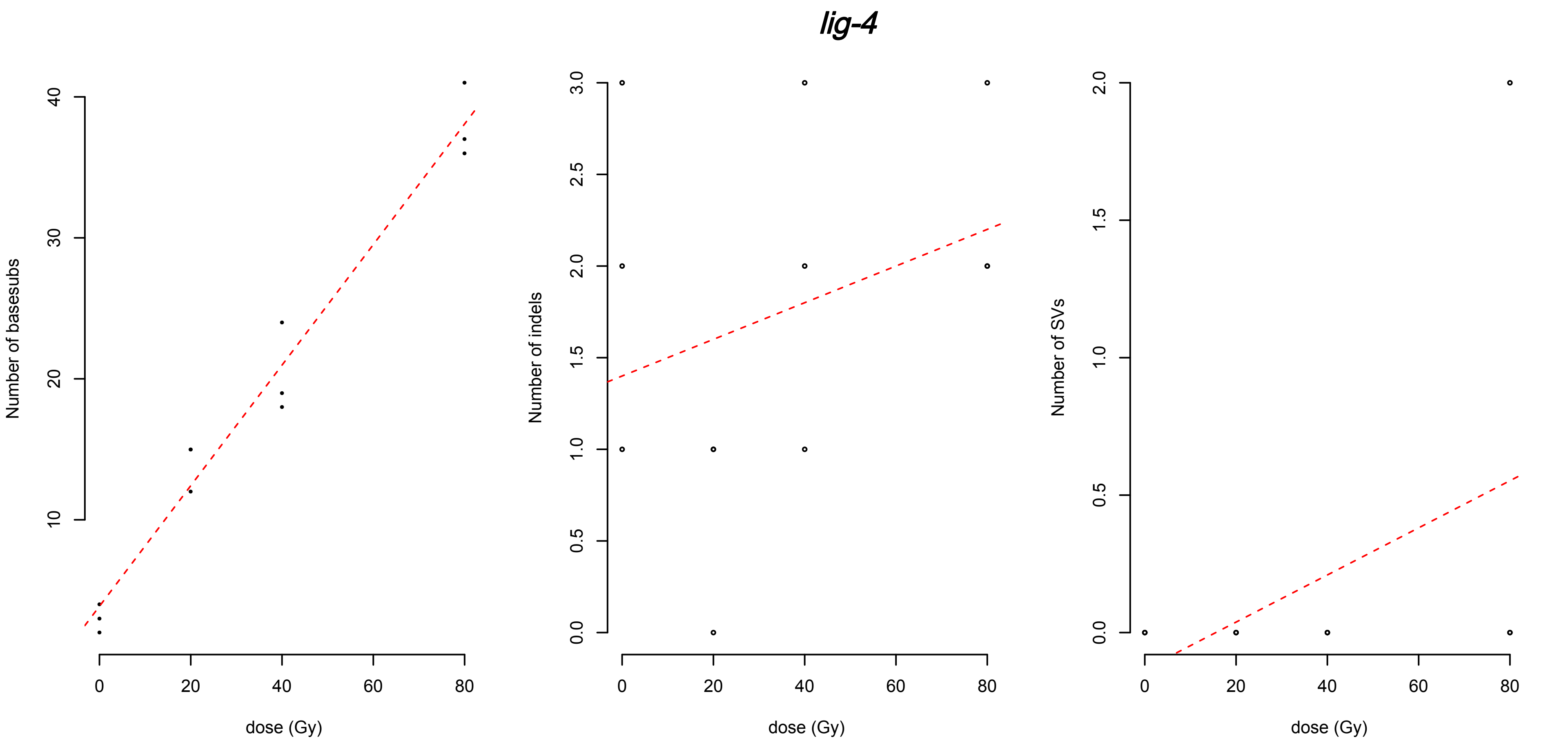
lig-4
Number of basesubs
Number of indels
Number of SVs
dose (Gy)
0
20
40
60
80
10
20
30
40
0.0
0.5
1.0
1.5
2.0
2.5
3.0

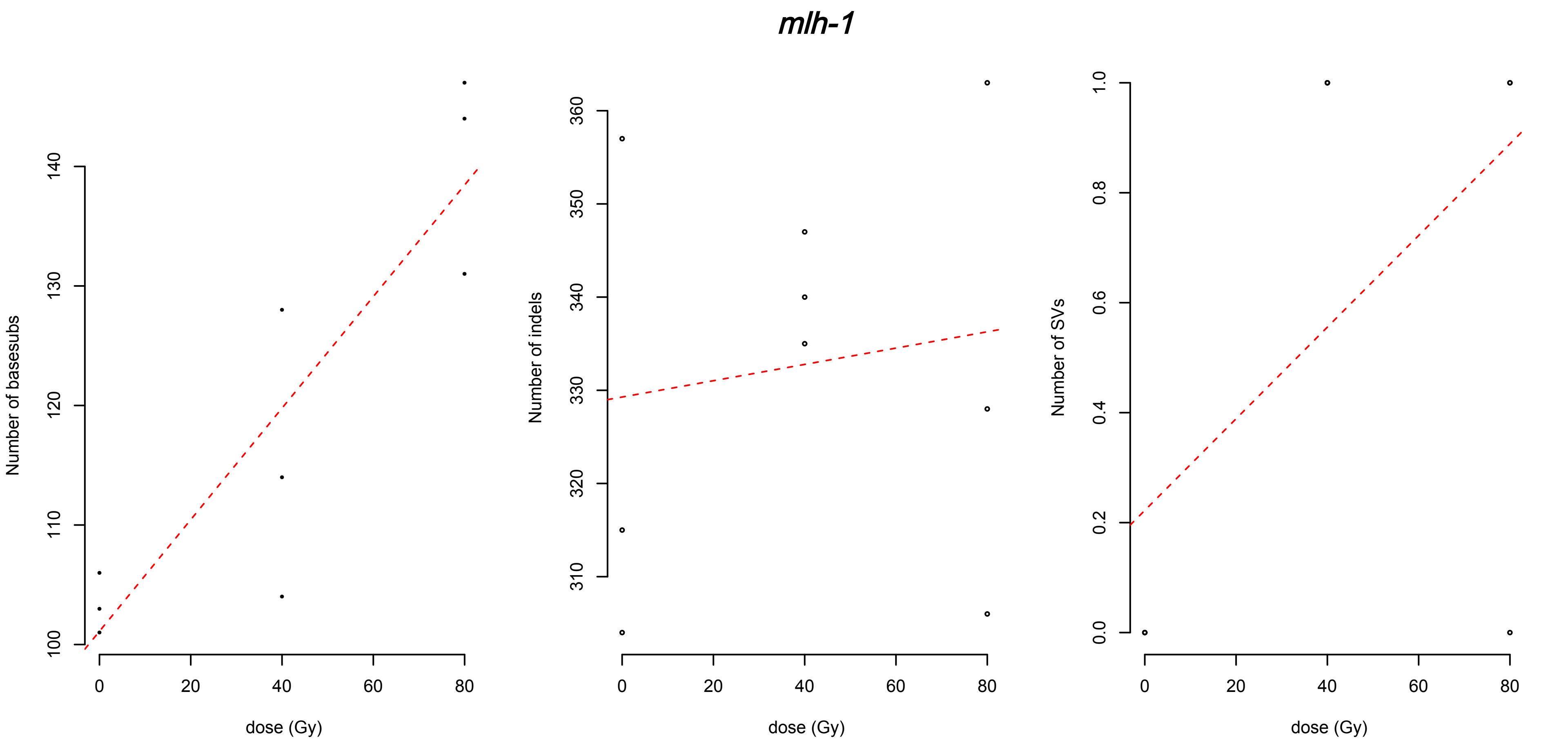

mlh-1
Number of basesubs
100
110
120
130
140
Number of indels
310
320
330
340
350
360
Number of SVs
0.0
0.2
0.4
0.6
0.8
1.0
0
20
40
60
80
dose (Gy)

mrt-2

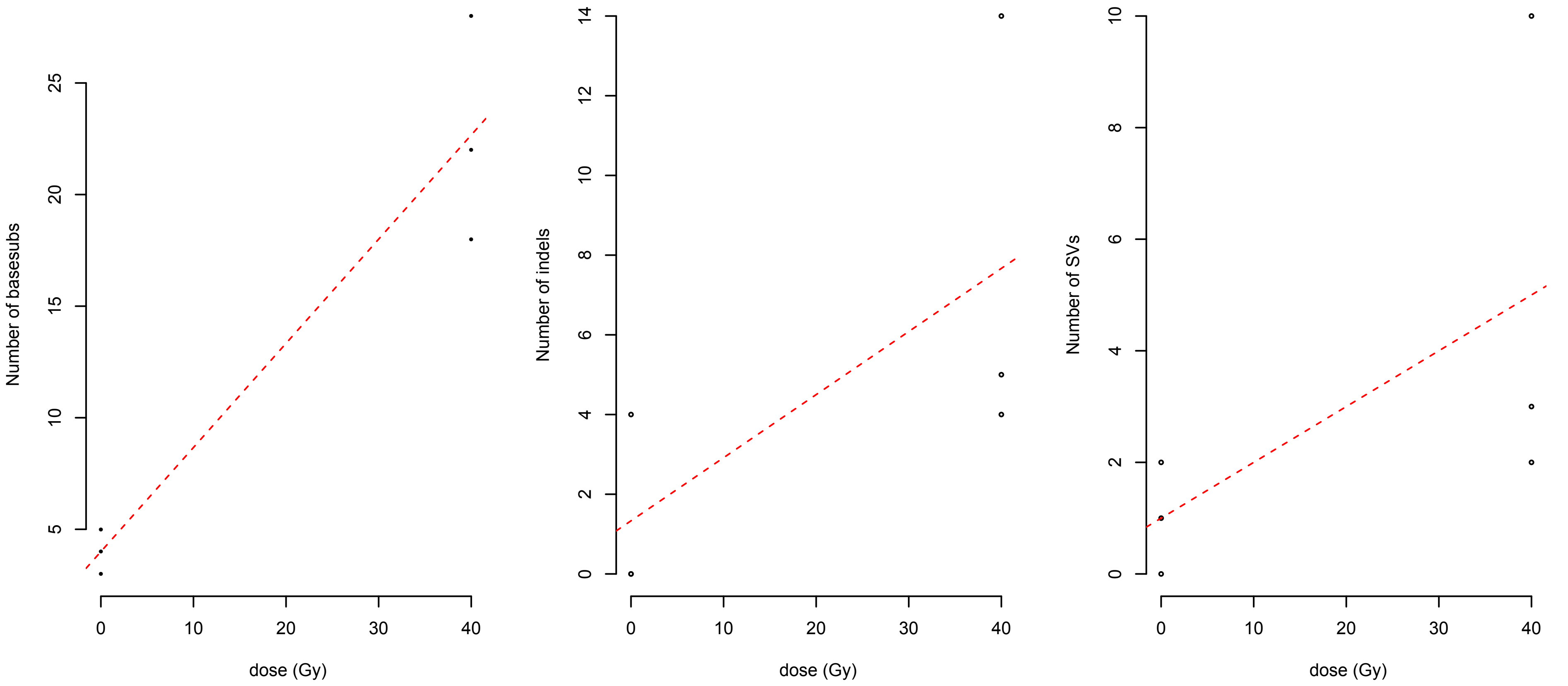

Number of basesubs
25
20
15
10
5
0
10
20
30
40
dose (Gy)
Number of indels
14
12
10
8
6
4
2
0
0
10
20
30
40
dose (Gy)
Number of SVs
10
8
6
4
2
0
0
10
20
30
40
dose (Gy)

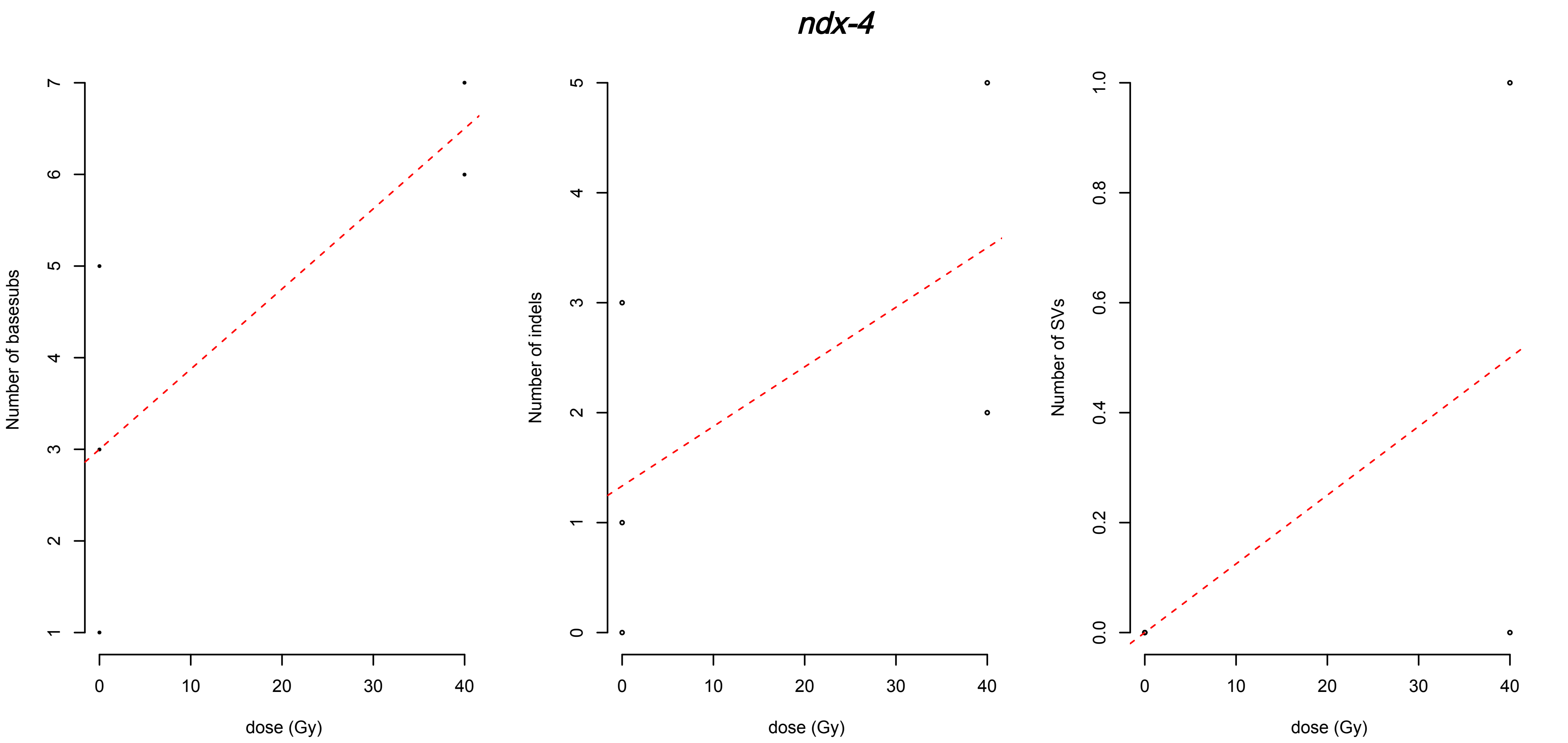
ndx-4
Number of basesubs
dose (Gy)
Number of indels
dose (Gy)
Number of SVs
dose (Gy)
1
2
3
4
5
6
7
0
1
2
3
4
5
0.0
0.2
0.4
0.6
0.8
1.0
0
10
20
30
40

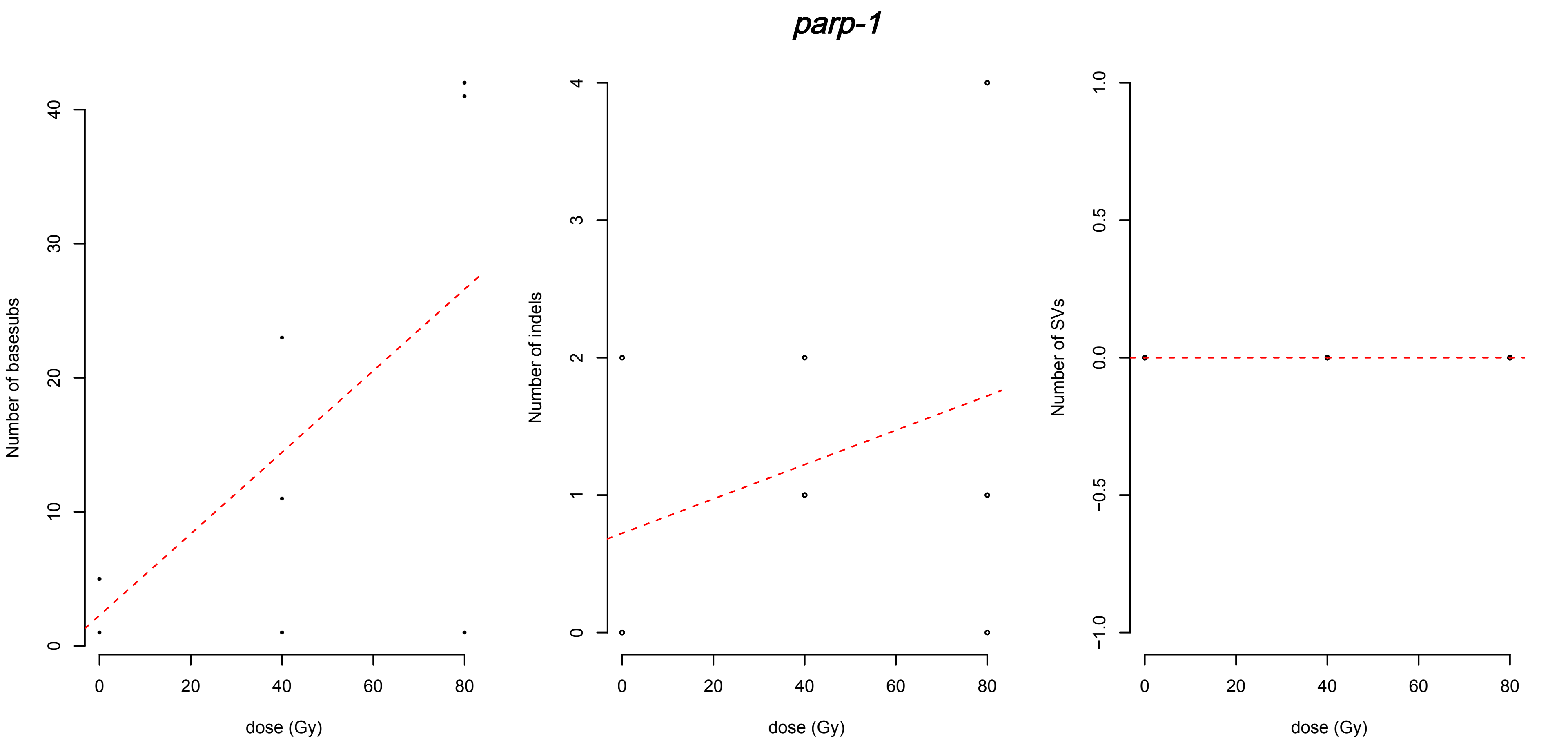
parp-1
Number of basesubs
Number of indels
Number of SVs
dose (Gy)
dose (Gy)
dose (Gy)
0
10
20
30
40
0
1
2
3
4
−1.0
−0.5
0.0
0.5
1.0
0
20
40
60
80

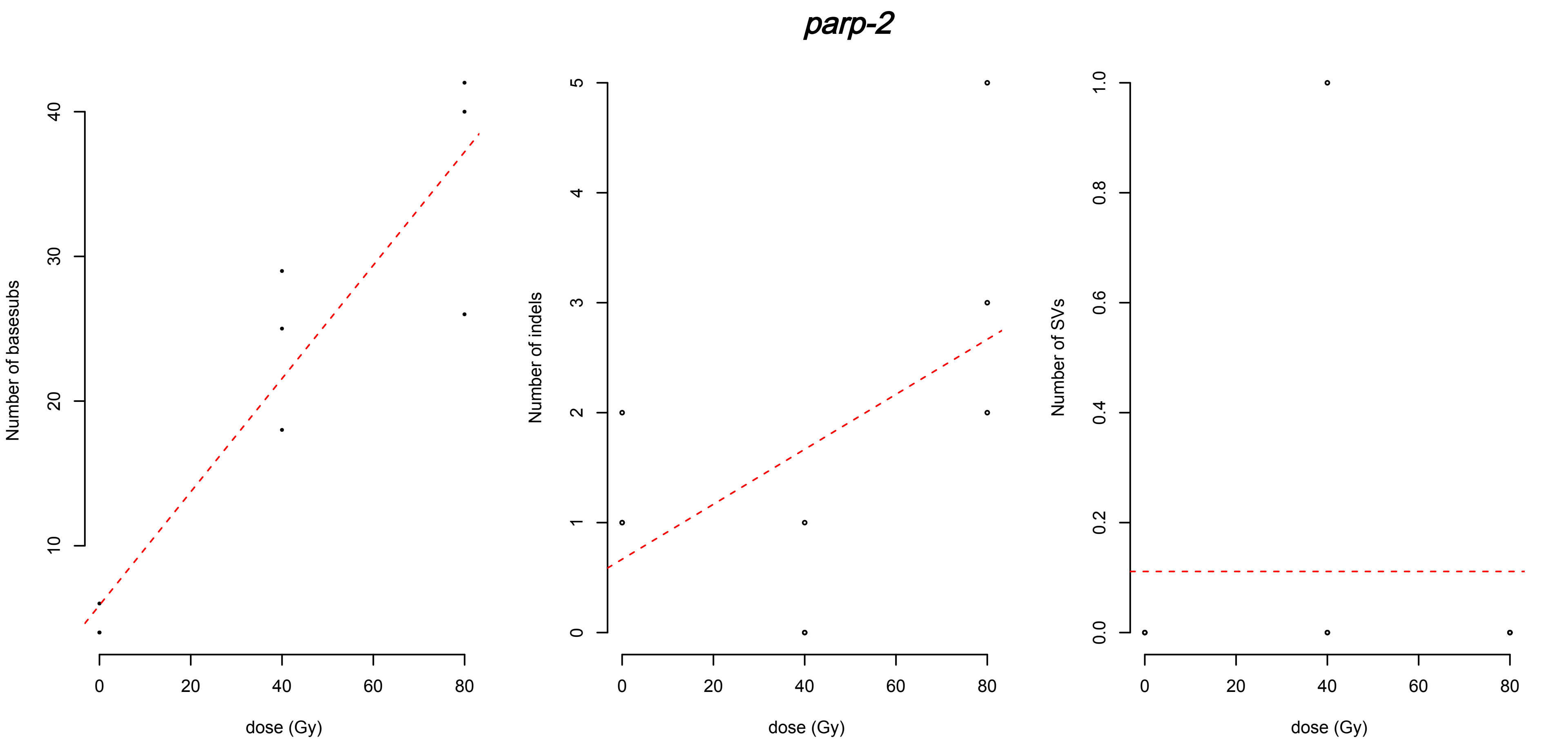

parp-2
Number of basesubs
10
20
30
40
0
20
40
60
80
dose (Gy)
Number of indels
0
1
2
3
4
5
0
20
40
60
80
dose (Gy)
Number of SVs
0.0
0.2
0.4
0.6
0.8
1.0
0
20
40
60
80
dose (Gy)

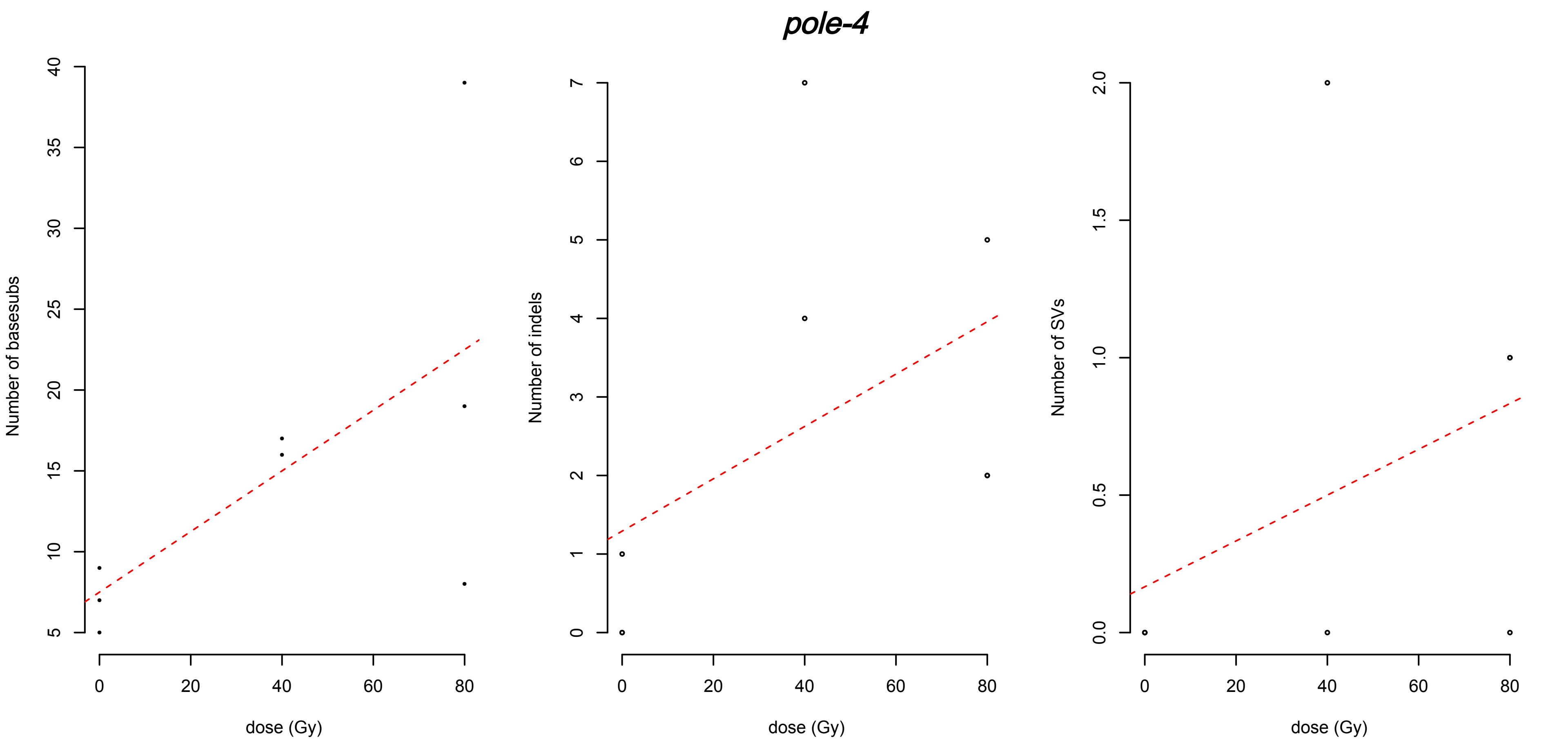
pole-4
Number of basesubs
Number of indels
Number of SVs
dose (Gy)
dose (Gy)
dose (Gy)
5
10
15
20
25
30
35
40
0
1
2
3
4
5
6
7
0.0
0.5
1.0
1.5
2.0
0
20
40
60
80

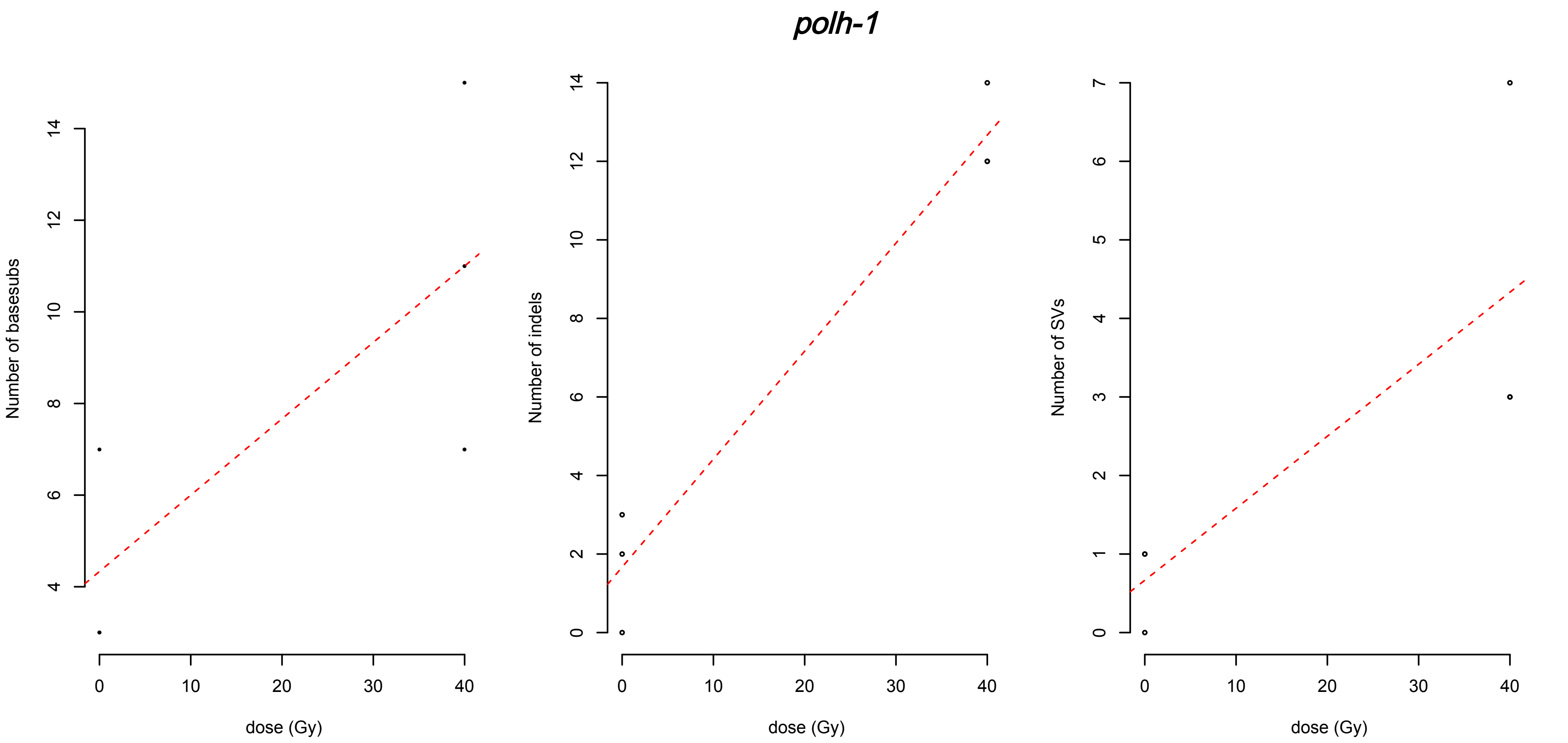

polh-1
Number of basesubs
Number of indels
Number of SVs
dose (Gy)
dose (Gy)
dose (Gy)

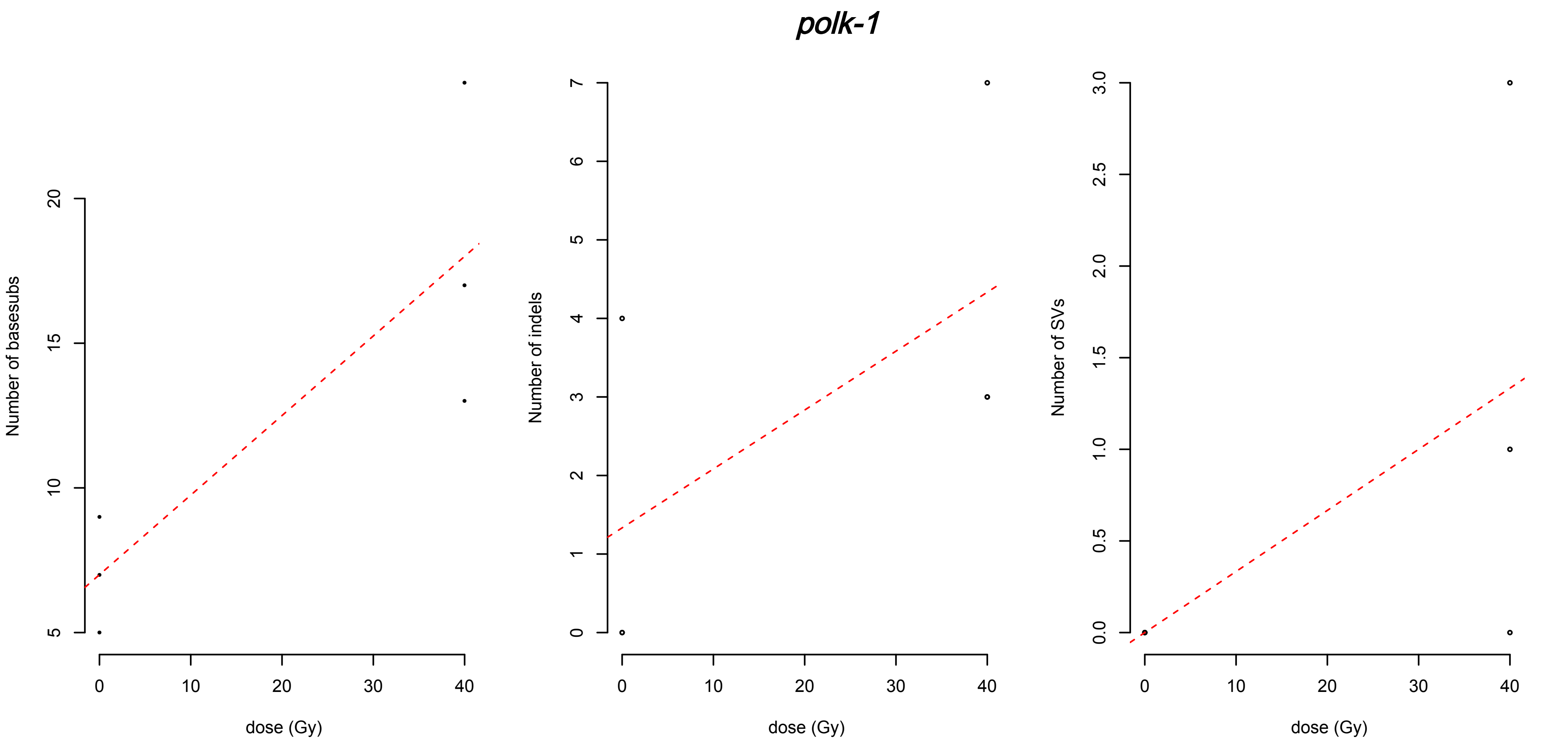

polk-1
Number of basesubs
Number of indels
Number of SVs
dose (Gy)
dose (Gy)
dose (Gy)

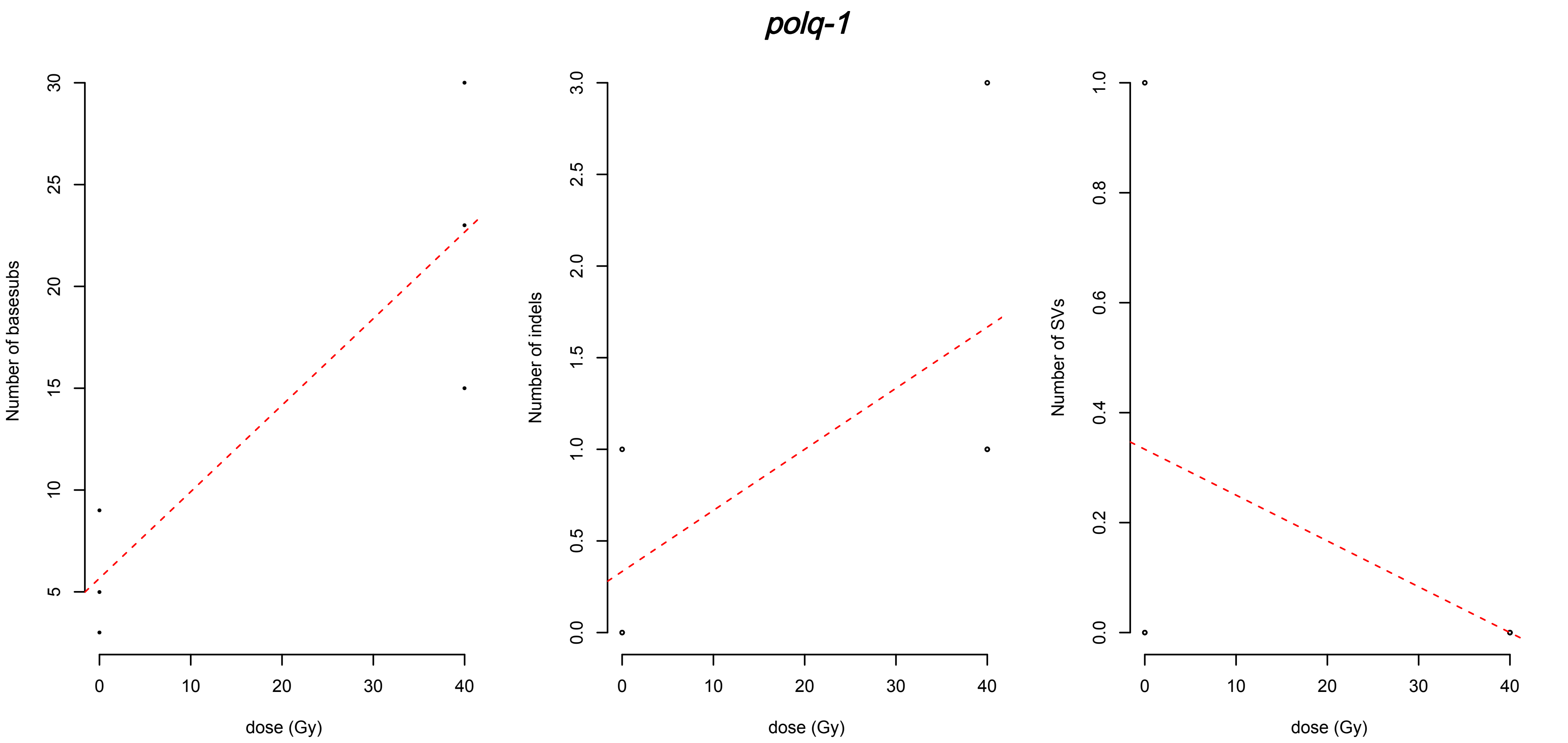
polq-1
Number of basesubs
Number of indels
Number of SVs
dose (Gy)
dose (Gy)
dose (Gy)

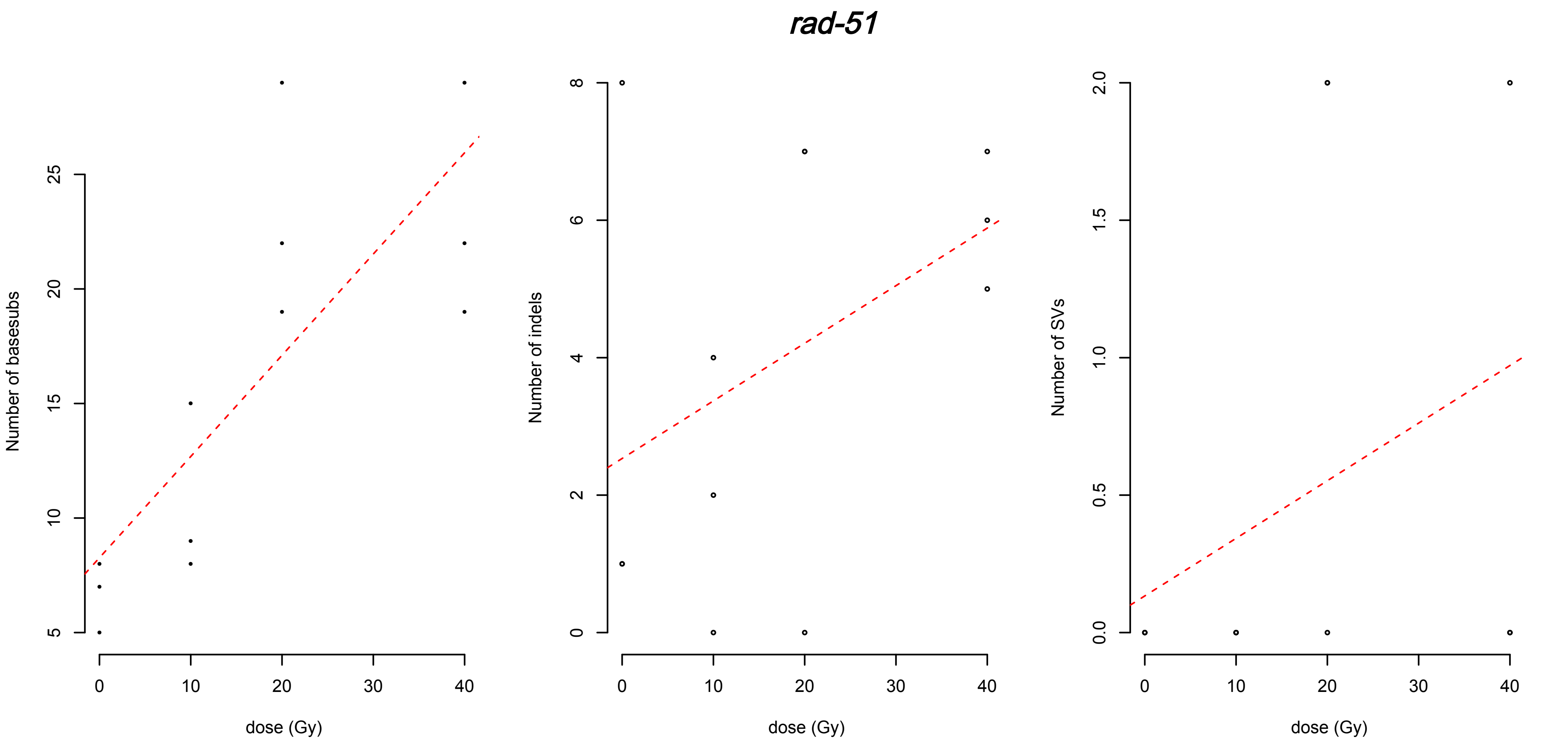

rad-51
Number of basesubs
5
10
15
20
25
0
10
20
30
40
dose (Gy)
Number of indels
0
2
4
6
8
0
10
20
30
40
dose (Gy)
Number of SVs
0.0
0.5
1.0
1.5
2.0
0
10
20
30
40
dose (Gy)

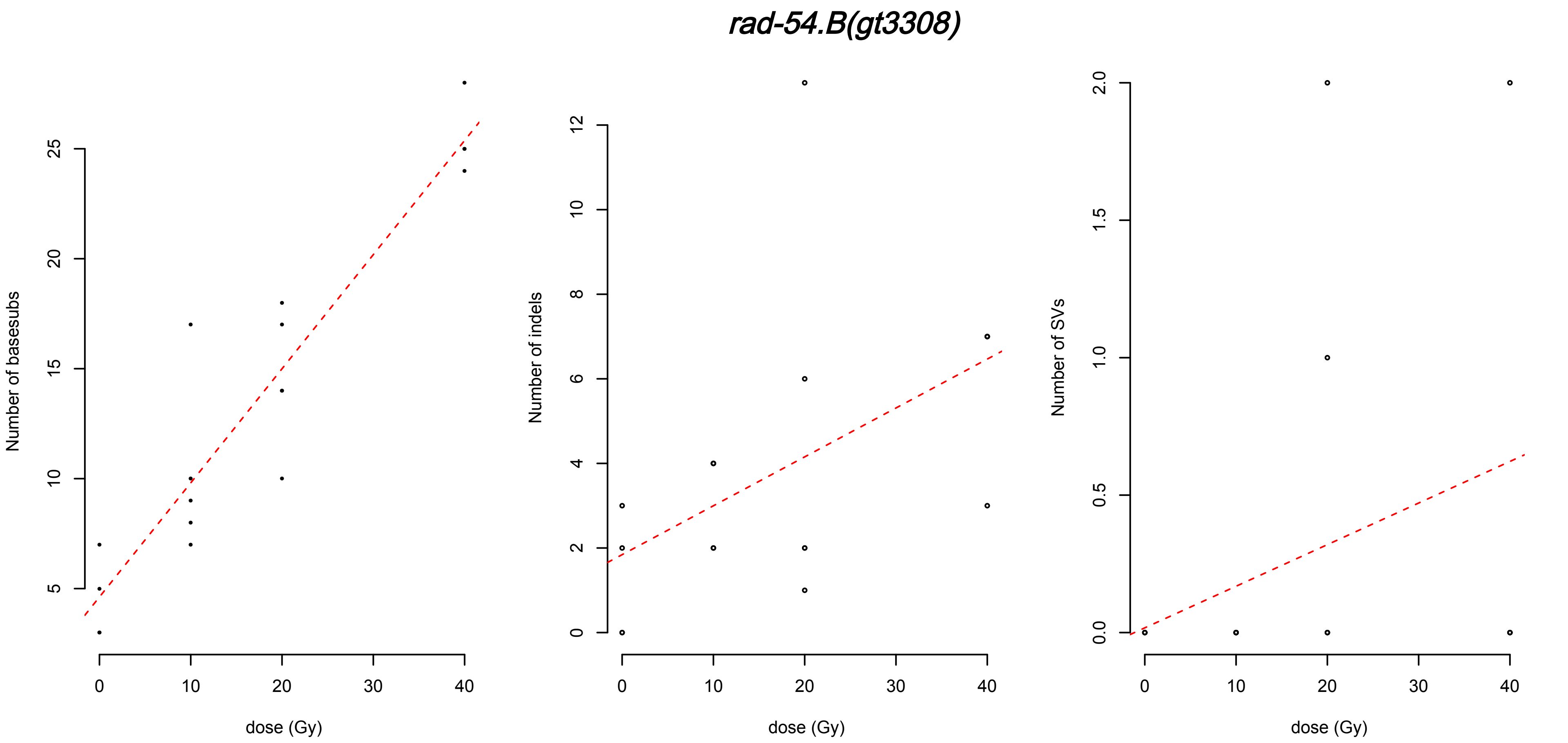
rad-54.B(gt3308)
Number of basesubs
Number of indels
Number of SVs
dose (Gy)
dose (Gy)
dose (Gy)

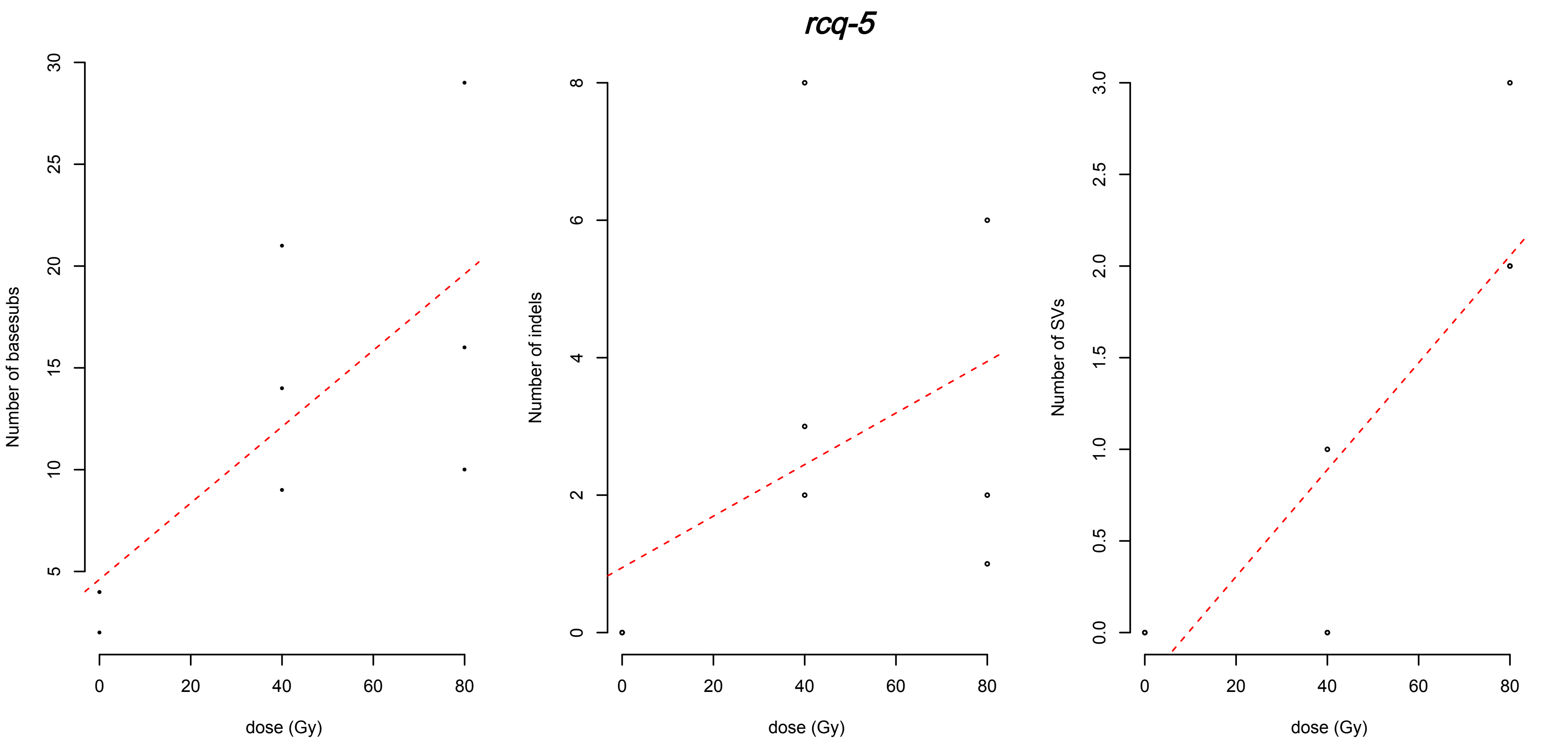

rcq-5
Number of basesubs
30
25
20
15
10
5
0
20
40
60
80
dose (Gy)
Number of indels
8
6
4
2
0
0
20
40
60
80
dose (Gy)
Number of SVs
3.0
2.5
2.0
1.5
1.0
0.5
0.0
0
20
40
60
80
dose (Gy)

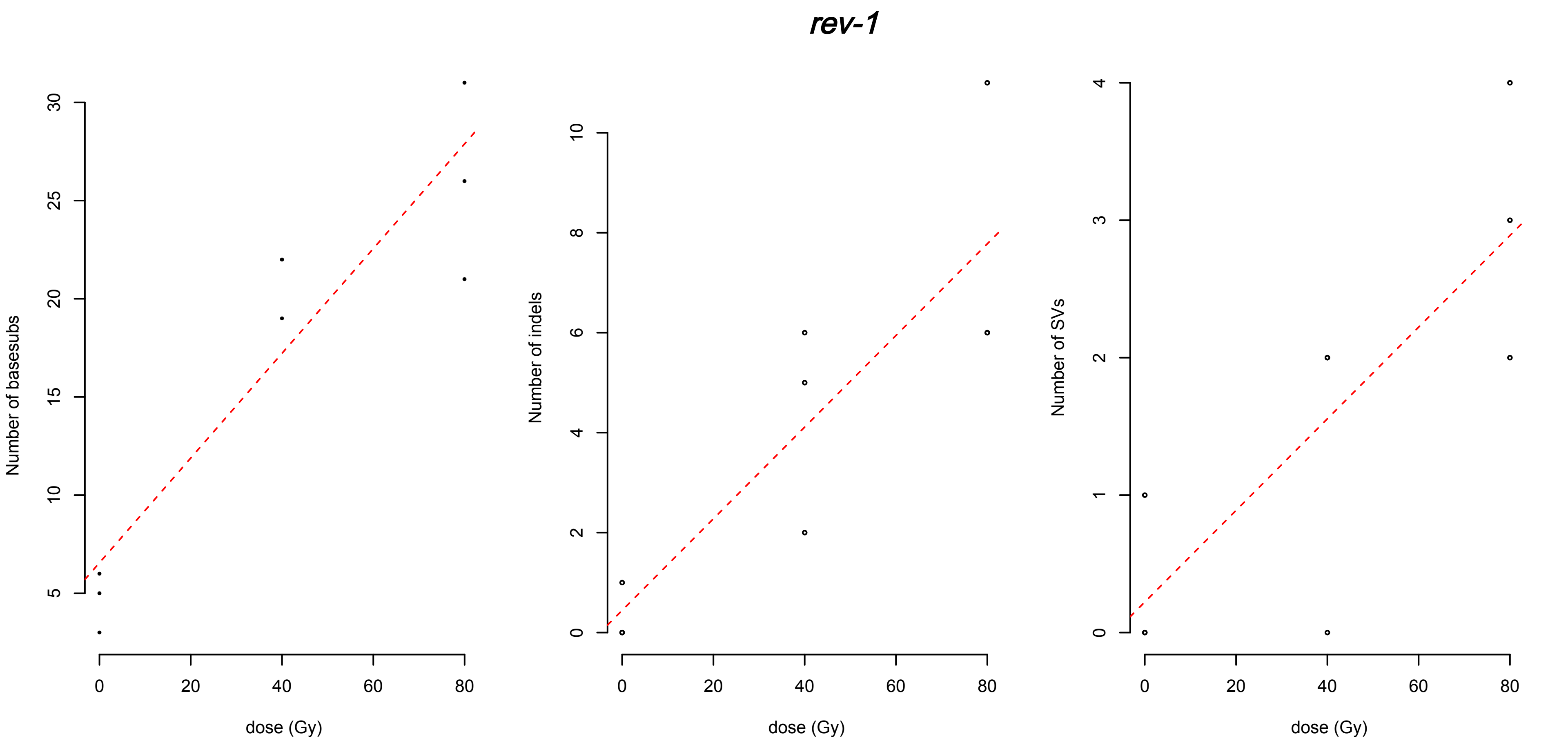

rev-1
Number of basesubs
5
10
15
20
25
30
0
20
40
60
80
dose (Gy)
Number of indels
0
2
4
6
8
10
0
20
40
60
80
dose (Gy)
Number of SVs
0
1
2
3
4
0
20
40
60
80
dose (Gy)

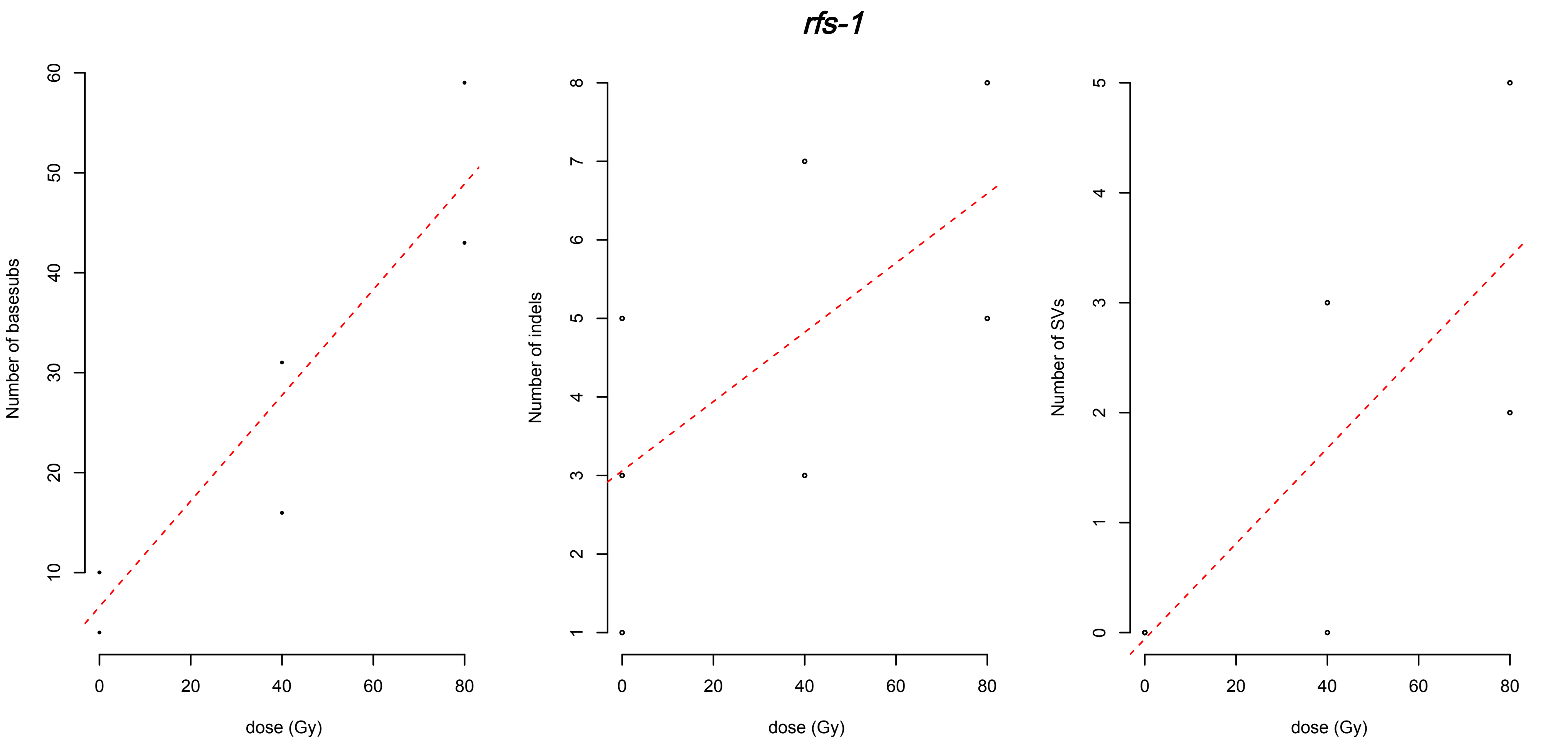
rfs-1
Number of basesubs
Number of indels
Number of SVs
dose (Gy)
dose (Gy)
dose (Gy)

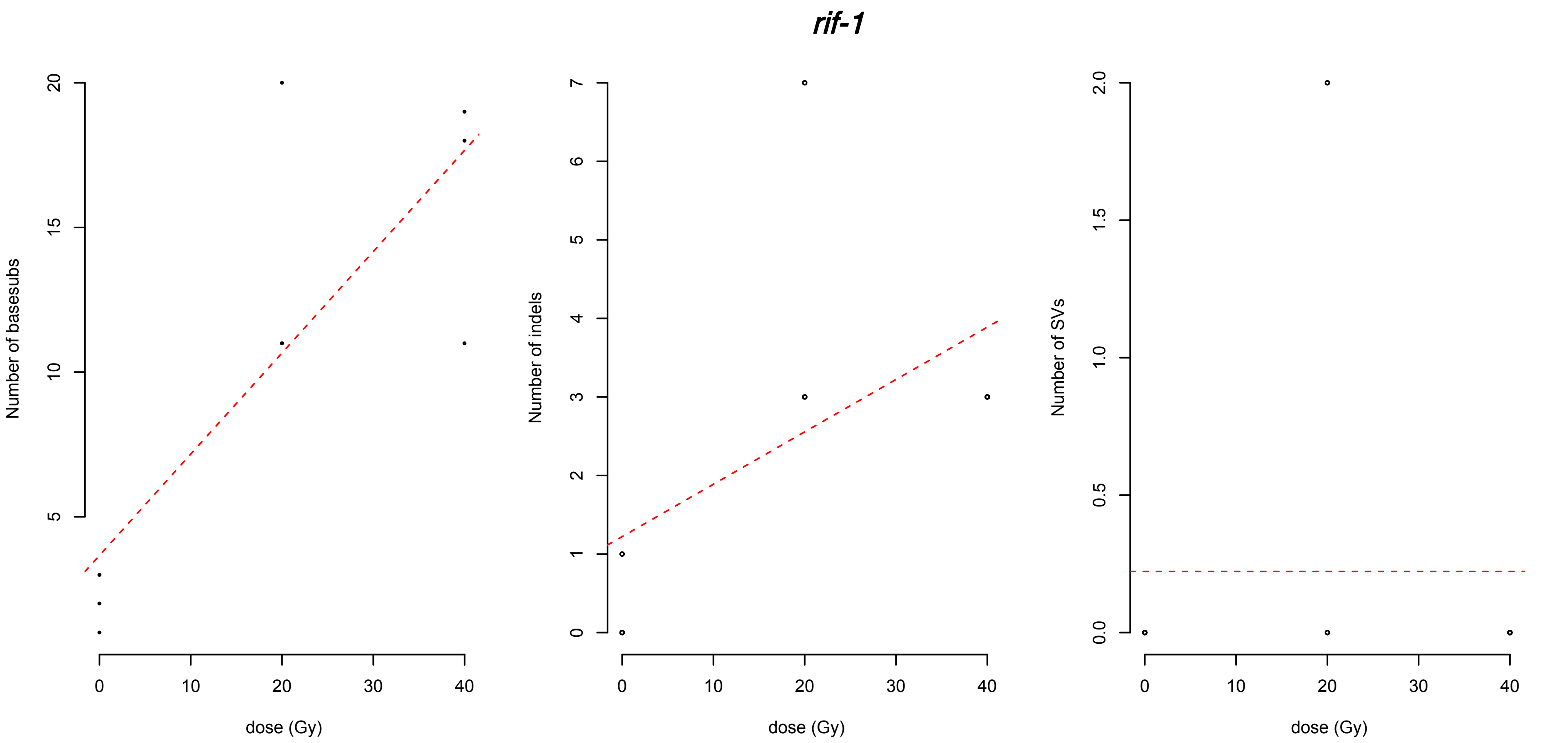
rif-1
Number of basesubs
5
10
15
20
0
10
20
30
40
dose (Gy)
Number of indels
0
1
2
3
4
5
6
7
0
10
20
30
40
dose (Gy)
Number of SVs
0.0
0.5
1.0
1.5
2.0
0
10
20
30
40
dose (Gy)

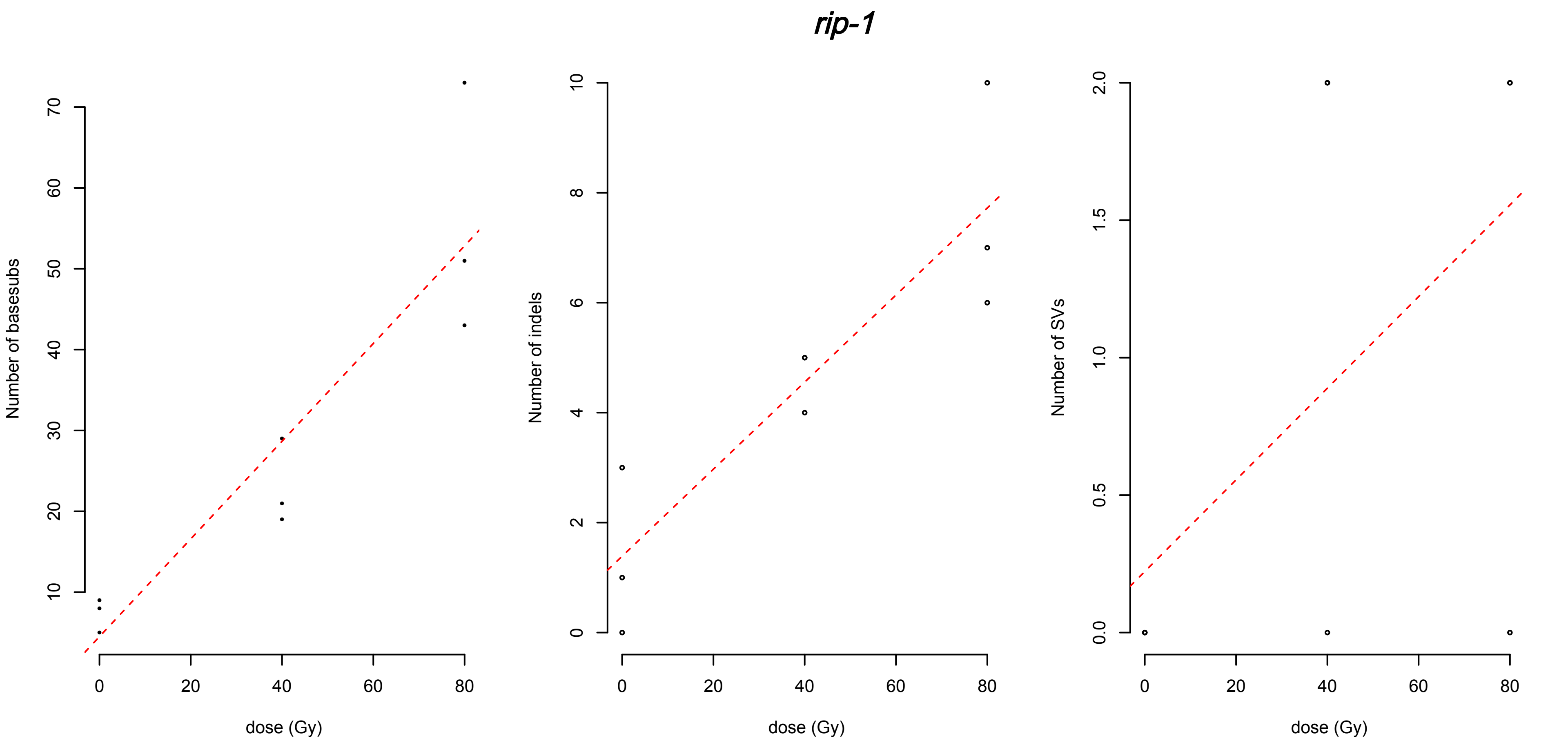

rip-1
Number of basesubs
10
20
30
40
50
60
70
dose (Gy)
0
20
40
60
80
Number of indels
0
2
4
6
8
10
dose (Gy)
0
20
40
60
80
Number of SVs
0.0
0.5
1.0
1.5
2.0
dose (Gy)
0
20
40
60
80

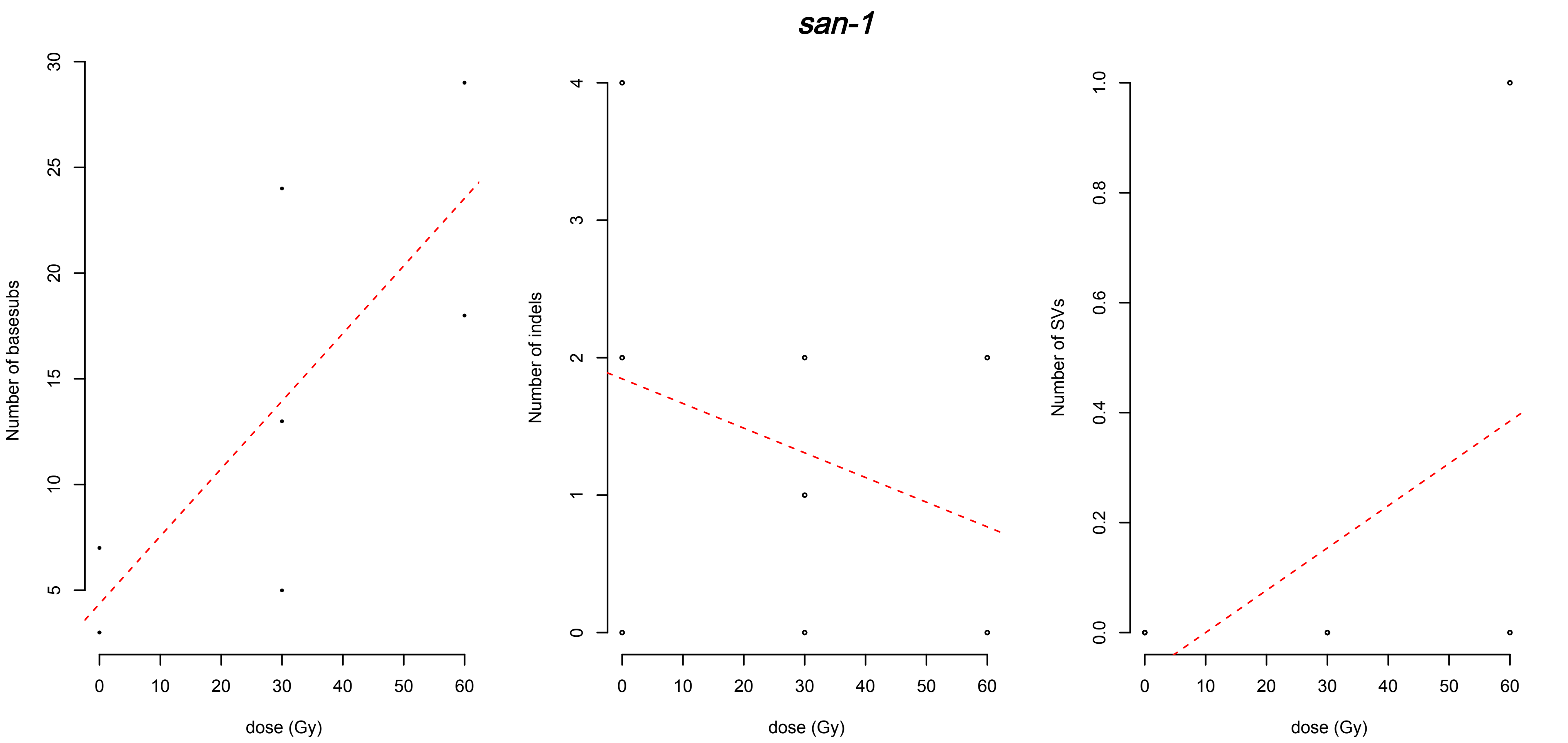
san-1
Number of basesubs
Number of indels
Number of SVs
dose (Gy)
dose (Gy)
dose (Gy)

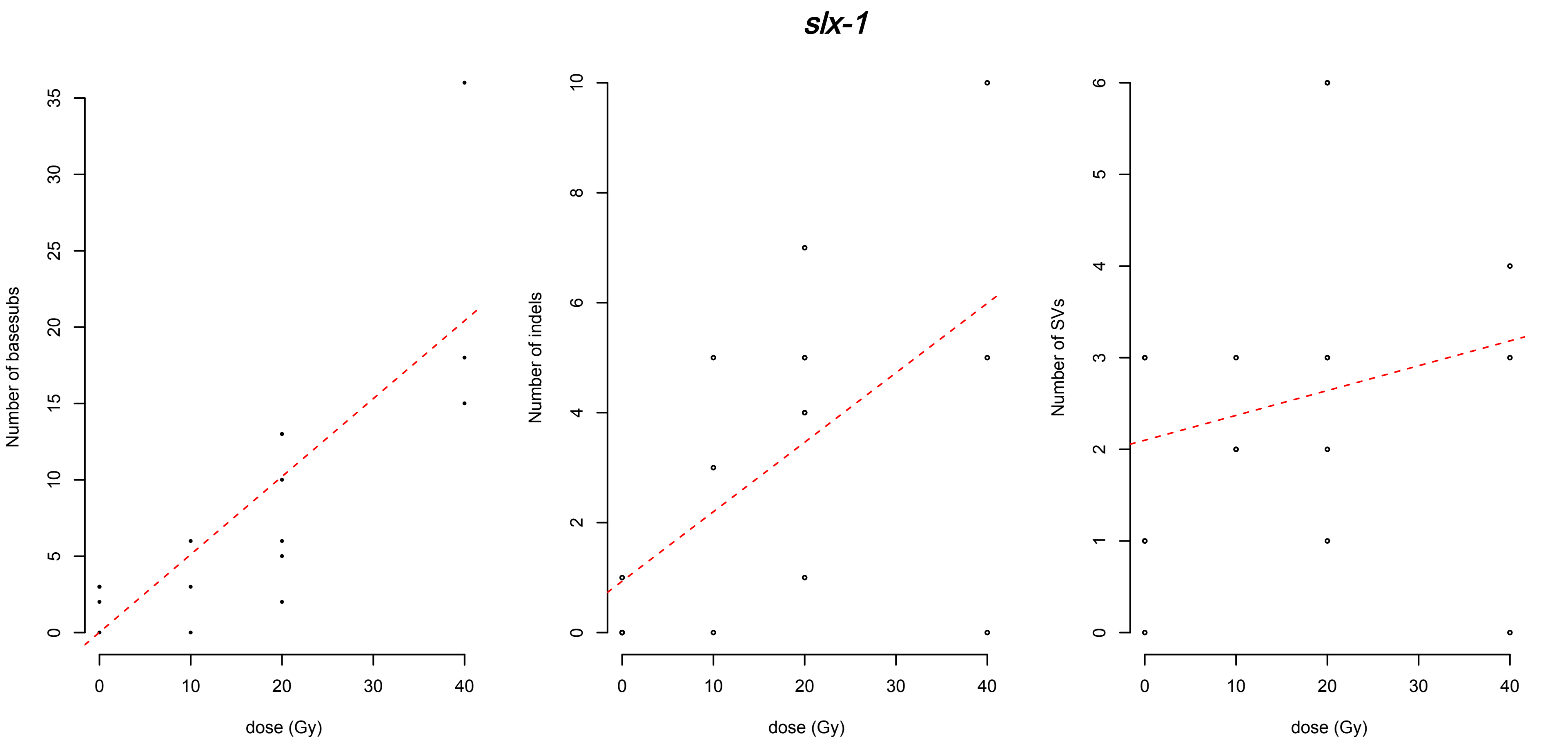
slx-1
Number of basesubs
0
5
10
15
20
25
30
35
dose (Gy)
0
10
20
30
40
Number of indels
0
2
4
6
8
10
dose (Gy)
0
10
20
30
40
Number of SVs
0
1
2
3
4
5
6
dose (Gy)
0
10
20
30
40

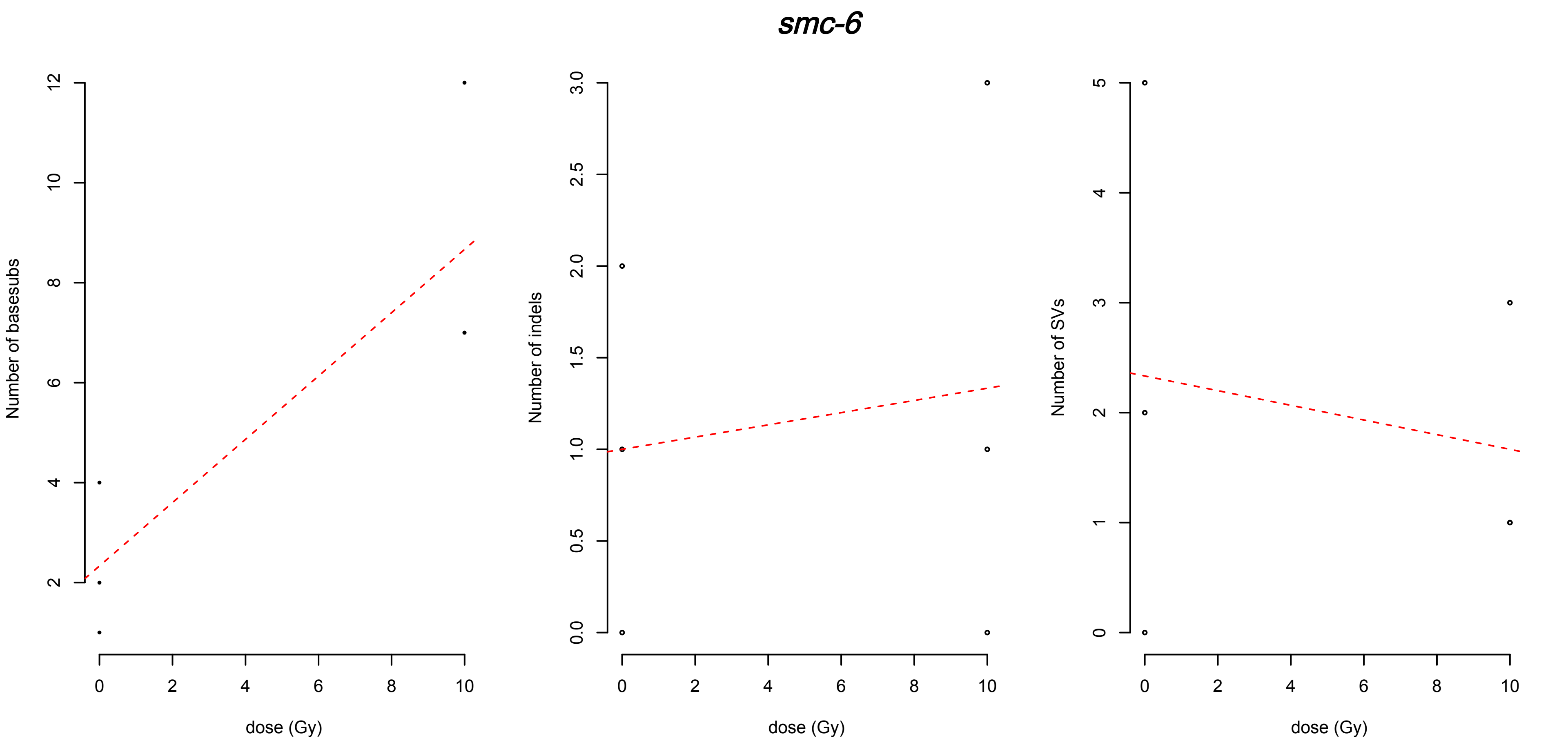

smc-6
Number of basesubs
Number of indels
Number of SVs
dose (Gy)
dose (Gy)
dose (Gy)

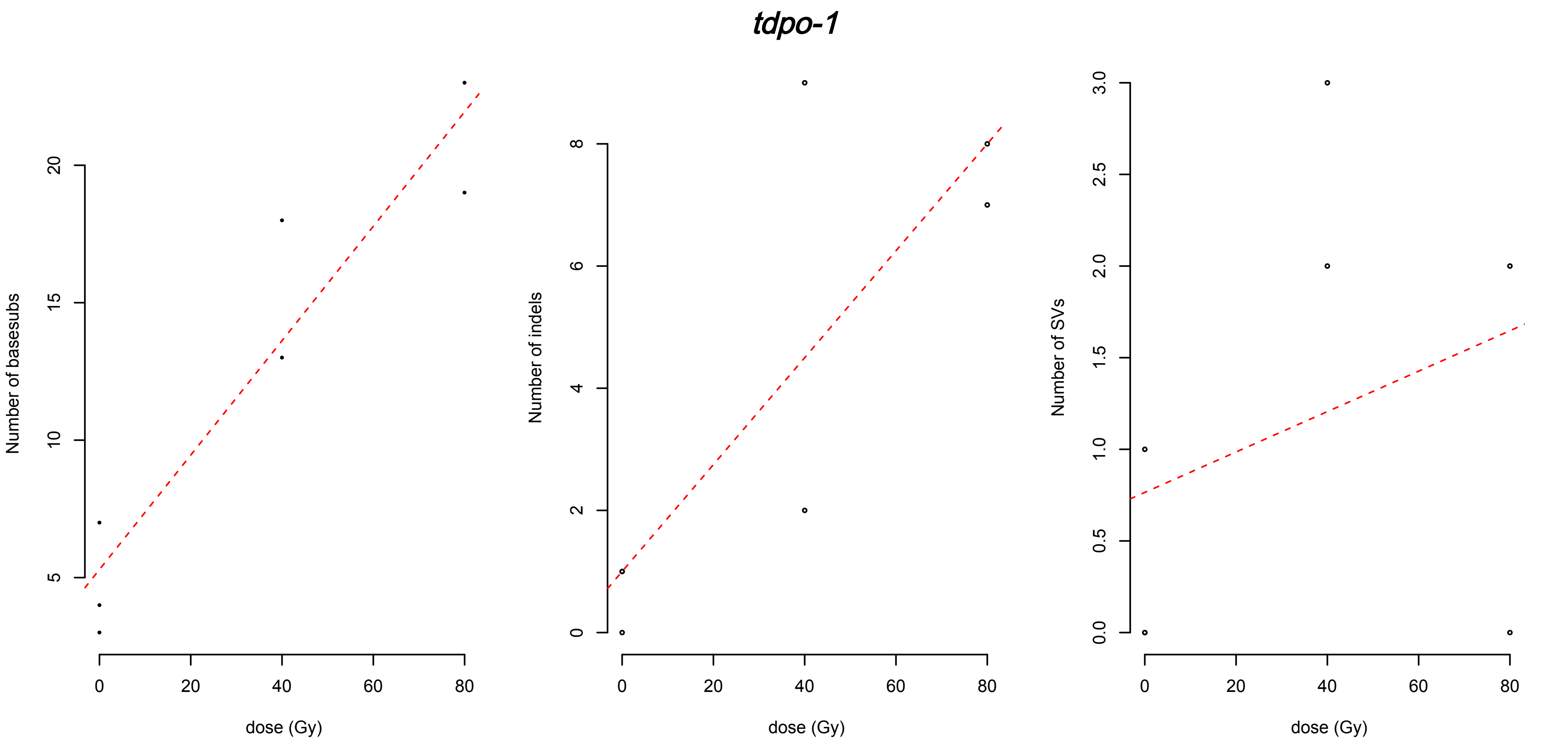

tdpo-1
Number of basesubs
Number of indels
Number of SVs
dose (Gy)
dose (Gy)
dose (Gy)

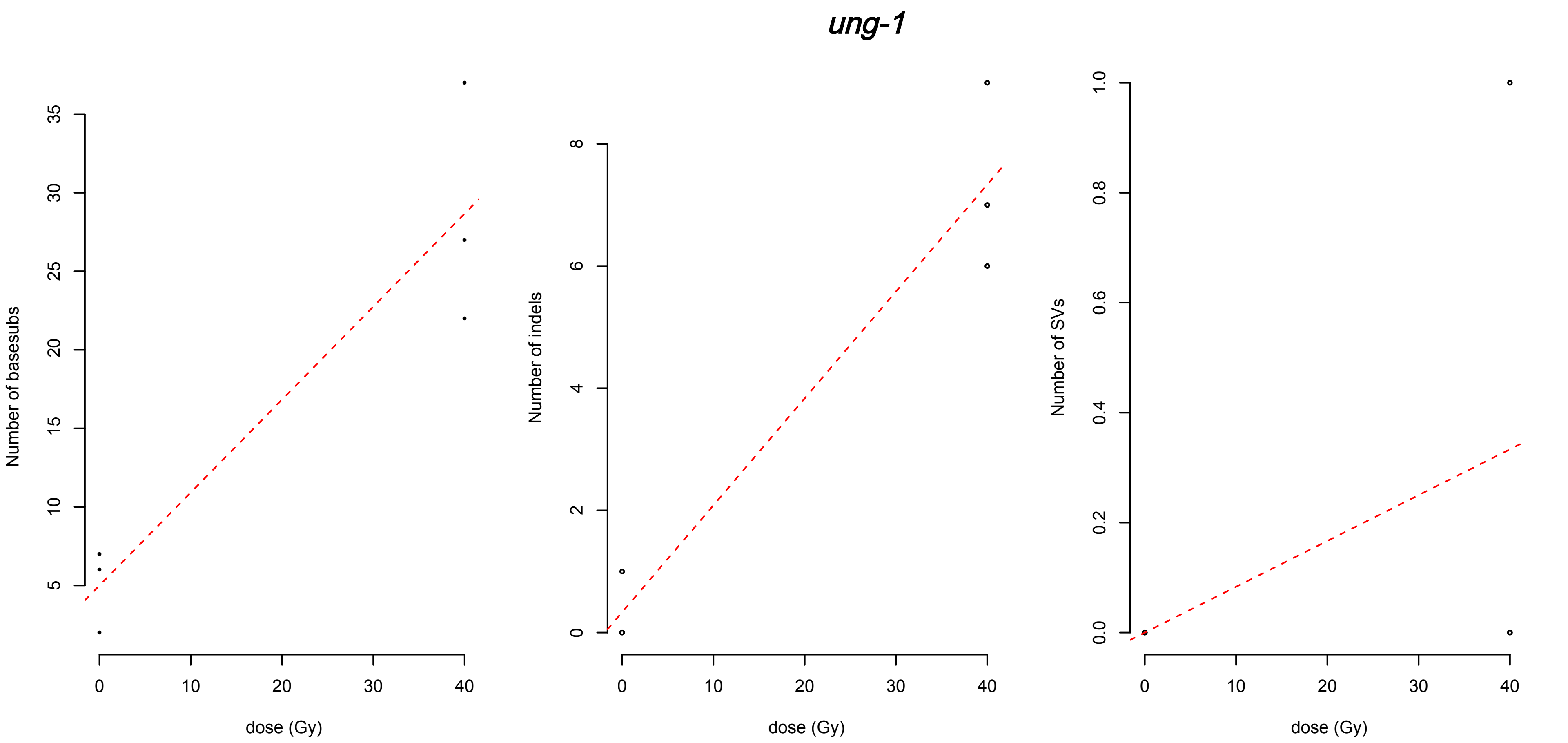

ung-1
Number of basesubs
5
10
15
20
25
30
35
0
10
20
30
40
dose (Gy)
Number of indels
0
2
4
6
8
0
10
20
30
40
dose (Gy)
Number of SVs
0.0
0.2
0.4
0.6
0.8
1.0
0
10
20
30
40
dose (Gy)

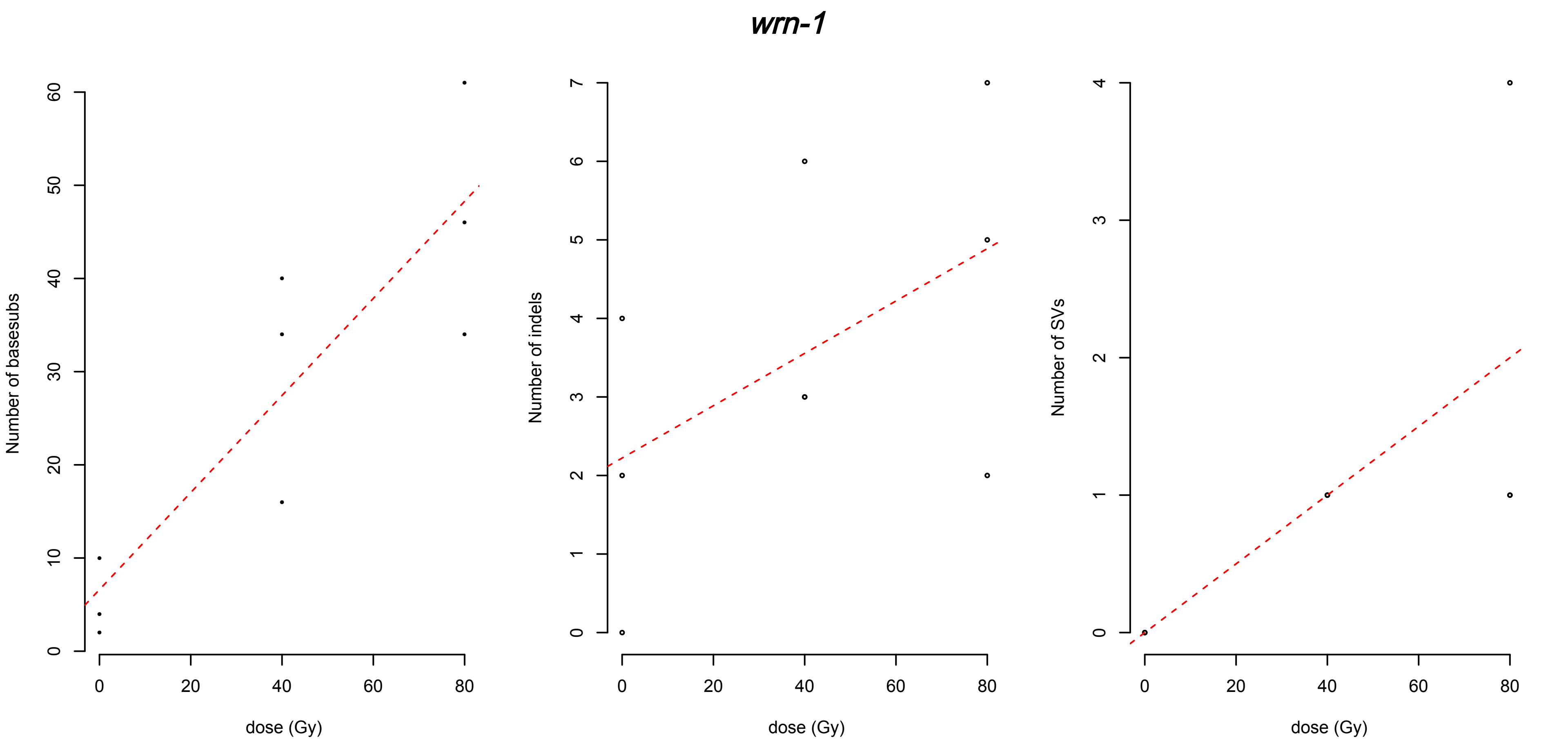

wrn-1
Number of basesubs
dose (Gy)
Number of indels
dose (Gy)
Number of SVs
dose (Gy)

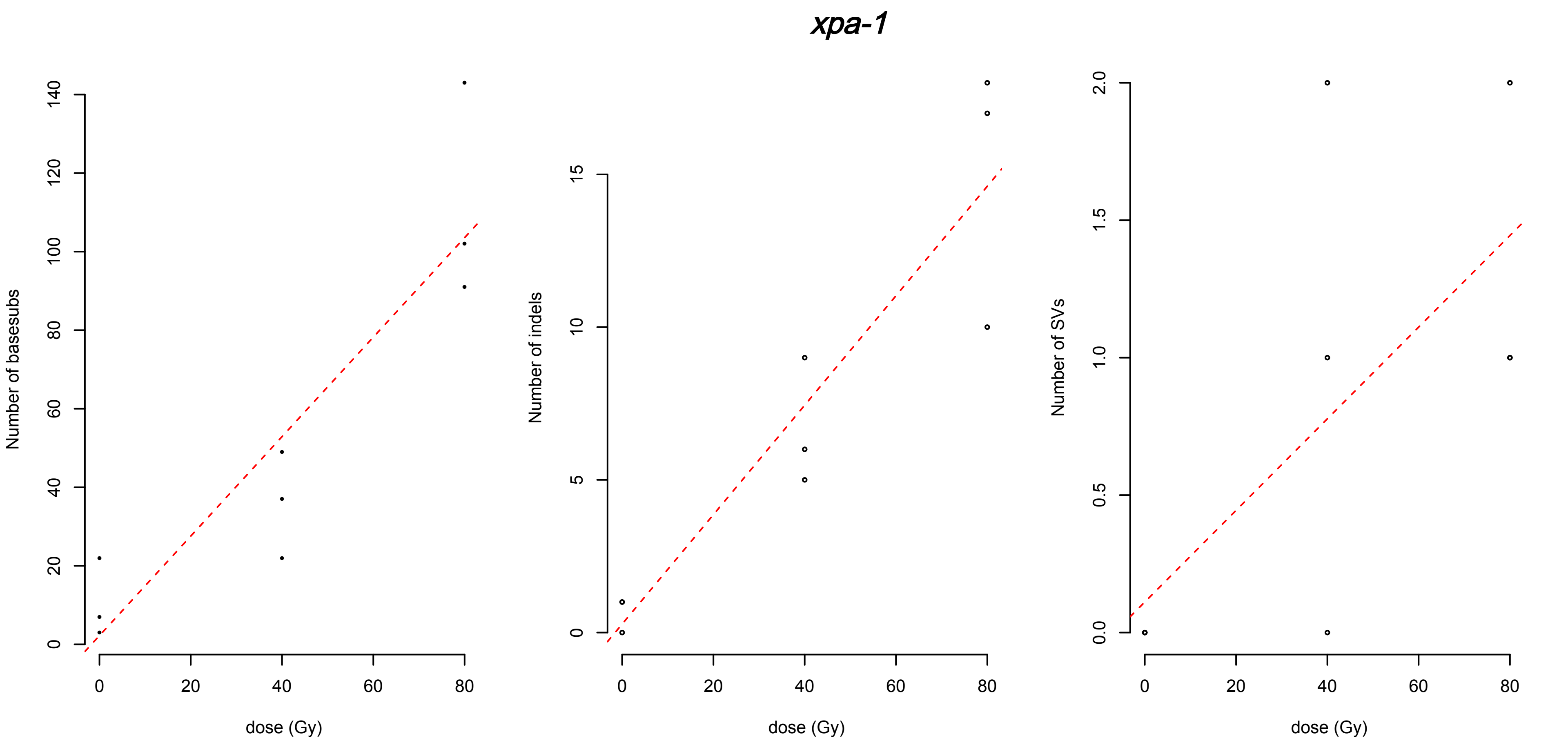

xpa-1
Number of basesubs
Number of indels
Number of SVs
dose (Gy)
dose (Gy)
dose (Gy)

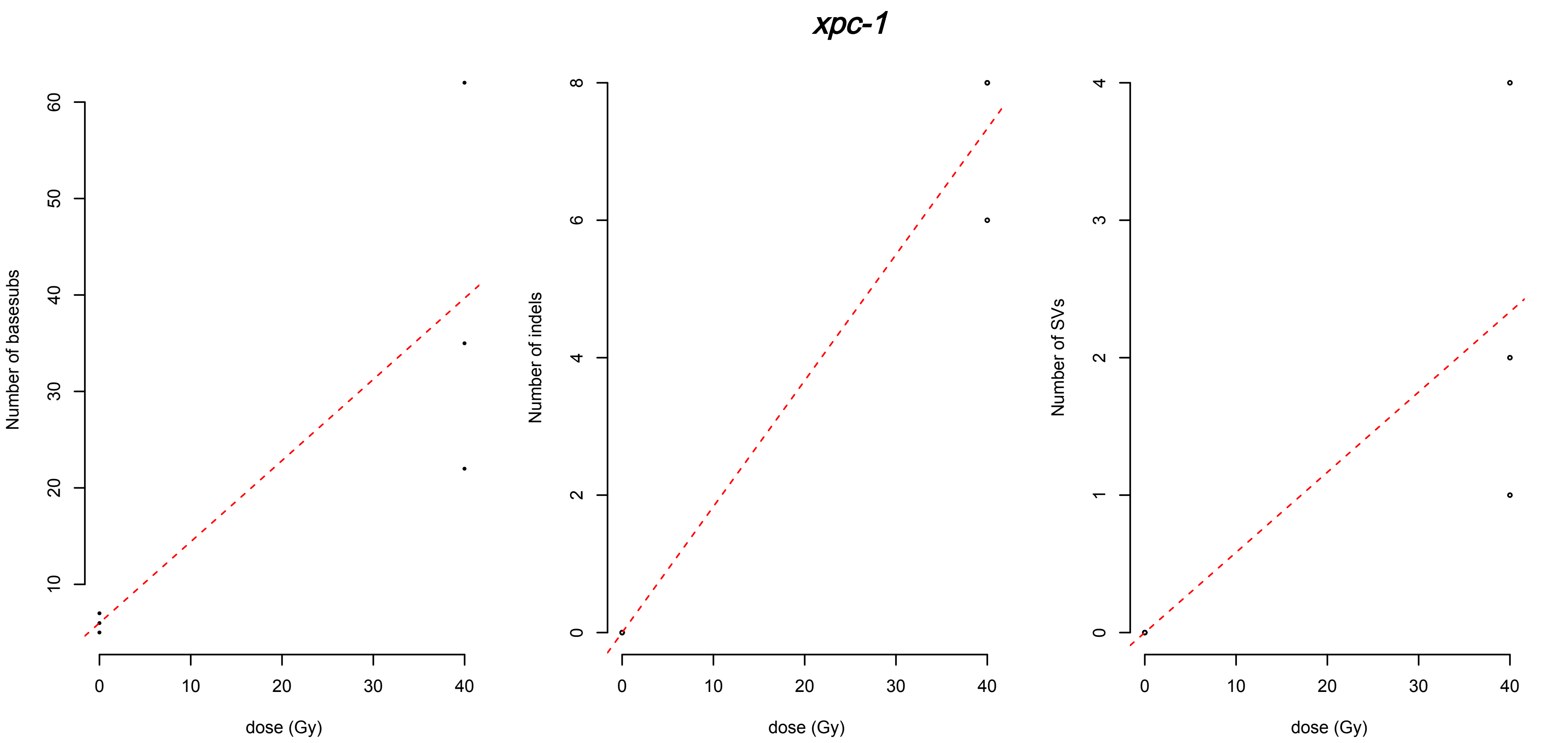

xpc-1
Number of basesubs
10
20
30
40
50
60
0
10
20
30
40
dose (Gy)
Number of indels
0
2
4
6
8
0
10
20
30
40
dose (Gy)
Number of SVs
0
1
2
3
4
0
10
20
30
40
dose (Gy)

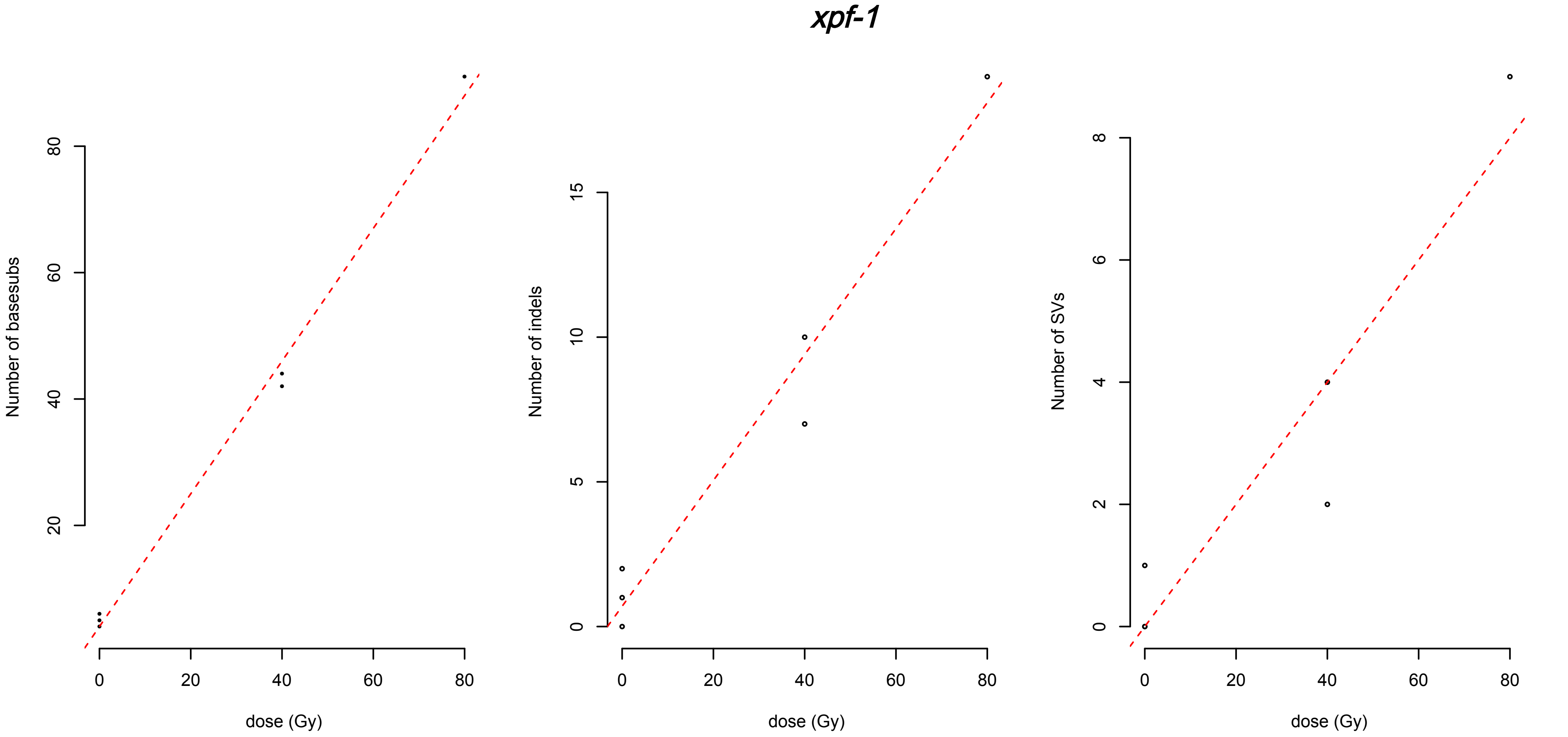

xpf-1
Number of basesubs
80
60
40
20
0
20
40
60
80
dose (Gy)
Number of indels
15
10
5
0
0
20
40
60
80
dose (Gy)
Number of SVs
8
6
4
2
0
0
20
40
60
80
dose (Gy)
