# Supplementary material for: C. elegans genome-wide analysis reveals DNA repair pathways that act cooperatively to preserve genome integrity upon ionizing radiation
Source: PLoS One. 2021 Oct 6;16(10):e0258269. doi: 10.1371/journal.pone.0258269 (PMC8494335; doi:10.1371/journal.pone.0258269)
Supplement: S2 File — Each barplot reflects the number of mutations per average dose of 80 Gy of Cs-137-radiation. Three stars indicate samples with SNVs, MNV, indels, or SV significantly different to wild-type (FDR < 5%). Bold lines below a mutation class indicate that a specific substitution type with the above classes differs from wild-type (FDR < 5%). (PDF) [file pone.0258269.s012.pdf]

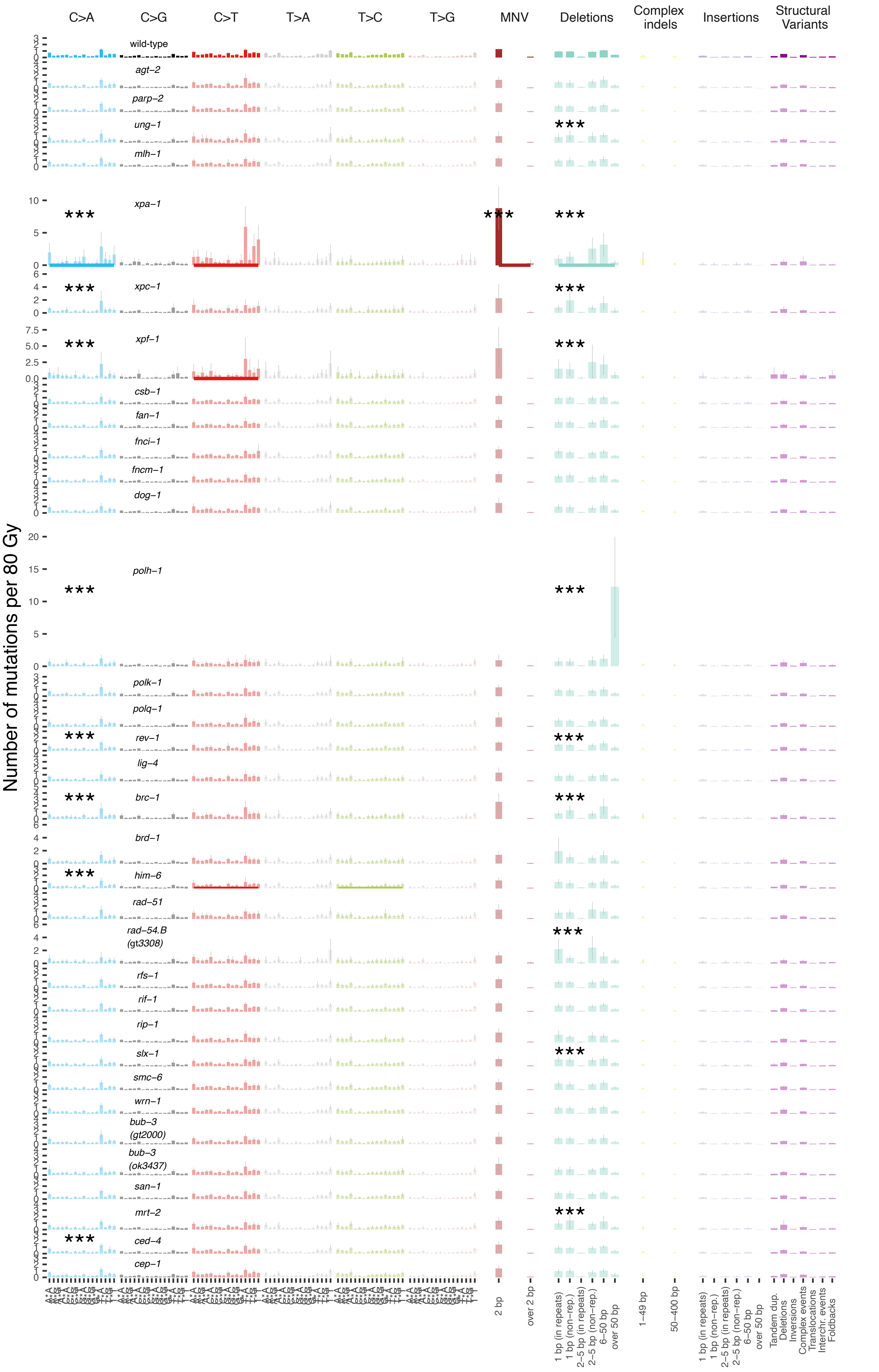

C>A
C>G
C>T
T>A
T>C
T>G
MNV
Deletions
Complex indels
Insertions
Structural Variants
Number of mutations per 80 Gy
wild-type
agt-2
parp-2
ung-1
mlh-1
xpa-1
xpc-1
xpf-1
csb-1
fan-1
fnci-1
fncm-1
dog-1
polh-1
polk-1
polq-1
rev-1
lig-4
brc-1
brd-1
him-6
rad-51
rad-54.B (gt3308)
rfs-1
rif-1
rip-1
slx-1
smc-6
wrn-1
bub-3 (gt2000)
bub-3 (ok3437)
san-1
mrt-2
ced-4
cep-1
***
2 bp
over 2 bp
1 bp (in repeats)
1 bp (non-rep.)
2–5 bp (in repeats)
2–5 bp (non-rep.)
6–50 bp
over 50 bp
1–49 bp
50–400 bp
Tandem dup.
Deletions
Inversions
Complex events
Translocations
Interchr. events
Foldbacks
