# Supplementary material for: C. elegans genome-wide analysis reveals DNA repair pathways that act cooperatively to preserve genome integrity upon ionizing radiation
Source: PLoS One. 2021 Oct 6;16(10):e0258269. doi: 10.1371/journal.pone.0258269 (PMC8494335; doi:10.1371/journal.pone.0258269)
Supplement: S3 File — Chromosomal location of mutations observed after exposure to the indicated radiation doses across the 5 C. elegans autosomes (I-V) and the X chromosome for all genotypes. Small circles, triangles, and squares, indicate single SNVs, indels, and DNVs, respectively. Larger circles, triangles, and squares, indicate clustered SNVs, indels, and DNVs, respectively. (PDF) [file pone.0258269.s013.pdf]

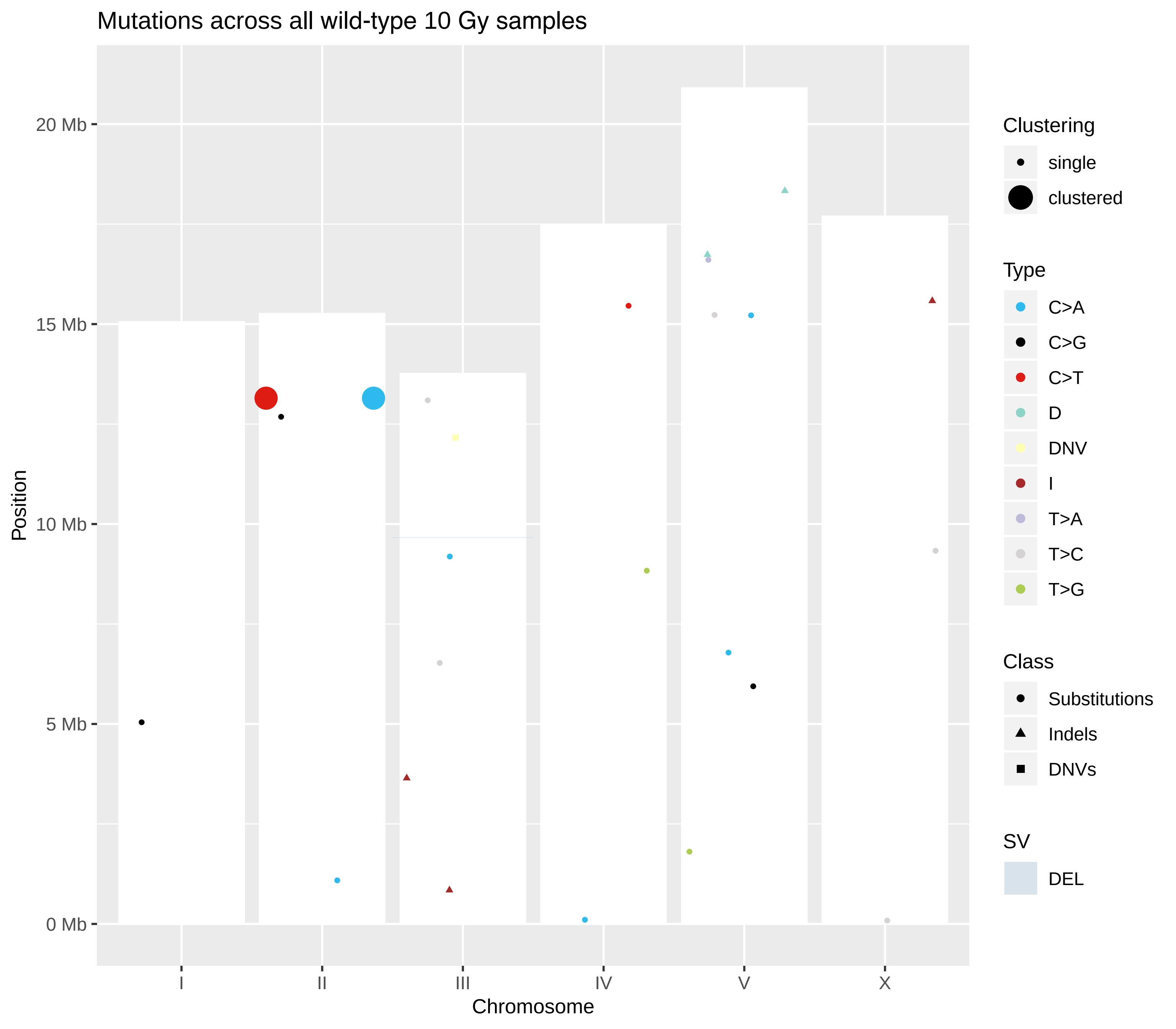
Mutations across all wild-type 10 Gy samples
Position
20 Mb
15 Mb
10 Mb
5 Mb
0 Mb
I
II
III
IV
V
X
Chromosome
Clustering
single
clustered
Type
C>A
C>G
C>T
D
DNV
I
T>A
T>C
T>G
Class
Substitutions
Indels
DNVs
SV
DEL

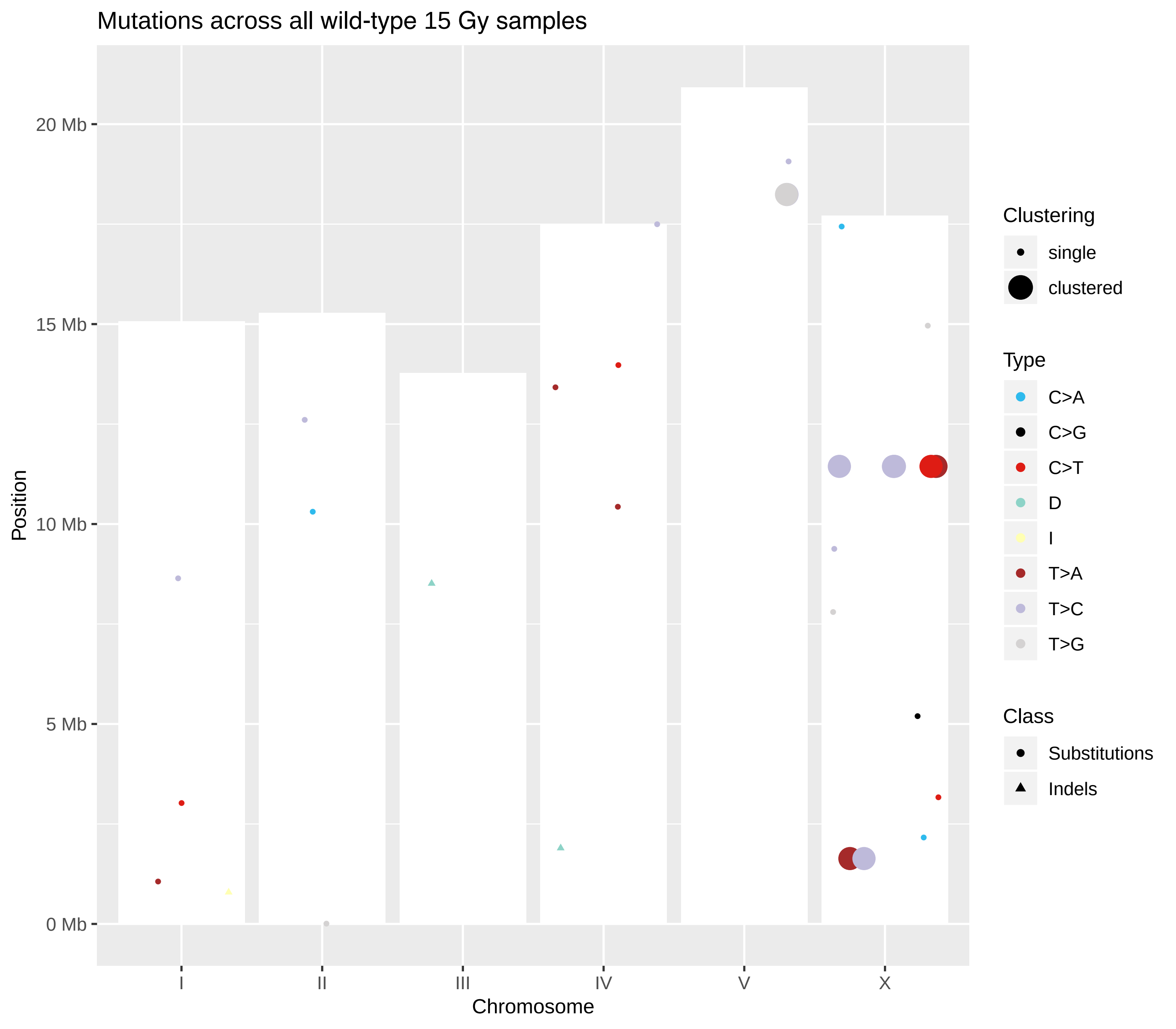

Mutations across all wild-type 15 Gy samples
20 Mb
15 Mb
10 Mb
5 Mb
0 Mb
Position
I
II
III
IV
V
X
Chromosome
Clustering
single
clustered
Type
C>A
C>G
C>T
D
I
T>A
T>C
T>G
Class
Substitutions
Indels

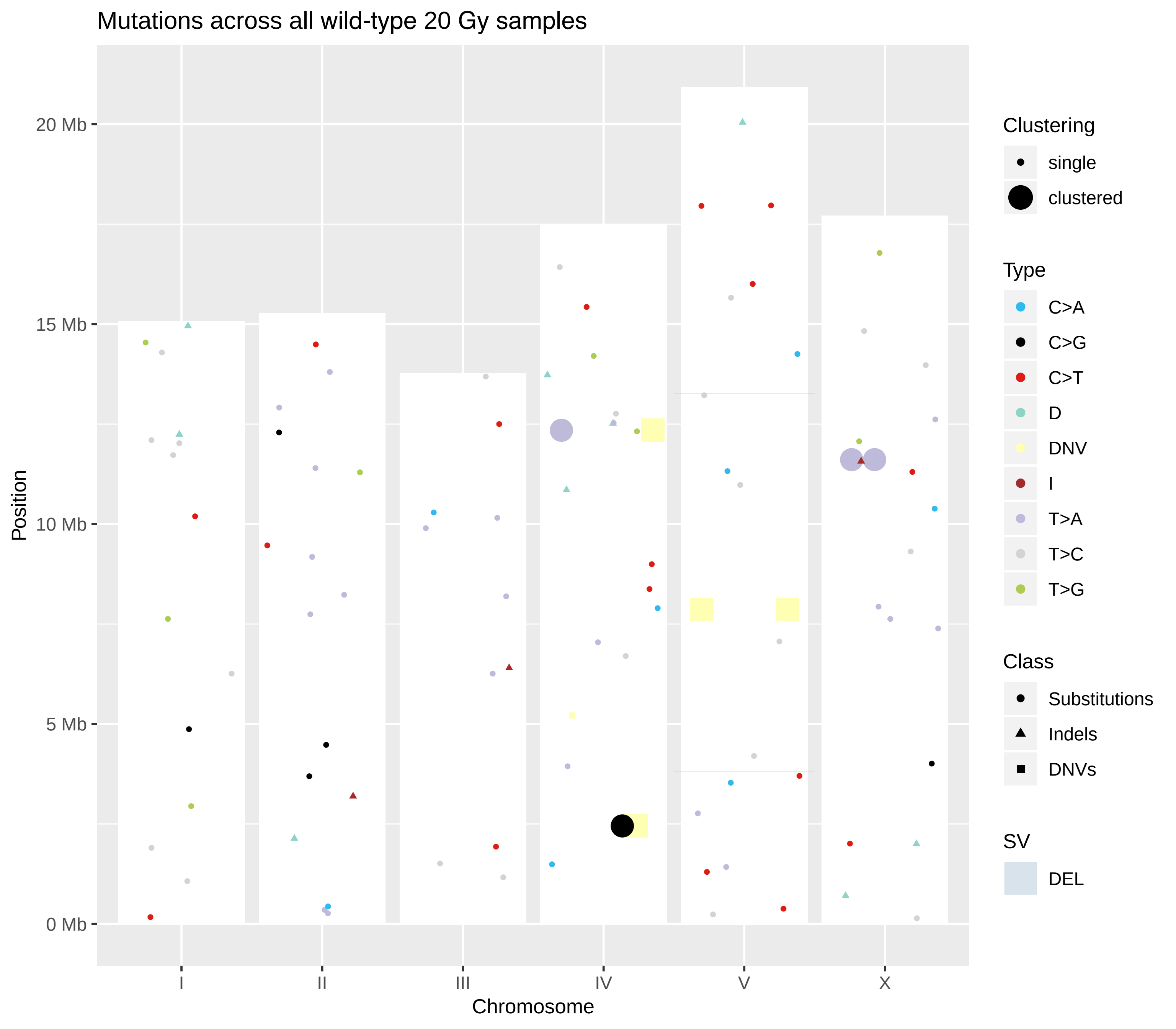
Mutations across all wild-type 20 Gy samples
Position
20 Mb
15 Mb
10 Mb
5 Mb
0 Mb
I
II
III
IV
V
X
Chromosome
Clustering
single
clustered
Type
C>A
C>G
C>T
D
DNV
I
T>A
T>C
T>G
Class
Substitutions
Indels
DNVs
SV
DEL

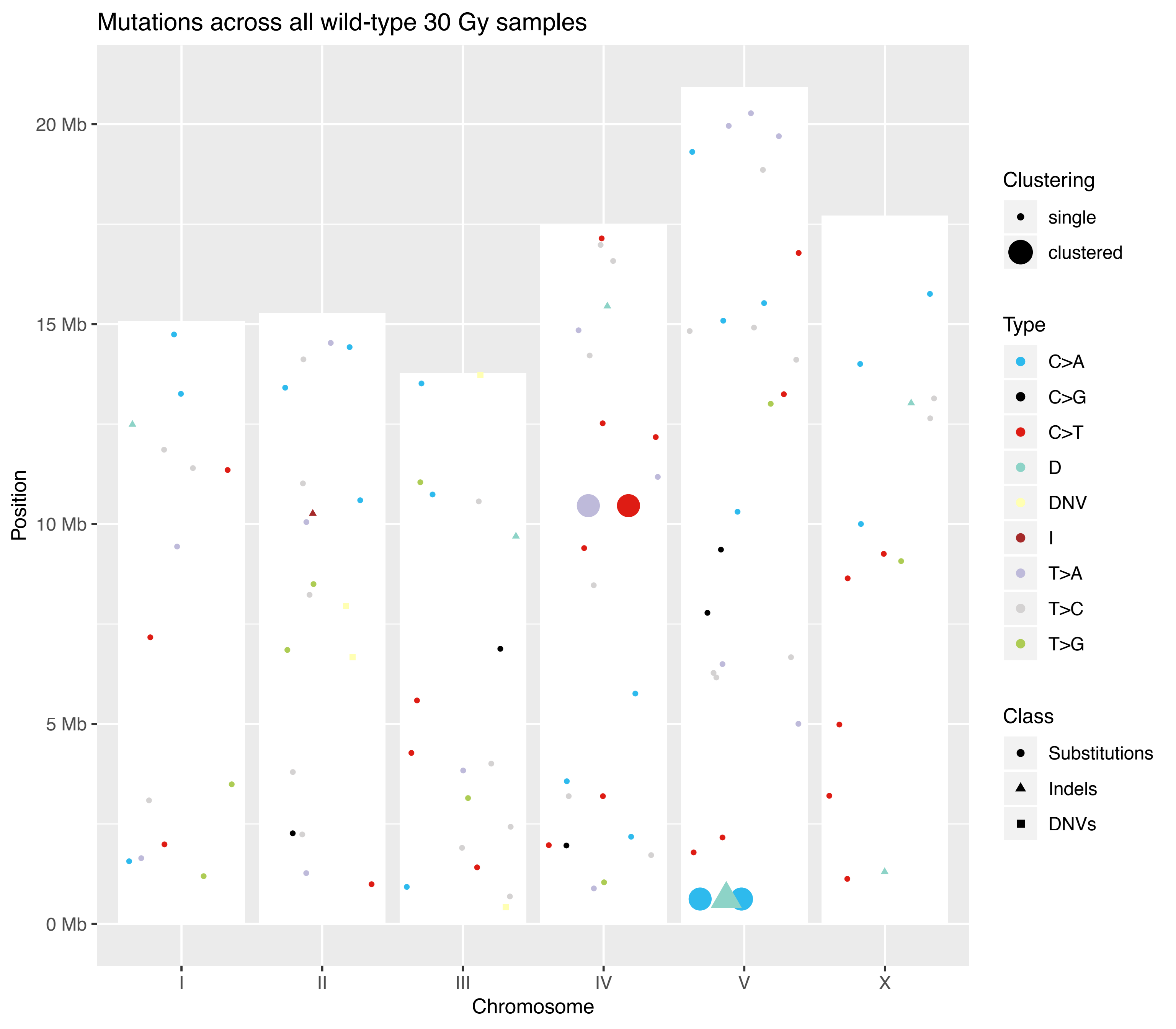

Mutations across all wild-type 30 Gy samples
Position
20 Mb
15 Mb
10 Mb
5 Mb
0 Mb
I
II
III
IV
V
X
Chromosome
Clustering
single
clustered
Type
C>A
C>G
C>T
D
DNV
I
T>A
T>C
T>G
Class
Substitutions
Indels
DNVs

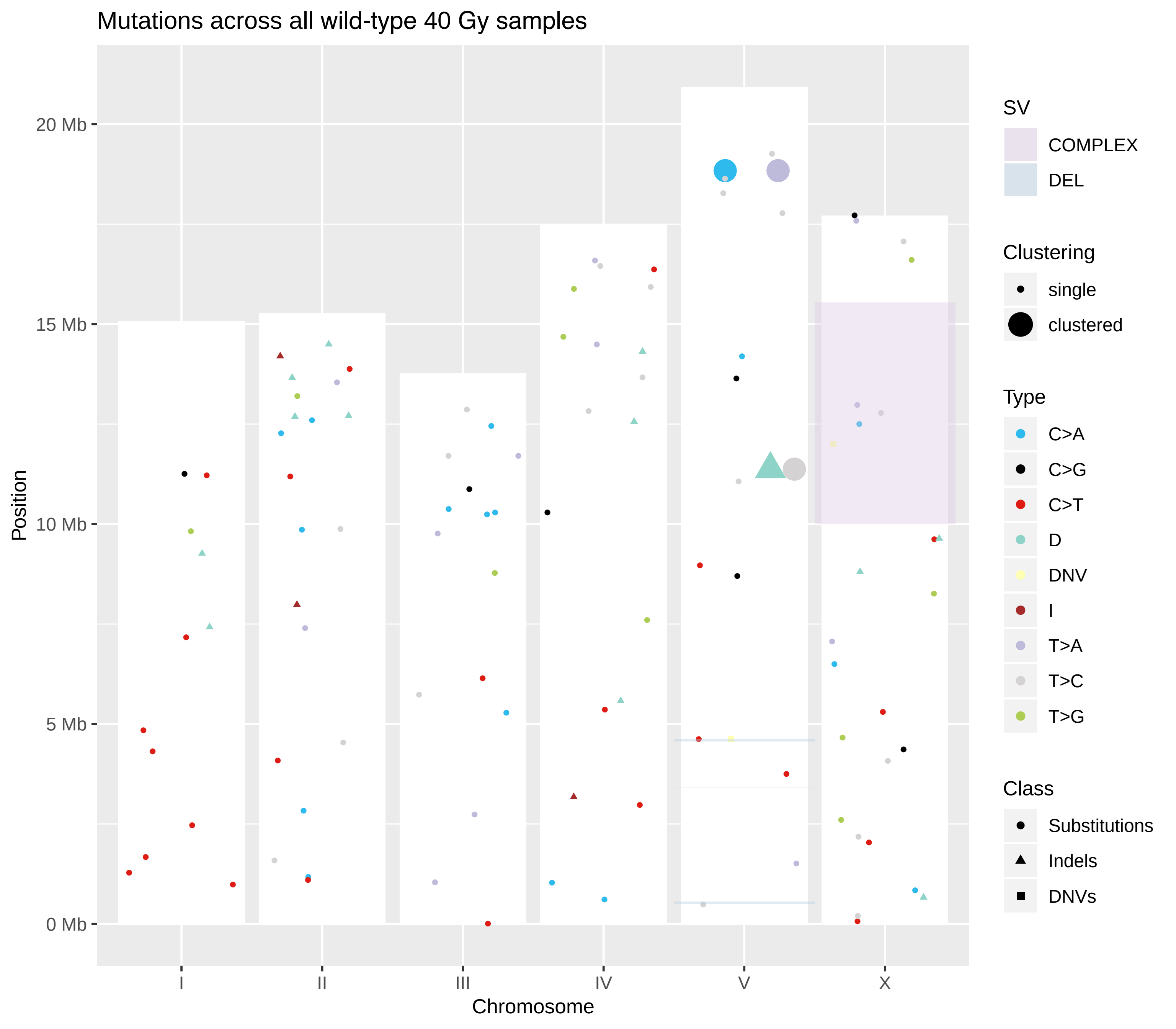

Mutations across all wild-type 40 Gy samples
Position
20 Mb
15 Mb
10 Mb
5 Mb
0 Mb
I
II
III
IV
V
X
Chromosome
SV
COMPLEX
DEL
Clustering
single
clustered
Type
C>A
C>G
C>T
D
DNV
I
T>A
T>C
T>G
Class
Substitutions
Indels
DNVs

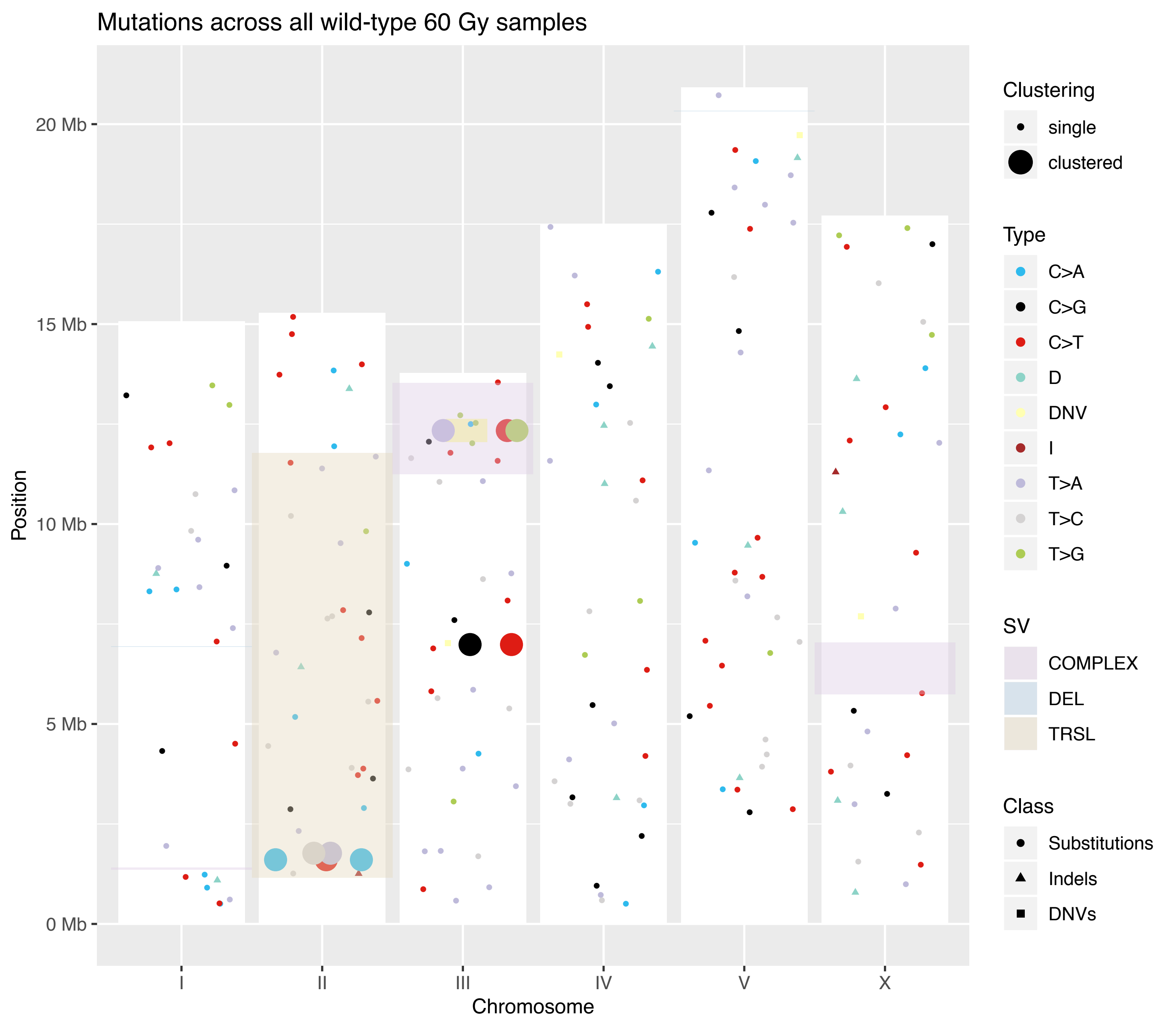
Mutations across all wild-type 60 Gy samples
Position
20 Mb
15 Mb
10 Mb
5 Mb
0 Mb
I
II
III
IV
V
X
Chromosome
Clustering
single
clustered
Type
C>A
C>G
C>T
D
DNV
I
T>A
T>C
T>G
SV
COMPLEX
DEL
TRSL
Class
Substitutions
Indels
DNVs

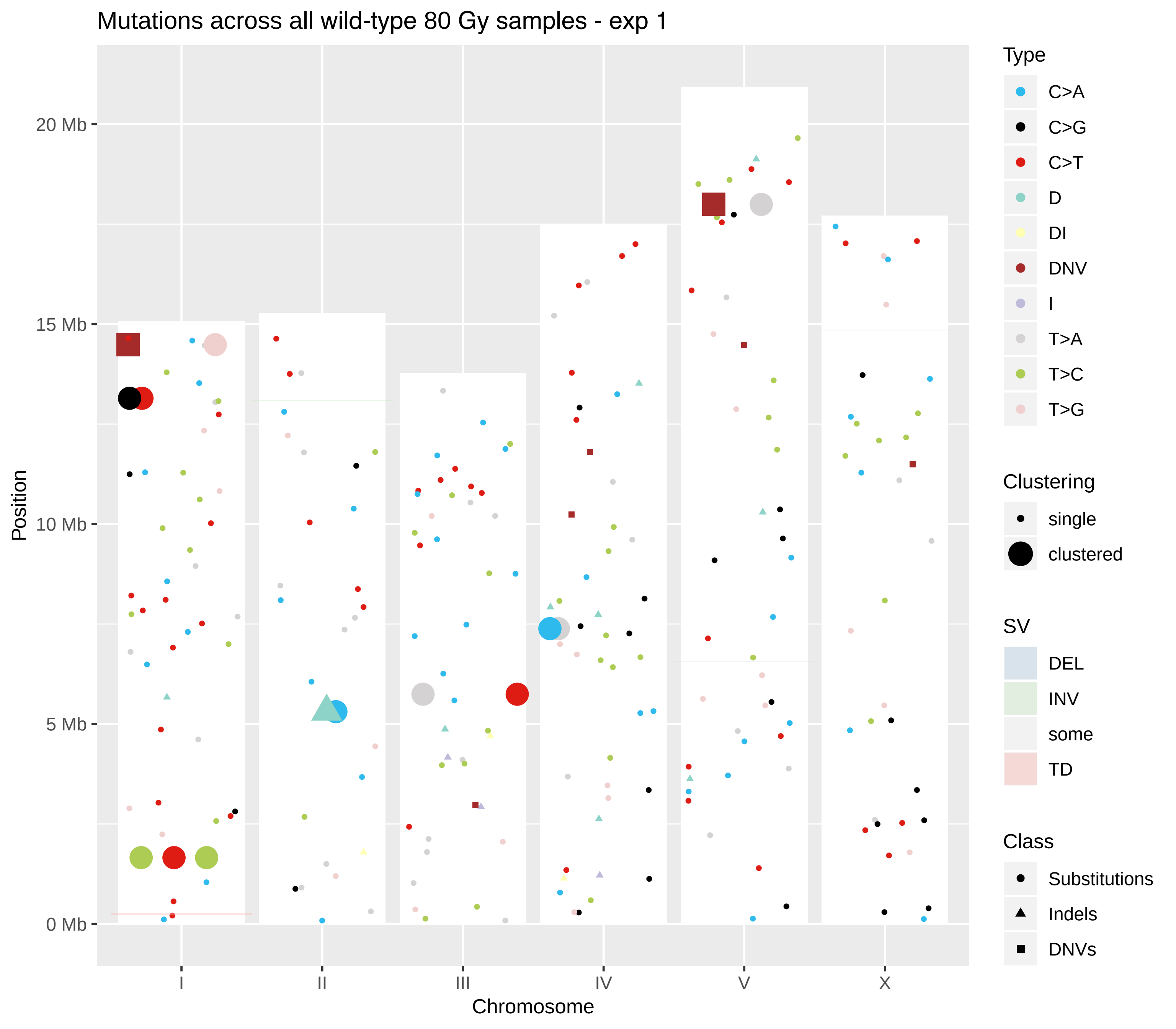

Mutations across all wild-type 80 Gy samples - exp 1
Position
20 Mb
15 Mb
10 Mb
5 Mb
0 Mb
I
II
III
IV
V
X
Chromosome
Type
C>A
C>G
C>T
D
DI
DNV
I
T>A
T>C
T>G
Clustering
single
clustered
SV
DEL
INV
some
TD
Class
Substitutions
Indels
DNVs

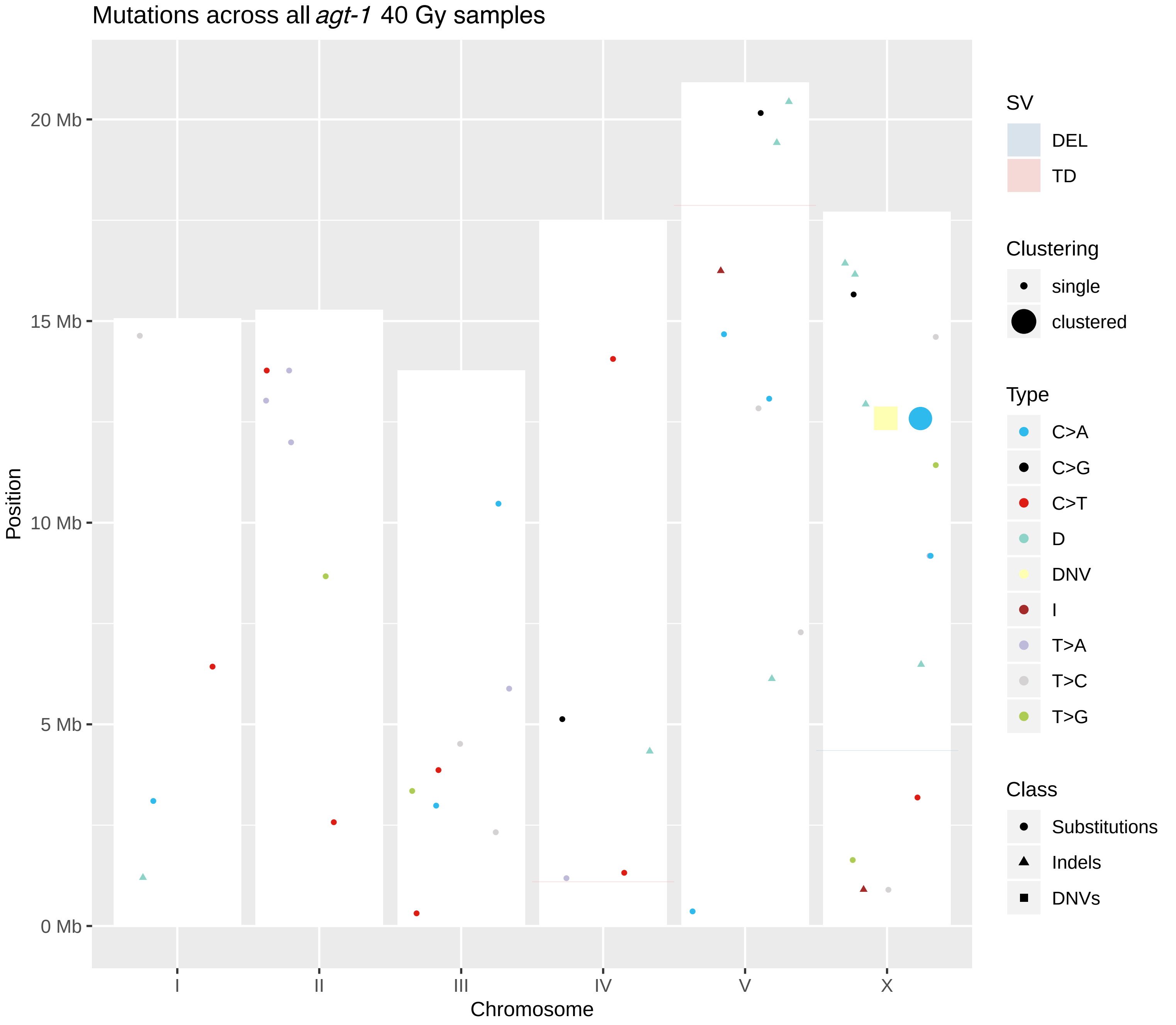
Mutations across all *agt-1* 40 Gy samples
Position
20 Mb
15 Mb
10 Mb
5 Mb
0 Mb
I
II
III
IV
V
X
Chromosome
SV
DEL
TD
Clustering
single
clustered
Type
C>A
C>G
C>T
D
DNV
I
T>A
T>C
T>G
Class
Substitutions
Indels
DNVs

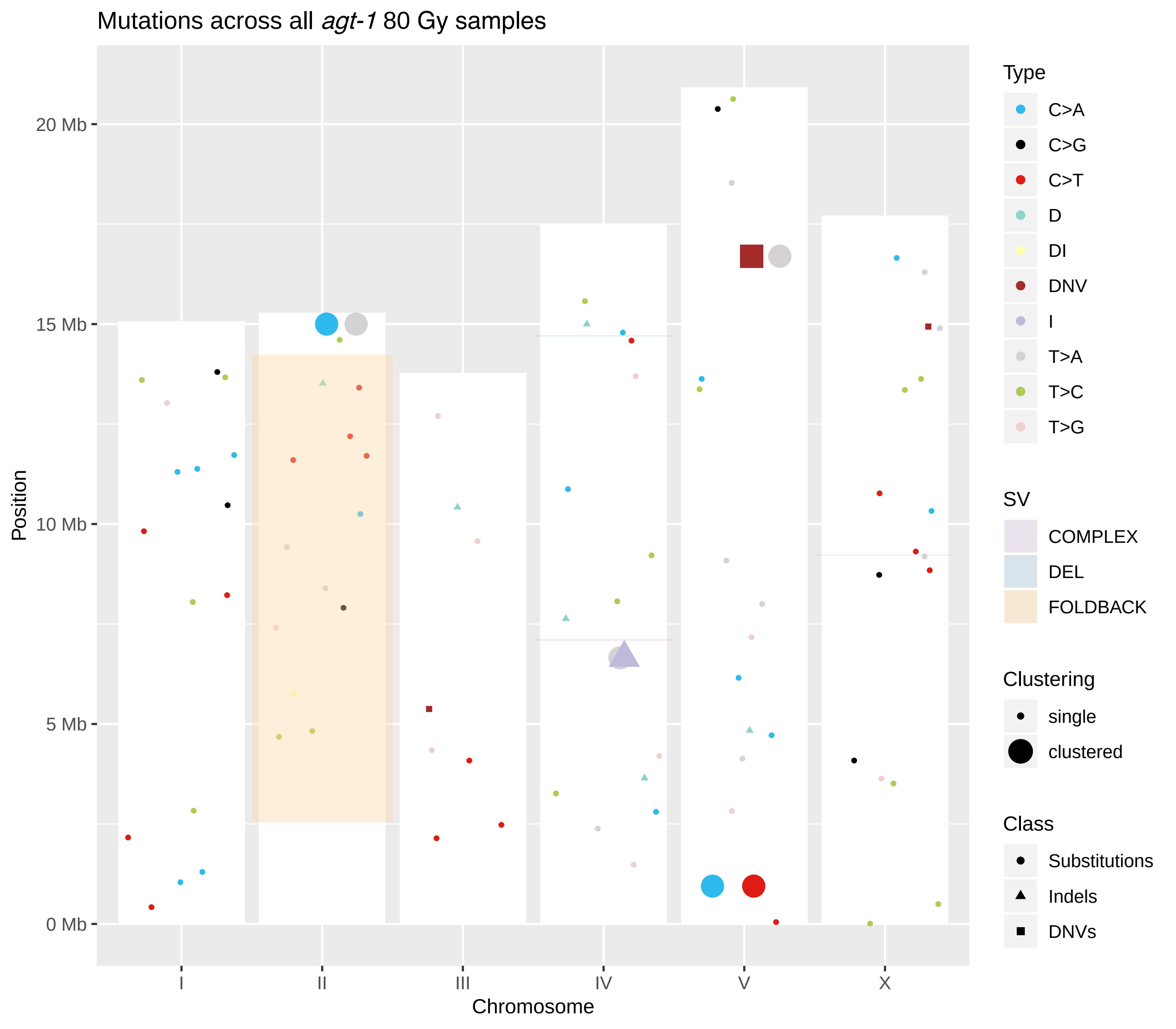
Mutations across all agt-1 80 Gy samples
Position
20 Mb
15 Mb
10 Mb
5 Mb
0 Mb
Chromosome
I
II
III
IV
V
X
Type
C>A
C>G
C>T
D
DI
DNV
I
T>A
T>C
T>G
SV
COMPLEX
DEL
FOLDBACK
Clustering
single
clustered
Class
Substitutions
Indels
DNVs

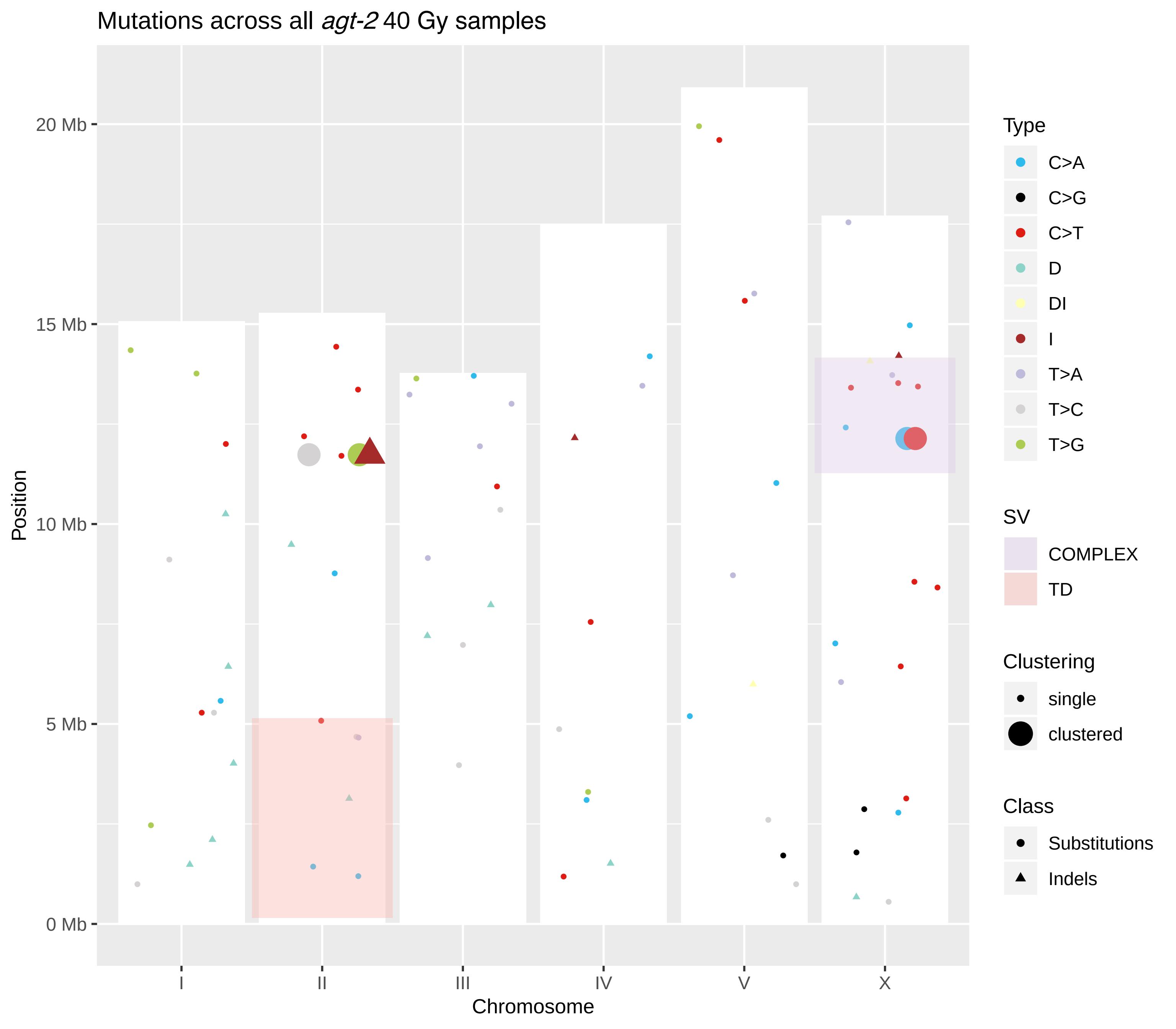
Mutations across all *agt-2* 40 Gy samples
Position
20 Mb
15 Mb
10 Mb
5 Mb
0 Mb
I
II
III
IV
V
X
Chromosome
Type
C>A
C>G
C>T
D
DI
I
T>A
T>C
T>G
SV
COMPLEX
TD
Clustering
single
clustered
Class
Substitutions
Indels

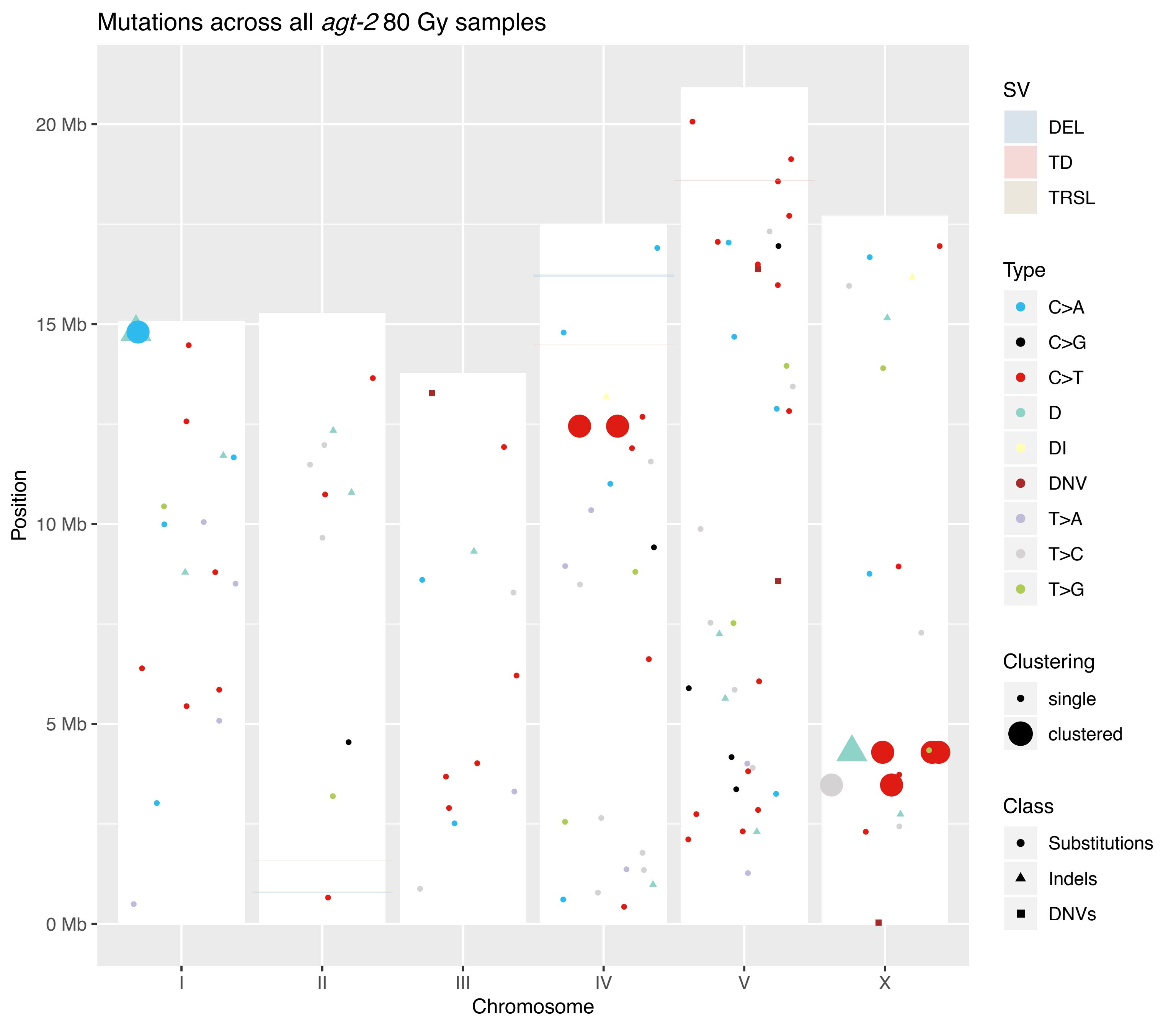

Mutations across all *agt-2* 80 Gy samples
Position
20 Mb
15 Mb
10 Mb
5 Mb
0 Mb
I
II
III
IV
V
X
Chromosome
SV
DEL
TD
TRSL
Type
C>A
C>G
C>T
D
DI
DNV
T>A
T>C
T>G
Clustering
single
clustered
Class
Substitutions
Indels
DNVs

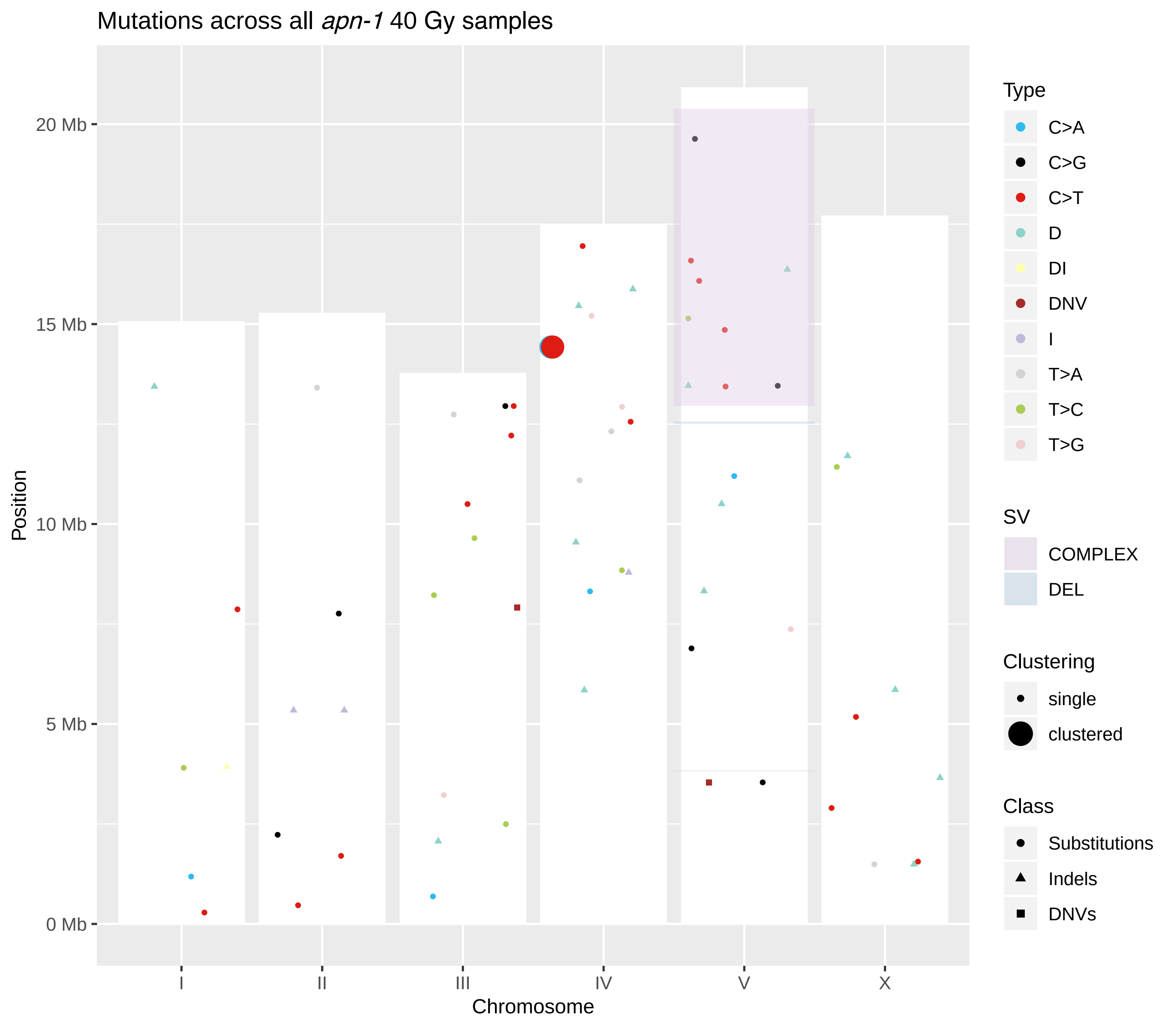

Mutations across all *apn-1* 40 Gy samples
Position
20 Mb
15 Mb
10 Mb
5 Mb
0 Mb
I
II
III
IV
V
X
Chromosome
Type
C>A
C>G
C>T
D
DI
DNV
I
T>A
T>C
T>G
SV
COMPLEX
DEL
Clustering
single
clustered
Class
Substitutions
Indels
DNVs

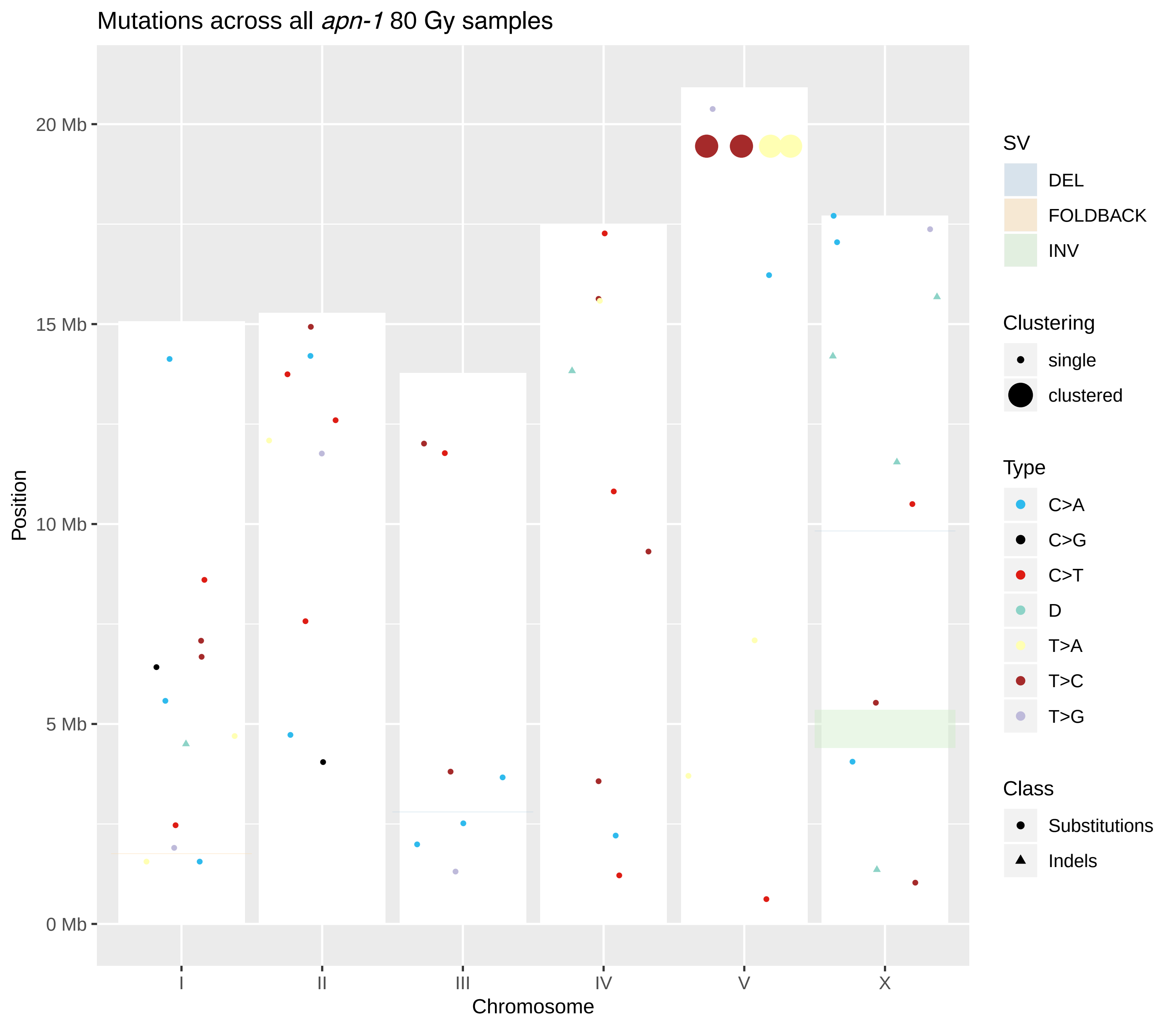

Mutations across all *apn-1* 80 Gy samples
Position
20 Mb
15 Mb
10 Mb
5 Mb
0 Mb
I
II
III
IV
V
X
Chromosome
SV
DEL
FOLDBACK
INV
Clustering
single
clustered
Type
C>A
C>G
C>T
D
T>A
T>C
T>G
Class
Substitutions
Indels

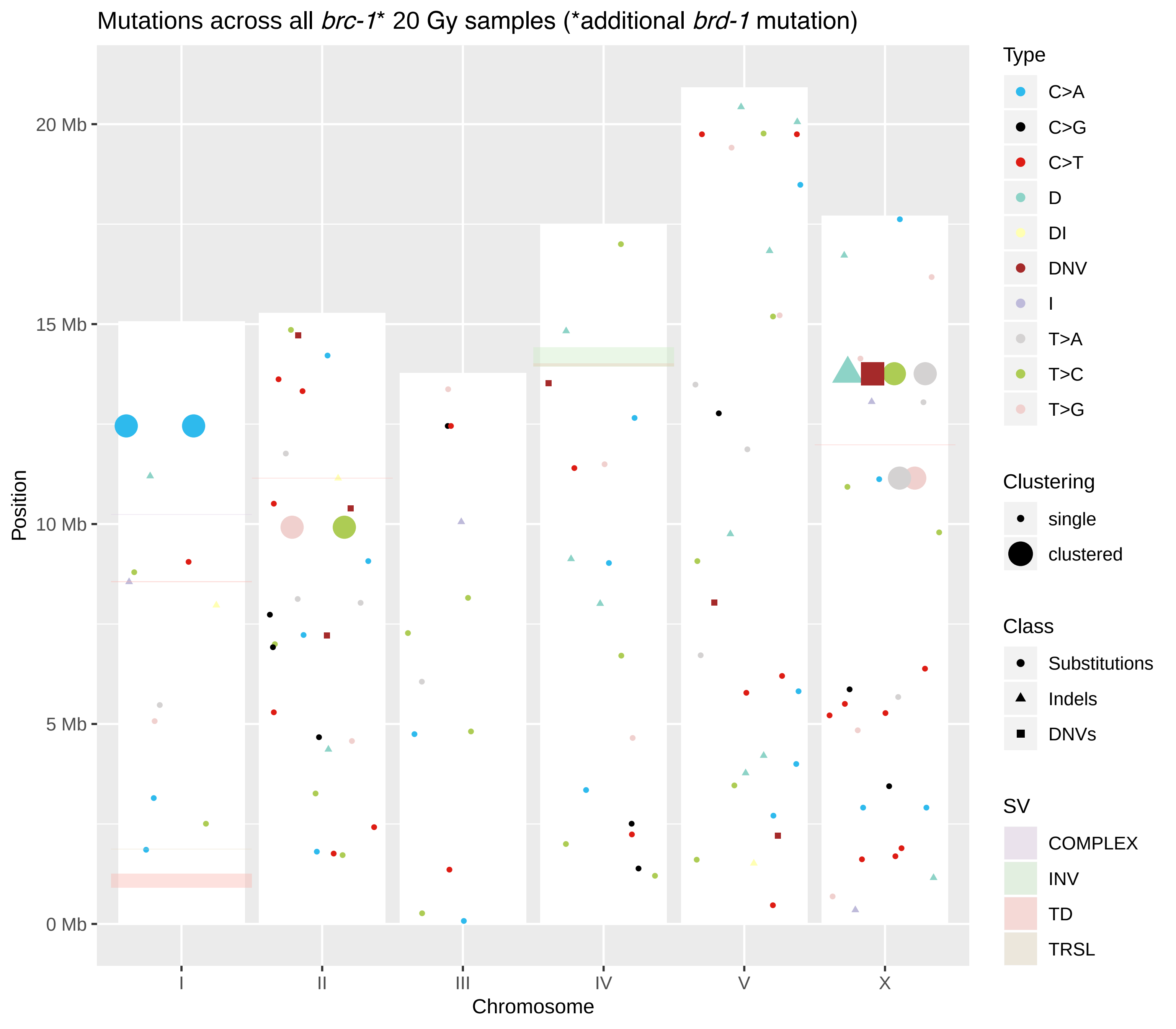

Mutations across all brc-1* 20 Gy samples (*additional brd-1 mutation)
Position
20 Mb
15 Mb
10 Mb
5 Mb
0 Mb
Chromosome
I
II
III
IV
V
X
Type
C>A
C>G
C>T
D
DI
DNV
I
T>A
T>C
T>G
Clustering
single
clustered
Class
Substitutions
Indels
DNVs
SV
COMPLEX
INV
TD
TRSL

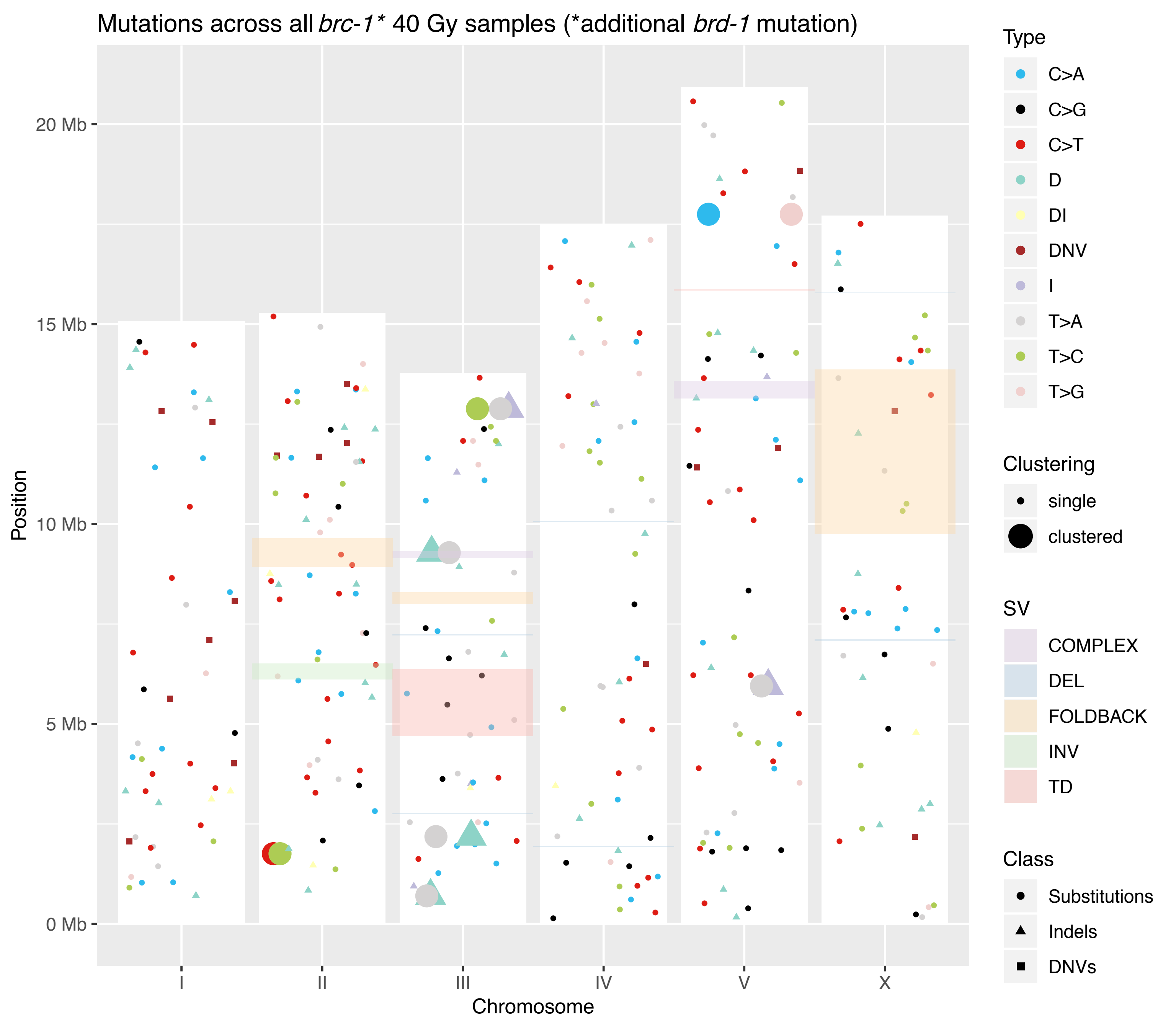
Mutations across all brc-1* 40 Gy samples (*additional brd-1 mutation)
Position
20 Mb
15 Mb
10 Mb
5 Mb
0 Mb
I
II
III
IV
V
X
Chromosome
Type
C>A
C>G
C>T
D
DI
DNV
I
T>A
T>C
T>G
Clustering
single
clustered
SV
COMPLEX
DEL
FOLDBACK
INV
TD
Class
Substitutions
Indels
DNVs

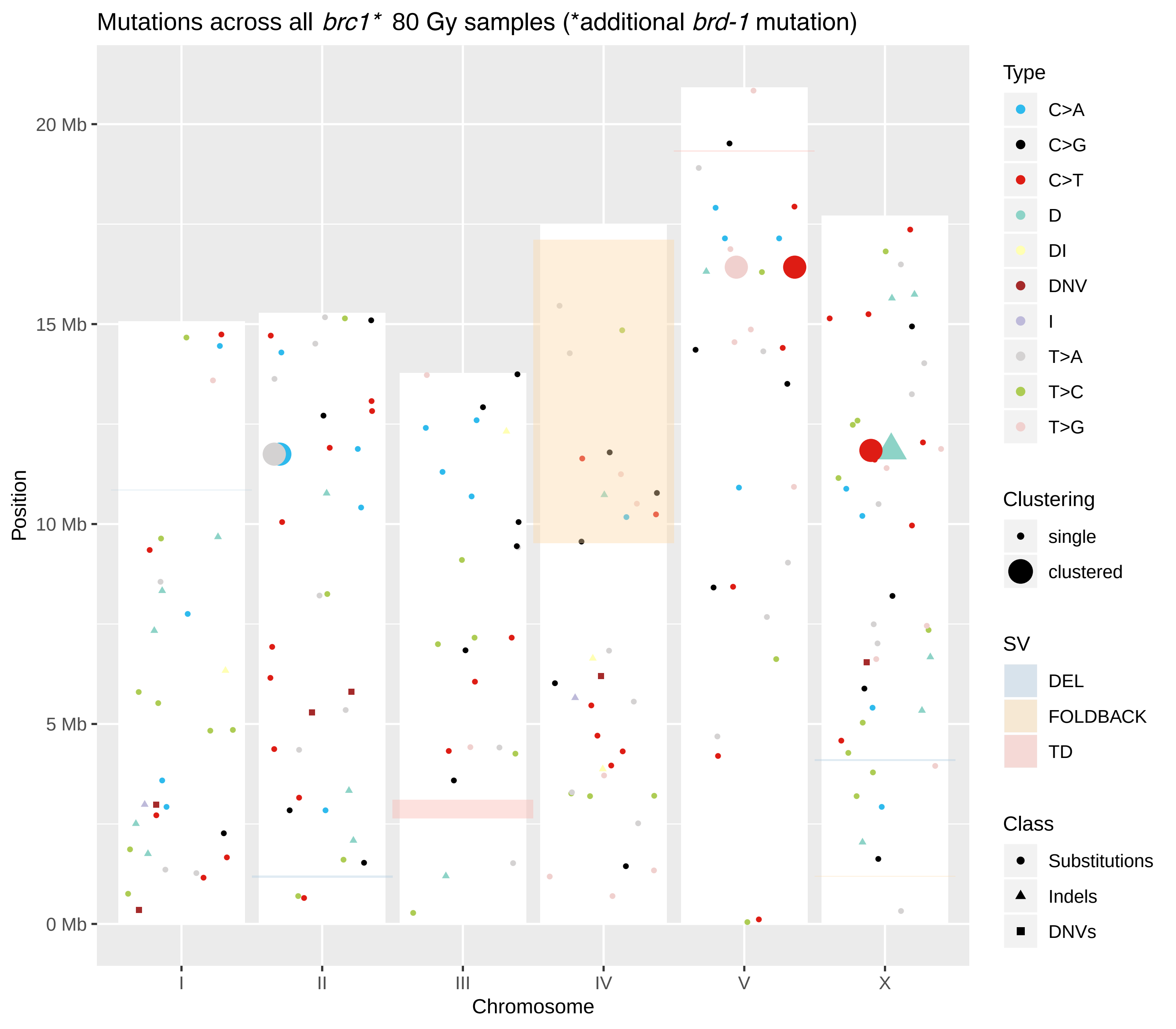
Mutations across all brc1* 80 Gy samples (*additional brd-1 mutation)
Position
20 Mb
15 Mb
10 Mb
5 Mb
0 Mb
Chromosome
I
II
III
IV
V
X
Type
C>A
C>G
C>T
D
DI
DNV
I
T>A
T>C
T>G
Clustering
single
clustered
SV
DEL
FOLDBACK
TD
Class
Substitutions
Indels
DNVs

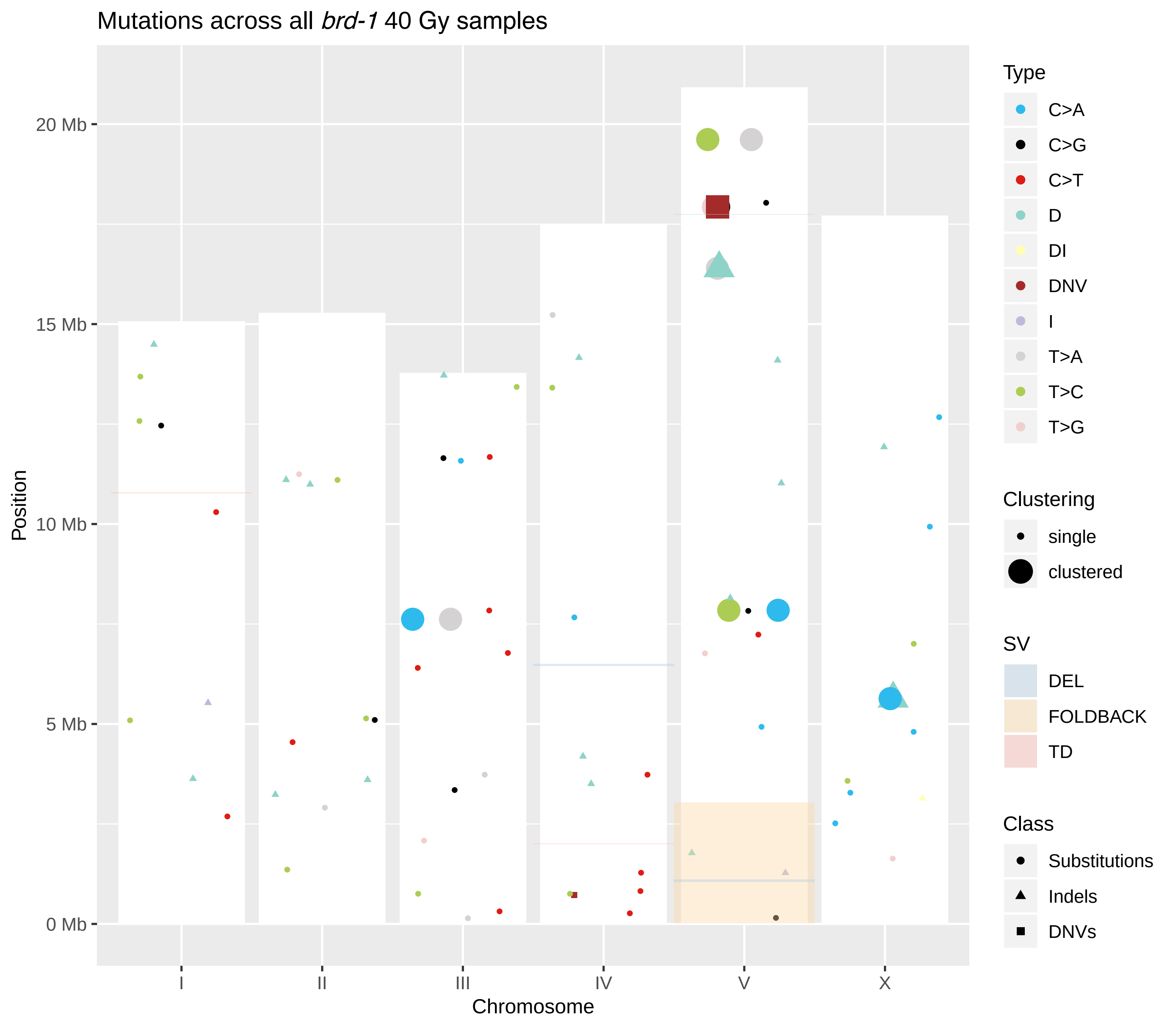
Mutations across all brd-1 40 Gy samples
Position
20 Mb
15 Mb
10 Mb
5 Mb
0 Mb
I
II
III
IV
V
X
Chromosome
Type
C>A
C>G
C>T
D
DI
DNV
I
T>A
T>C
T>G
Clustering
single
clustered
SV
DEL
FOLDBACK
TD
Class
Substitutions
Indels
DNVs

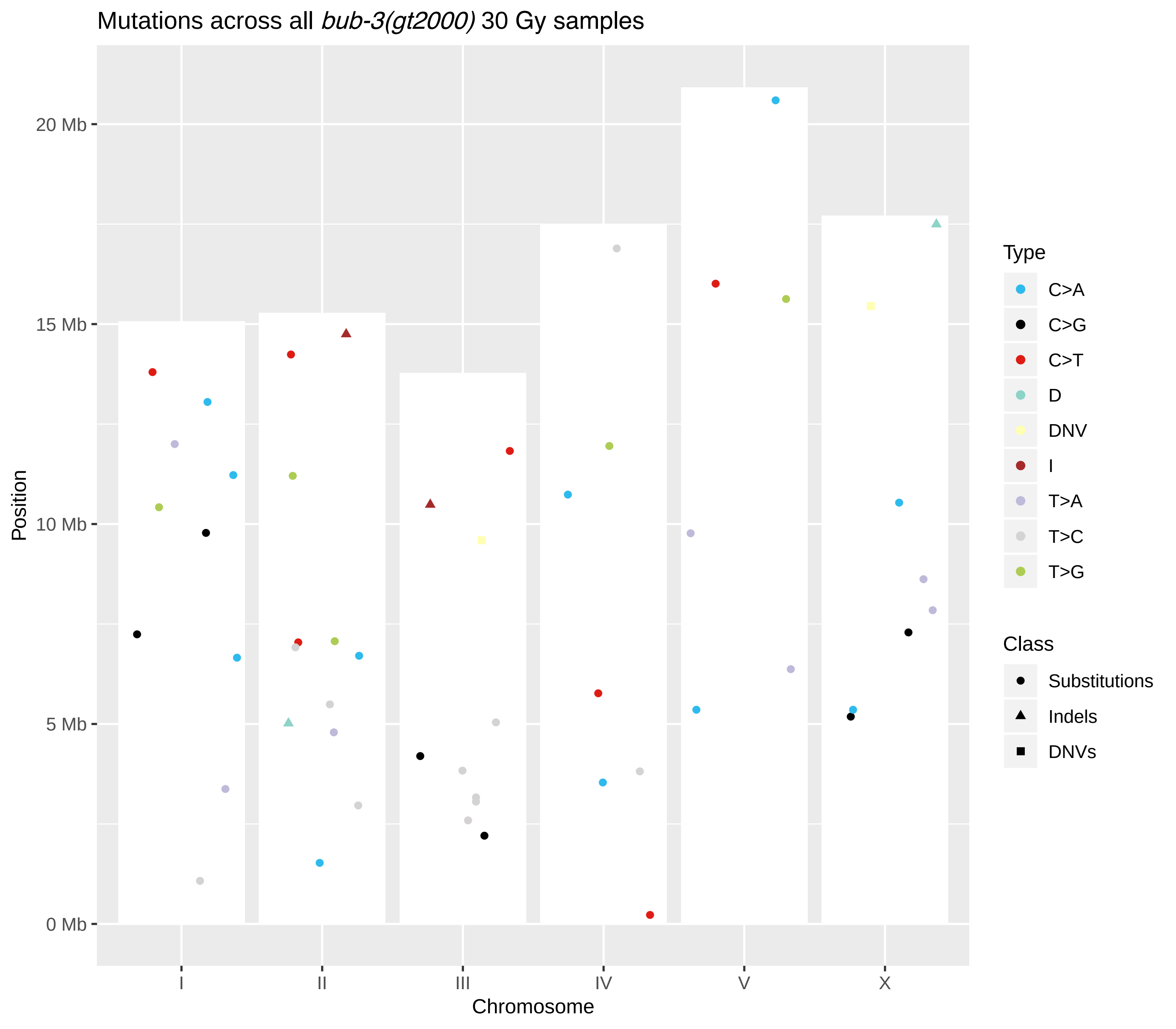
Mutations across all bub-3(gt2000) 30 Gy samples
Position
20 Mb
15 Mb
10 Mb
5 Mb
0 Mb
I
II
III
IV
V
X
Chromosome
Type
C>A
C>G
C>T
D
DNV
I
T>A
T>C
T>G
Class
Substitutions
Indels
DNVs

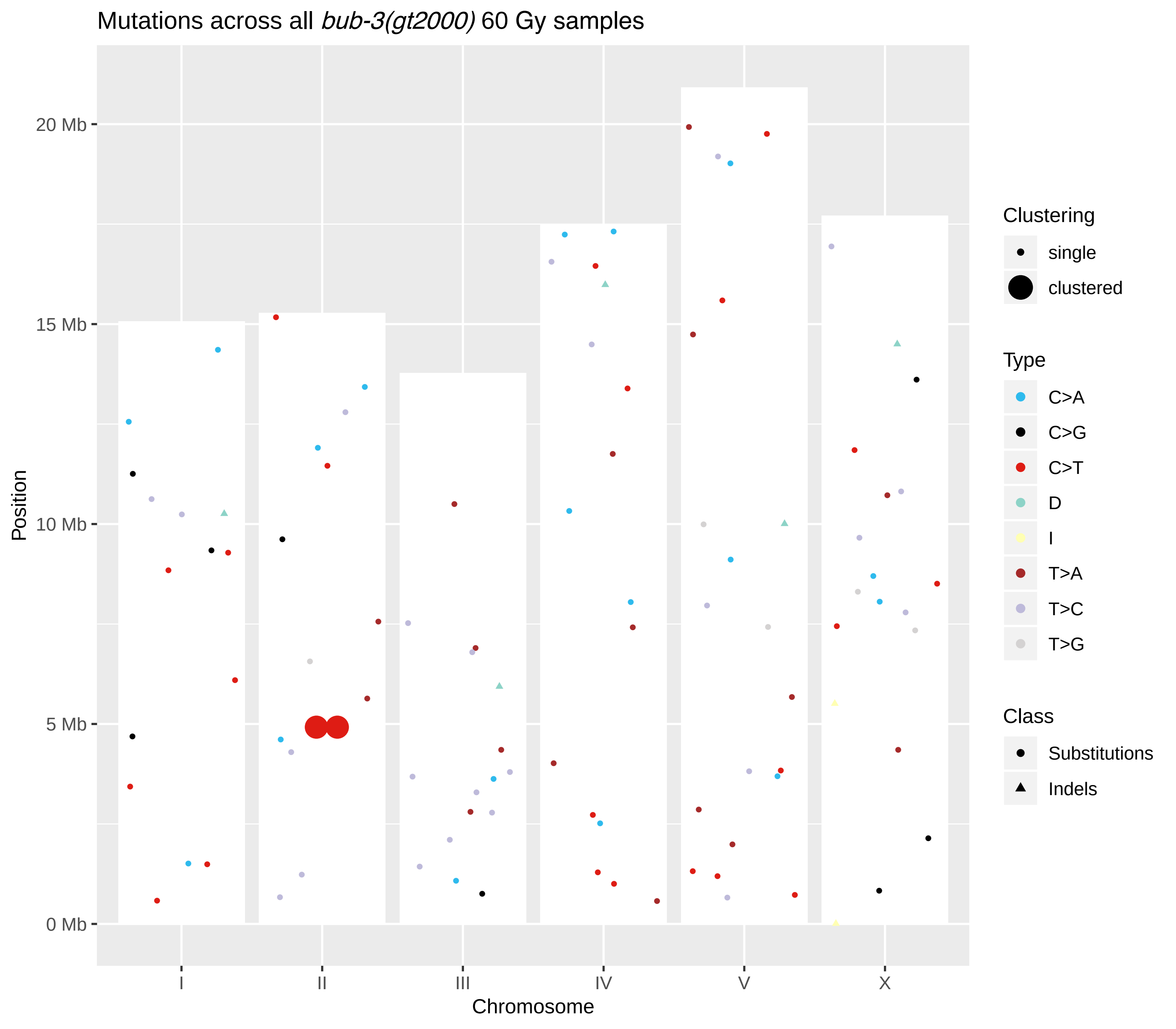
Mutations across all *bub-3(gt2000)* 60 Gy samples
Position
20 Mb
15 Mb
10 Mb
5 Mb
0 Mb
I
II
III
IV
V
X
Chromosome
Clustering
single
clustered
Type
C>A
C>G
C>T
D
I
T>A
T>C
T>G
Class
Substitutions
Indels

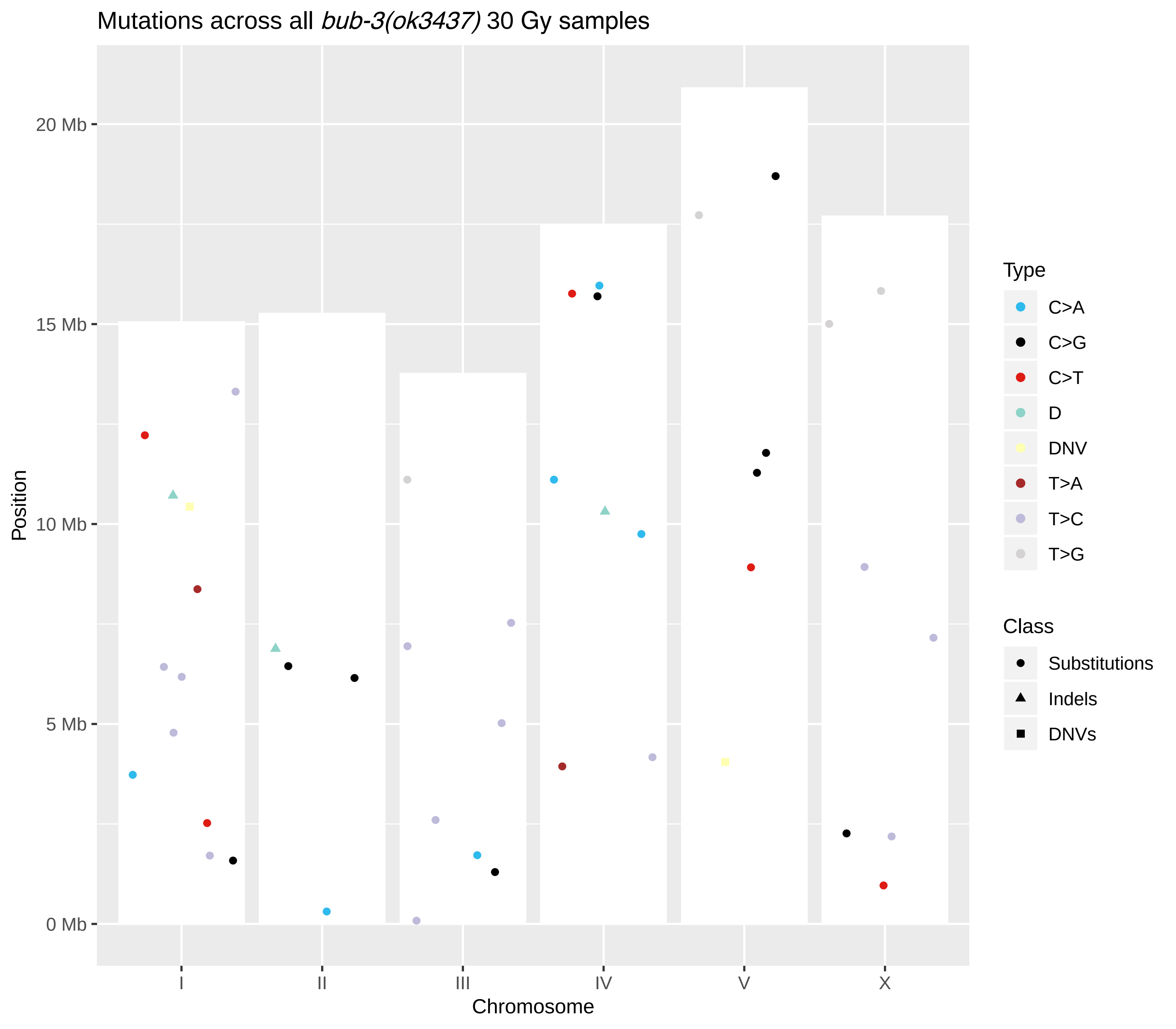
Mutations across all bub-3(ok3437) 30 Gy samples
Position
20 Mb
15 Mb
10 Mb
5 Mb
0 Mb
I
II
III
IV
V
X
Chromosome
Type
C>A
C>G
C>T
D
DNV
T>A
T>C
T>G
Class
Substitutions
Indels
DNVs

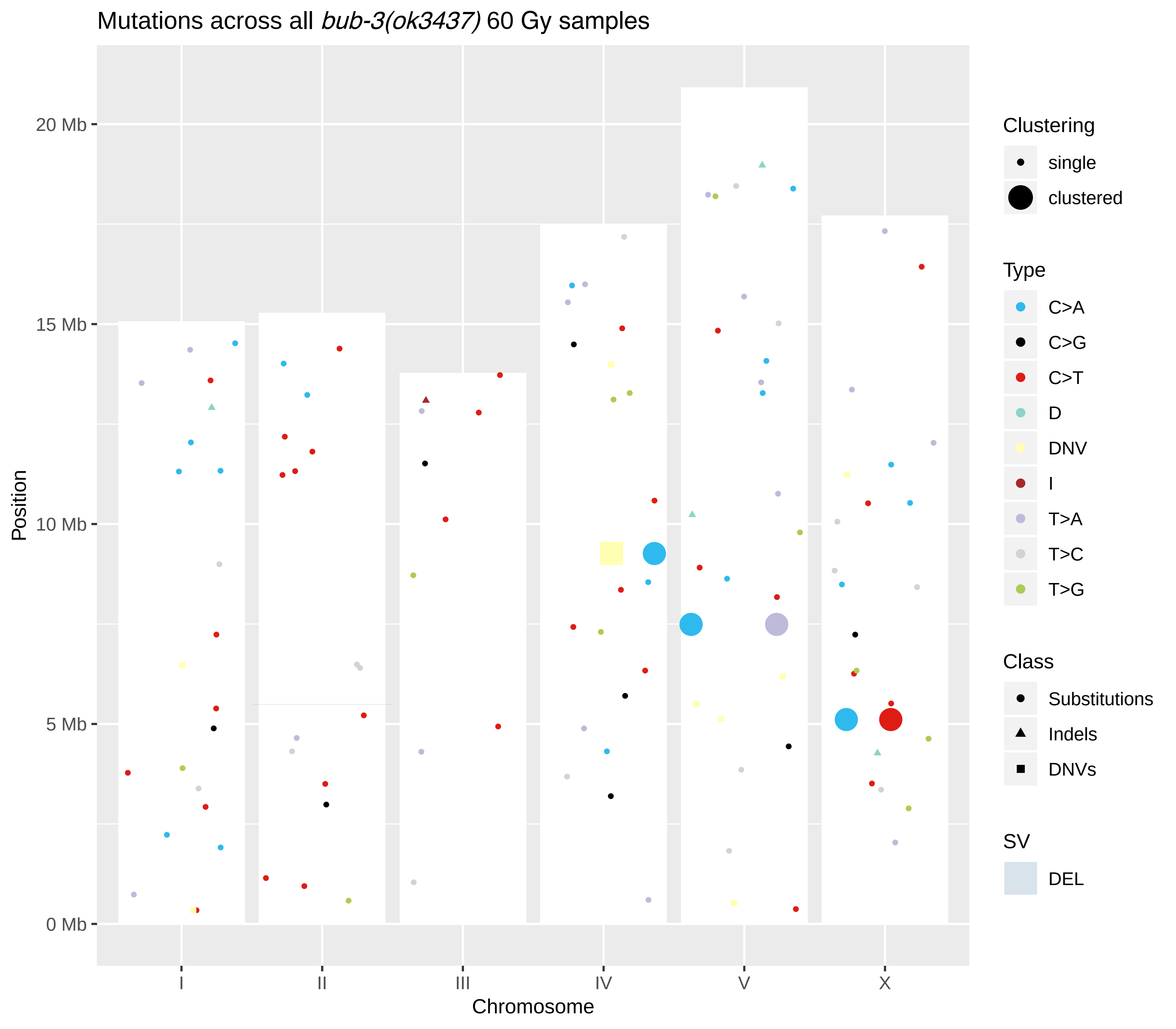
Mutations across all bub-3(ok3437) 60 Gy samples
Position
20 Mb
15 Mb
10 Mb
5 Mb
0 Mb
I
II
III
IV
V
X
Chromosome
Clustering
single
clustered
Type
C>A
C>G
C>T
D
DNV
I
T>A
T>C
T>G
Class
Substitutions
Indels
DNVs
SV
DEL

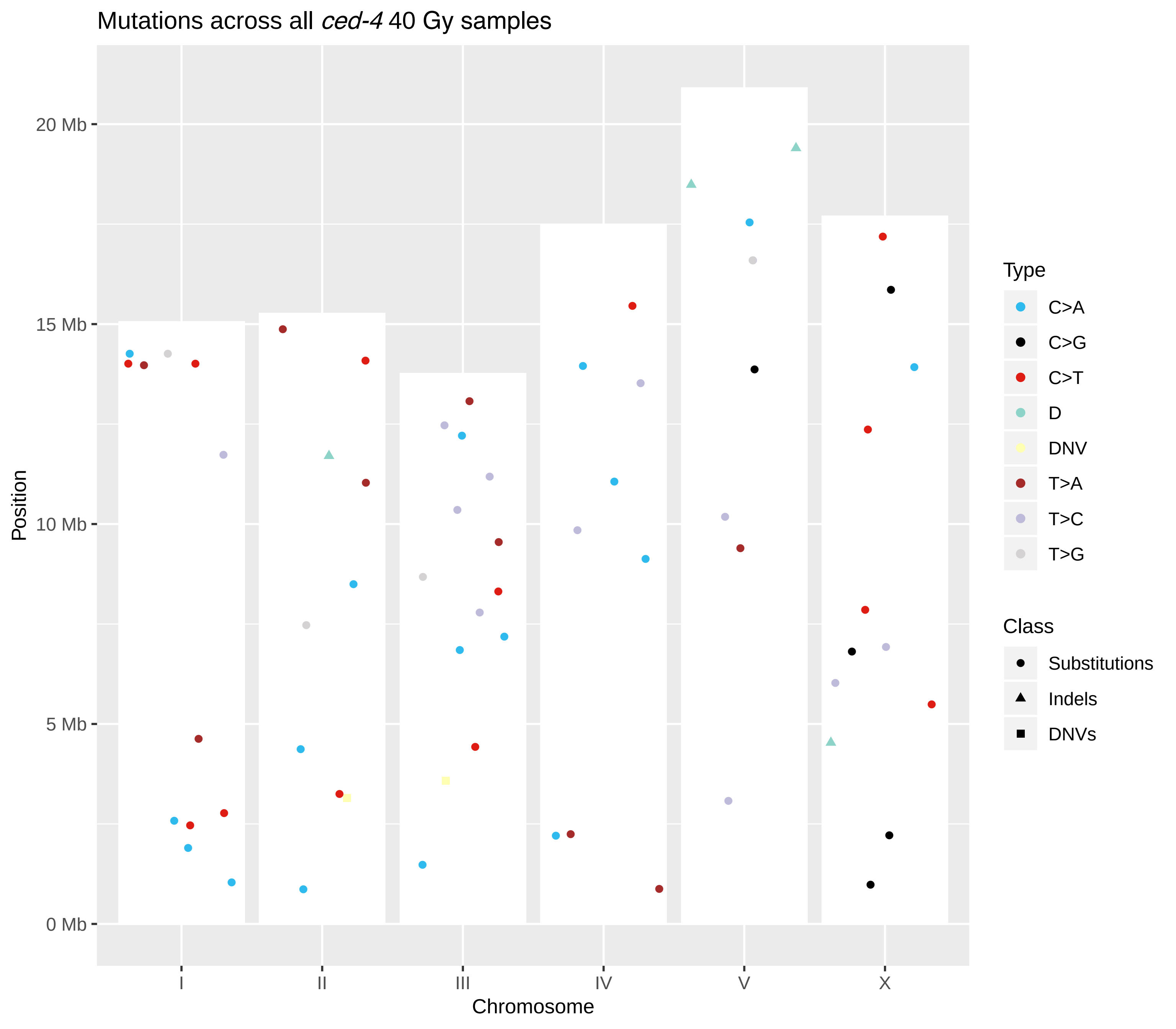

Mutations across all ced-4 40 Gy samples
20 Mb
15 Mb
10 Mb
5 Mb
0 Mb
Position
I
II
III
IV
V
X
Chromosome
Type
C>A
C>G
C>T
D
DNV
T>A
T>C
T>G
Class
Substitutions
Indels
DNVs

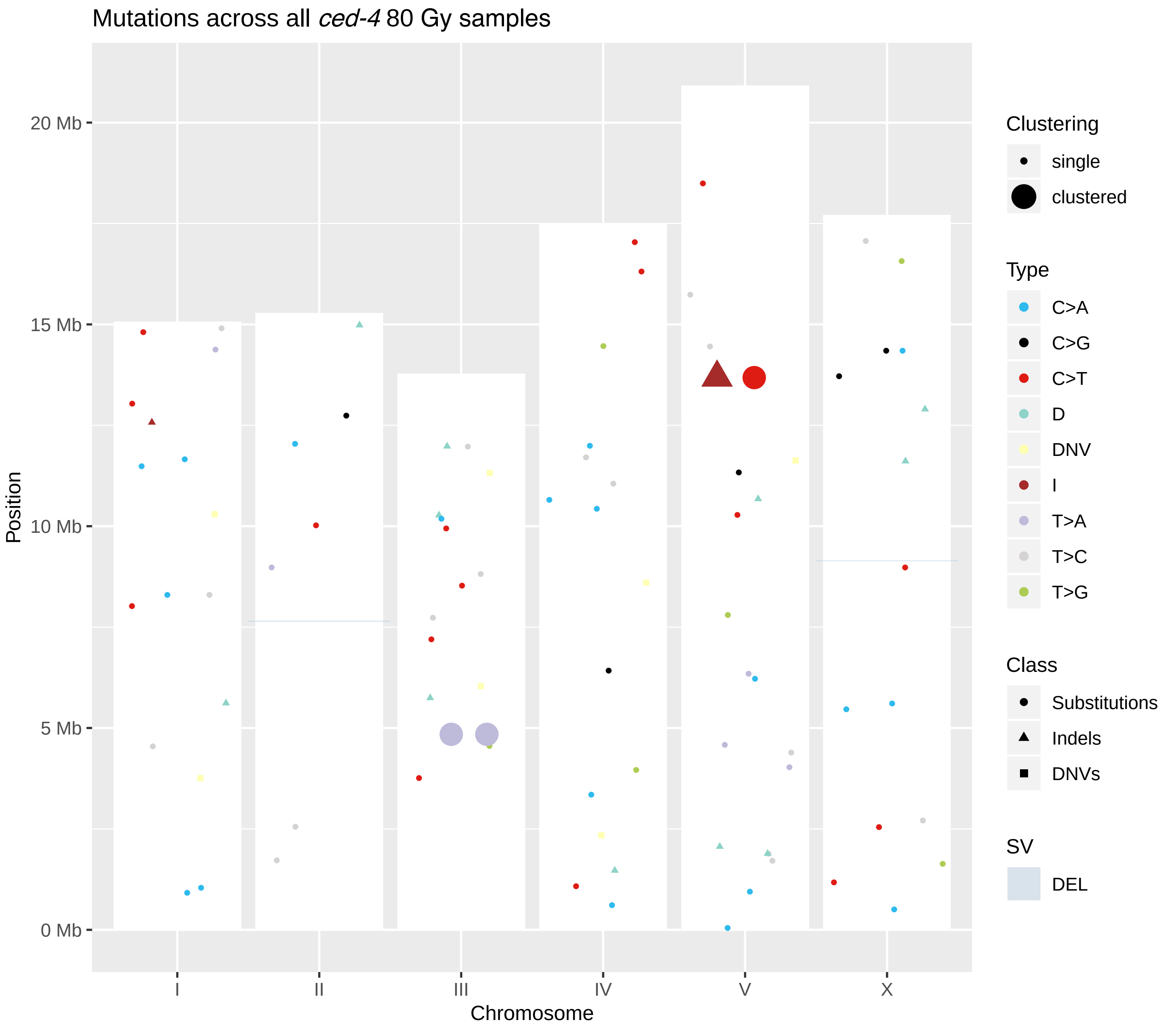
Mutations across all ced-4 80 Gy samples
Position
20 Mb
15 Mb
10 Mb
5 Mb
0 Mb
I
II
III
IV
V
X
Chromosome
Clustering
single
clustered
Type
C>A
C>G
C>T
D
DNV
I
T>A
T>C
T>G
Class
Substitutions
Indels
DNVs
SV
DEL

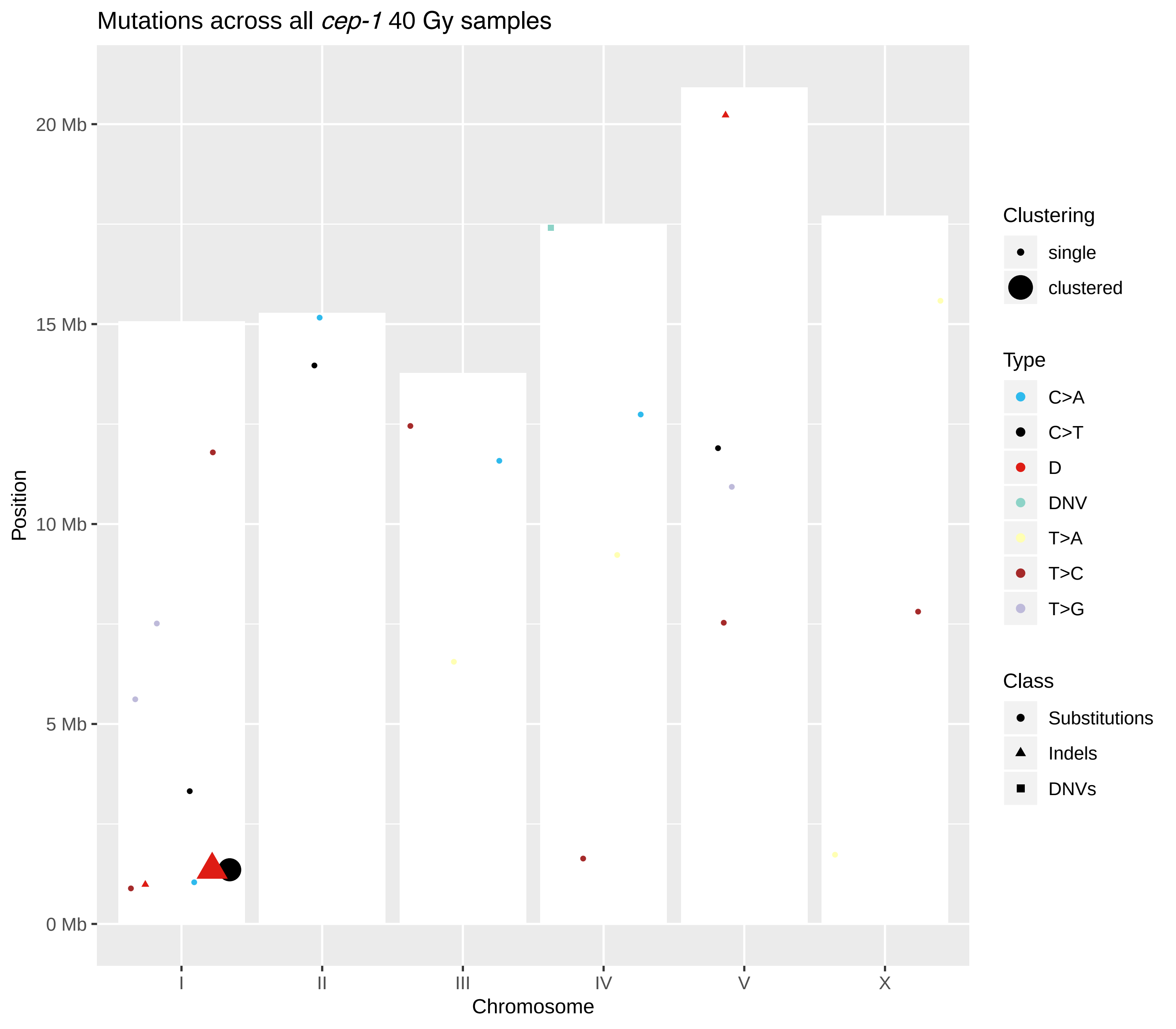

Mutations across all *cep-1* 40 Gy samples
20 Mb
15 Mb
10 Mb
5 Mb
0 Mb
Position
I
II
III
IV
V
X
Chromosome
Clustering
single
clustered
Type
C>A
C>T
D
DNV
T>A
T>C
T>G
Class
Substitutions
Indels
DNVs

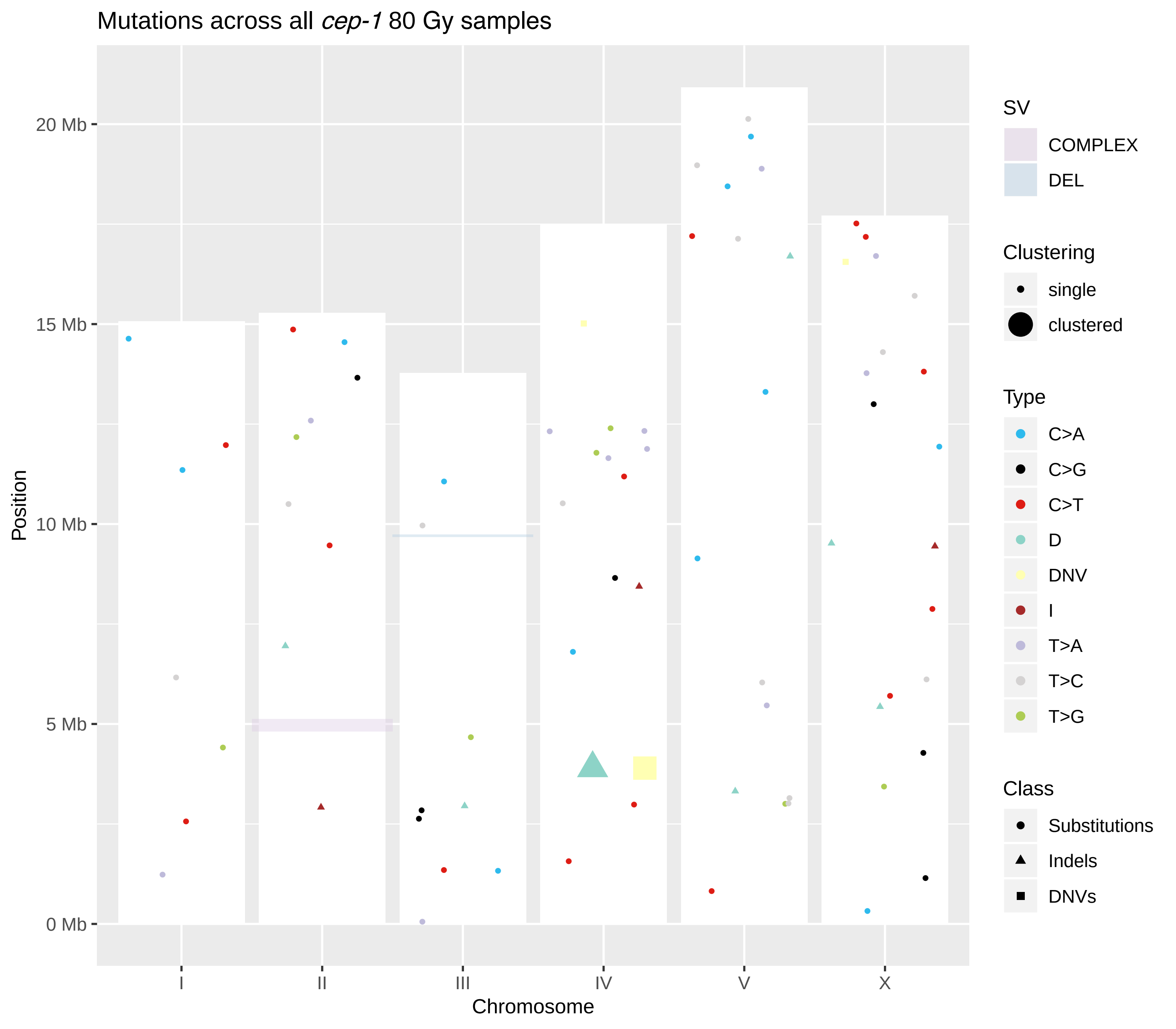

Mutations across all cep-1 80 Gy samples
Position
20 Mb
15 Mb
10 Mb
5 Mb
0 Mb
I
II
III
IV
V
X
Chromosome
SV
COMPLEX
DEL
Clustering
single
clustered
Type
C>A
C>G
C>T
D
DNV
I
T>A
T>C
T>G
Class
Substitutions
Indels
DNVs

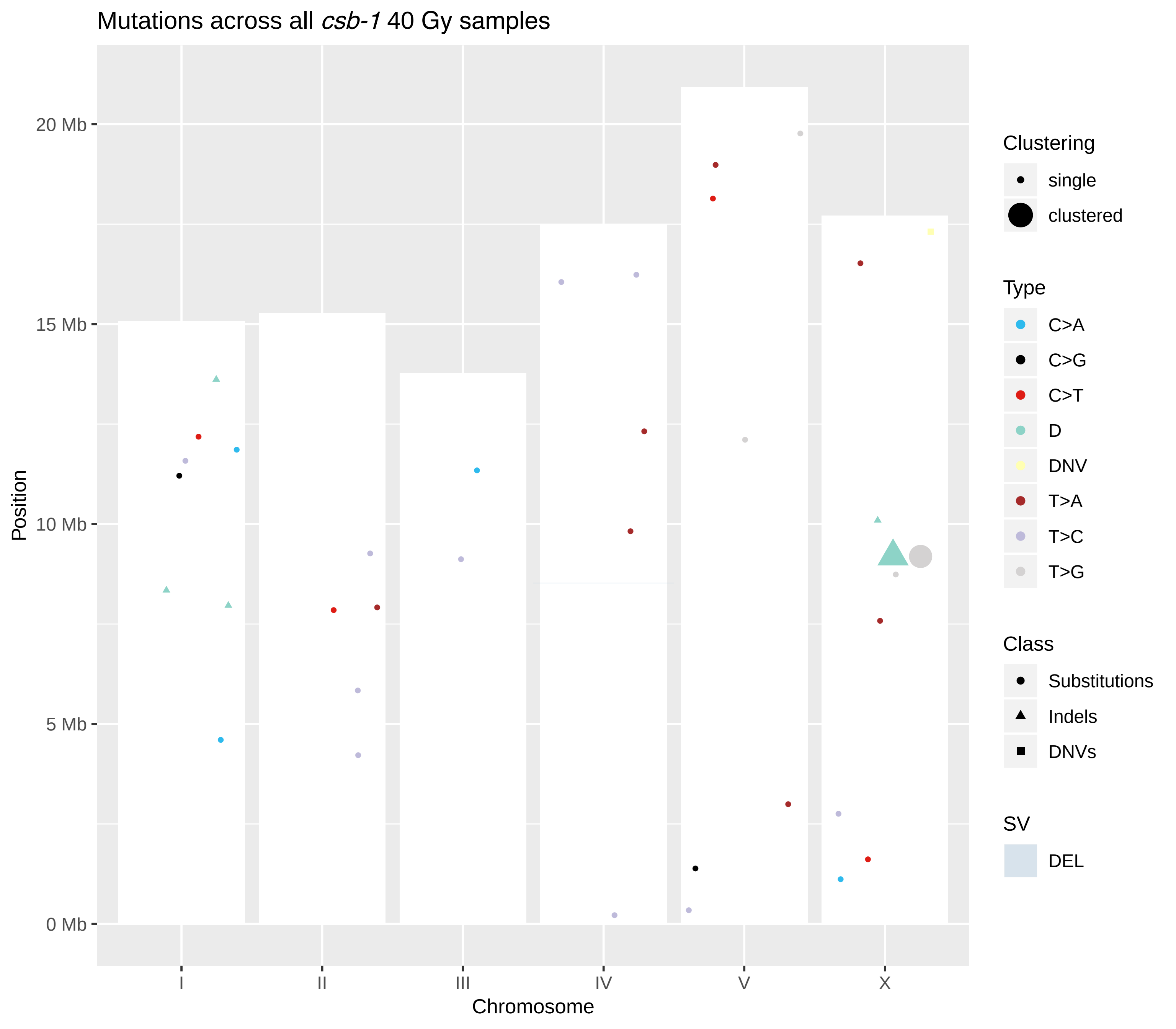

Mutations across all csb-1 40 Gy samples
20 Mb
15 Mb
10 Mb
5 Mb
0 Mb
Position
I
II
III
IV
V
X
Chromosome
Clustering
single
clustered
Type
C>A
C>G
C>T
D
DNV
T>A
T>C
T>G
Class
Substitutions
Indels
DNVs
SV
DEL

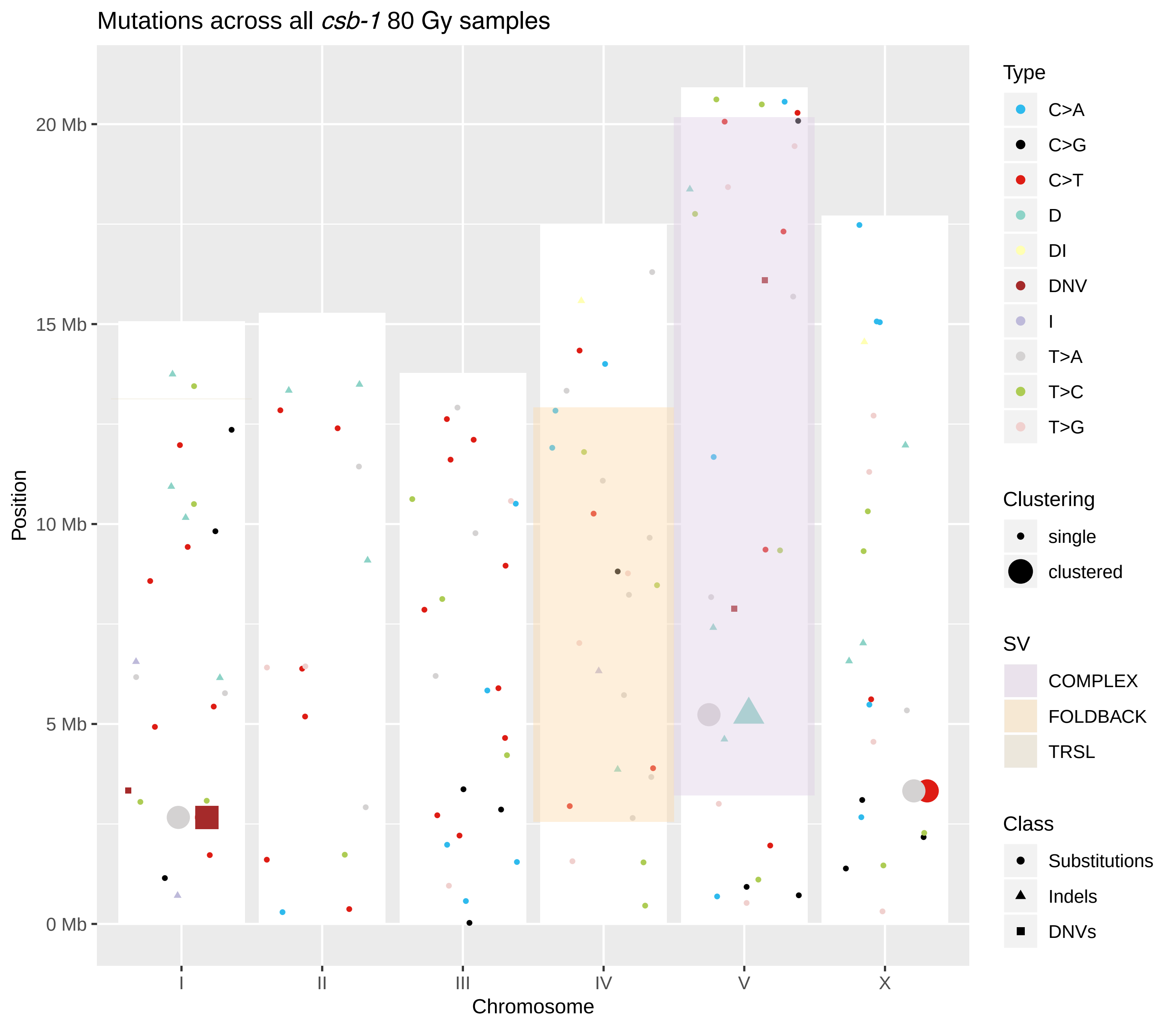

Mutations across all csb-1 80 Gy samples
Position
20 Mb
15 Mb
10 Mb
5 Mb
0 Mb
I
II
III
IV
V
X
Chromosome
Type
C>A
C>G
C>T
D
DI
DNV
I
T>A
T>C
T>G
Clustering
single
clustered
SV
COMPLEX
FOLDBACK
TRSL
Class
Substitutions
Indels
DNVs

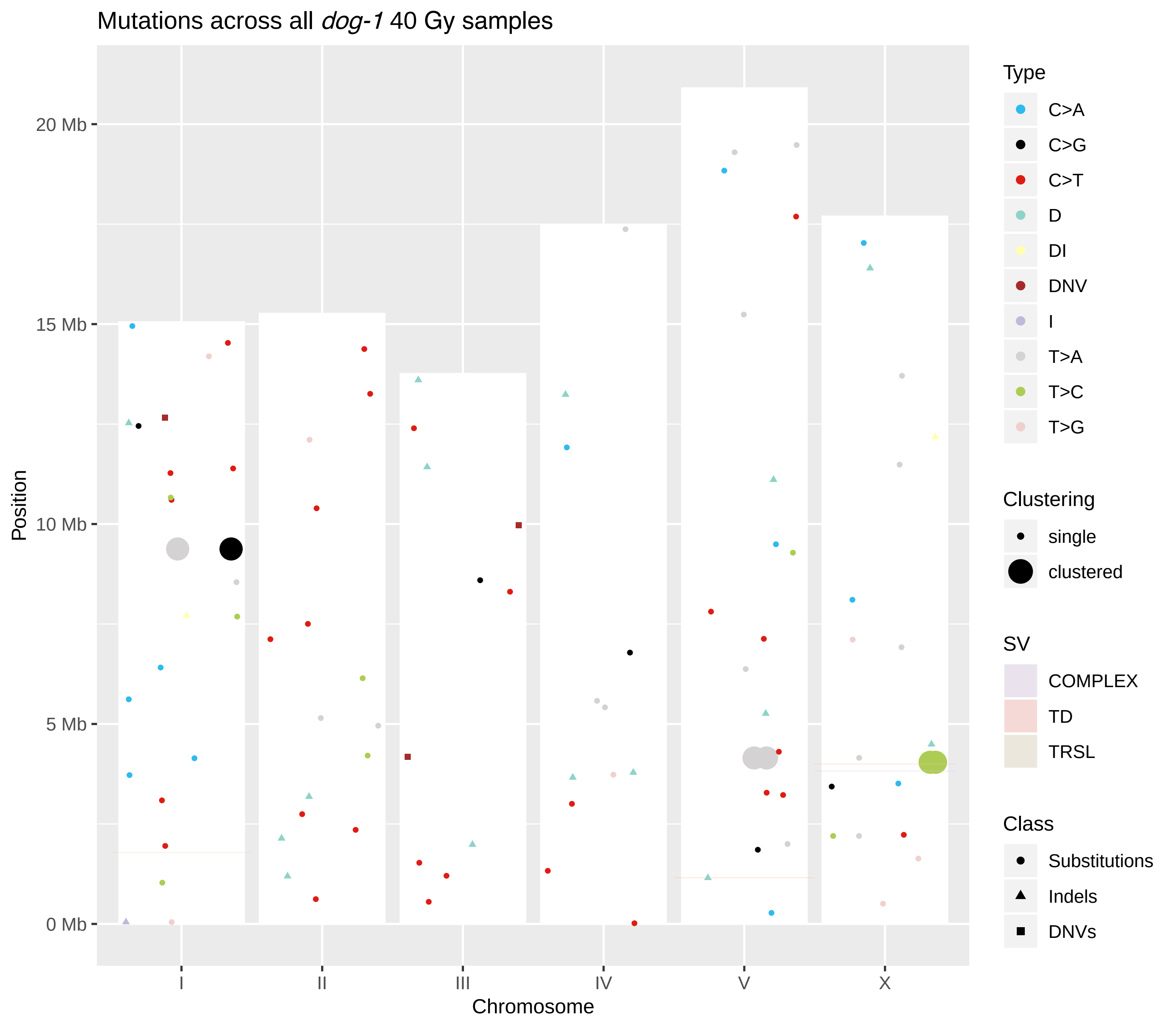
Mutations across all dog-1 40 Gy samples
Position
20 Mb
15 Mb
10 Mb
5 Mb
0 Mb
I
II
III
IV
V
X
Chromosome
Type
C>A
C>G
C>T
D
DI
DNV
I
T>A
T>C
T>G
Clustering
single
clustered
SV
COMPLEX
TD
TRSL
Class
Substitutions
Indels
DNVs

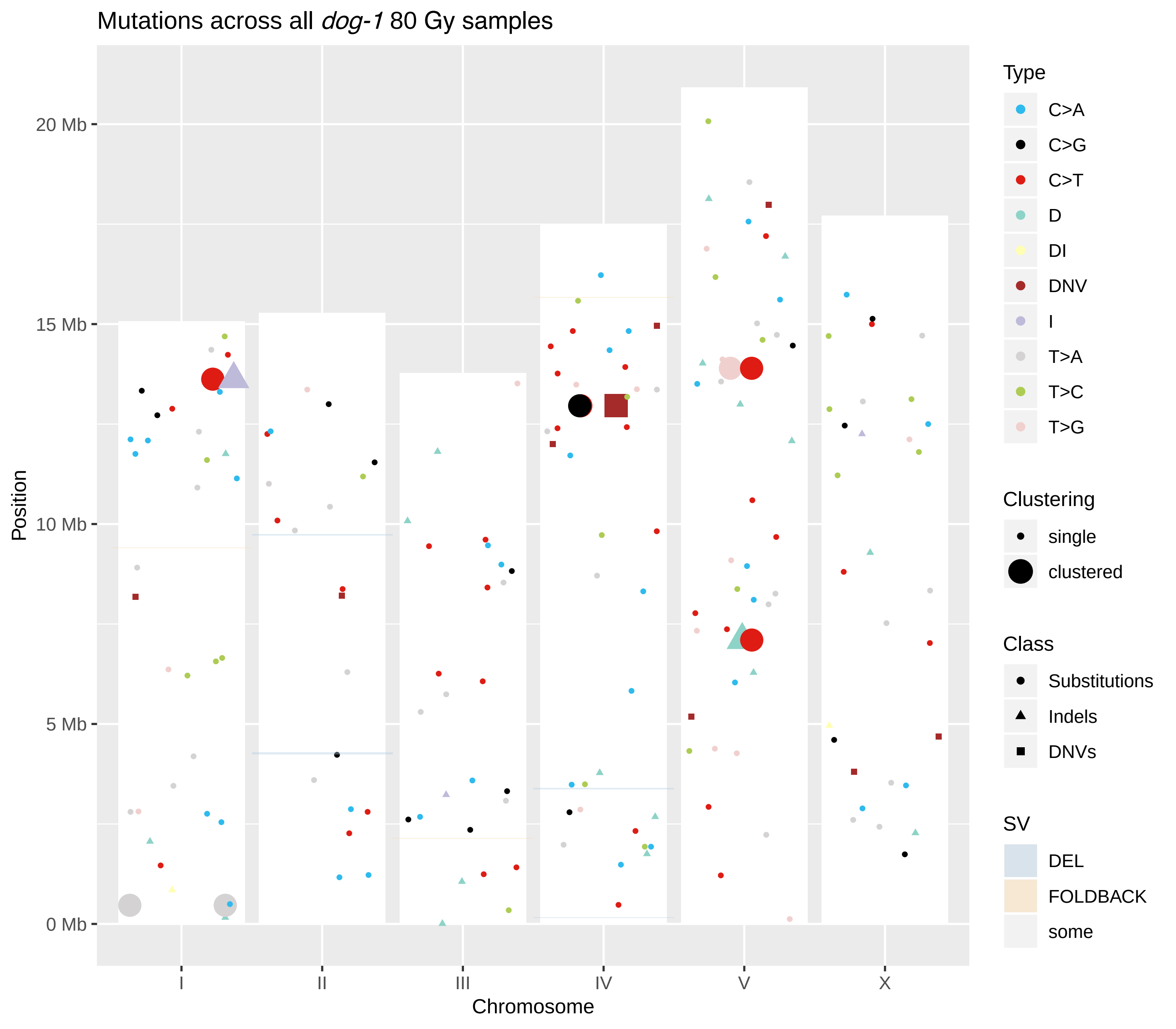
Mutations across all dog-1 80 Gy samples
Position
20 Mb
15 Mb
10 Mb
5 Mb
0 Mb
Chromosome
I
II
III
IV
V
X
Type
C>A
C>G
C>T
D
DI
DNV
I
T>A
T>C
T>G
Clustering
single
clustered
Class
Substitutions
Indels
DNVs
SV
DEL
FOLDBACK
some

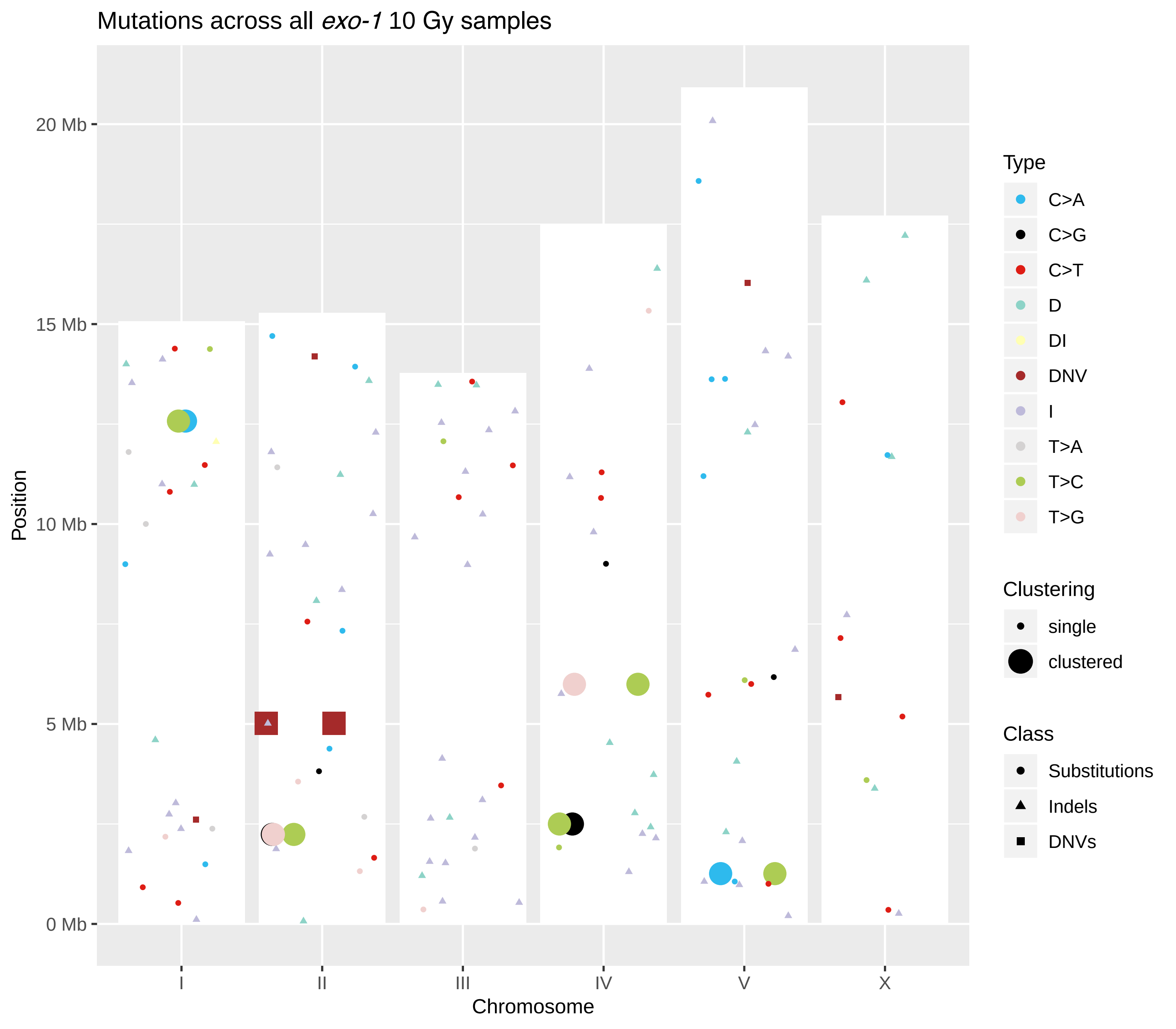
Mutations across all exo-1 10 Gy samples
20 Mb
15 Mb
10 Mb
5 Mb
0 Mb
Position
I
II
III
IV
V
X
Chromosome
Type
C>A
C>G
C>T
D
DI
DNV
I
T>A
T>C
T>G
Clustering
single
clustered
Class
Substitutions
Indels
DNVs

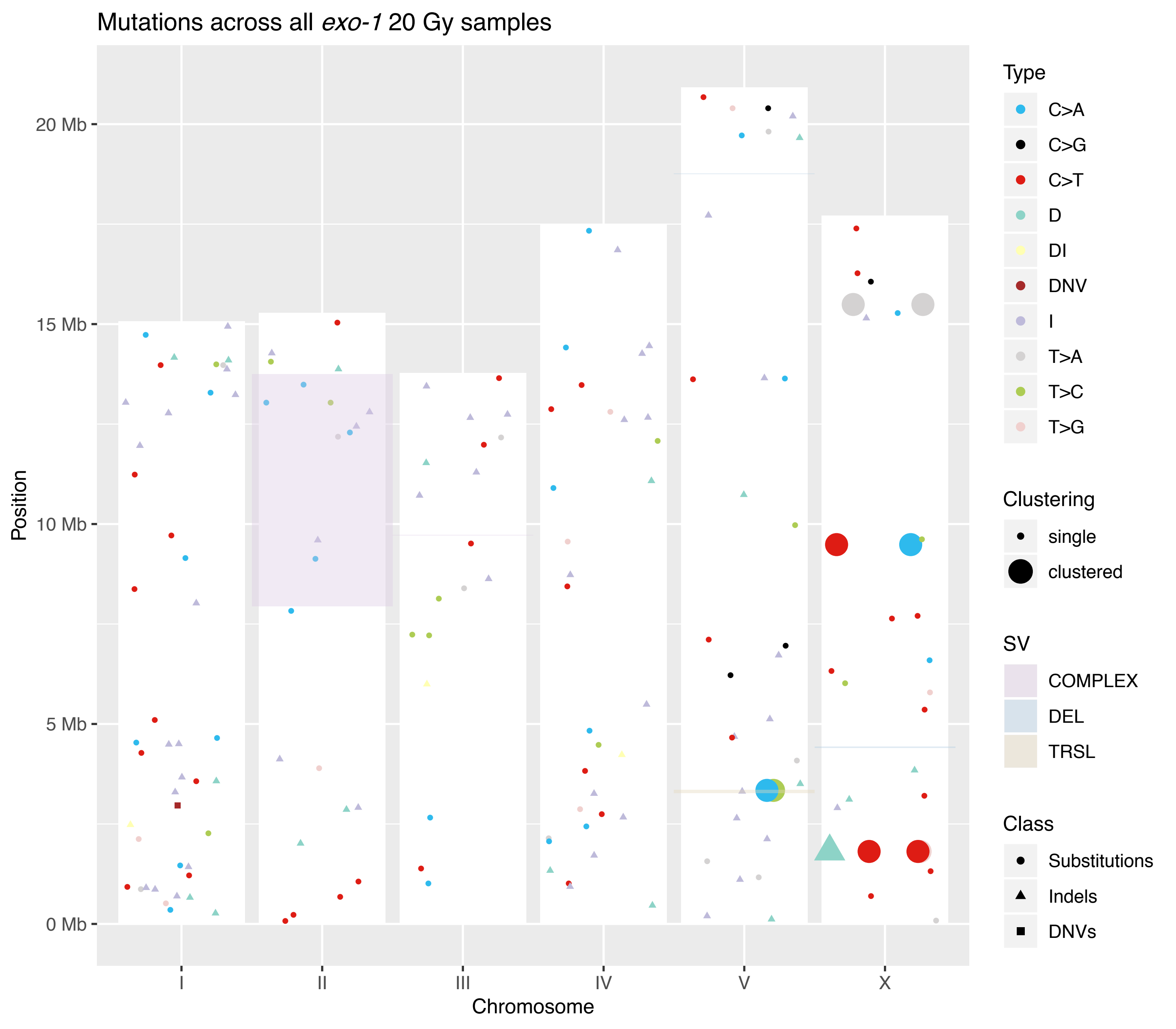

Mutations across all exo-1 20 Gy samples
Position
20 Mb
15 Mb
10 Mb
5 Mb
0 Mb
Chromosome
I
II
III
IV
V
X
Type
C>A
C>G
C>T
D
DI
DNV
I
T>A
T>C
T>G
Clustering
single
clustered
SV
COMPLEX
DEL
TRSL
Class
Substitutions
Indels
DNVs

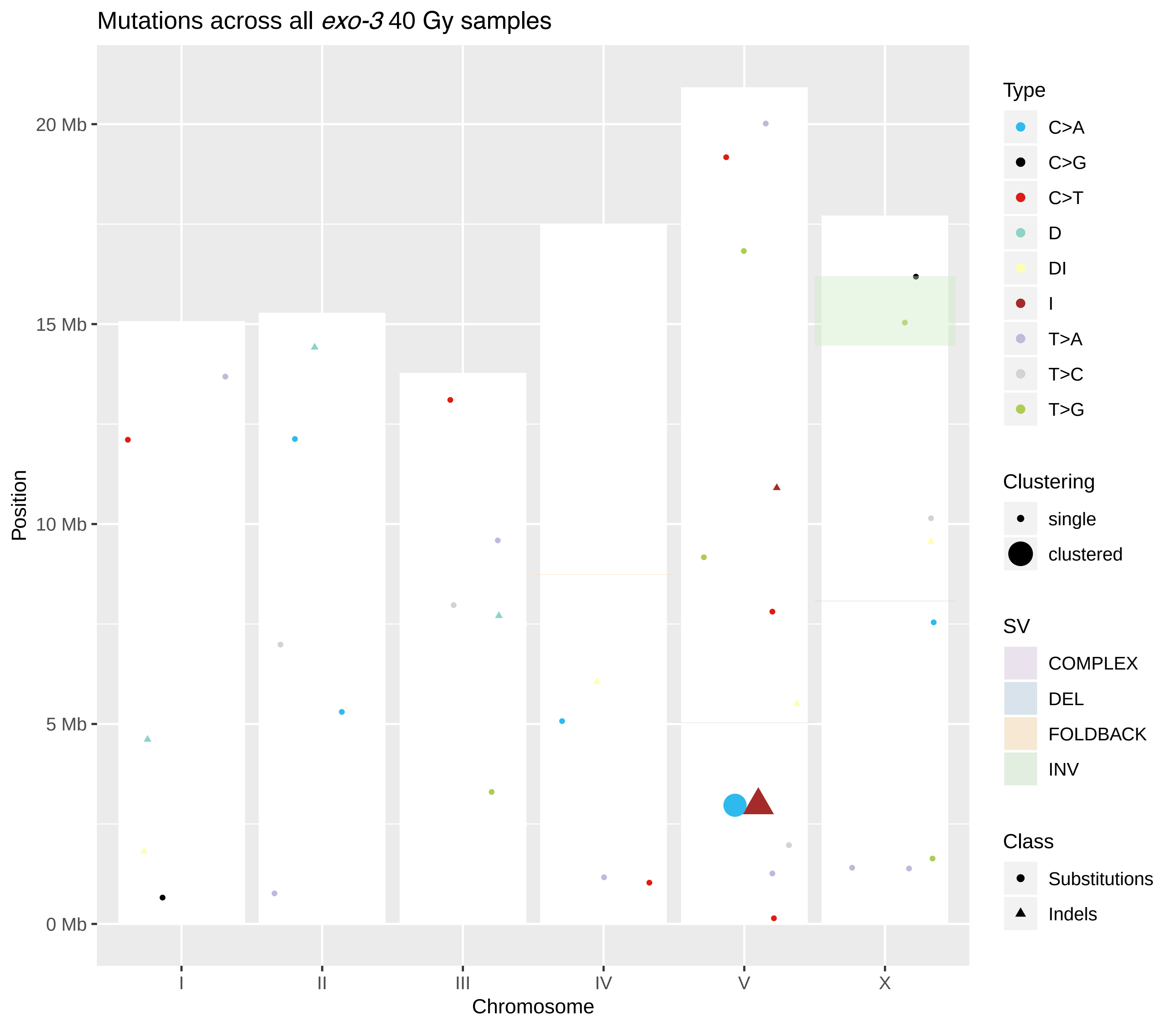
Mutations across all exo-3 40 Gy samples
Position
20 Mb
15 Mb
10 Mb
5 Mb
0 Mb
I
II
III
IV
V
X
Chromosome
Type
C>A
C>G
C>T
D
DI
I
T>A
T>C
T>G
Clustering
single
clustered
SV
COMPLEX
DEL
FOLDBACK
INV
Class
Substitutions
Indels

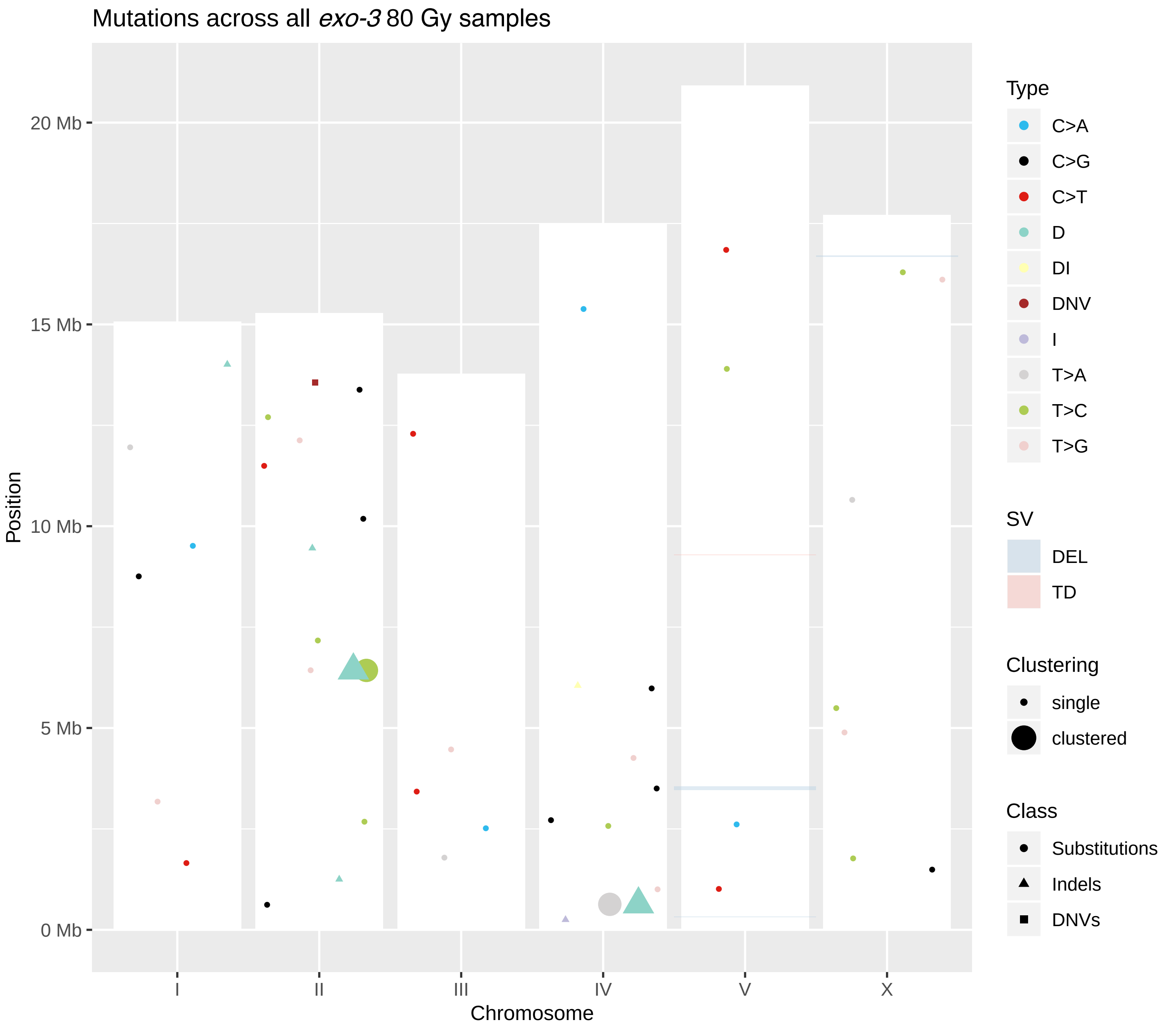
Mutations across all exo-3 80 Gy samples
Position
20 Mb
15 Mb
10 Mb
5 Mb
0 Mb
I
II
III
IV
V
X
Chromosome
Type
C>A
C>G
C>T
D
DI
DNV
I
T>A
T>C
T>G
SV
DEL
TD
Clustering
single
clustered
Class
Substitutions
Indels
DNVs

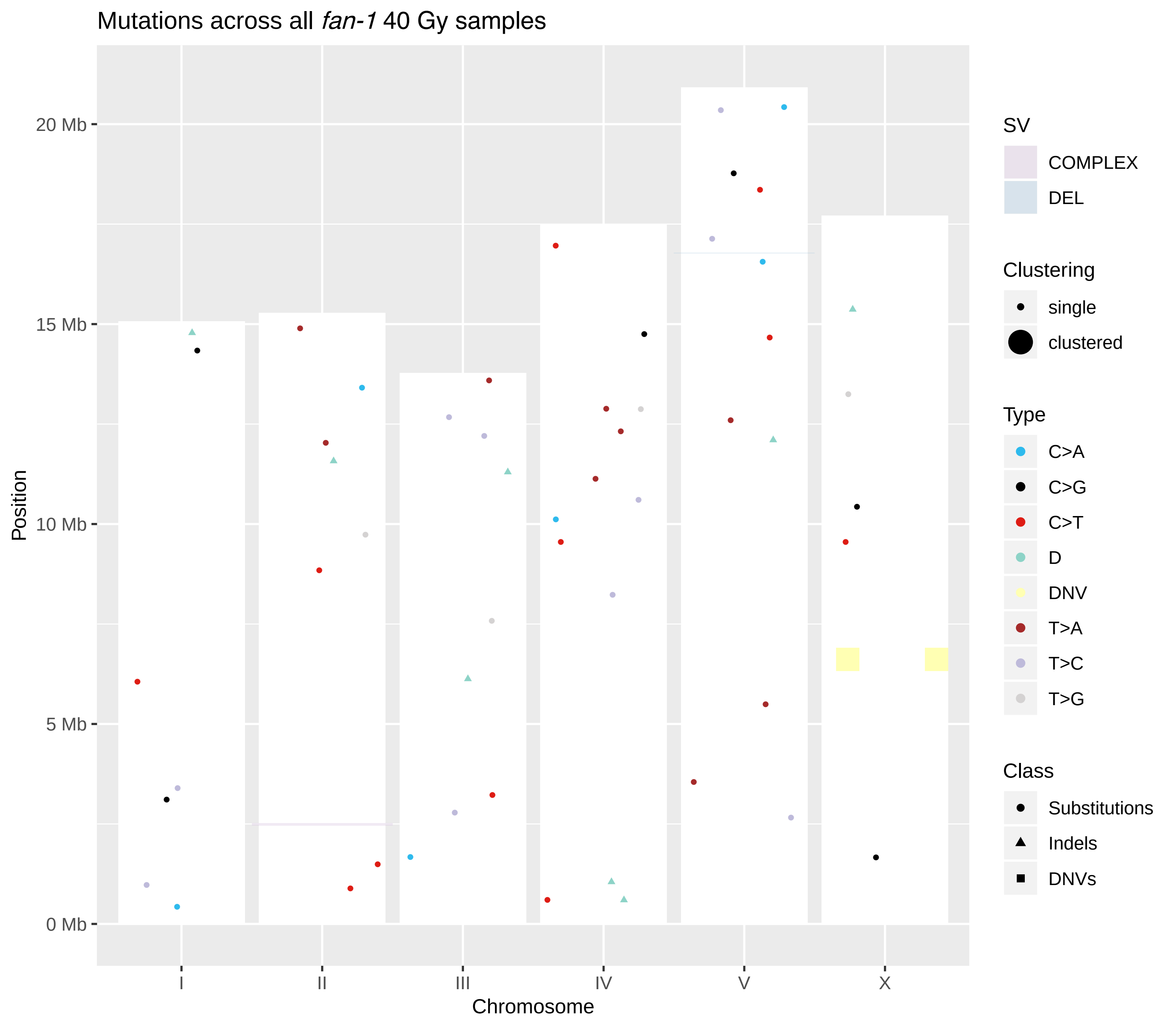
Mutations across all fan-1 40 Gy samples
Position
20 Mb
15 Mb
10 Mb
5 Mb
0 Mb
I
II
III
IV
V
X
Chromosome
SV
COMPLEX
DEL
Clustering
single
clustered
Type
C>A
C>G
C>T
D
DNV
T>A
T>C
T>G
Class
Substitutions
Indels
DNVs

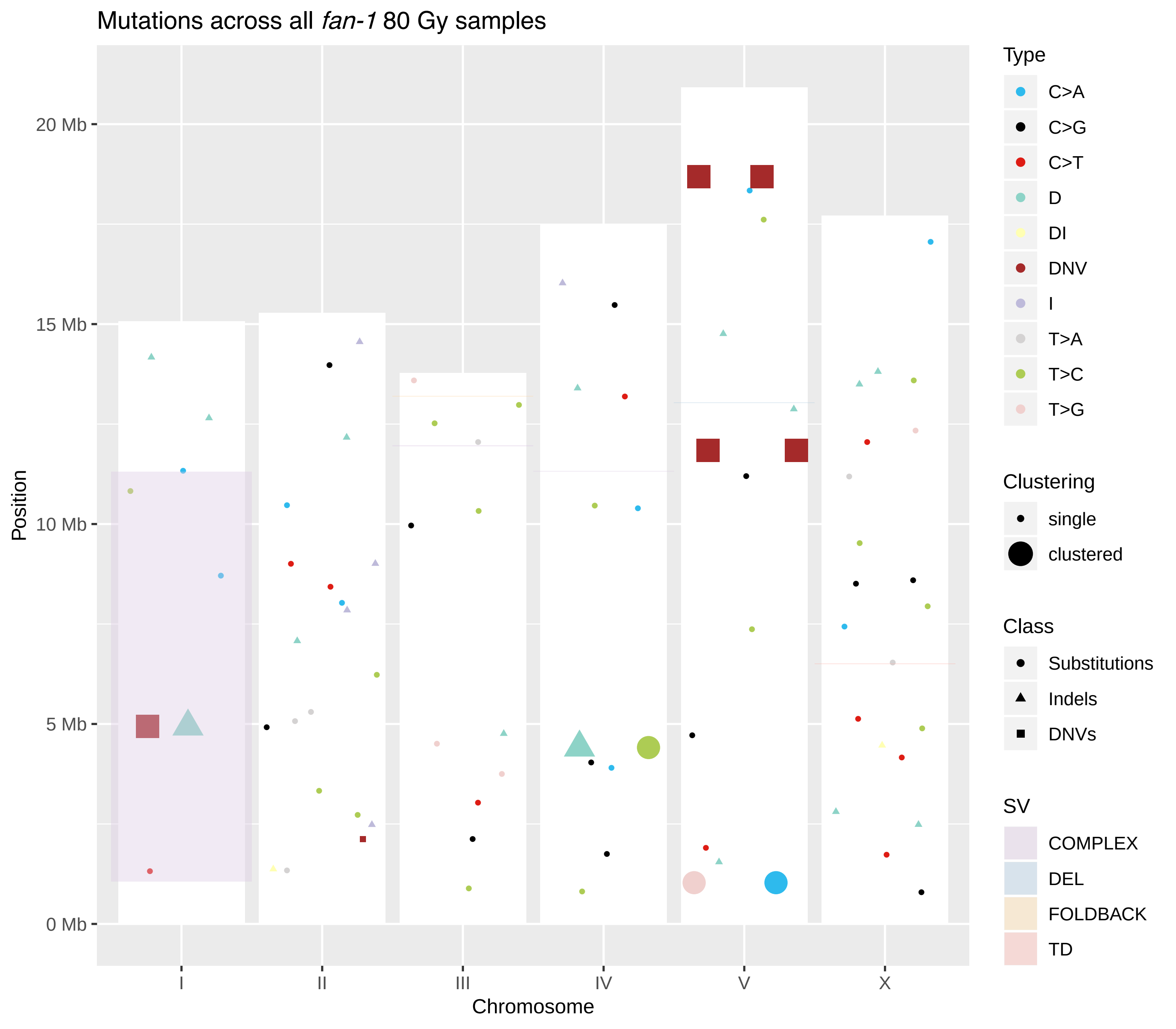

Mutations across all *fan-1* 80 Gy samples
Position
20 Mb
15 Mb
10 Mb
5 Mb
0 Mb
Chromosome
I
II
III
IV
V
X
Type
C>A
C>G
C>T
D
DI
DNV
I
T>A
T>C
T>G
Clustering
single
clustered
Class
Substitutions
Indels
DNVs
SV
COMPLEX
DEL
FOLDBACK
TD

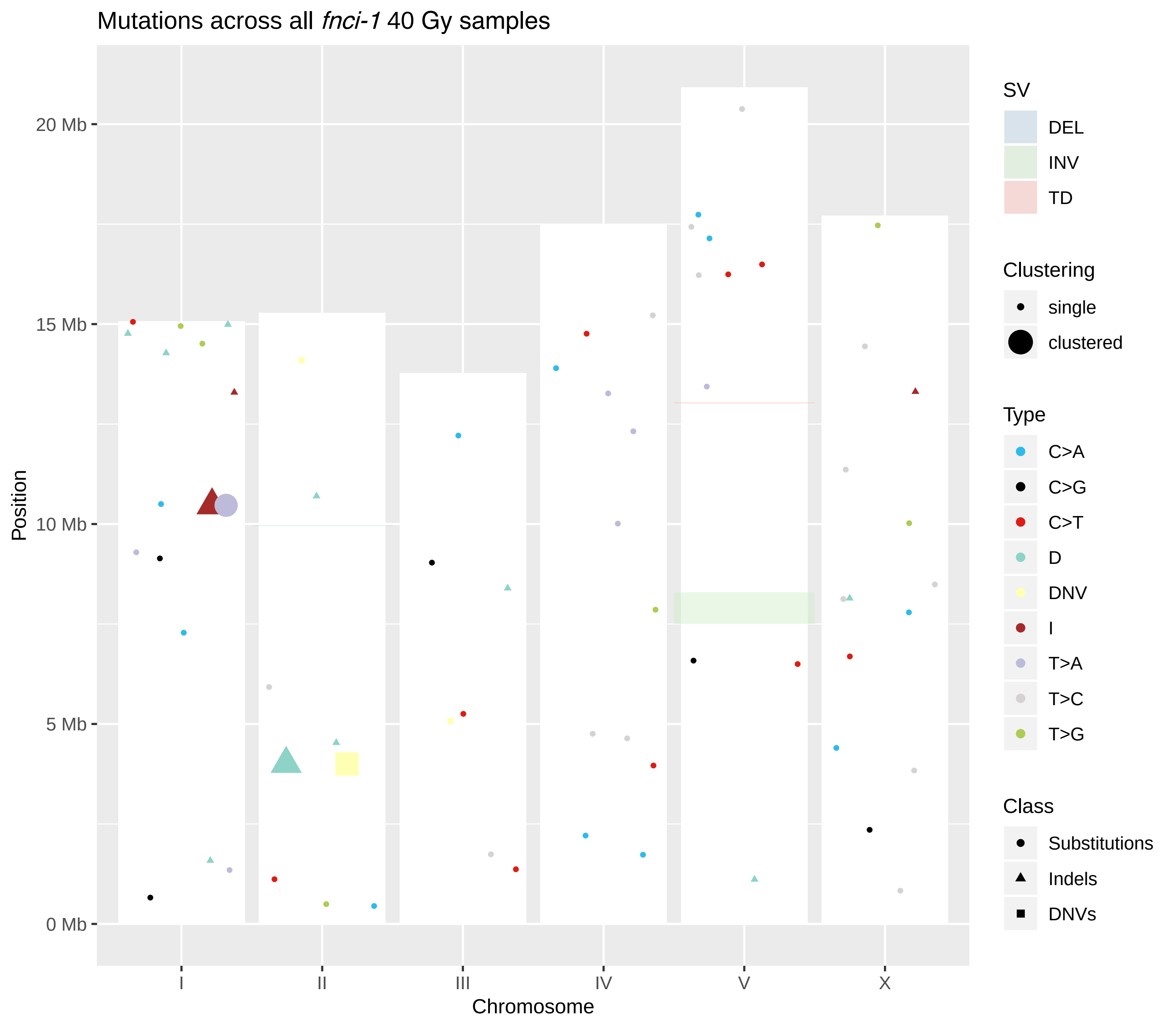
Mutations across all fnci-1 40 Gy samples
Position
20 Mb
15 Mb
10 Mb
5 Mb
0 Mb
I
II
III
IV
V
X
Chromosome
SV
DEL
INV
TD
Clustering
single
clustered
Type
C>A
C>G
C>T
D
DNV
I
T>A
T>C
T>G
Class
Substitutions
Indels
DNVs

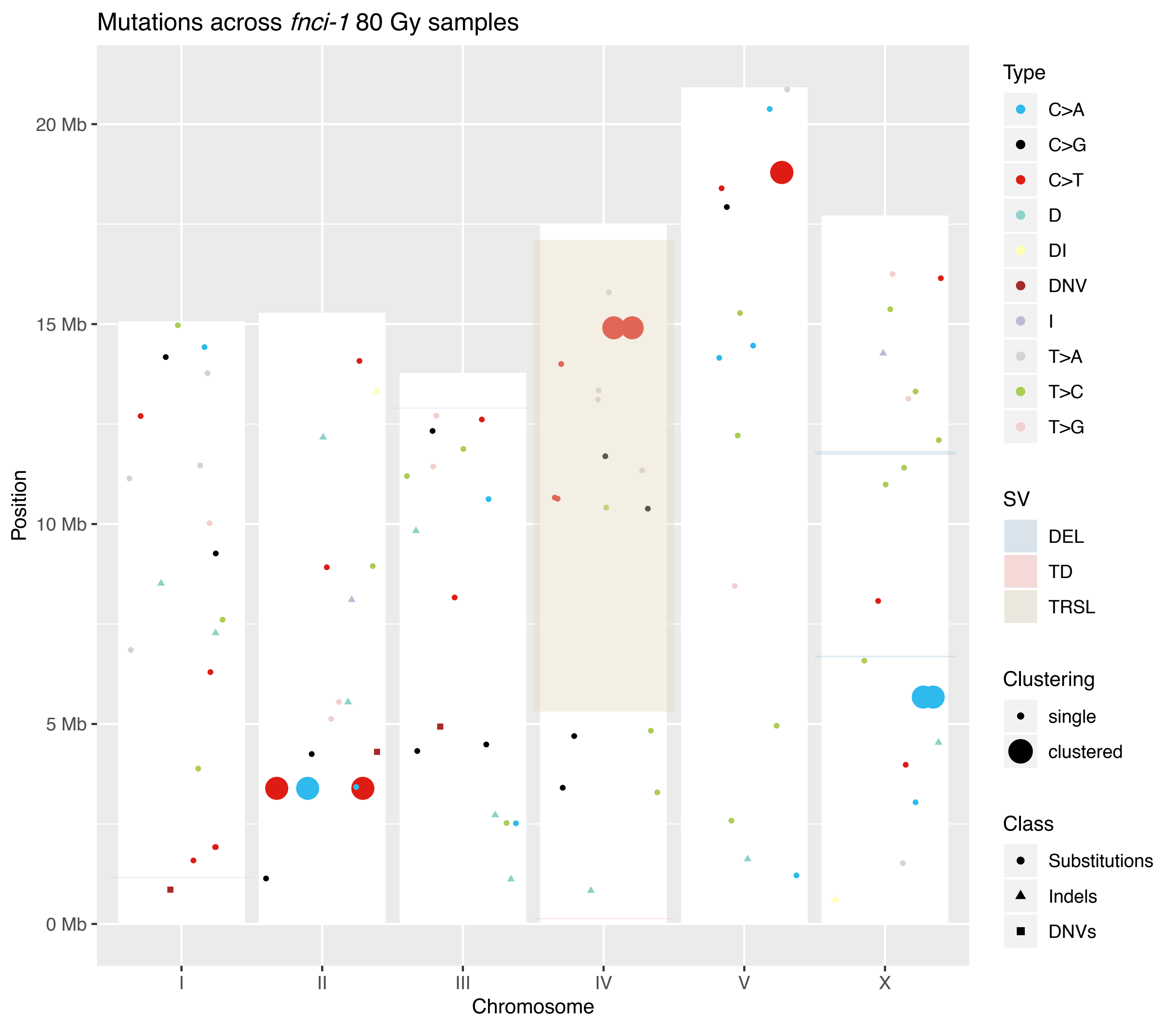

Mutations across fnci-1 80 Gy samples
Position
20 Mb
15 Mb
10 Mb
5 Mb
0 Mb
I
II
III
IV
V
X
Chromosome
Type
C>A
C>G
C>T
D
DI
DNV
I
T>A
T>C
T>G
SV
DEL
TD
TRSL
Clustering
single
clustered
Class
Substitutions
Indels
DNVs

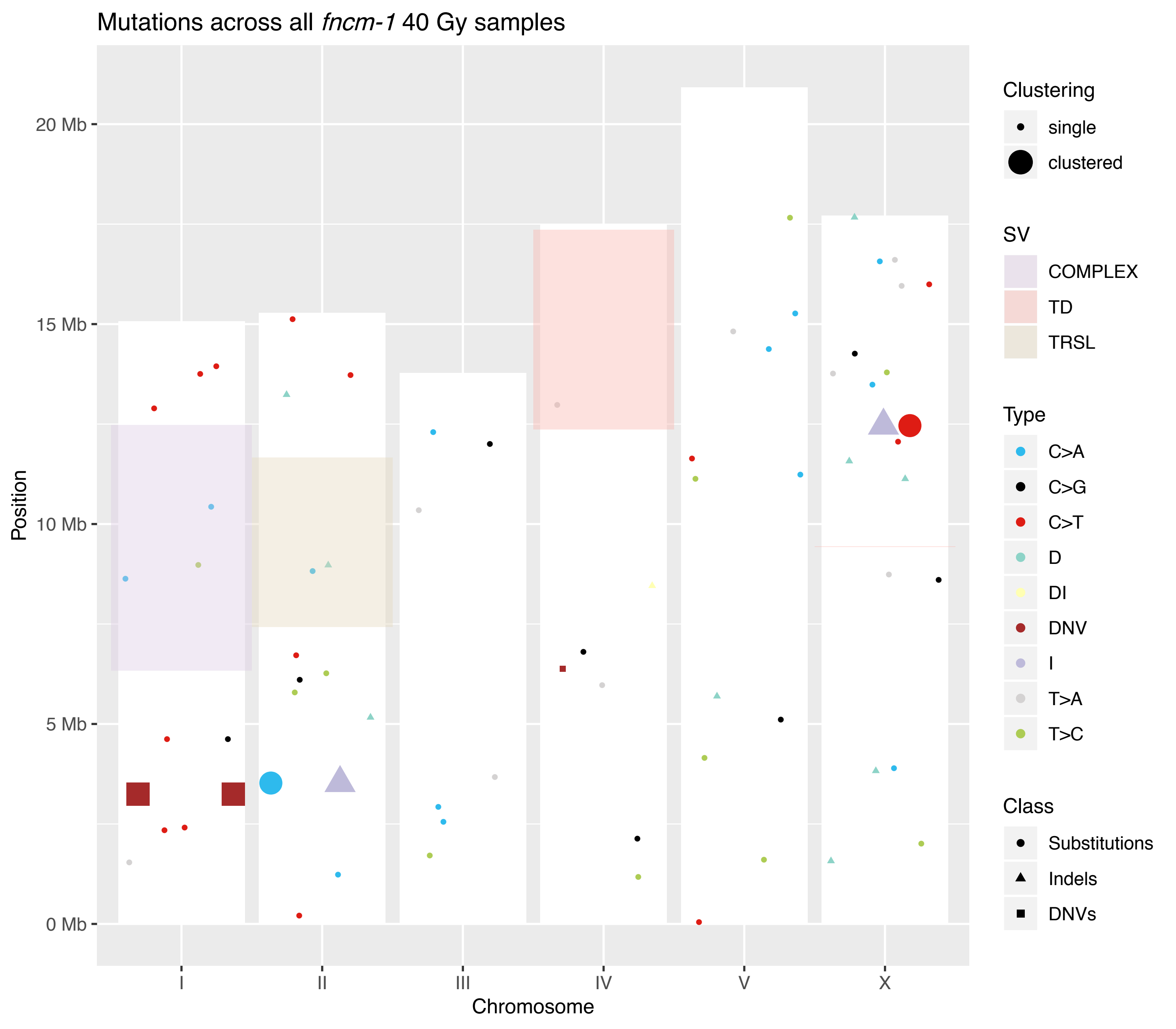
Mutations across all fncm-1 40 Gy samples
Position
20 Mb
15 Mb
10 Mb
5 Mb
0 Mb
I
II
III
IV
V
X
Chromosome
Clustering
single
clustered
SV
COMPLEX
TD
TRSL
Type
C>A
C>G
C>T
D
DI
DNV
I
T>A
T>C
Class
Substitutions
Indels
DNVs

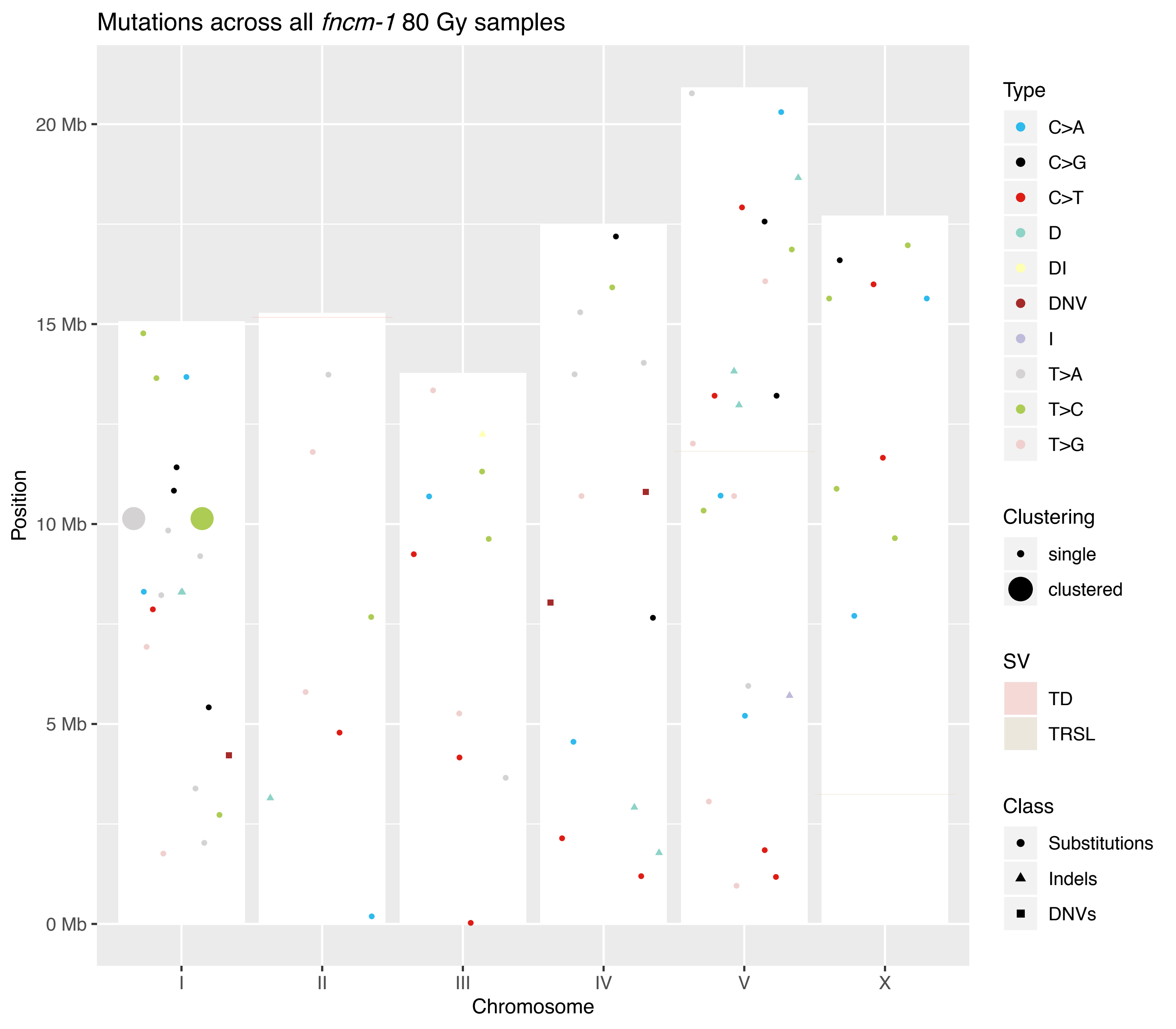

Mutations across all fncm-1 80 Gy samples
Position
20 Mb
15 Mb
10 Mb
5 Mb
0 Mb
I
II
III
IV
V
X
Chromosome
Type
C>A
C>G
C>T
D
DI
DNV
I
T>A
T>C
T>G
Clustering
single
clustered
SV
TD
TRSL
Class
Substitutions
Indels
DNVs

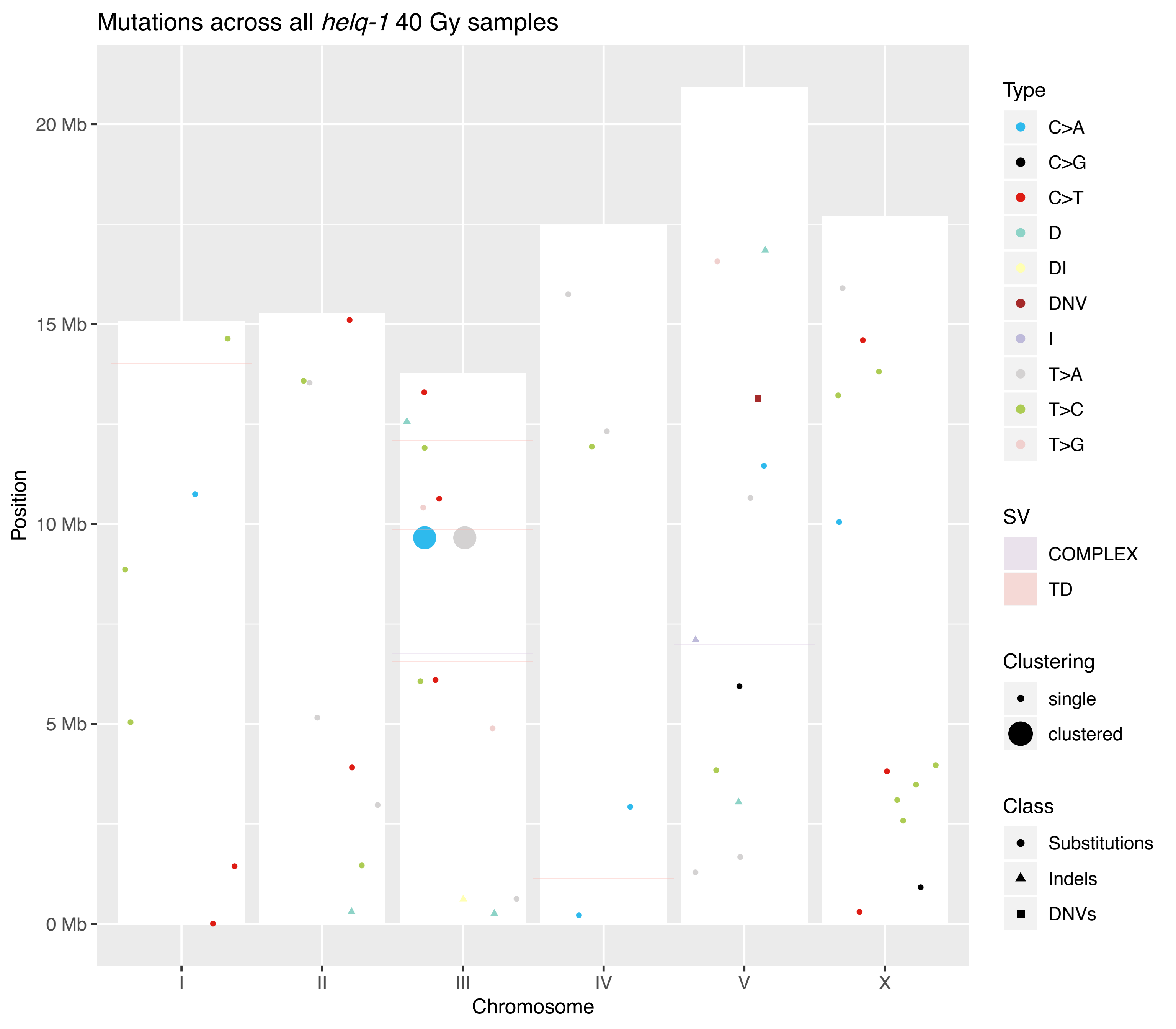
Mutations across all helq-1 40 Gy samples
20 Mb
15 Mb
10 Mb
5 Mb
0 Mb
Position
I
II
III
IV
V
X
Chromosome
Type
C>A
C>G
C>T
D
DI
DNV
I
T>A
T>C
T>G
SV
COMPLEX
TD
Clustering
single
clustered
Class
Substitutions
Indels
DNVs

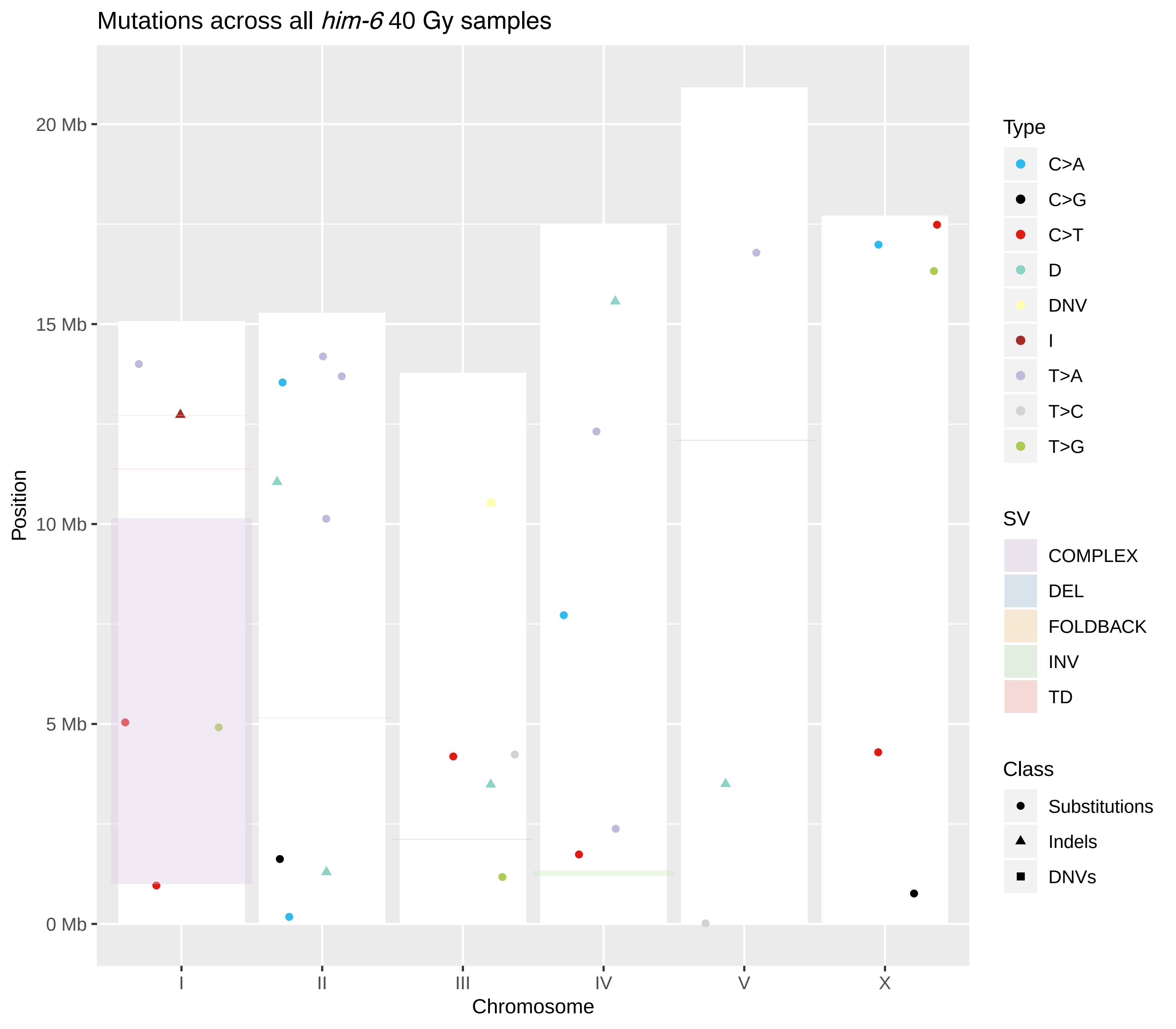

Mutations across all him-6 40 Gy samples
Position
20 Mb
15 Mb
10 Mb
5 Mb
0 Mb
I
II
III
IV
V
X
Chromosome
Type
C>A
C>G
C>T
D
DNV
I
T>A
T>C
T>G
SV
COMPLEX
DEL
FOLDBACK
INV
TD
Class
Substitutions
Indels
DNVs

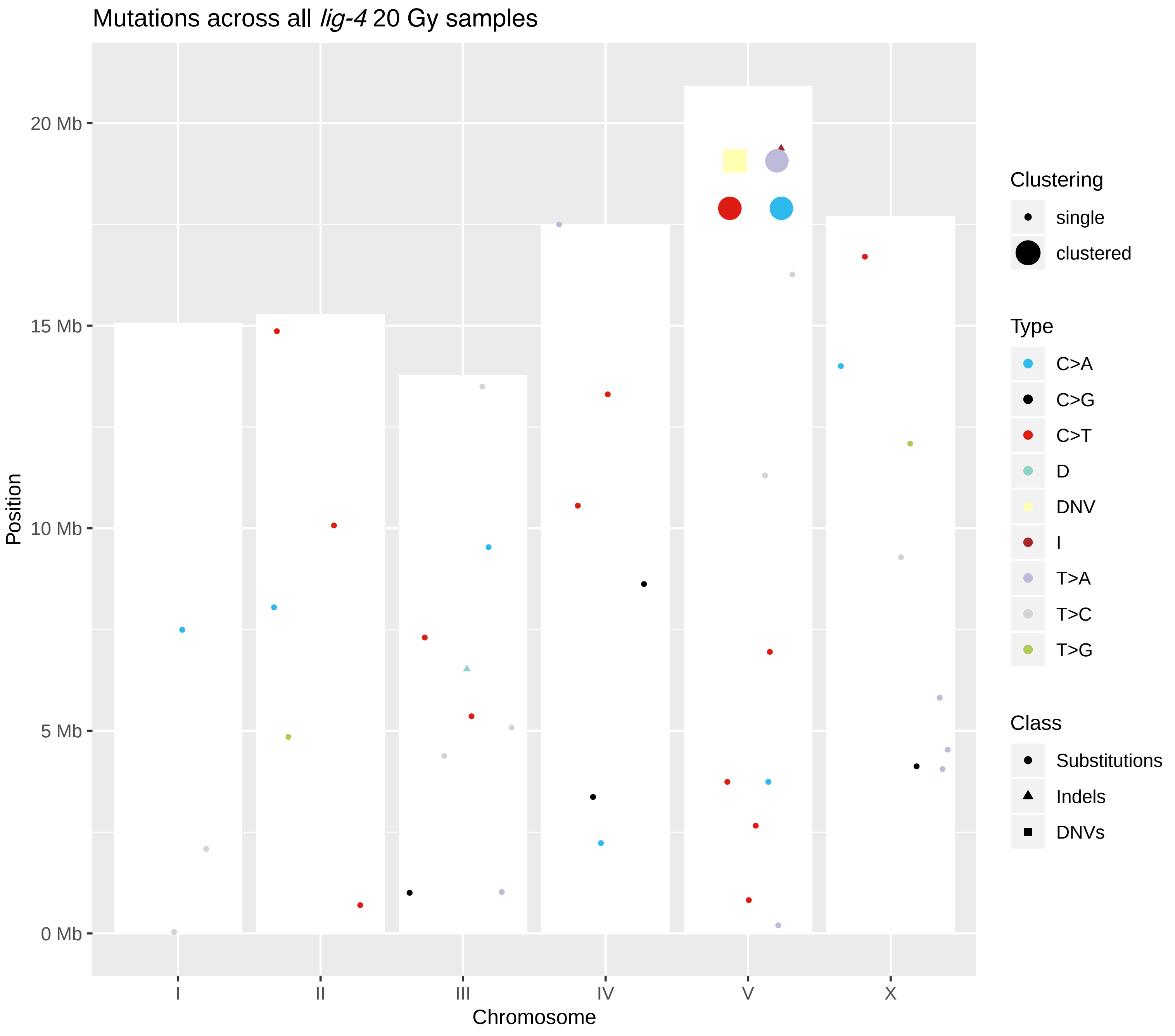

Mutations across all lig-4 20 Gy samples
20 Mb
15 Mb
10 Mb
5 Mb
0 Mb
Position
I
II
III
IV
V
X
Chromosome
Clustering
single
clustered
Type
C>A
C>G
C>T
D
DNV
I
T>A
T>C
T>G
Class
Substitutions
Indels
DNVs

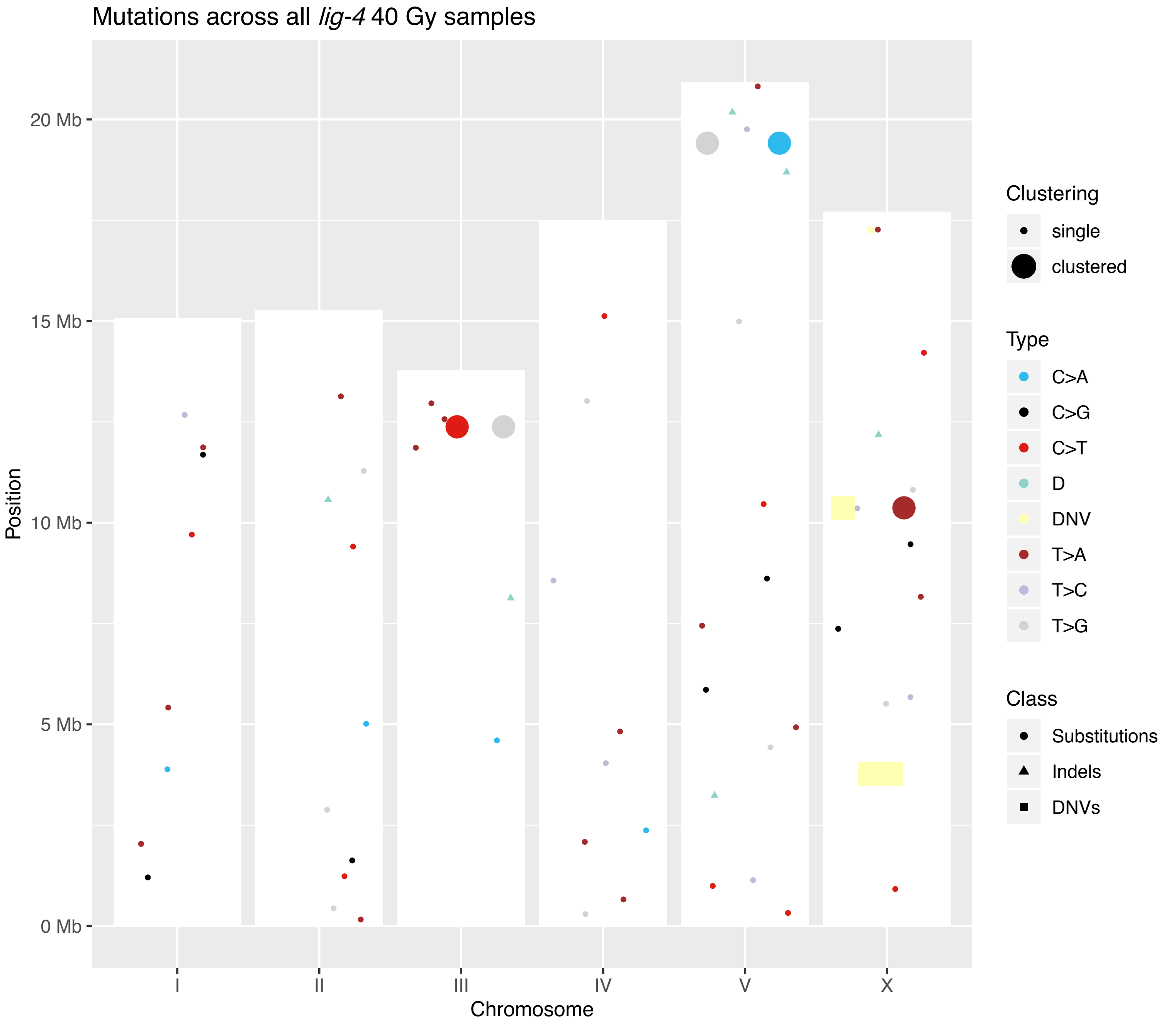

Mutations across all lig-4 40 Gy samples
Position
20 Mb
15 Mb
10 Mb
5 Mb
0 Mb
I
II
III
IV
V
X
Chromosome
Clustering
single
clustered
Type
C>A
C>G
C>T
D
DNV
T>A
T>C
T>G
Class
Substitutions
Indels
DNVs

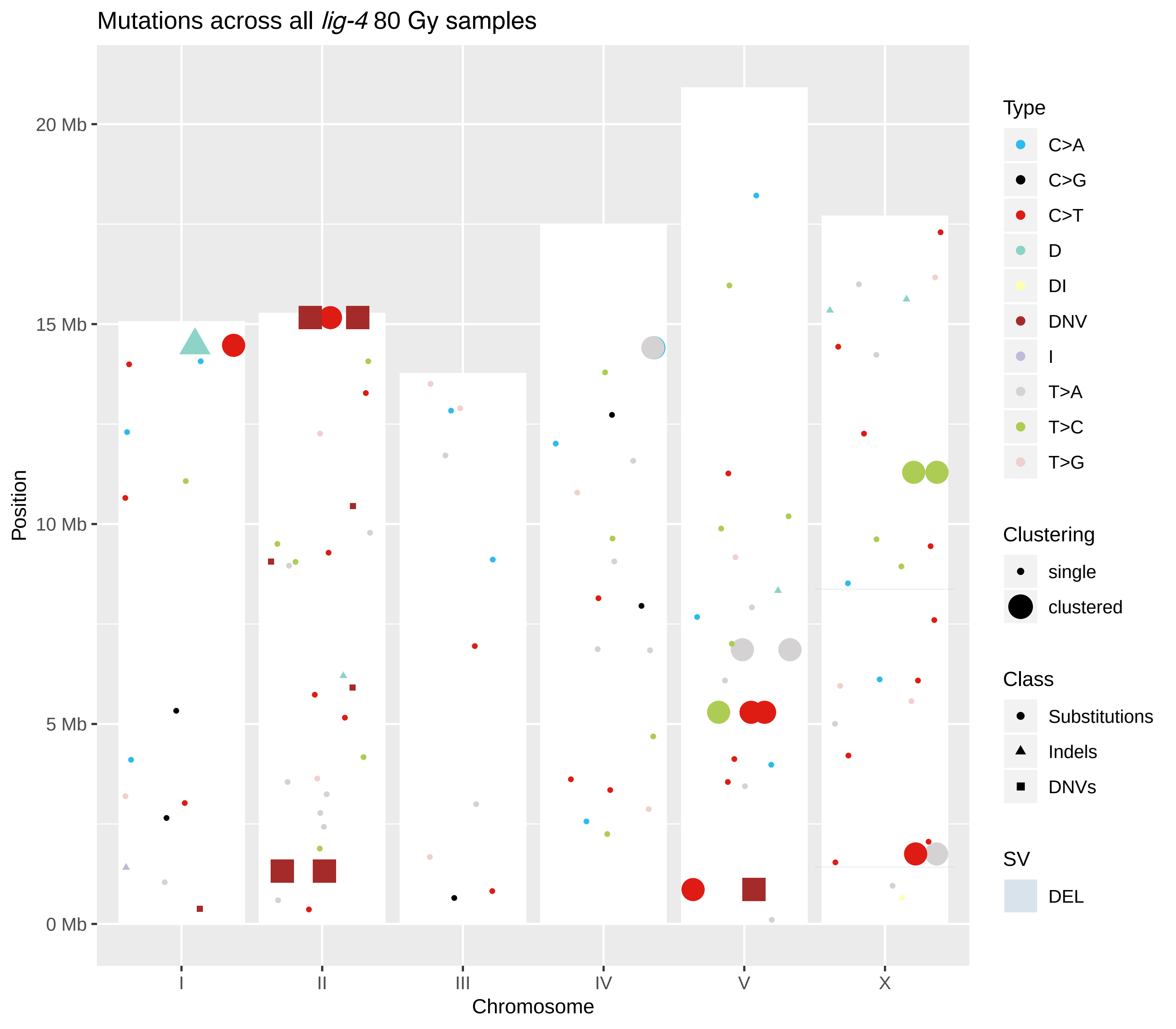

Mutations across all lig-4 80 Gy samples
Position
20 Mb
15 Mb
10 Mb
5 Mb
0 Mb
I
II
III
IV
V
X
Chromosome
Type
C>A
C>G
C>T
D
DI
DNV
I
T>A
T>C
T>G
Clustering
single
clustered
Class
Substitutions
Indels
DNVs
SV
DEL

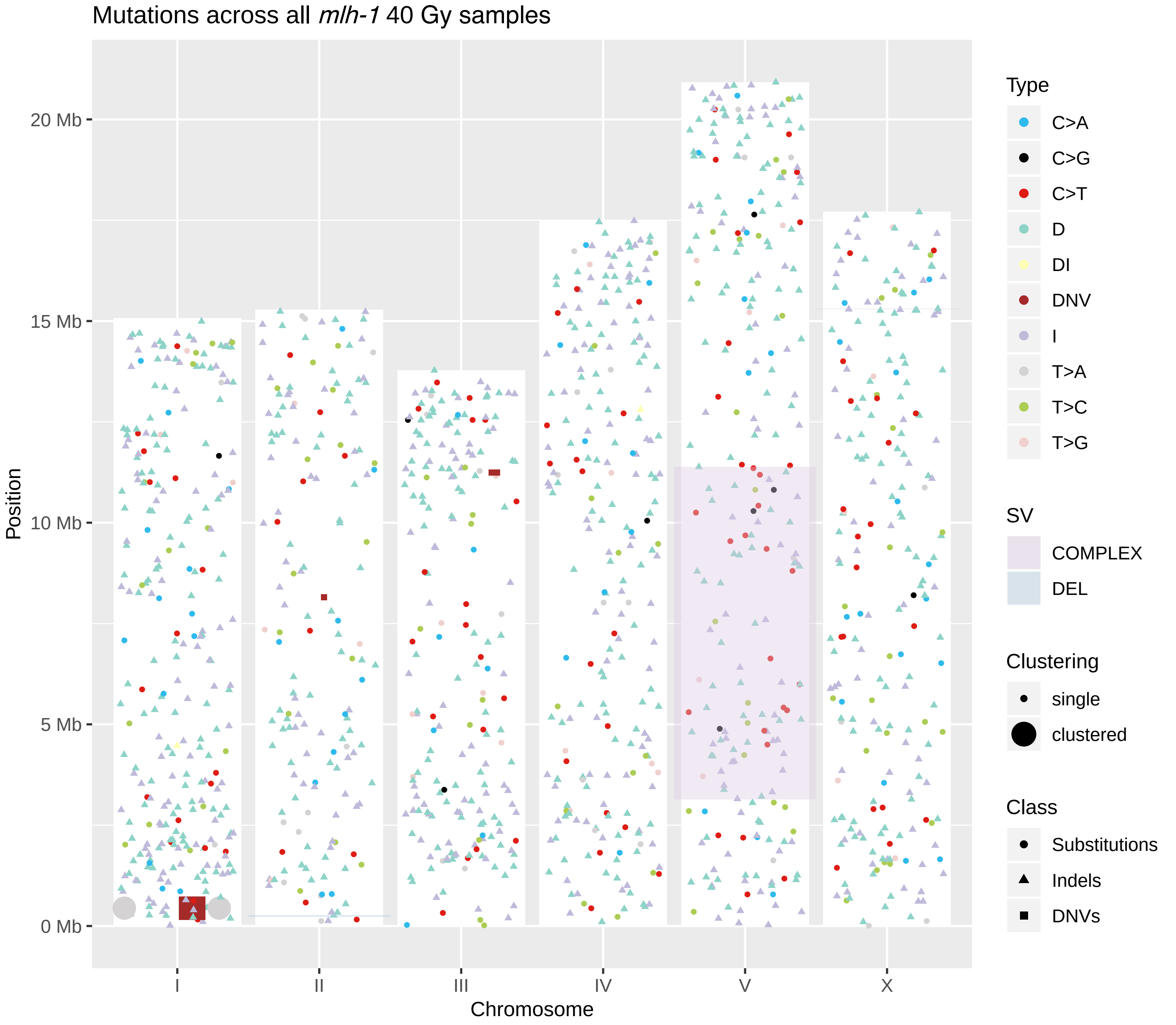

Mutations across all mlh-1 40 Gy samples
Position
20 Mb
15 Mb
10 Mb
5 Mb
0 Mb
I
II
III
IV
V
X
Chromosome
Type
C>A
C>G
C>T
D
DI
DNV
I
T>A
T>C
T>G
SV
COMPLEX
DEL
Clustering
single
clustered
Class
Substitutions
Indels
DNVs

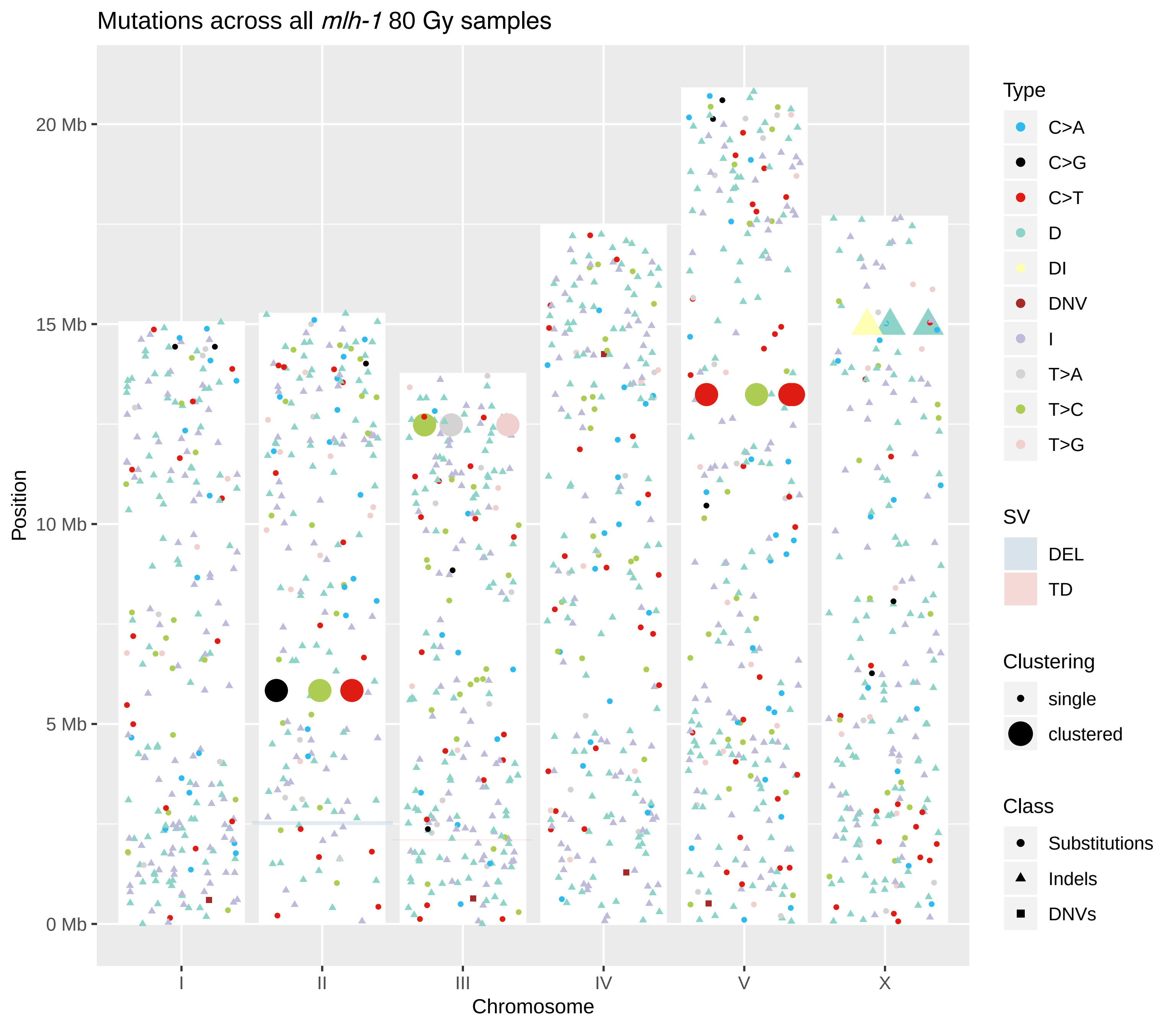

Mutations across all mlh-1 80 Gy samples
Position
20 Mb
15 Mb
10 Mb
5 Mb
0 Mb
I
II
III
IV
V
X
Chromosome
Type
C>A
C>G
C>T
D
DI
DNV
I
T>A
T>C
T>G
SV
DEL
TD
Clustering
single
clustered
Class
Substitutions
Indels
DNVs

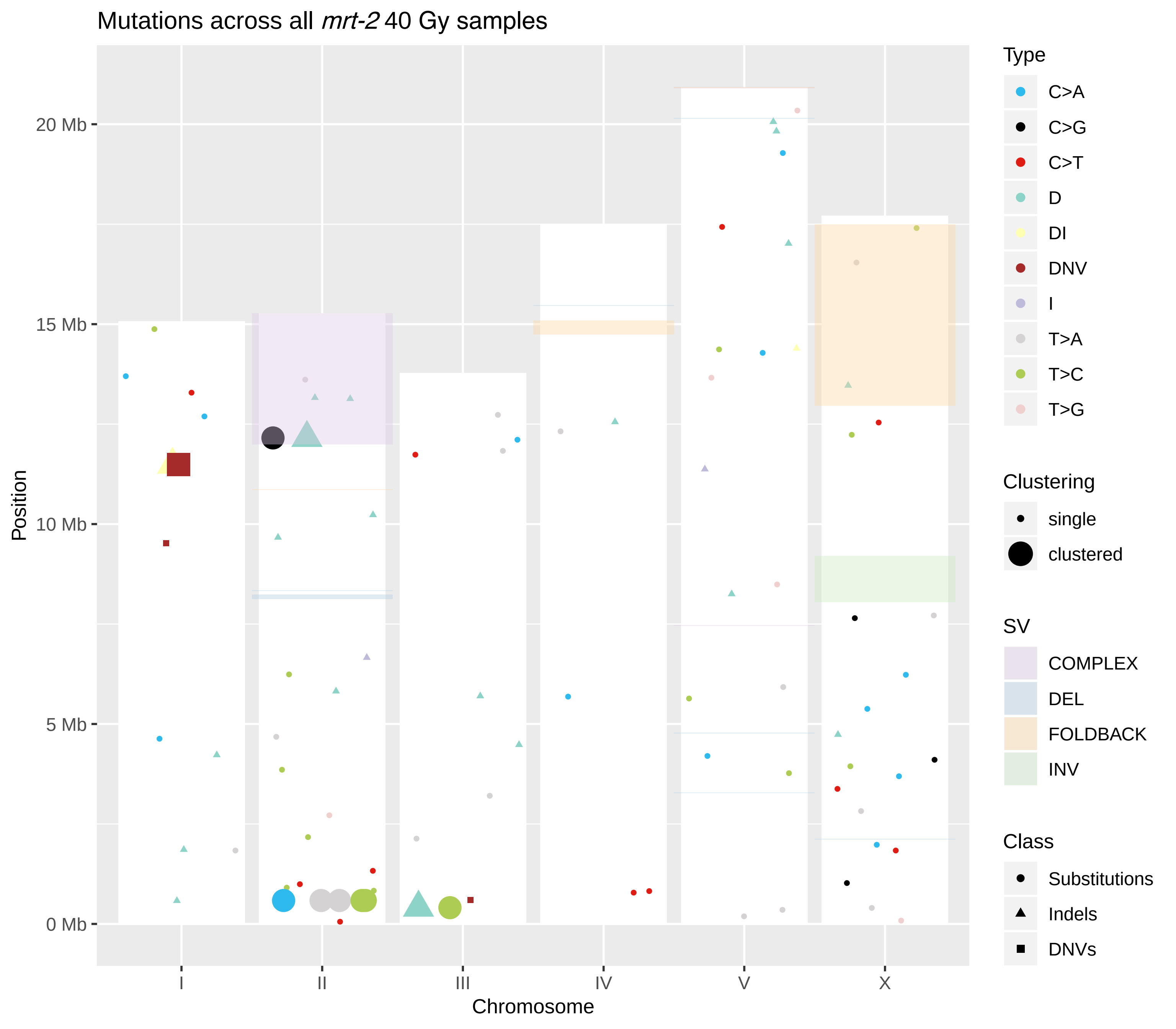
Mutations across all mrt-2 40 Gy samples
Position
20 Mb
15 Mb
10 Mb
5 Mb
0 Mb
Chromosome
I
II
III
IV
V
X
Type
C>A
C>G
C>T
D
DI
DNV
I
T>A
T>C
T>G
Clustering
single
clustered
SV
COMPLEX
DEL
FOLDBACK
INV
Class
Substitutions
Indels
DNVs

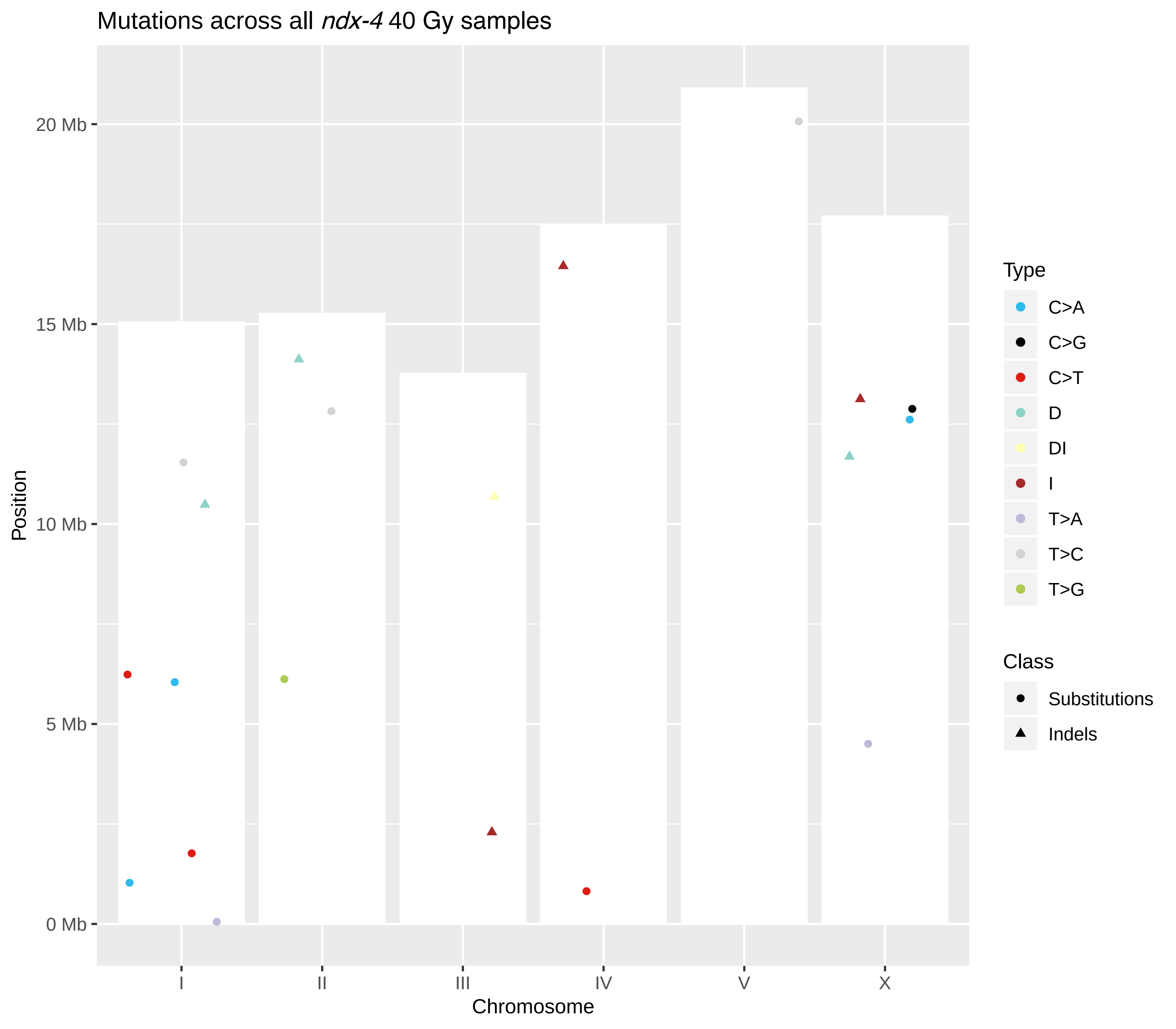
Mutations across all ndx-4 40 Gy samples
Position
20 Mb
15 Mb
10 Mb
5 Mb
0 Mb
I
II
III
IV
V
X
Chromosome
Type
C>A
C>G
C>T
D
DI
I
T>A
T>C
T>G
Class
Substitutions
Indels

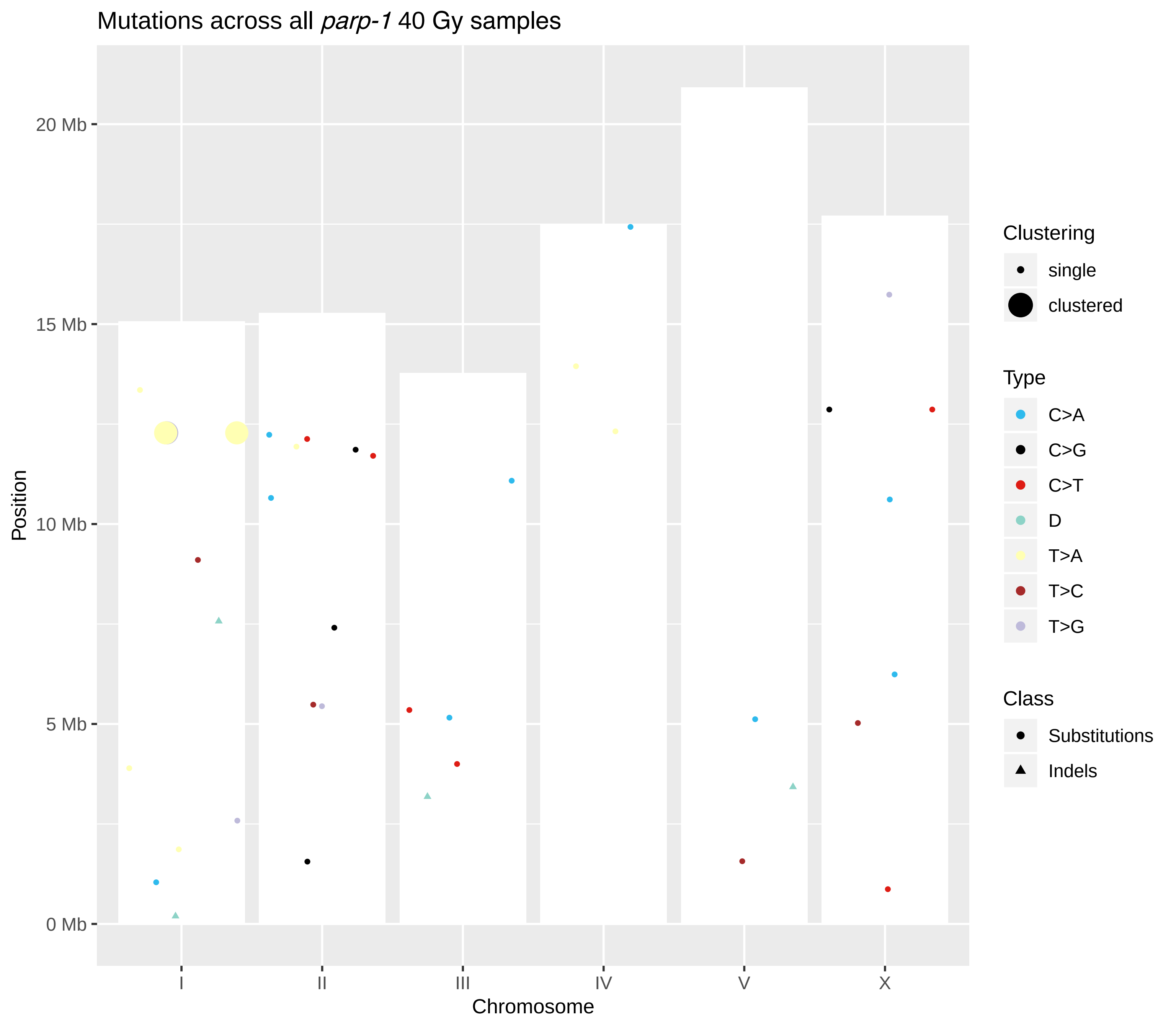
Mutations across all parp-1 40 Gy samples
20 Mb
15 Mb
10 Mb
5 Mb
0 Mb
Position
I
II
III
IV
V
X
Chromosome
Clustering
single
clustered
Type
C>A
C>G
C>T
D
T>A
T>C
T>G
Class
Substitutions
Indels

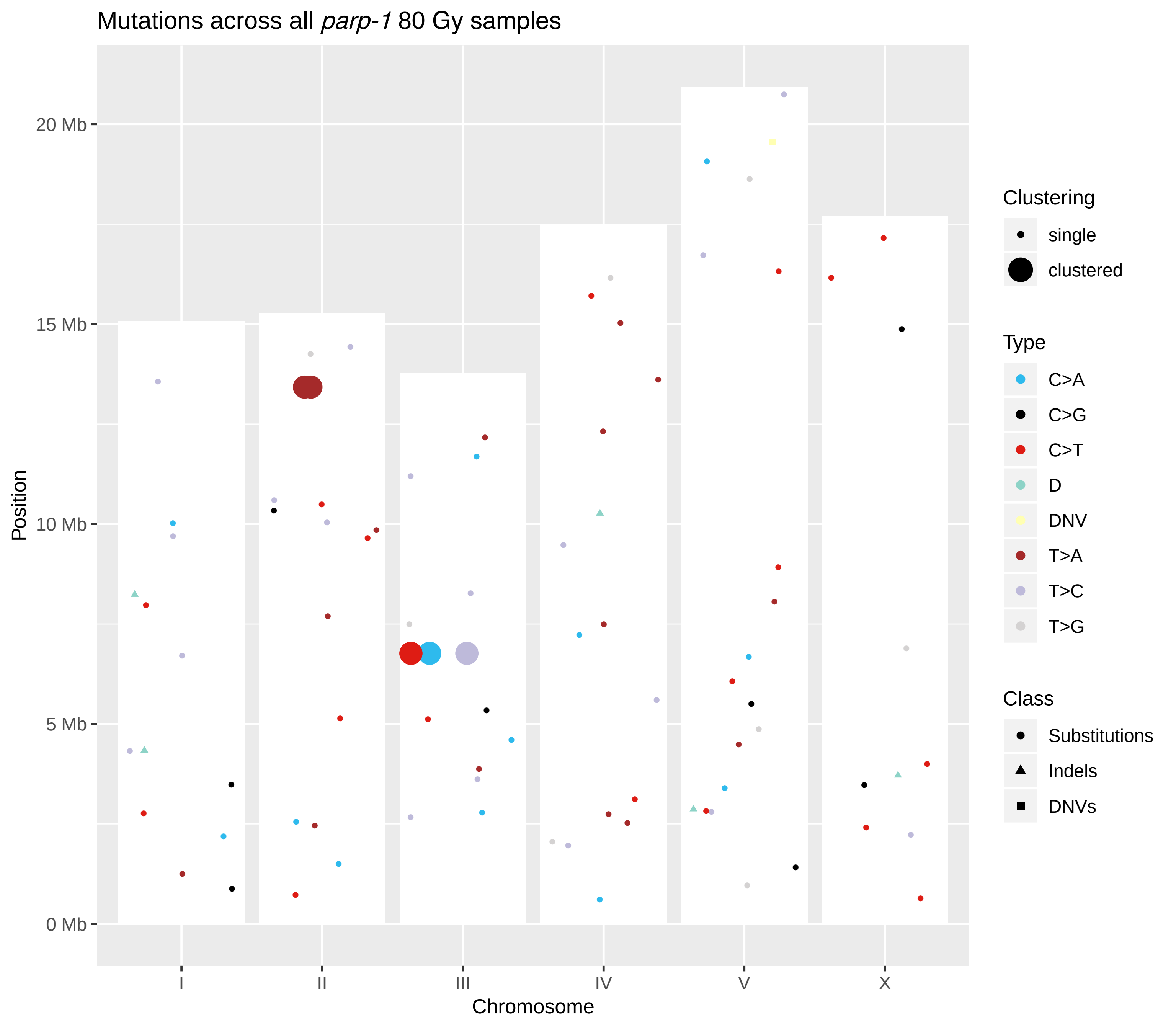

Mutations across all parp-1 80 Gy samples
Position
20 Mb
15 Mb
10 Mb
5 Mb
0 Mb
Chromosome
I
II
III
IV
V
X
Clustering
single
clustered
Type
C>A
C>G
C>T
D
DNV
T>A
T>C
T>G
Class
Substitutions
Indels
DNVs

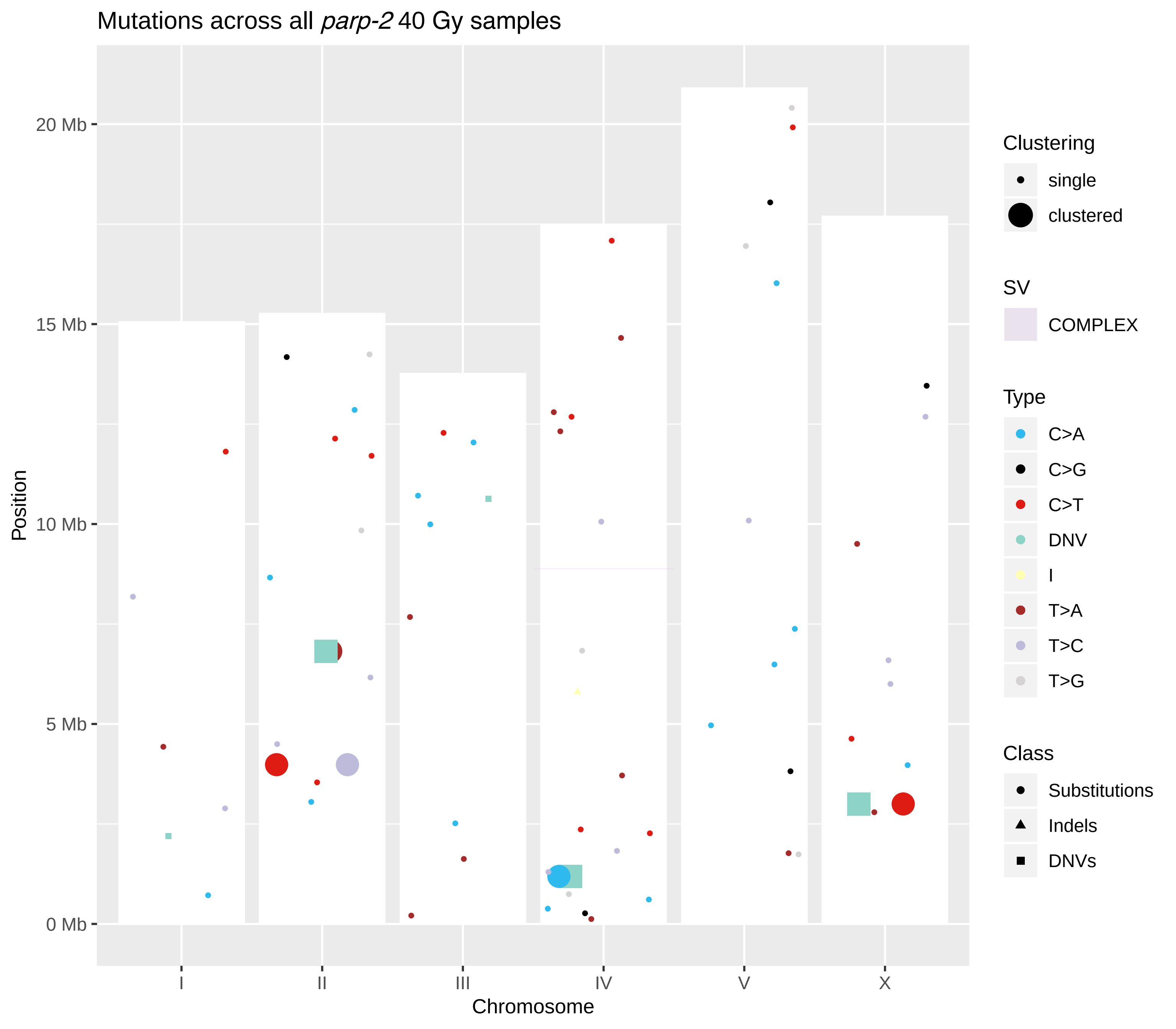

Mutations across all parp-2 40 Gy samples
Position
20 Mb
15 Mb
10 Mb
5 Mb
0 Mb
I
II
III
IV
V
X
Chromosome
Clustering
single
clustered
SV
COMPLEX
Type
C>A
C>G
C>T
DNV
I
T>A
T>C
T>G
Class
Substitutions
Indels
DNVs

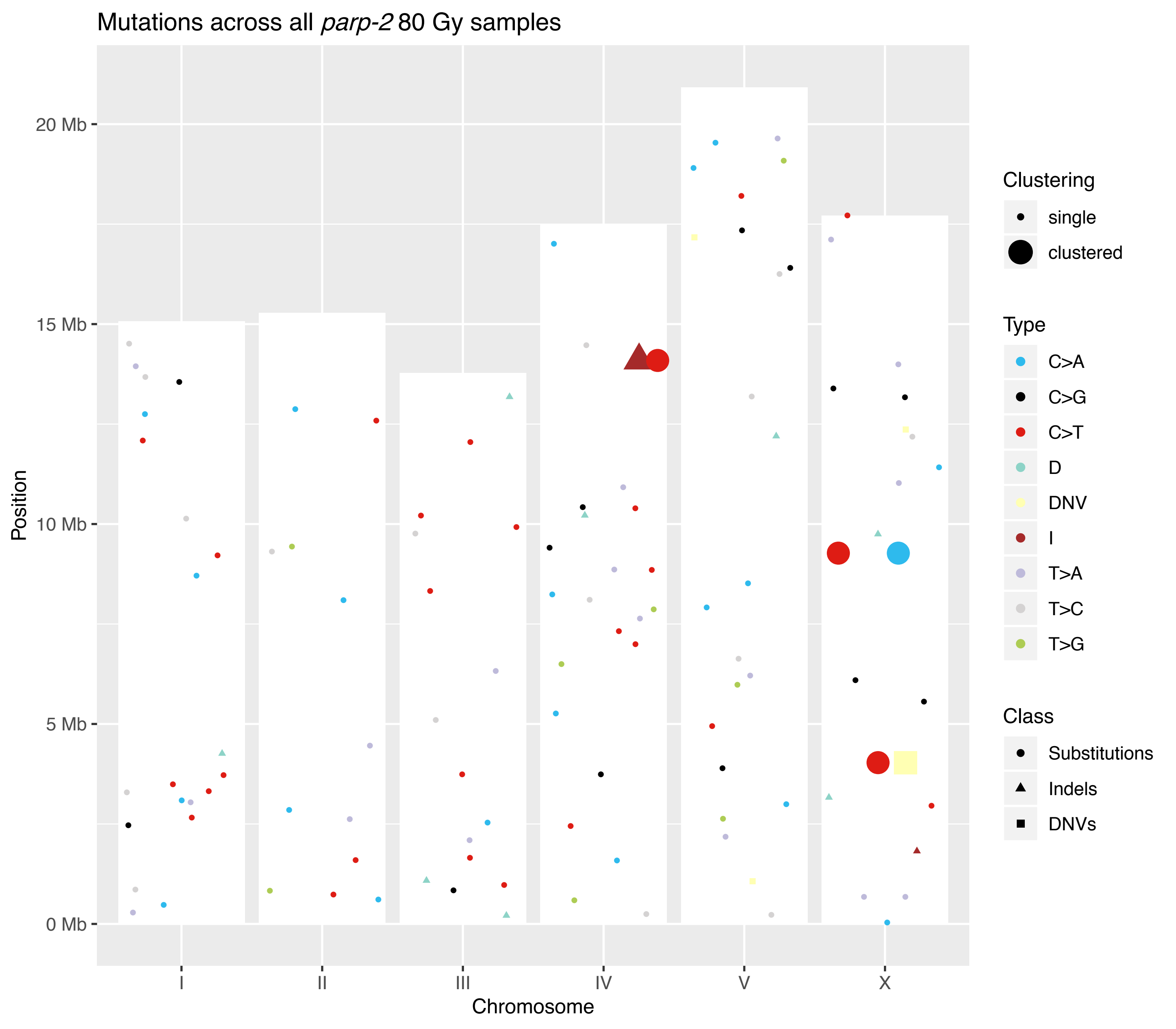
Mutations across all parp-2 80 Gy samples
Position
20 Mb
15 Mb
10 Mb
5 Mb
0 Mb
I
II
III
IV
V
X
Chromosome
Clustering
single
clustered
Type
C>A
C>G
C>T
D
DNV
I
T>A
T>C
T>G
Class
Substitutions
Indels
DNVs

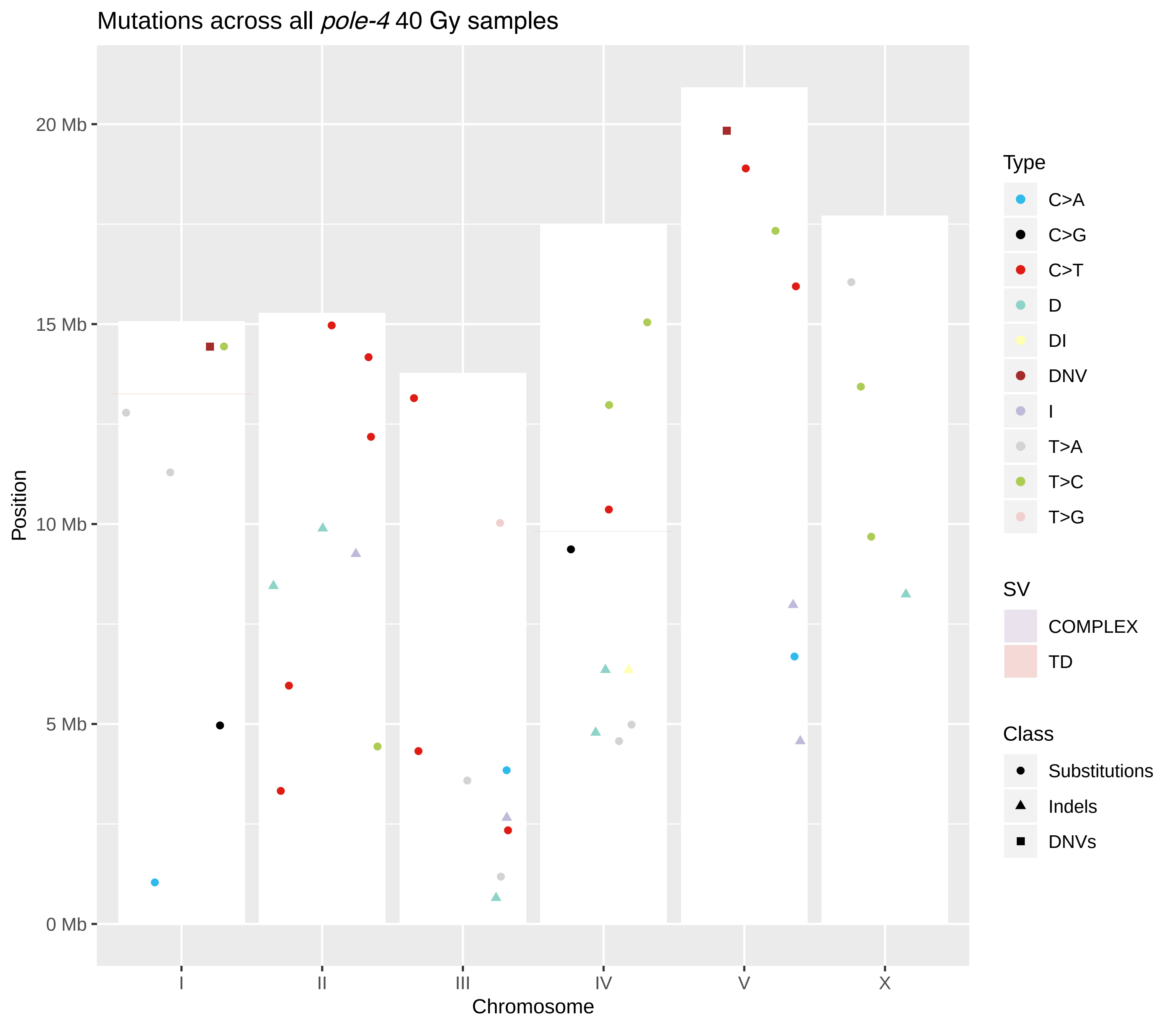

Mutations across all *pole-4* 40 Gy samples
Position
20 Mb
15 Mb
10 Mb
5 Mb
0 Mb
I
II
III
IV
V
X
Chromosome
Type
C>A
C>G
C>T
D
DI
DNV
I
T>A
T>C
T>G
SV
COMPLEX
TD
Class
Substitutions
Indels
DNVs

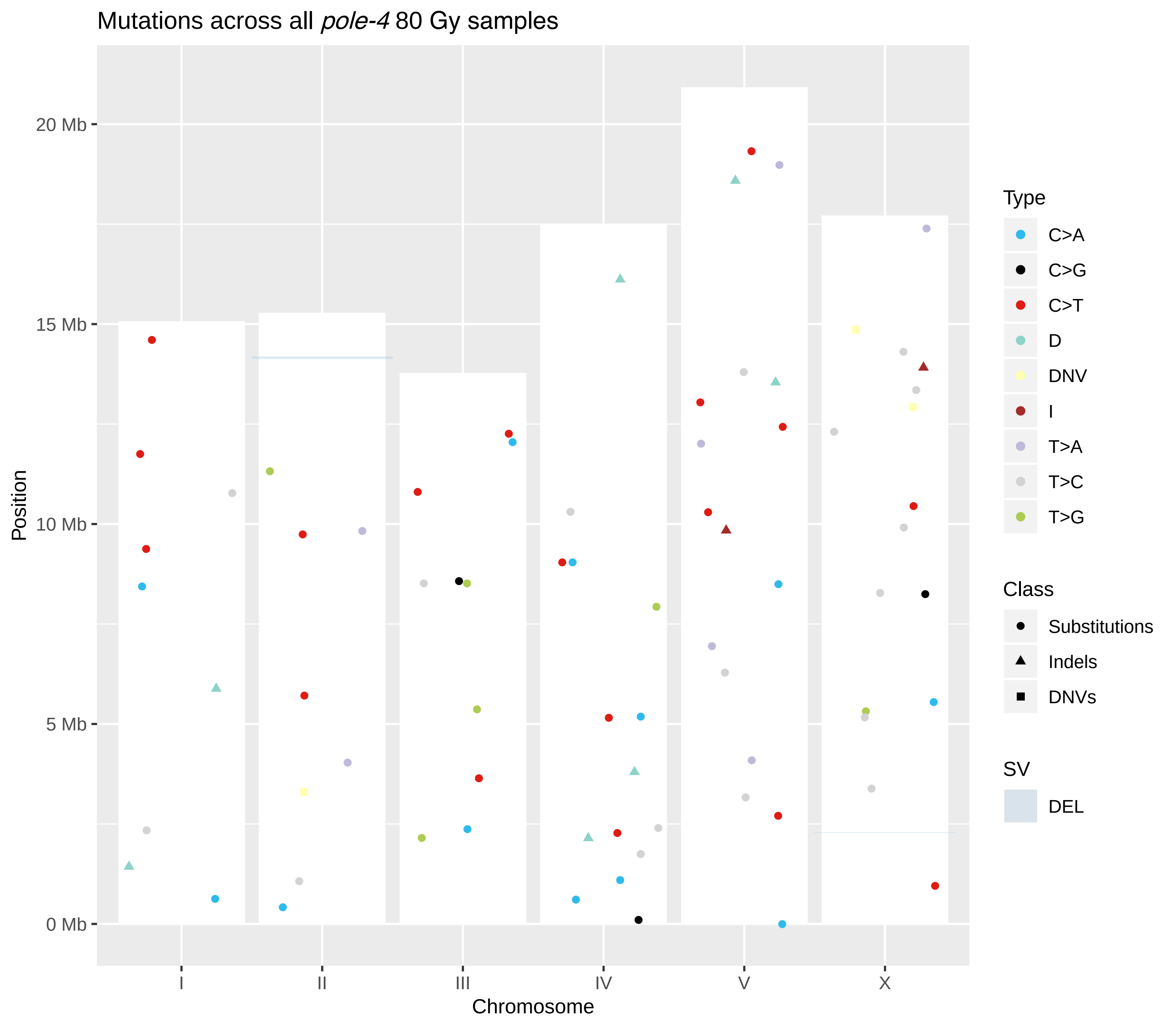
Mutations across all *pole-4* 80 Gy samples
Position
20 Mb
15 Mb
10 Mb
5 Mb
0 Mb
I
II
III
IV
V
X
Chromosome
Type
C>A
C>G
C>T
D
DNV
I
T>A
T>C
T>G
Class
Substitutions
Indels
DNVs
SV
DEL

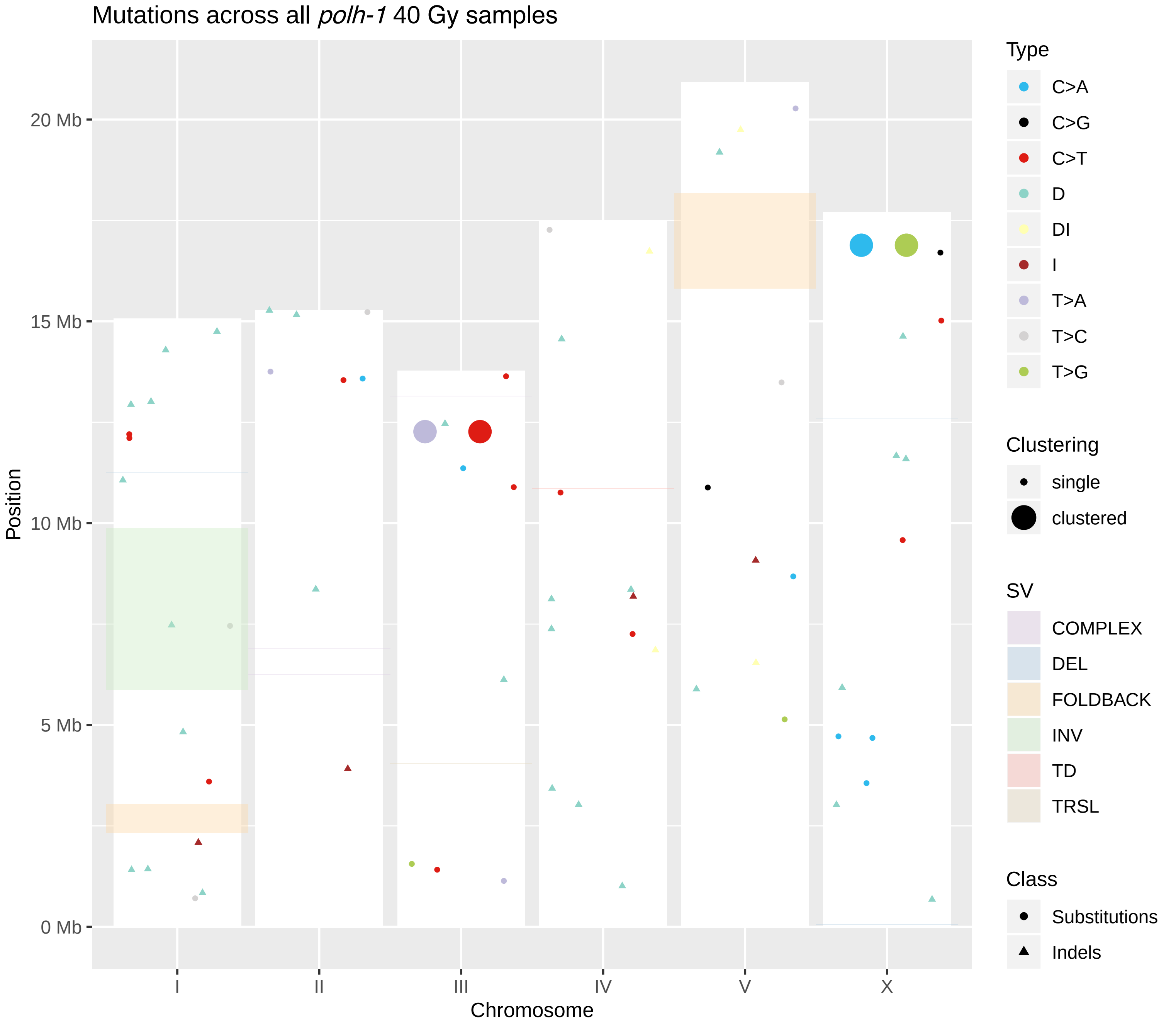
Mutations across all polh-1 40 Gy samples
Position
20 Mb
15 Mb
10 Mb
5 Mb
0 Mb
I
II
III
IV
V
X
Chromosome
Type
C>A
C>G
C>T
D
DI
I
T>A
T>C
T>G
Clustering
single
clustered
SV
COMPLEX
DEL
FOLDBACK
INV
TD
TRSL
Class
Substitutions
Indels

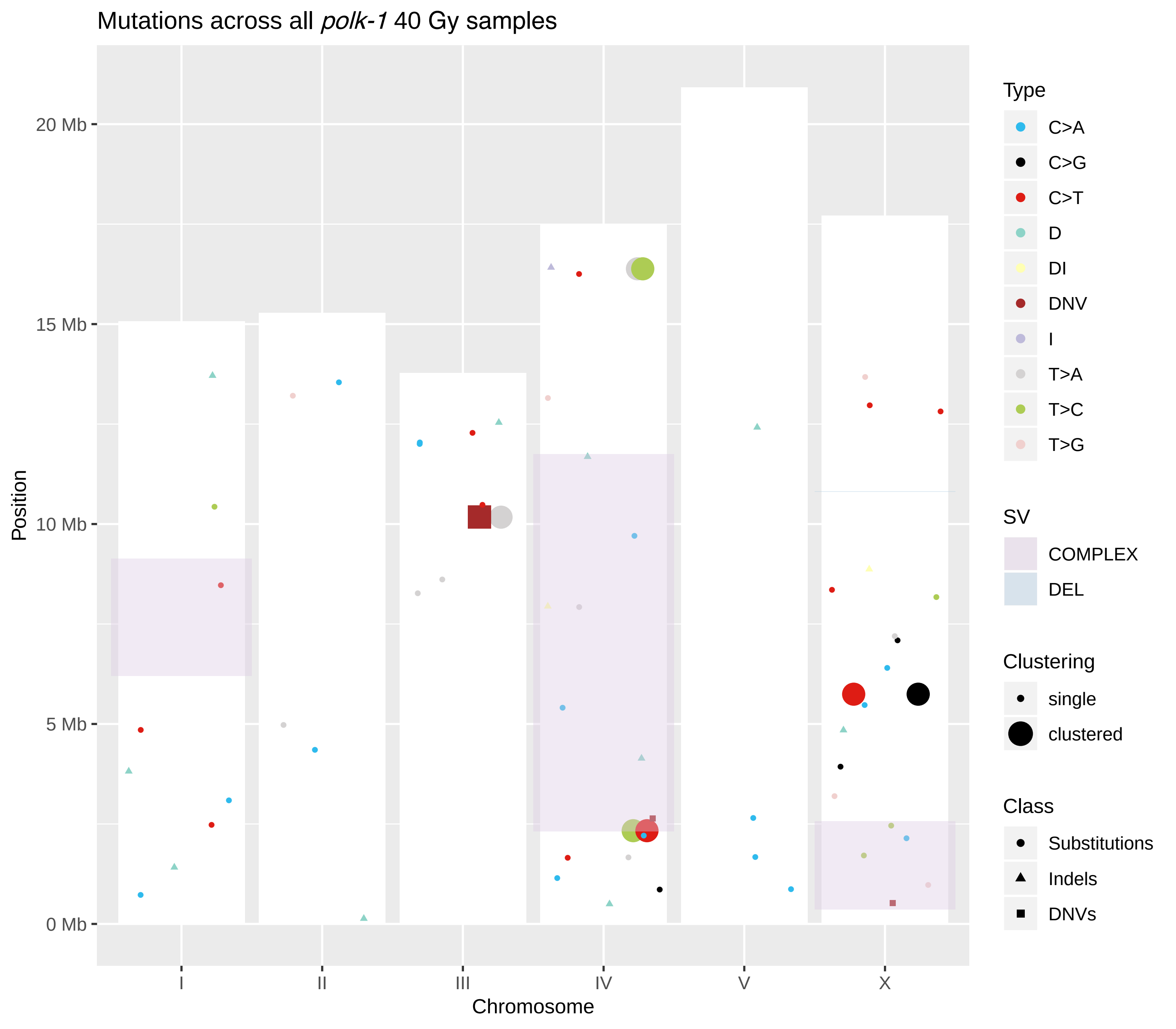

Mutations across all *polk-1* 40 Gy samples
Position
20 Mb
15 Mb
10 Mb
5 Mb
0 Mb
I
II
III
IV
V
X
Chromosome
Type
C>A
C>G
C>T
D
DI
DNV
I
T>A
T>C
T>G
SV
COMPLEX
DEL
Clustering
single
clustered
Class
Substitutions
Indels
DNVs

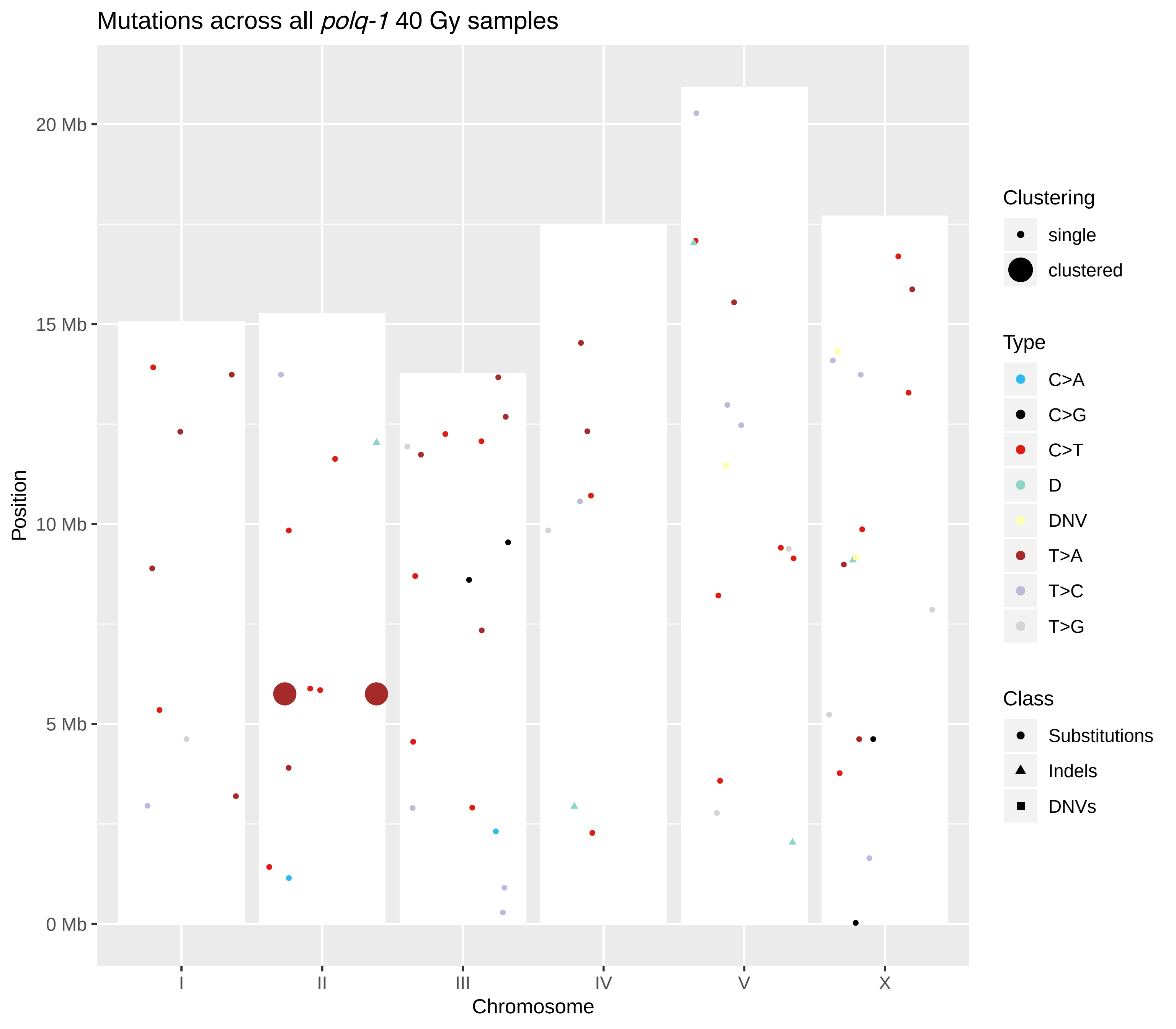
Mutations across all polq-1 40 Gy samples
20 Mb
15 Mb
10 Mb
5 Mb
0 Mb
Position
I
II
III
IV
V
X
Chromosome
Clustering
single
clustered
Type
C>A
C>G
C>T
D
DNV
T>A
T>C
T>G
Class
Substitutions
Indels
DNVs

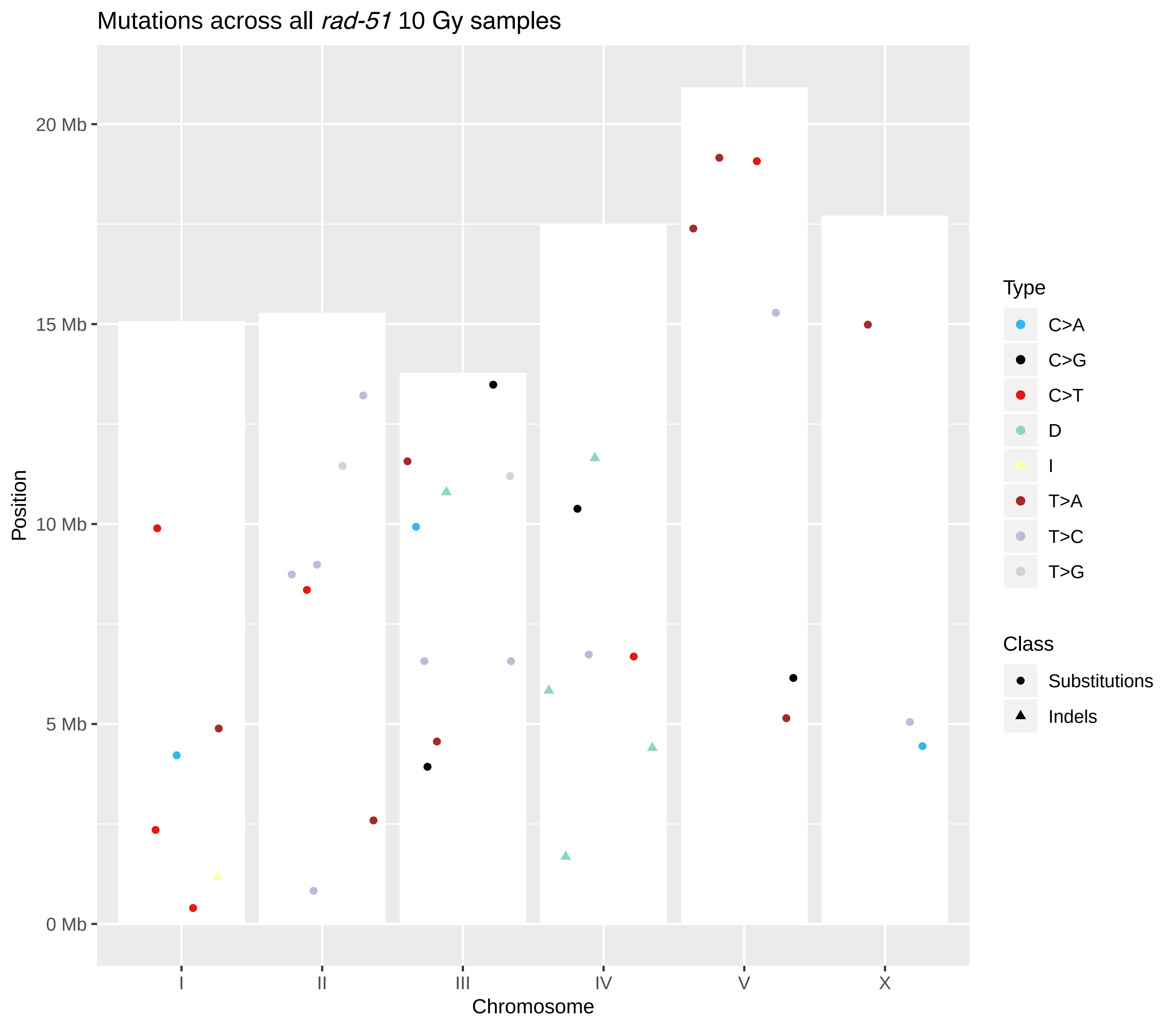

Mutations across all rad-51 10 Gy samples
Position
20 Mb
15 Mb
10 Mb
5 Mb
0 Mb
I
II
III
IV
V
X
Chromosome
Type
C>A
C>G
C>T
D
I
T>A
T>C
T>G
Class
Substitutions
Indels

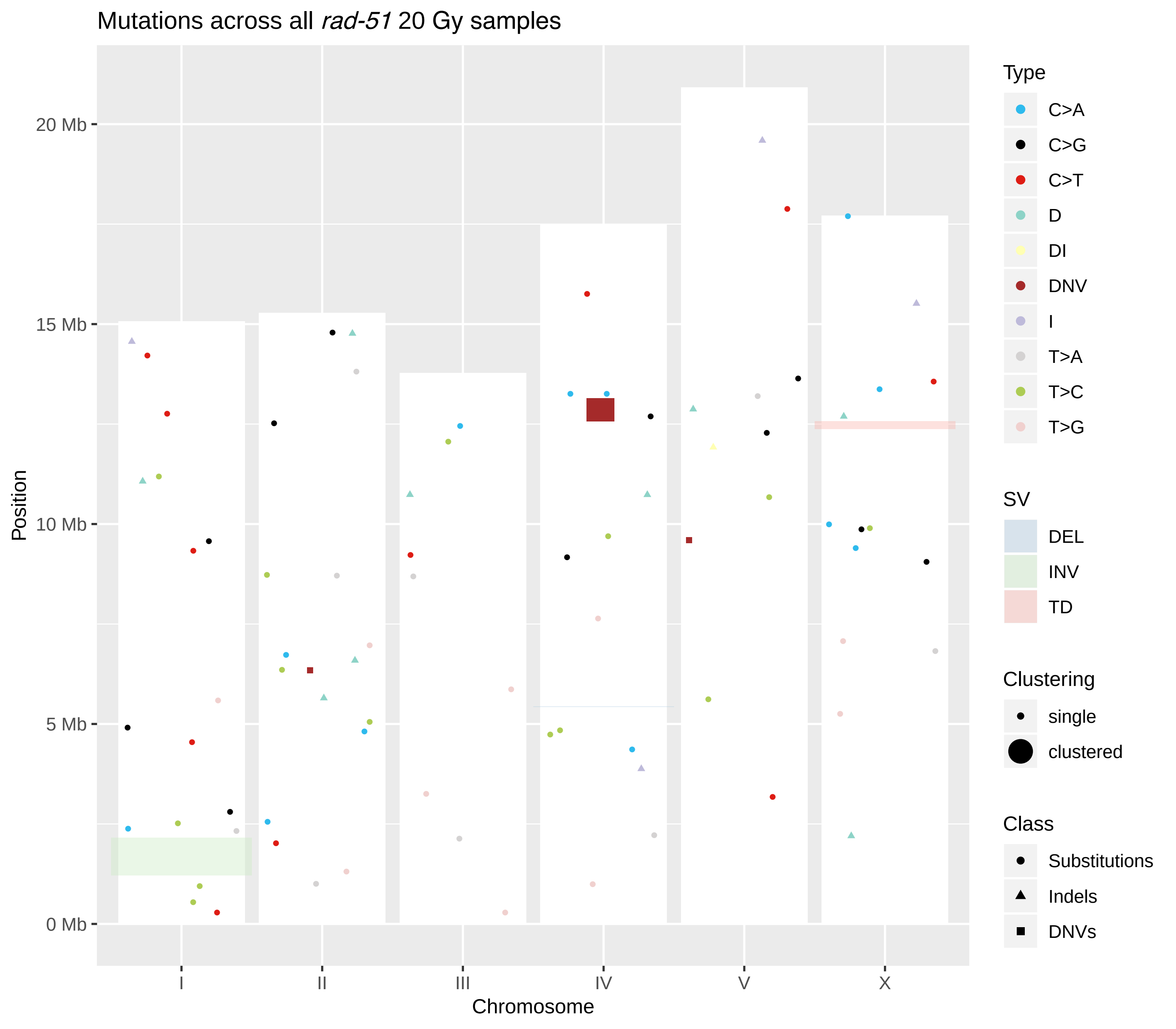
Mutations across all rad-51 20 Gy samples
Position
20 Mb
15 Mb
10 Mb
5 Mb
0 Mb
I
II
III
IV
V
X
Chromosome
Type
C>A
C>G
C>T
D
DI
DNV
I
T>A
T>C
T>G
SV
DEL
INV
TD
Clustering
single
clustered
Class
Substitutions
Indels
DNVs

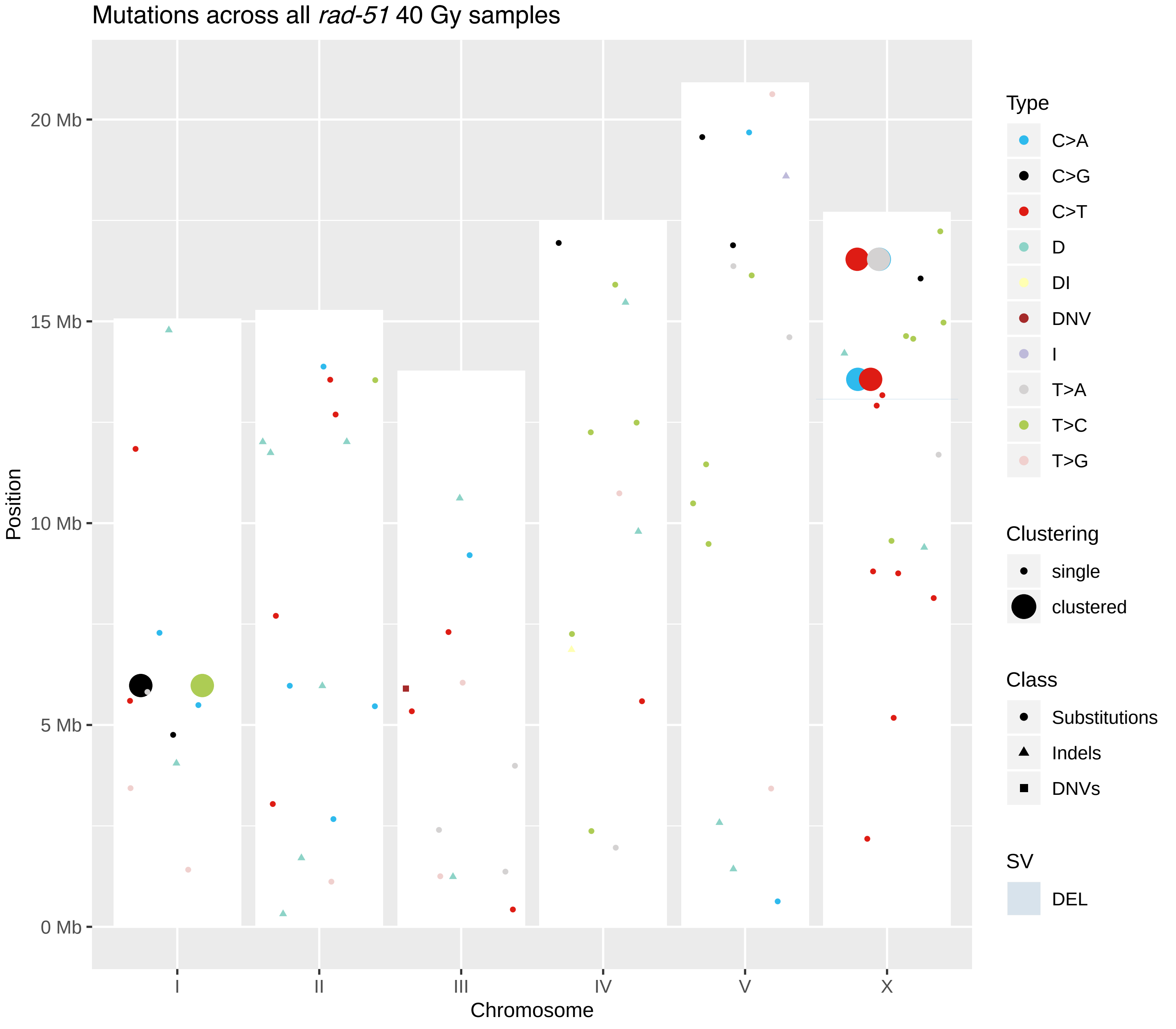
Mutations across all *rad-51* 40 Gy samples
Position
20 Mb
15 Mb
10 Mb
5 Mb
0 Mb
Chromosome
I
II
III
IV
V
X
Type
C>A
C>G
C>T
D
DI
DNV
I
T>A
T>C
T>G
Clustering
single
clustered
Class
Substitutions
Indels
DNVs
SV
DEL

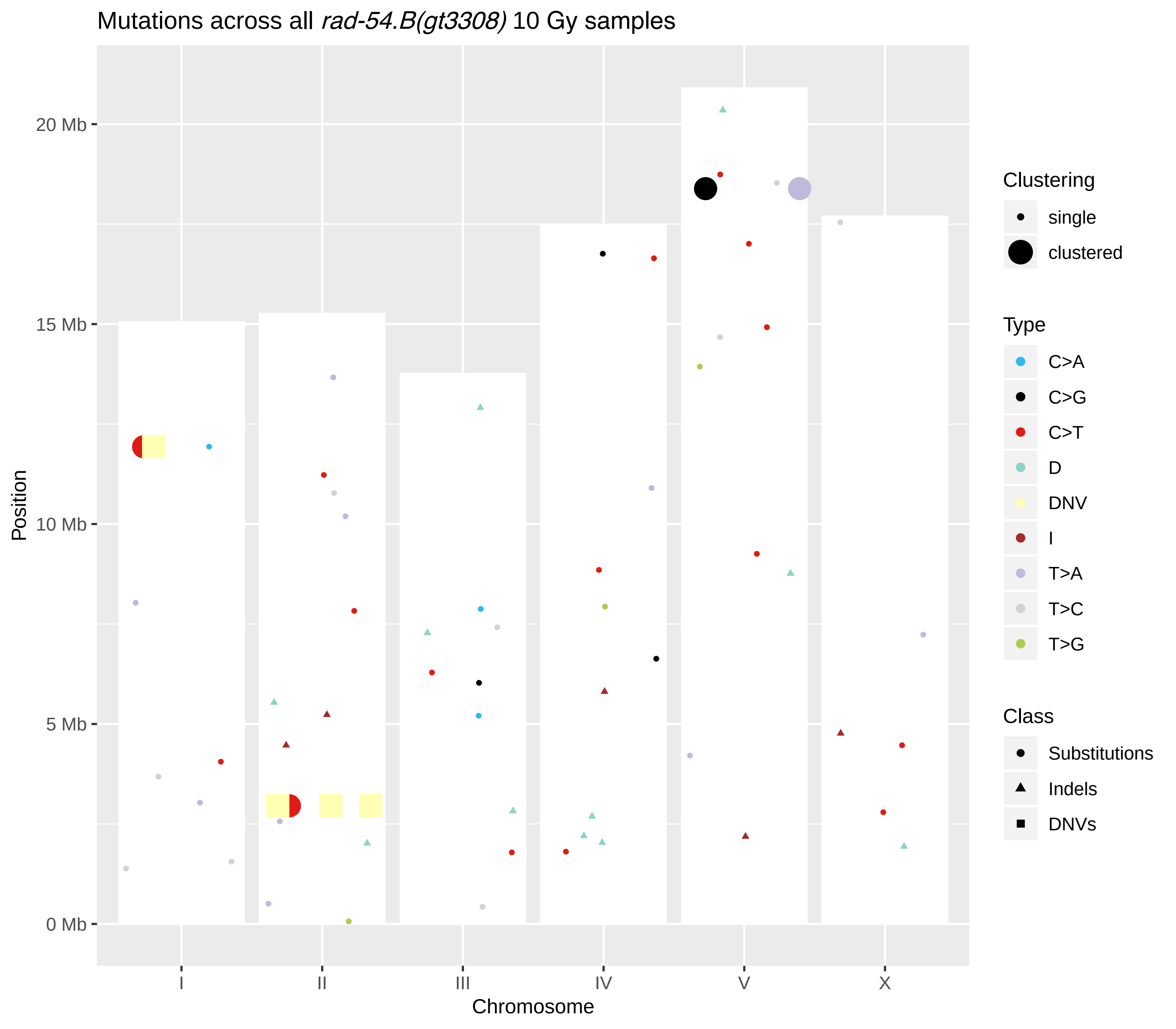

Mutations across all *rad-54.B(gt3308)* 10 Gy samples
Position
20 Mb
15 Mb
10 Mb
5 Mb
0 Mb
Chromosome
I
II
III
IV
V
X
Clustering
single
clustered
Type
C>A
C>G
C>T
D
DNV
I
T>A
T>C
T>G
Class
Substitutions
Indels
DNVs

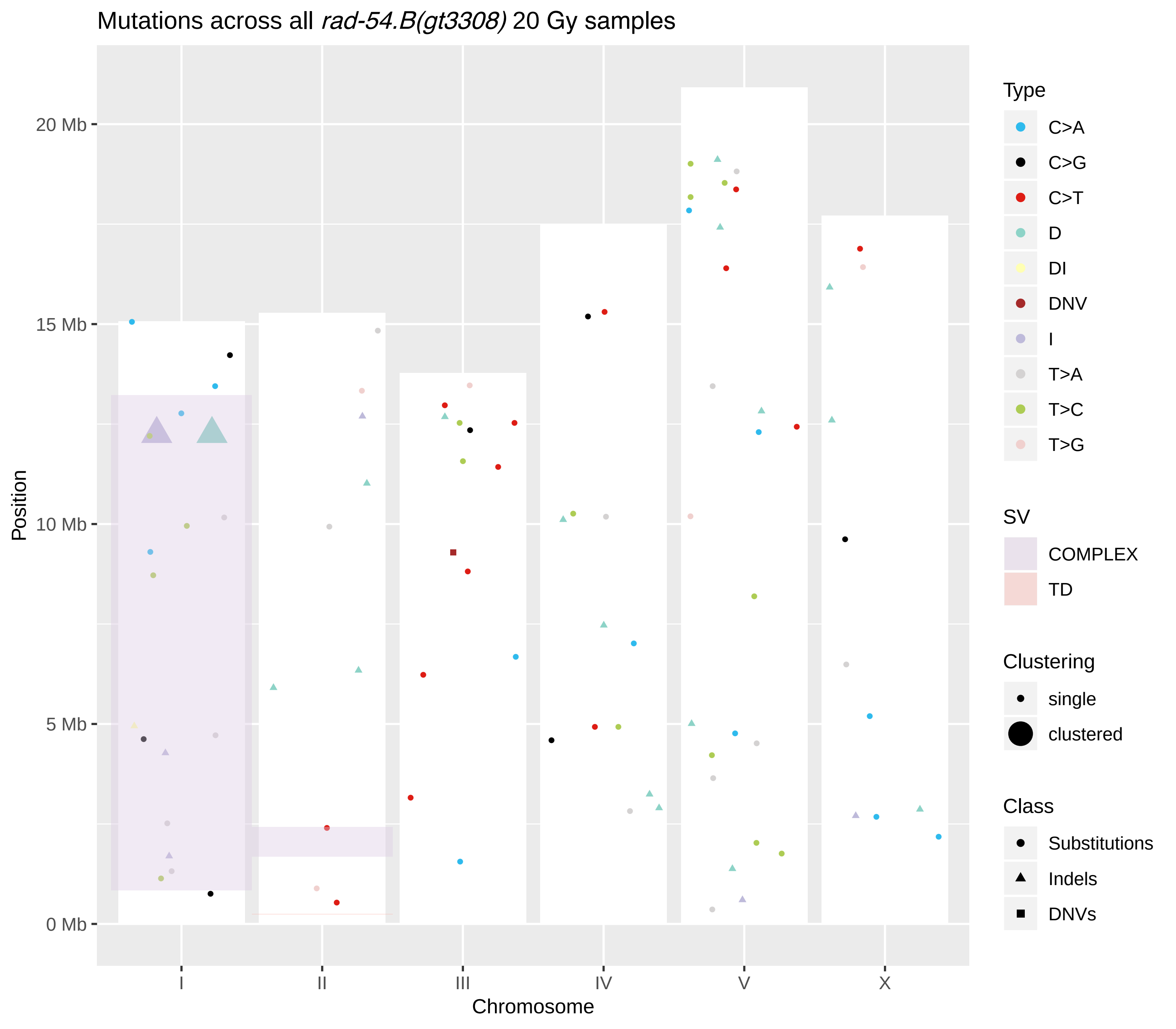

Mutations across all rad-54.B(gt3308) 20 Gy samples
20 Mb
15 Mb
10 Mb
5 Mb
0 Mb
Position
I
II
III
IV
V
X
Chromosome
Type
C>A
C>G
C>T
D
DI
DNV
I
T>A
T>C
T>G
SV
COMPLEX
TD
Clustering
single
clustered
Class
Substitutions
Indels
DNVs

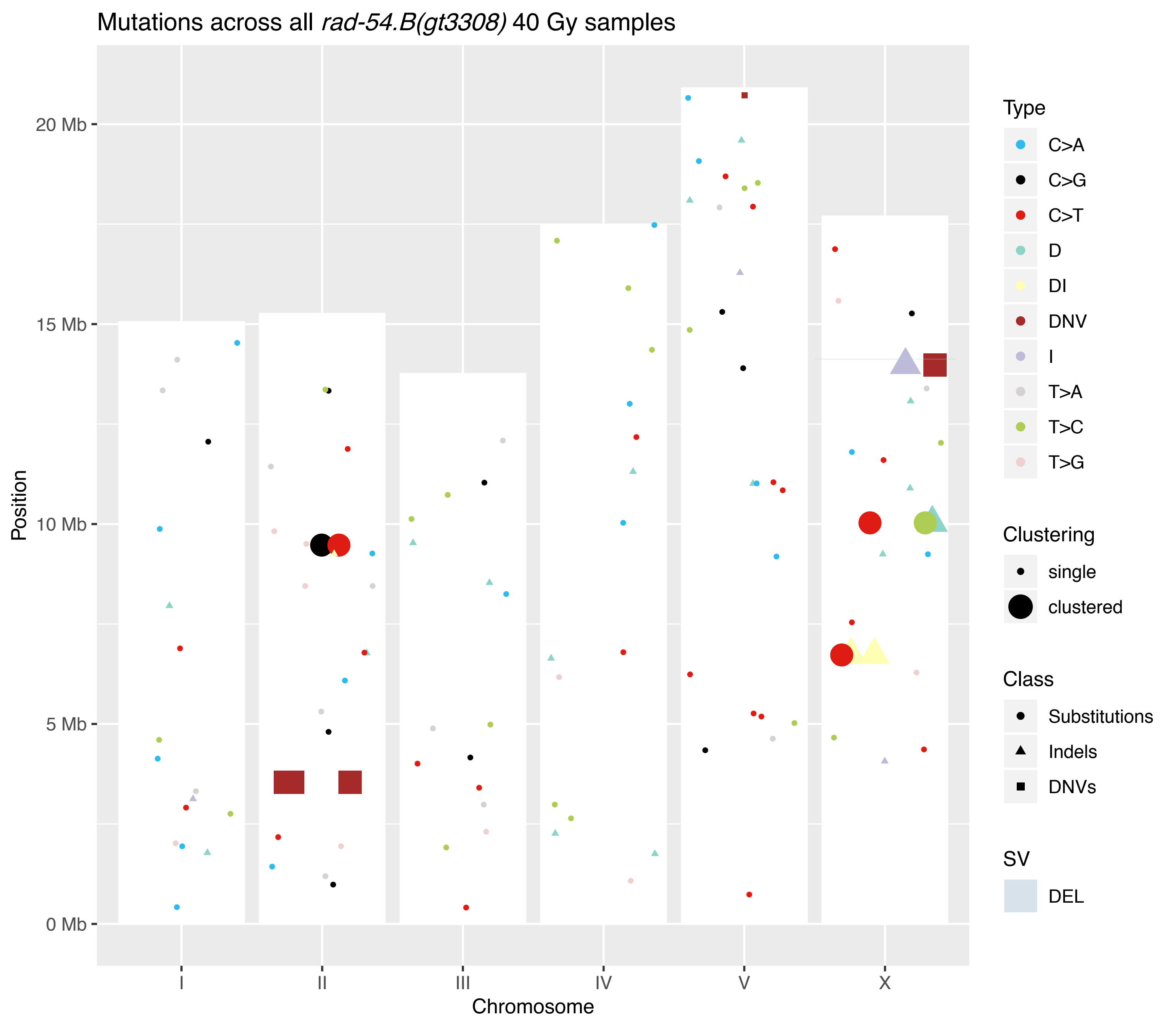

Mutations across all rad-54.B(gt3308) 40 Gy samples
Position
20 Mb
15 Mb
10 Mb
5 Mb
0 Mb
I
II
III
IV
V
X
Chromosome
Type
C>A
C>G
C>T
D
DI
DNV
I
T>A
T>C
T>G
Clustering
single
clustered
Class
Substitutions
Indels
DNVs
SV
DEL

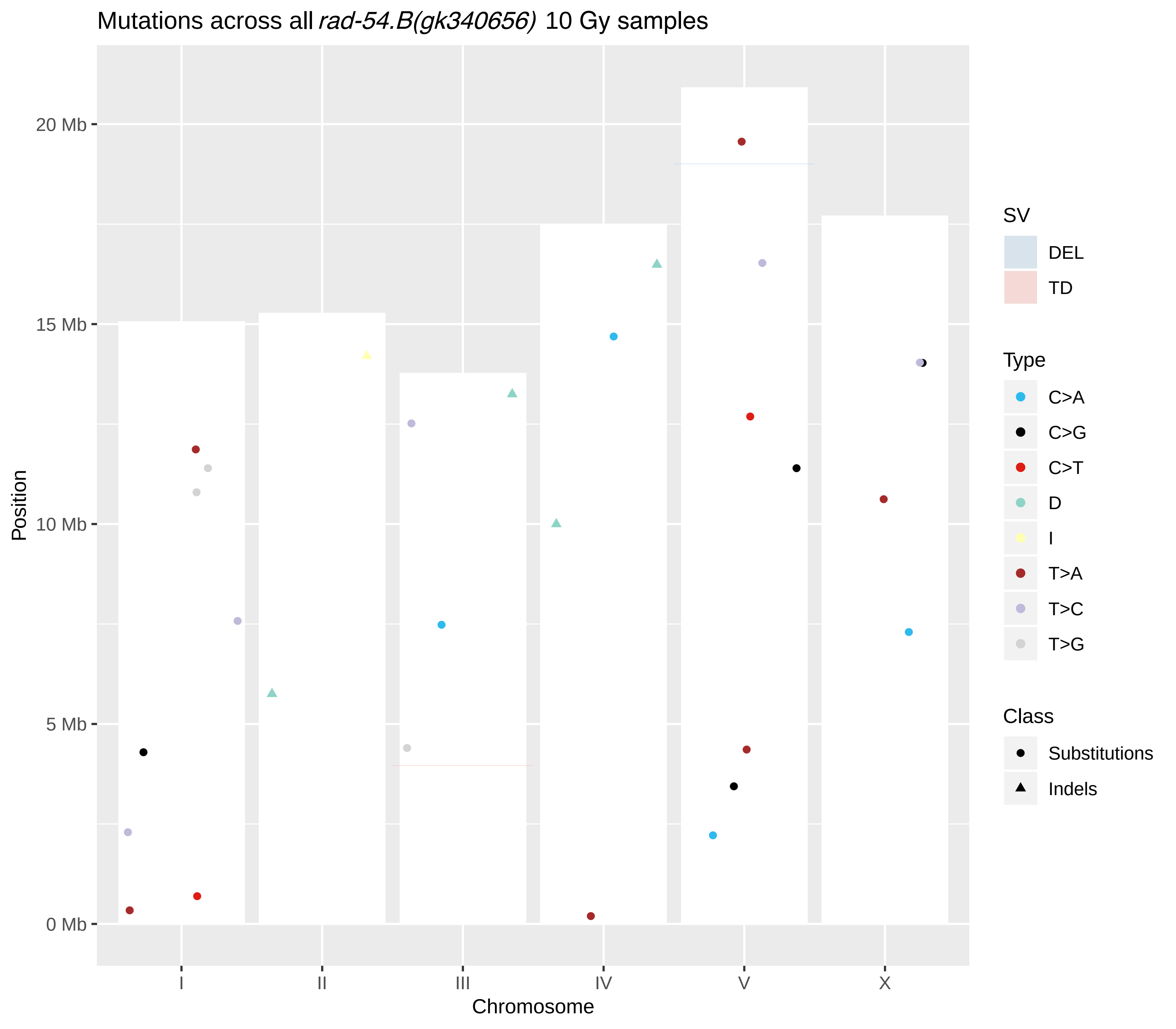

Mutations across all *rad-54.B(gk340656)* 10 Gy samples
20 Mb
15 Mb
10 Mb
5 Mb
0 Mb
Position
I
II
III
IV
V
X
Chromosome
SV
DEL
TD
Type
C>A
C>G
C>T
D
I
T>A
T>C
T>G
Class
Substitutions
Indels

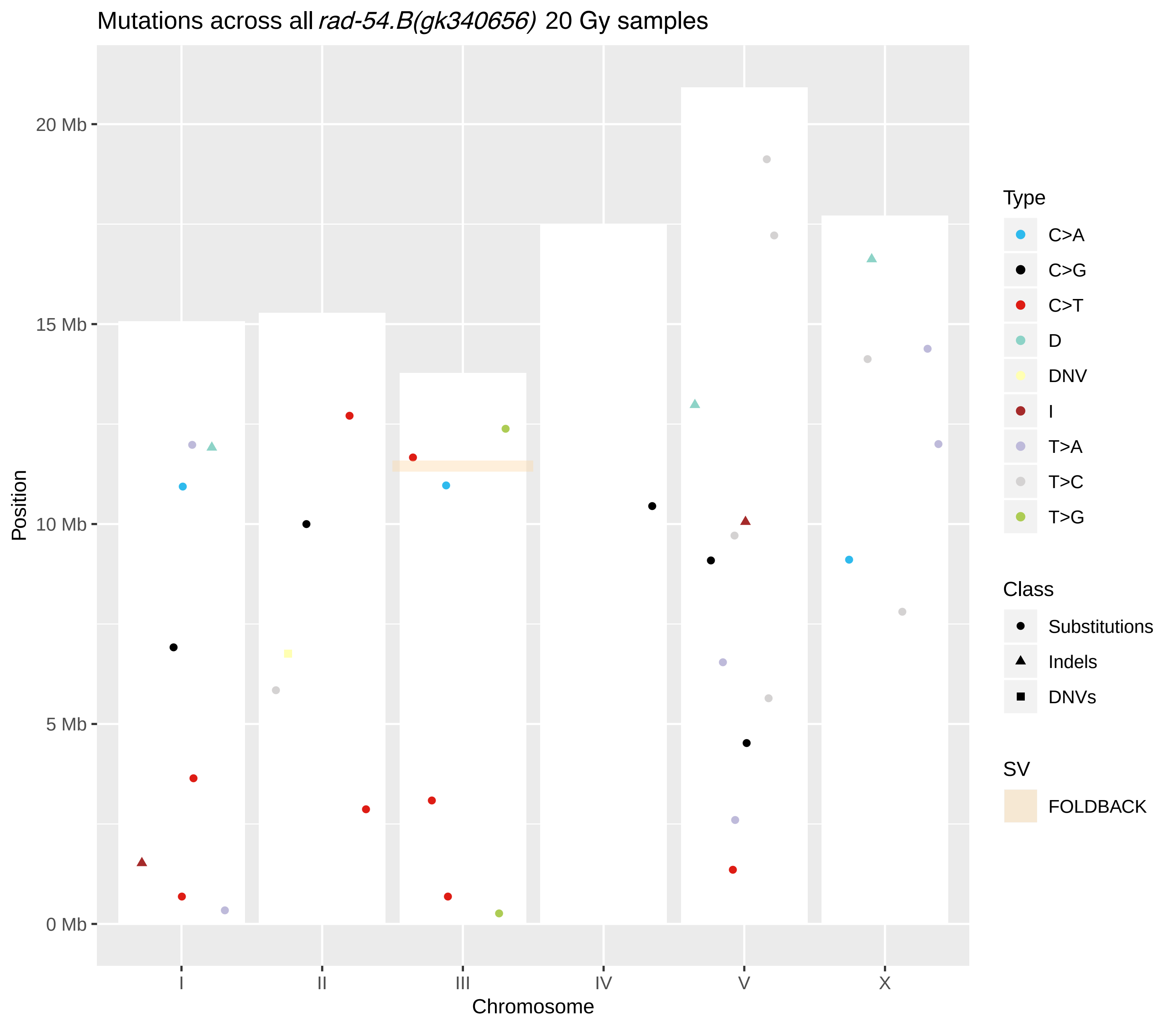
Mutations across all rad-54.B(gk340656) 20 Gy samples
20 Mb
15 Mb
10 Mb
5 Mb
0 Mb
Position
I
II
III
IV
V
X
Chromosome
Type
C>A
C>G
C>T
D
DNV
I
T>A
T>C
T>G
Class
Substitutions
Indels
DNVs
SV
FOLDBACK

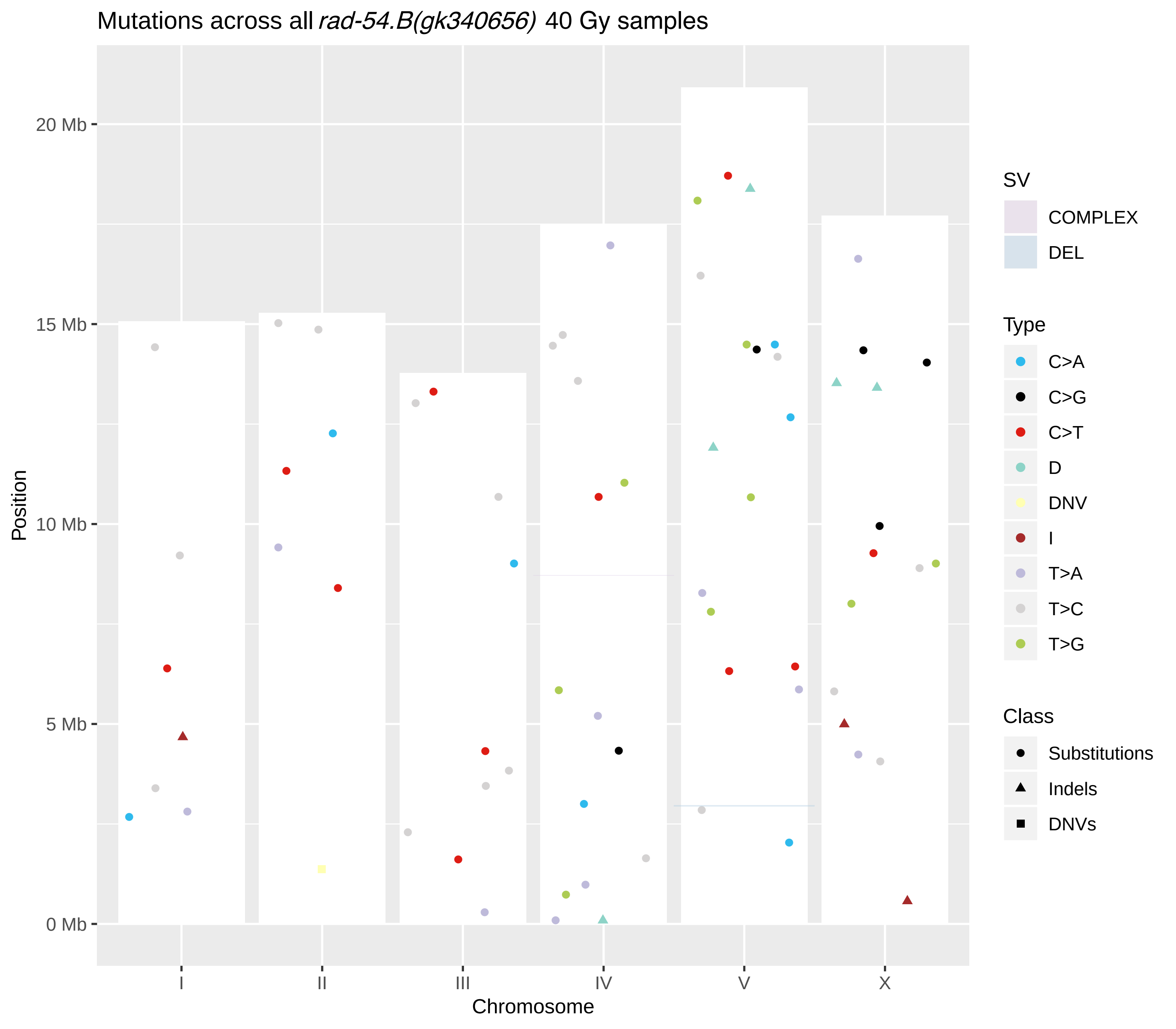
Mutations across all rad-54.B(gk340656) 40 Gy samples
20 Mb
15 Mb
10 Mb
5 Mb
0 Mb
Position
I
II
III
IV
V
X
Chromosome
SV
COMPLEX
DEL
Type
C>A
C>G
C>T
D
DNV
I
T>A
T>C
T>G
Class
Substitutions
Indels
DNVs

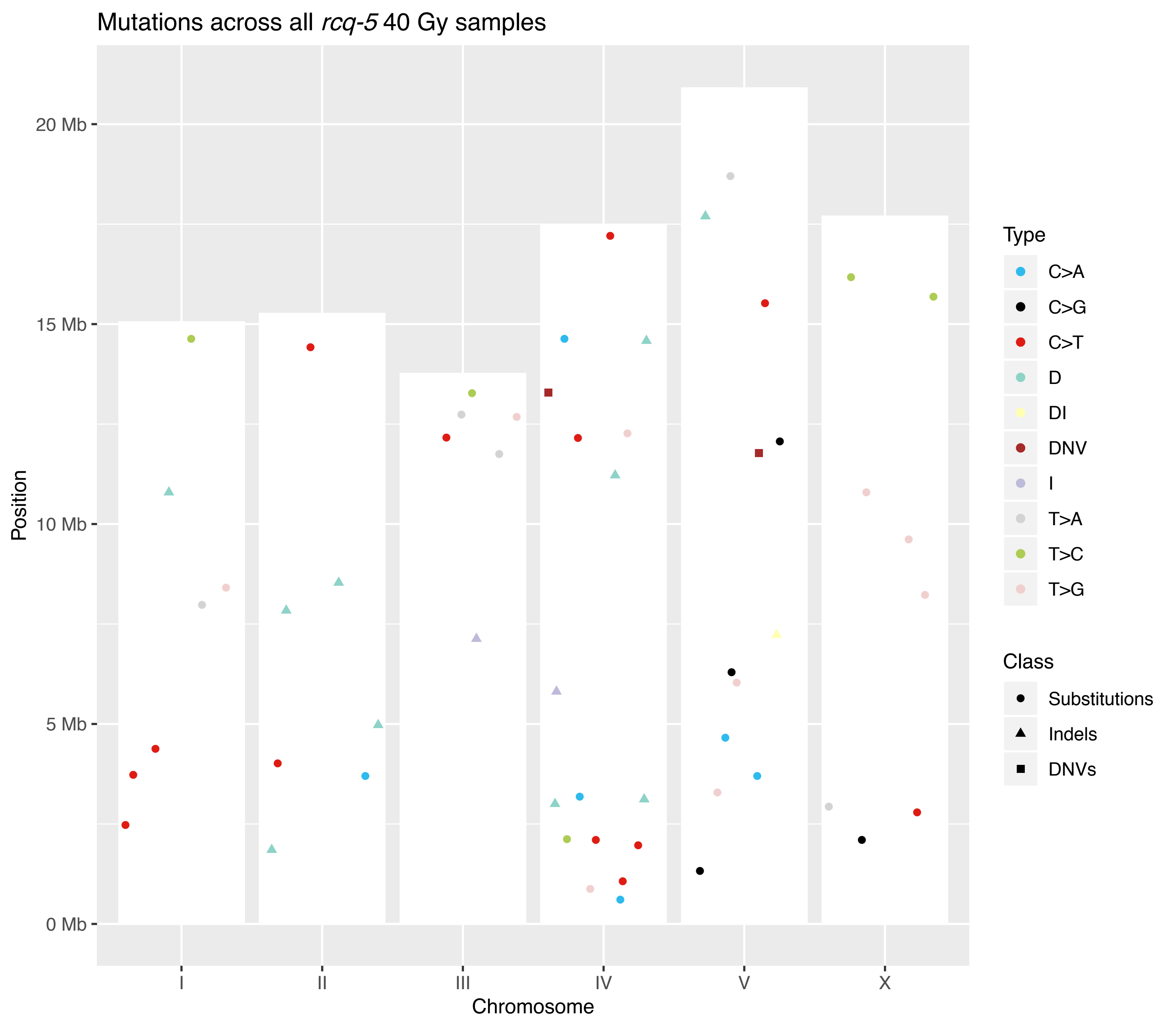
Mutations across all rcq-5 40 Gy samples
Position
20 Mb
15 Mb
10 Mb
5 Mb
0 Mb
I
II
III
IV
V
X
Chromosome
Type
C>A
C>G
C>T
D
DI
DNV
I
T>A
T>C
T>G
Class
Substitutions
Indels
DNVs

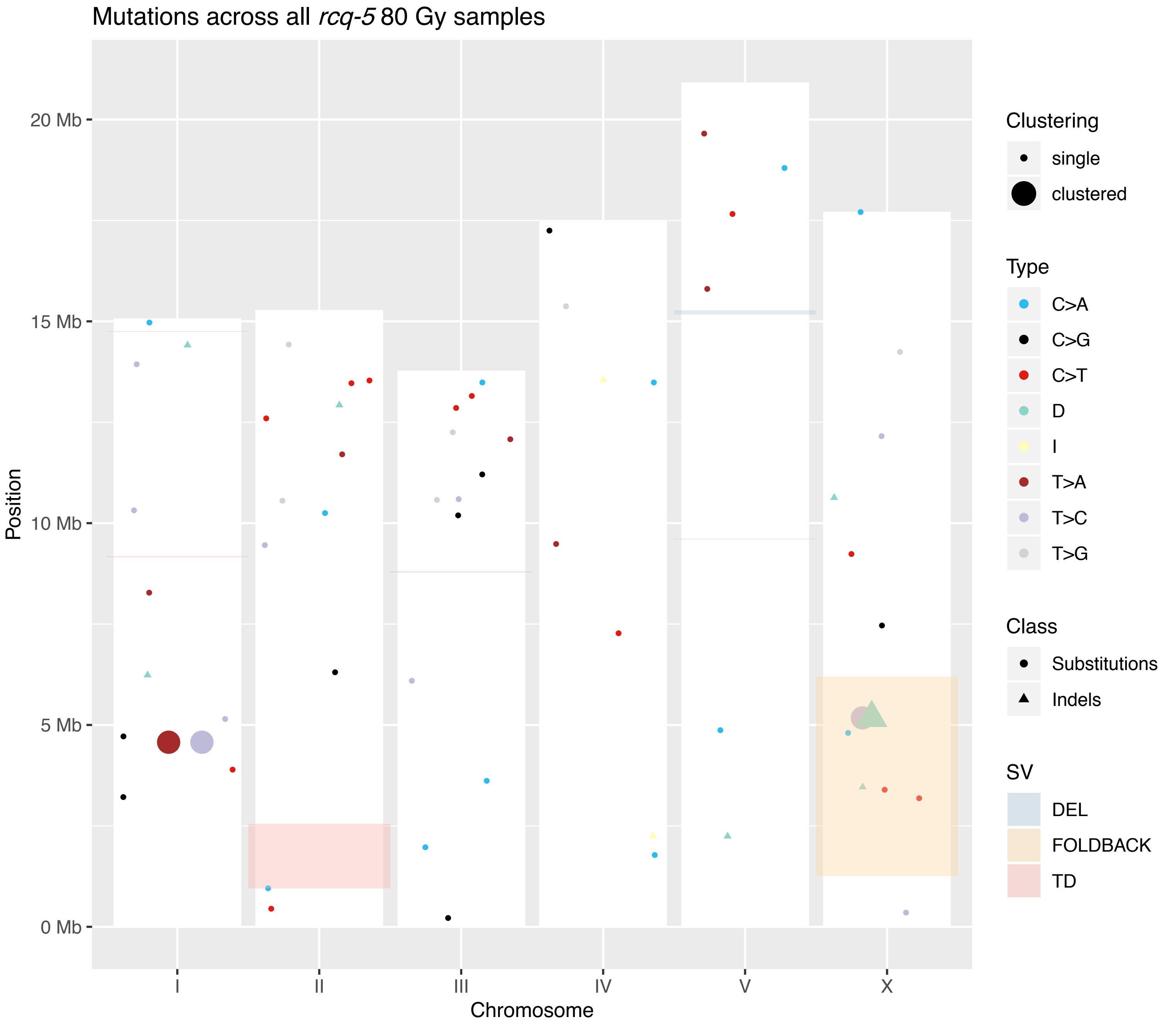

Mutations across all *rcq-5* 80 Gy samples
Position
20 Mb
15 Mb
10 Mb
5 Mb
0 Mb
I
II
III
IV
V
X
Chromosome
Clustering
single
clustered
Type
C>A
C>G
C>T
D
I
T>A
T>C
T>G
Class
Substitutions
Indels
SV
DEL
FOLDBACK
TD

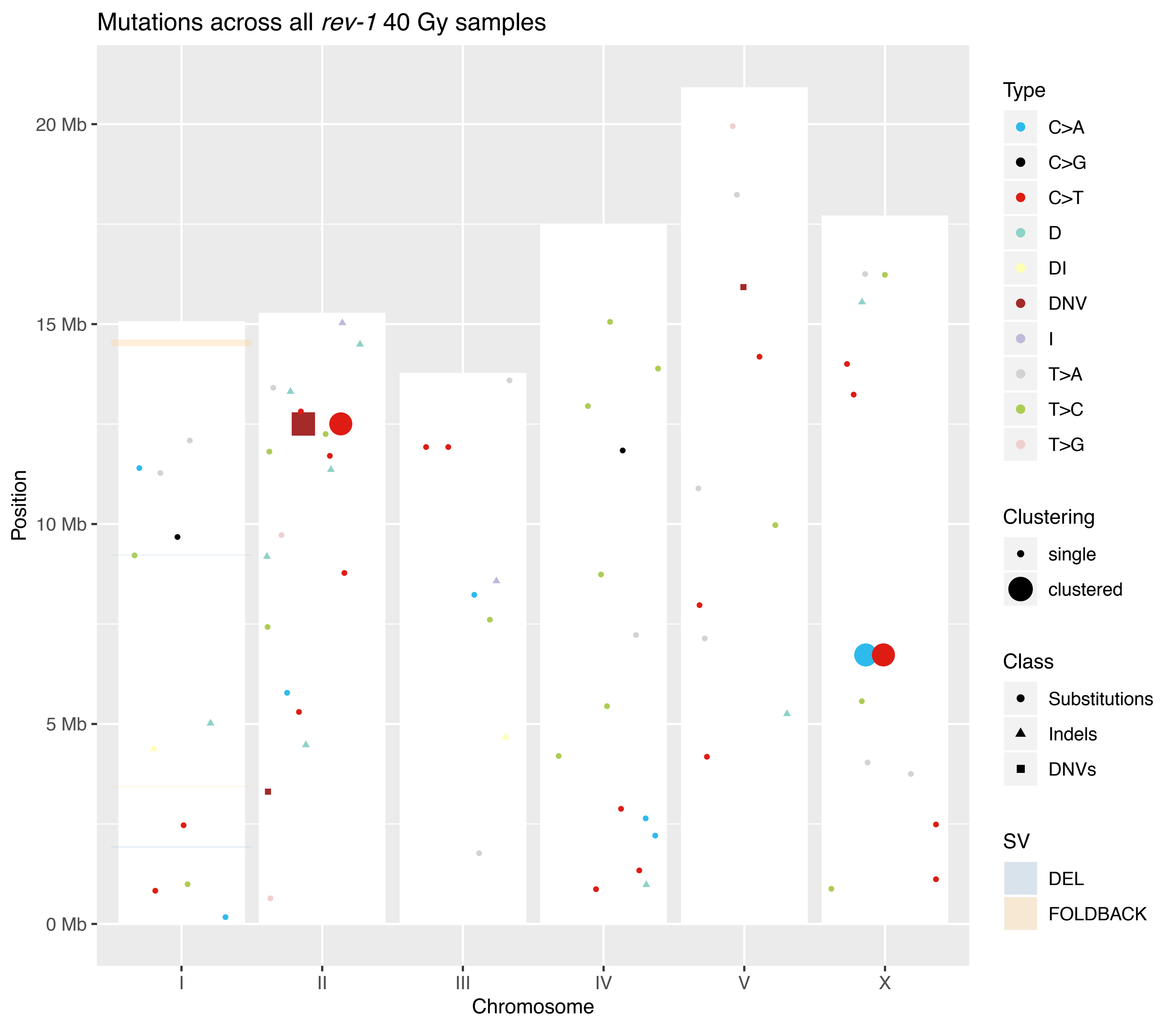
Mutations across all rev-1 40 Gy samples
Position
20 Mb
15 Mb
10 Mb
5 Mb
0 Mb
I
II
III
IV
V
X
Chromosome
Type
C>A
C>G
C>T
D
DI
DNV
I
T>A
T>C
T>G
Clustering
single
clustered
Class
Substitutions
Indels
DNVs
SV
DEL
FOLDBACK

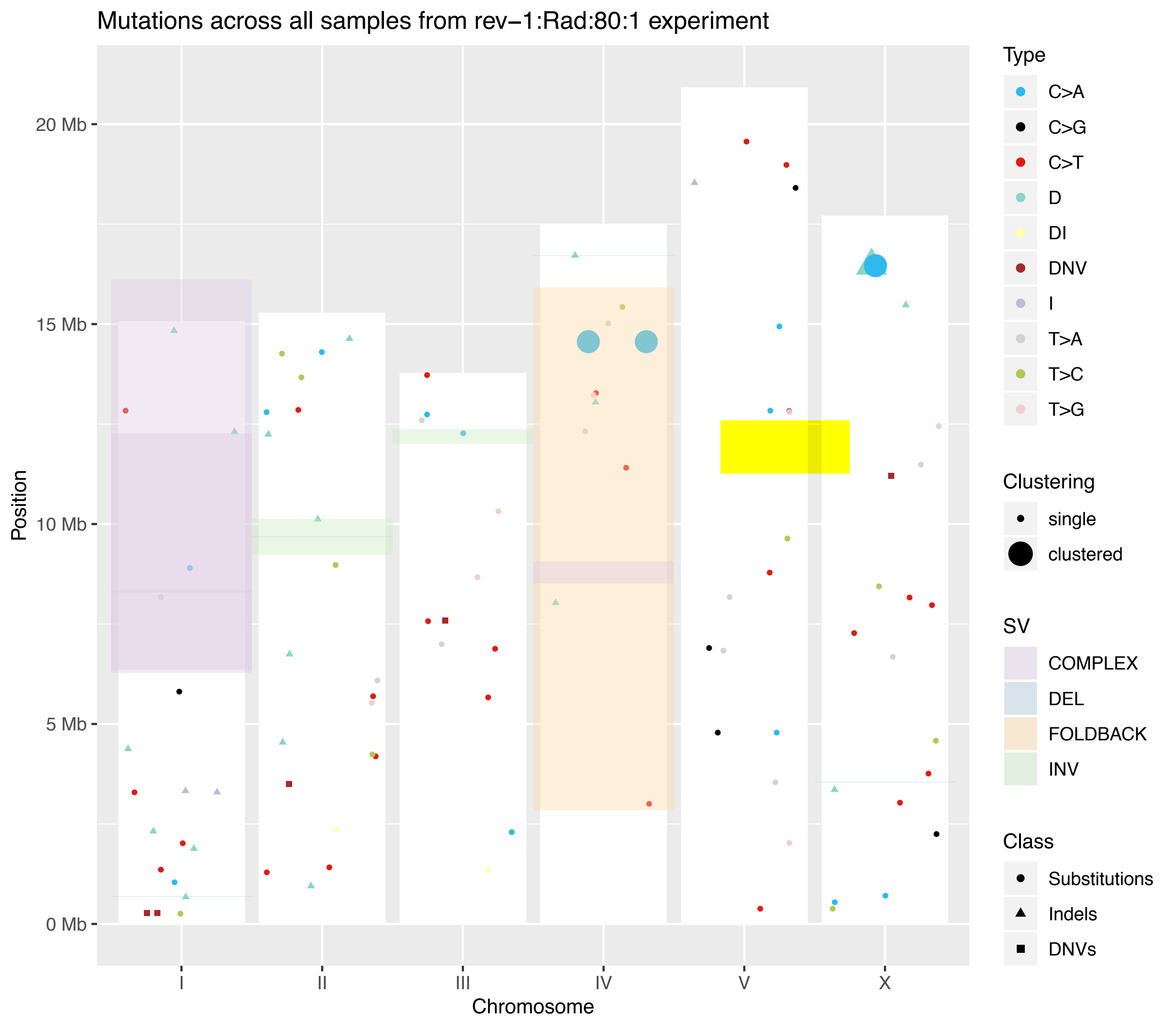

Mutations across all samples from rev−1:Rad:80:1 experiment
Position
20 Mb
15 Mb
10 Mb
5 Mb
0 Mb
Chromosome
I
II
III
IV
V
X
Type
C>A
C>G
C>T
D
DI
DNV
I
T>A
T>C
T>G
Clustering
single
clustered
SV
COMPLEX
DEL
FOLDBACK
INV
Class
Substitutions
Indels
DNVs

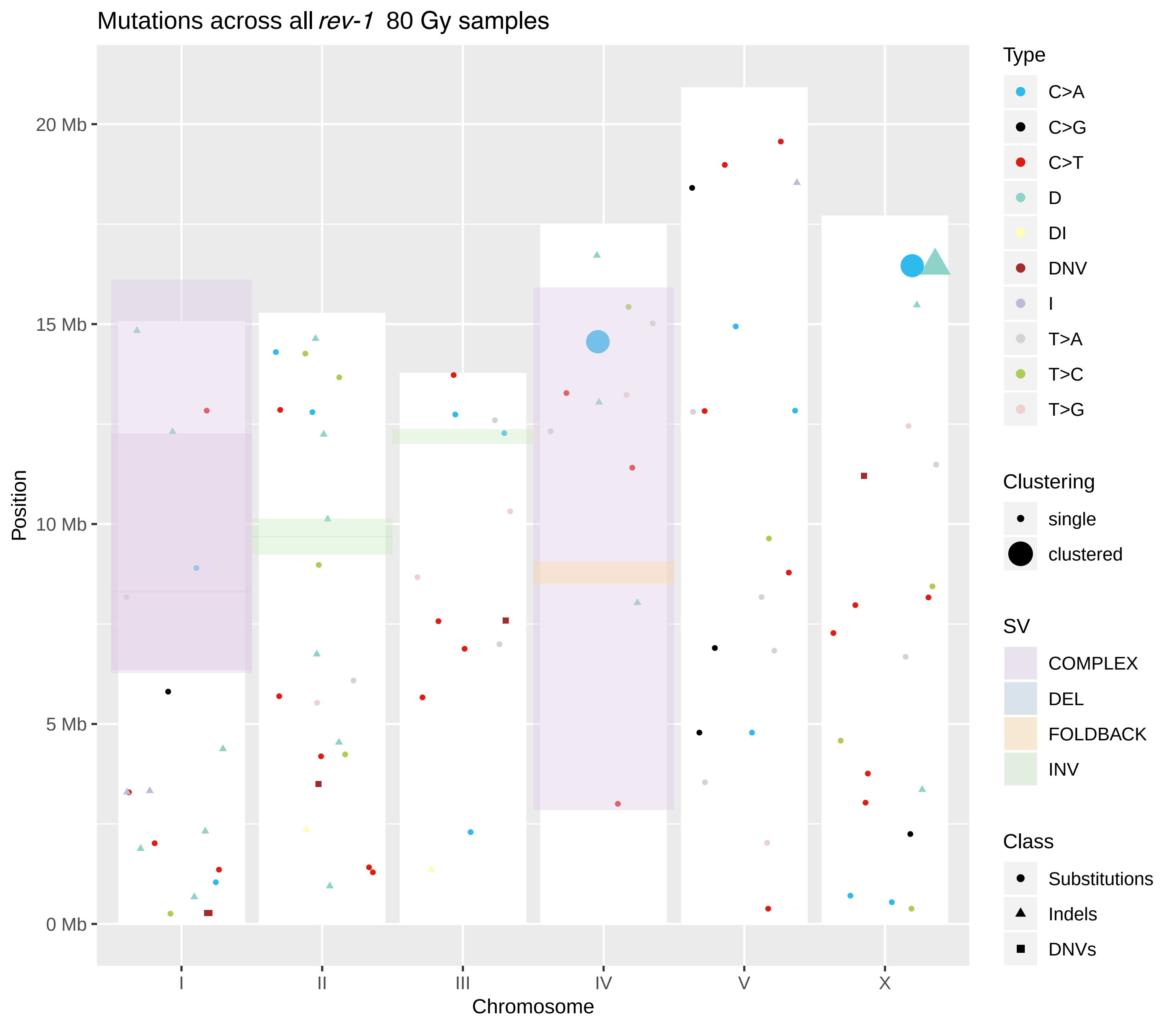

Mutations across all rev-1 80 Gy samples
Position
20 Mb
15 Mb
10 Mb
5 Mb
0 Mb
I
II
III
IV
V
X
Chromosome
Type
C>A
C>G
C>T
D
DI
DNV
I
T>A
T>C
T>G
Clustering
single
clustered
SV
COMPLEX
DEL
FOLDBACK
INV
Class
Substitutions
Indels
DNVs

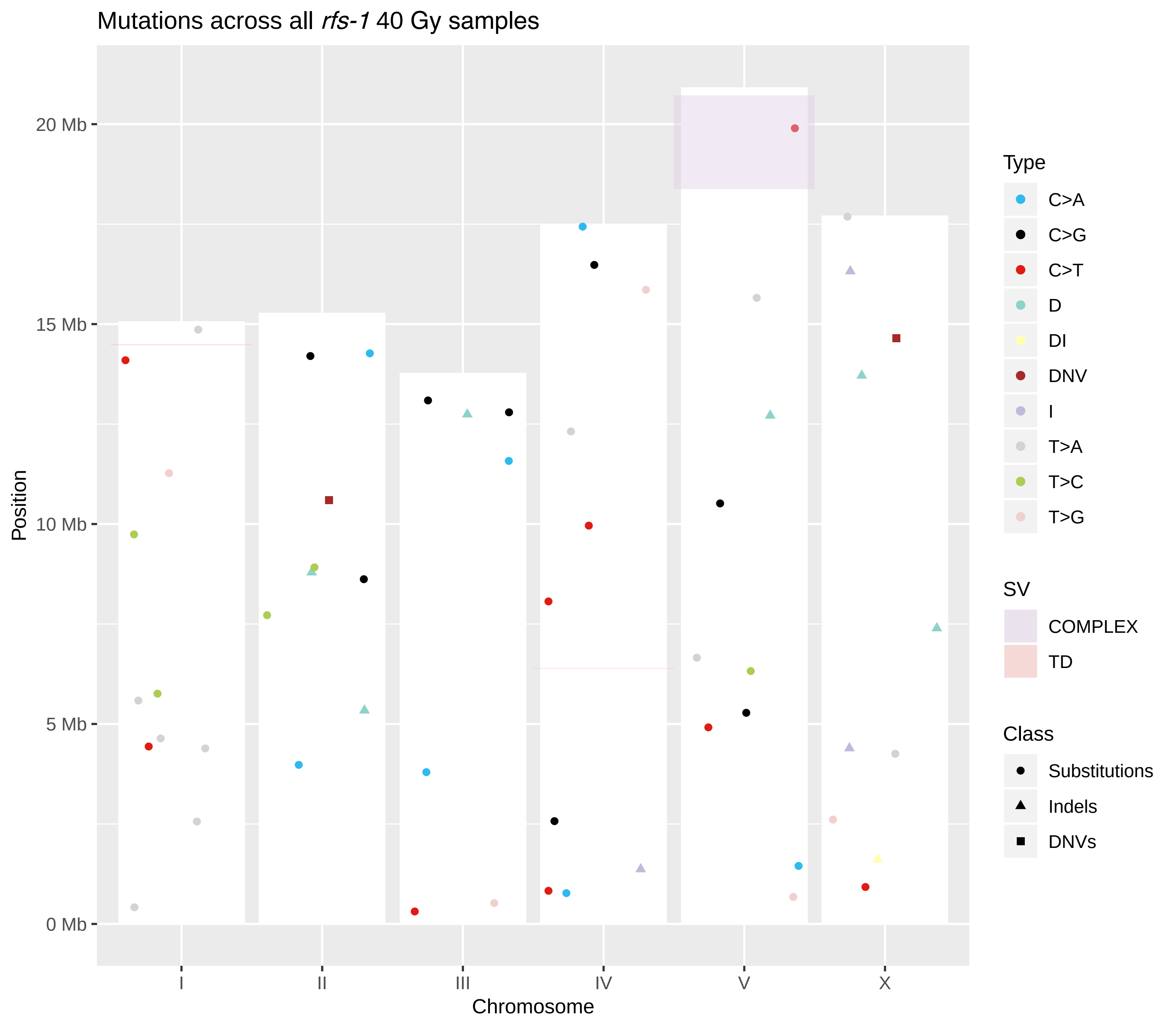

Mutations across all *rfs-1* 40 Gy samples
20 Mb
15 Mb
10 Mb
5 Mb
0 Mb
Position
I
II
III
IV
V
X
Chromosome
Type
C>A
C>G
C>T
D
DI
DNV
I
T>A
T>C
T>G
SV
COMPLEX
TD
Class
Substitutions
Indels
DNVs

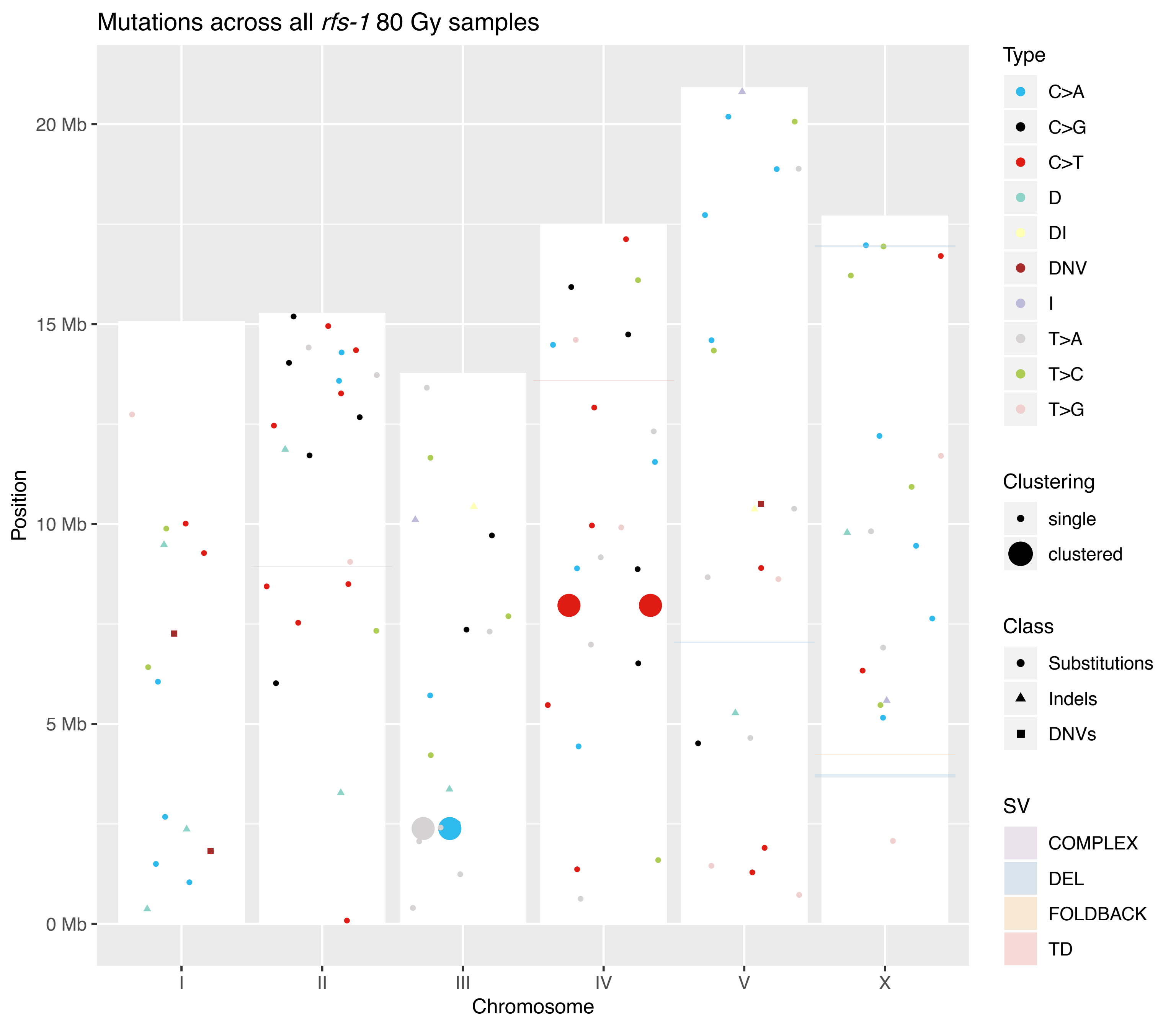
Mutations across all rfs-1 80 Gy samples
Position
20 Mb
15 Mb
10 Mb
5 Mb
0 Mb
I
II
III
IV
V
X
Chromosome
Type
C>A
C>G
C>T
D
DI
DNV
I
T>A
T>C
T>G
Clustering
single
clustered
Class
Substitutions
Indels
DNVs
SV
COMPLEX
DEL
FOLDBACK
TD

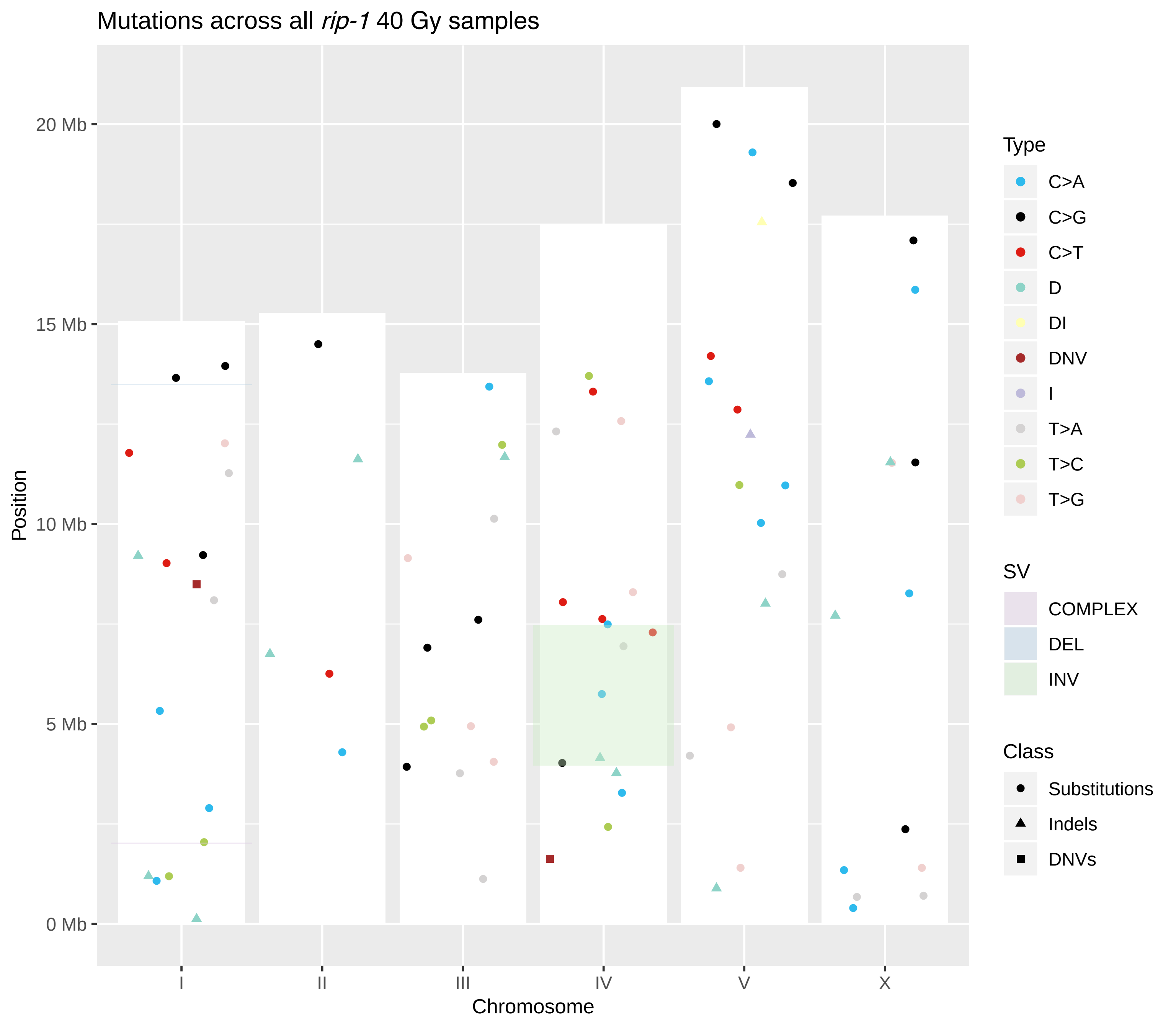
Mutations across all rip-1 40 Gy samples
Position
20 Mb
15 Mb
10 Mb
5 Mb
0 Mb
Chromosome
I
II
III
IV
V
X
Type
C>A
C>G
C>T
D
DI
DNV
I
T>A
T>C
T>G
SV
COMPLEX
DEL
INV
Class
Substitutions
Indels
DNVs

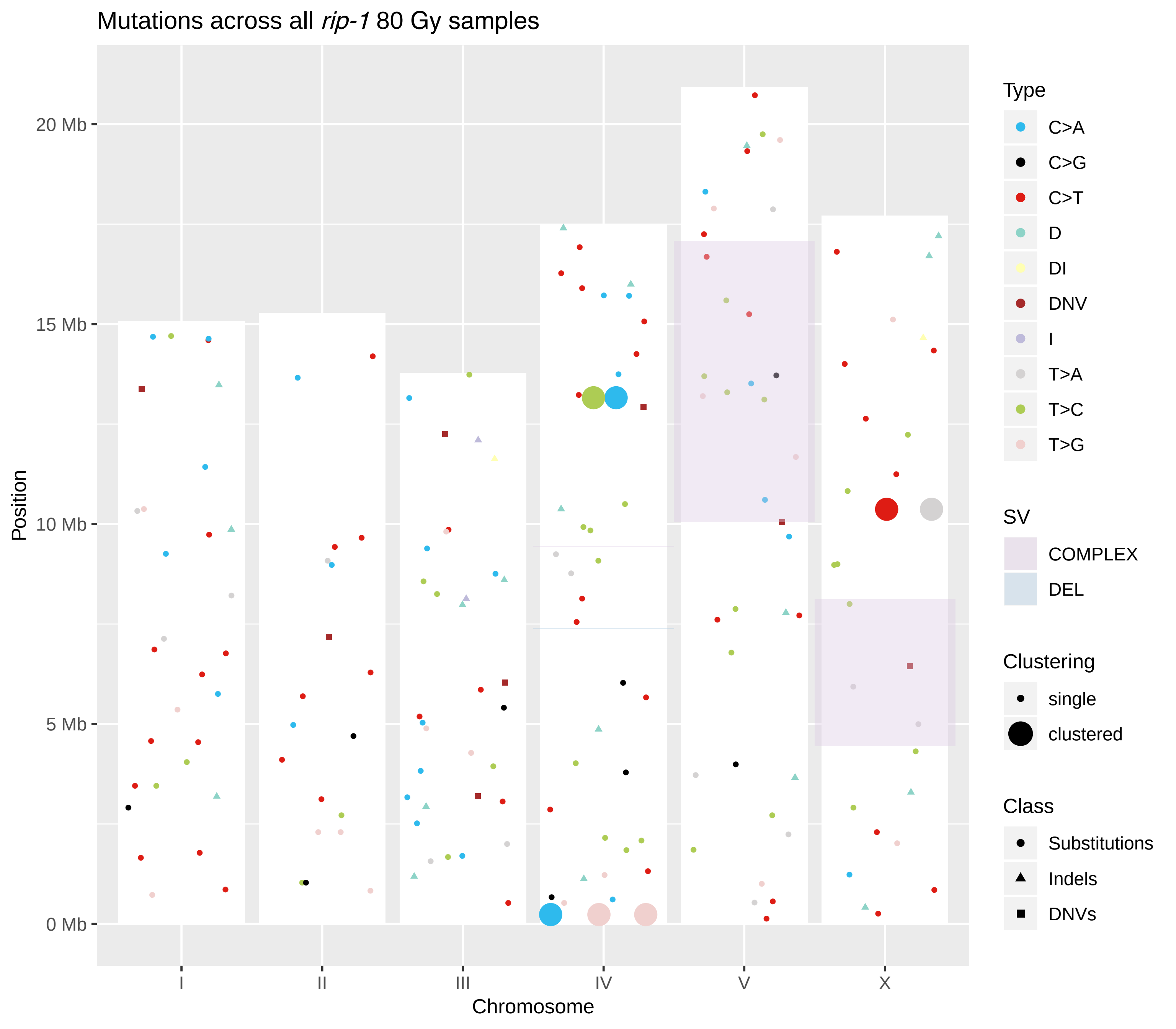
Mutations across all rip-1 80 Gy samples
Position
20 Mb
15 Mb
10 Mb
5 Mb
0 Mb
I
II
III
IV
V
X
Chromosome
Type
C>A
C>G
C>T
D
DI
DNV
I
T>A
T>C
T>G
SV
COMPLEX
DEL
Clustering
single
clustered
Class
Substitutions
Indels
DNVs

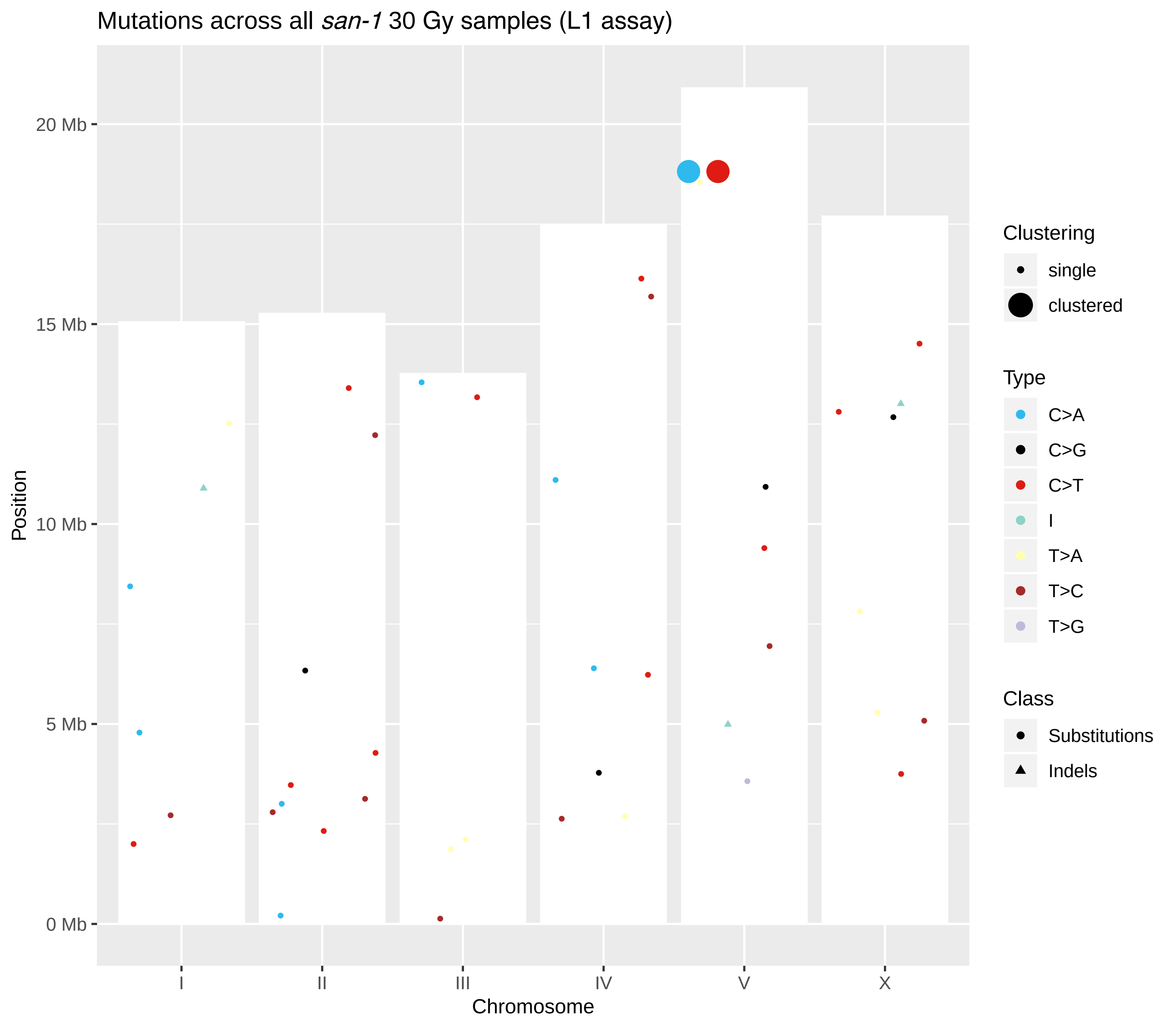

Mutations across all *san-1* 30 Gy samples (L1 assay)
Position
20 Mb
15 Mb
10 Mb
5 Mb
0 Mb
I
II
III
IV
V
X
Chromosome
Clustering
single
clustered
Type
C>A
C>G
C>T
I
T>A
T>C
T>G
Class
Substitutions
Indels

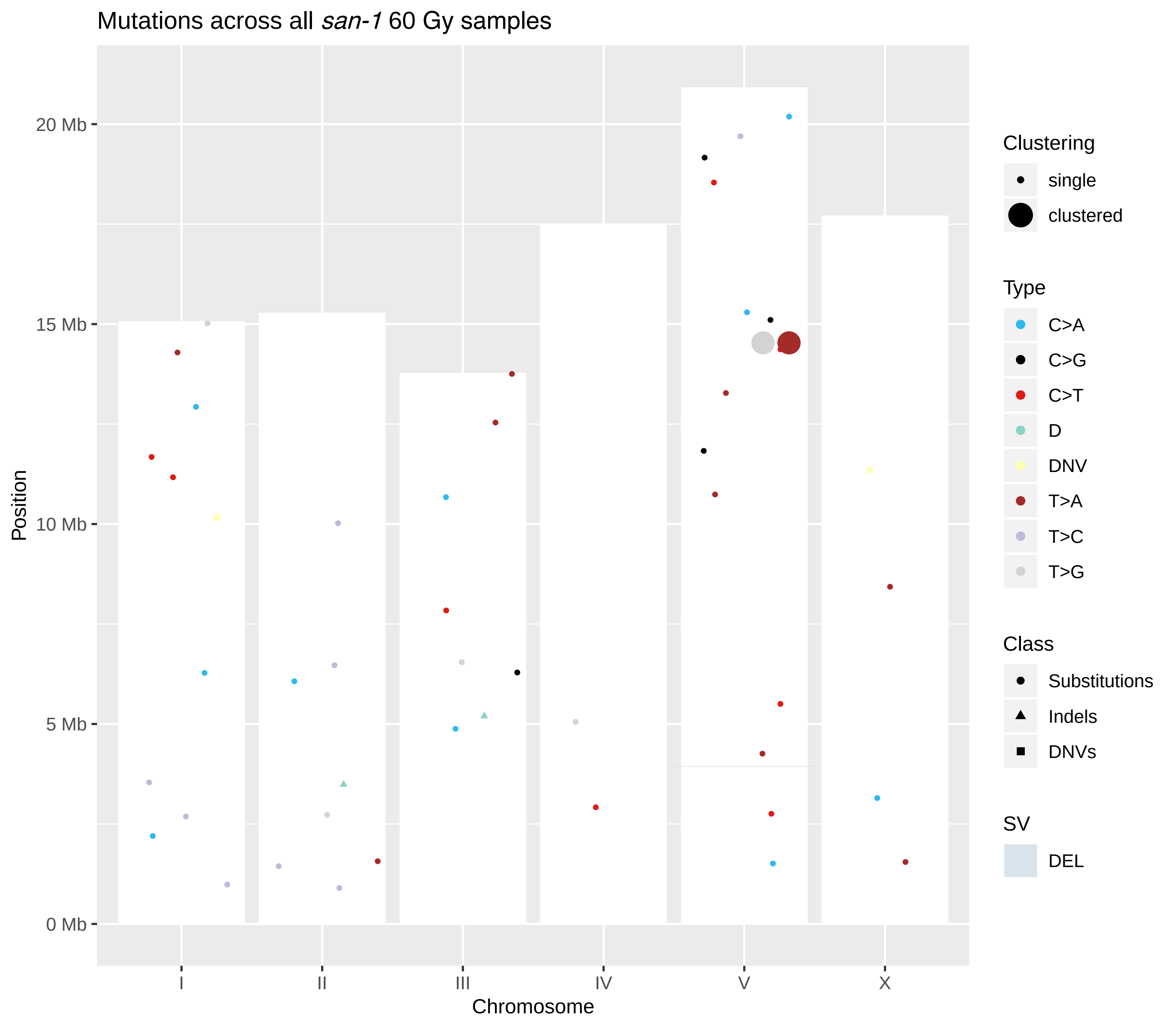

Mutations across all san-1 60 Gy samples
Position
20 Mb
15 Mb
10 Mb
5 Mb
0 Mb
I
II
III
IV
V
X
Chromosome
Clustering
single
clustered
Type
C>A
C>G
C>T
D
DNV
T>A
T>C
T>G
Class
Substitutions
Indels
DNVs
SV
DEL

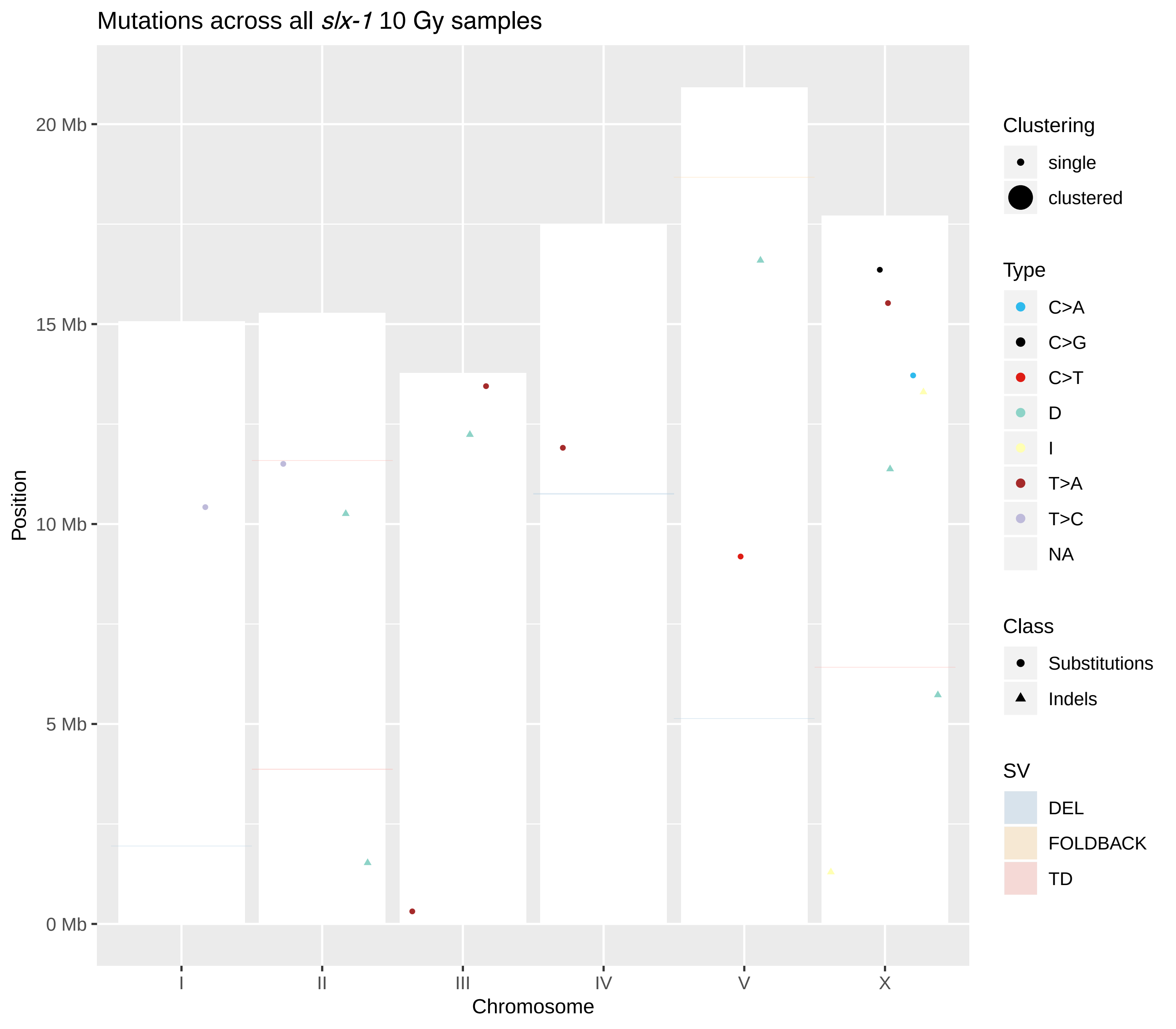
Mutations across all slx-1 10 Gy samples
Position
20 Mb
15 Mb
10 Mb
5 Mb
0 Mb
I
II
III
IV
V
X
Chromosome
Clustering
single
clustered
Type
C>A
C>G
C>T
D
I
T>A
T>C
NA
Class
Substitutions
Indels
SV
DEL
FOLDBACK
TD

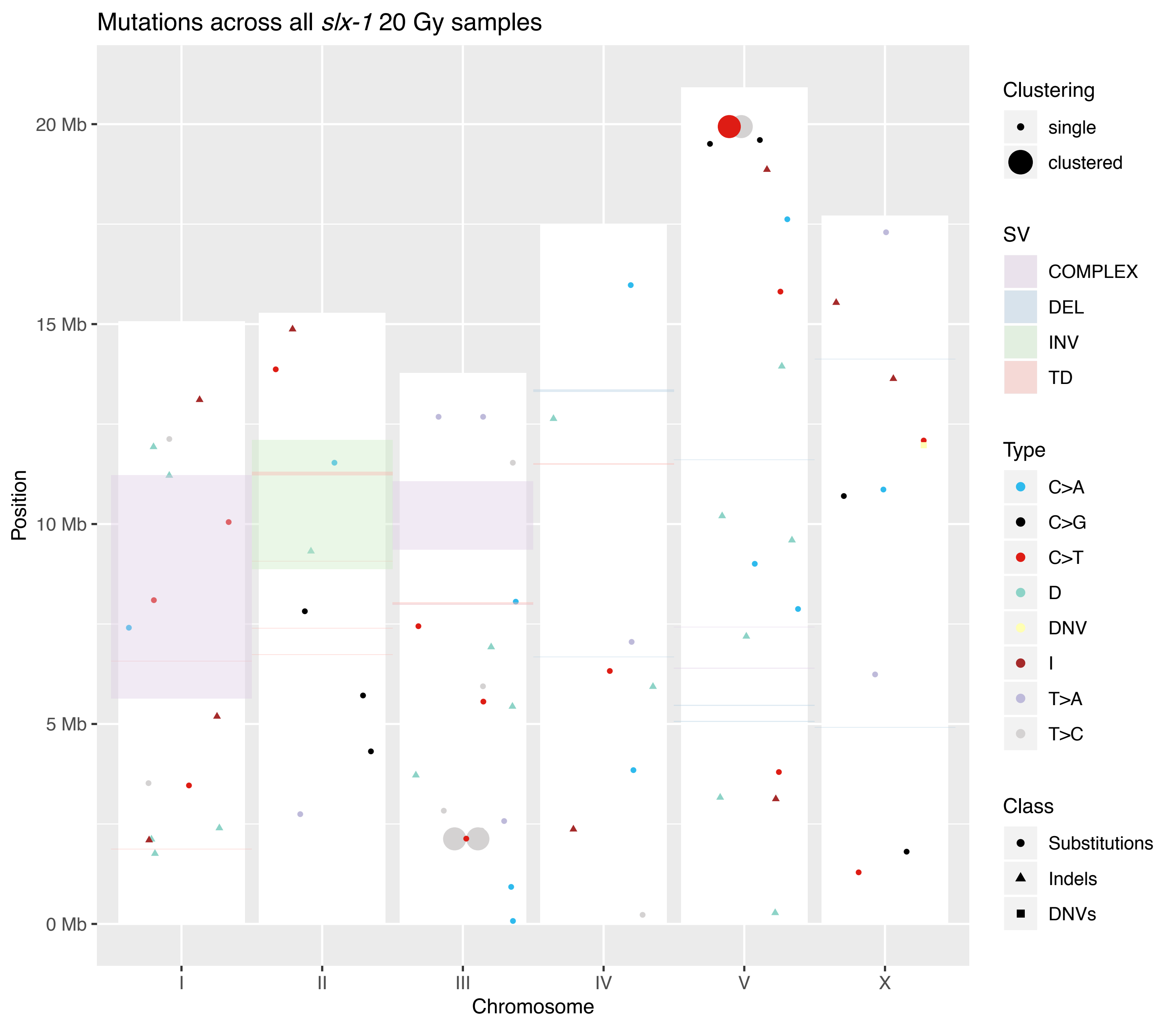

Mutations across all slx-1 20 Gy samples
Position
20 Mb
15 Mb
10 Mb
5 Mb
0 Mb
I
II
III
IV
V
X
Chromosome
Clustering
single
clustered
SV
COMPLEX
DEL
INV
TD
Type
C>A
C>G
C>T
D
DNV
I
T>A
T>C
Class
Substitutions
Indels
DNVs

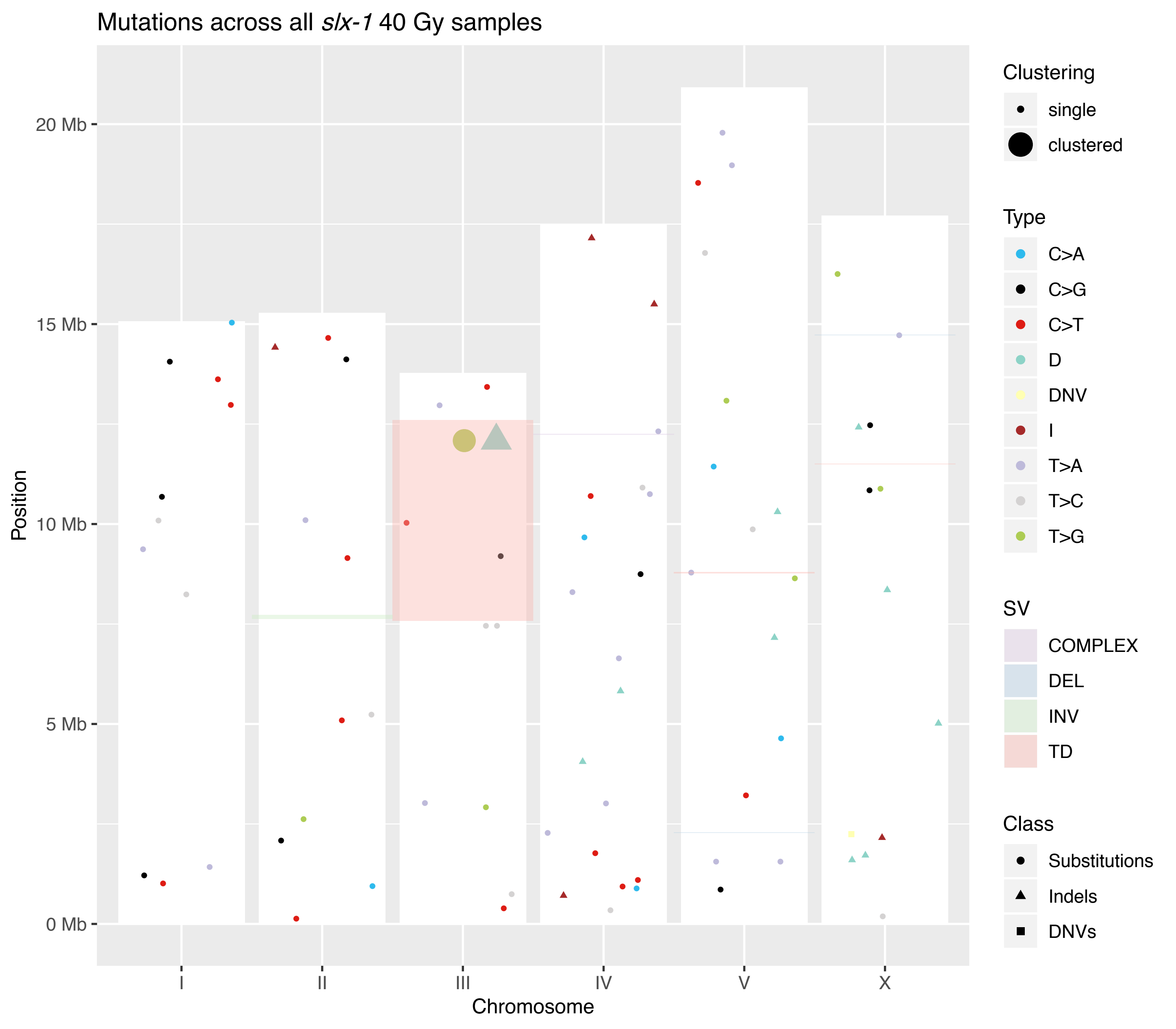

Mutations across all slx-1 40 Gy samples
Position
20 Mb
15 Mb
10 Mb
5 Mb
0 Mb
I
II
III
IV
V
X
Chromosome
Clustering
single
clustered
Type
C>A
C>G
C>T
D
DNV
I
T>A
T>C
T>G
SV
COMPLEX
DEL
INV
TD
Class
Substitutions
Indels
DNVs

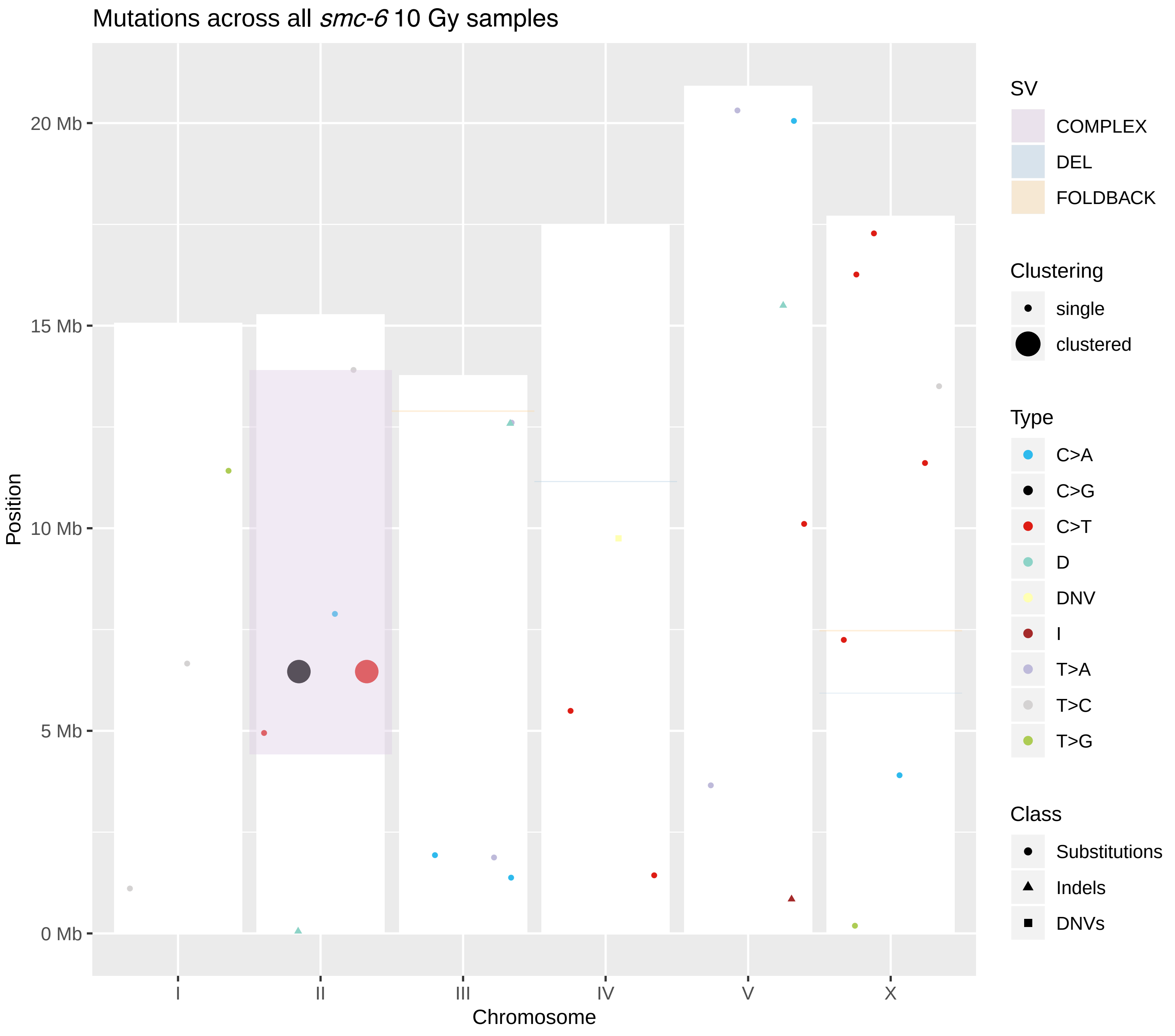

Mutations across all smc-6 10 Gy samples
Position
20 Mb
15 Mb
10 Mb
5 Mb
0 Mb
I
II
III
IV
V
X
Chromosome
SV
COMPLEX
DEL
FOLDBACK
Clustering
single
clustered
Type
C>A
C>G
C>T
D
DNV
I
T>A
T>C
T>G
Class
Substitutions
Indels
DNVs

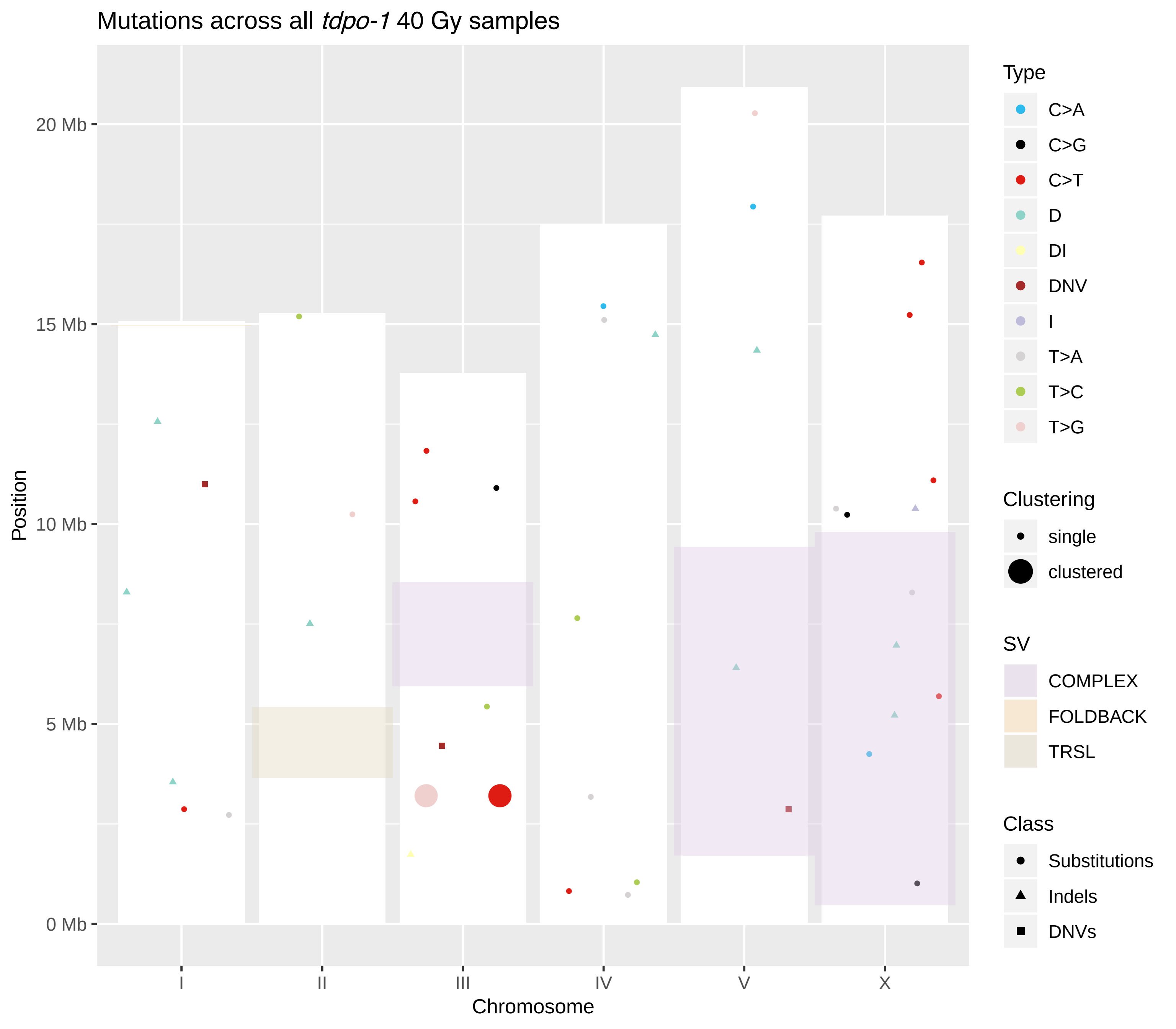

Mutations across all tdpo-1 40 Gy samples
Position
20 Mb
15 Mb
10 Mb
5 Mb
0 Mb
I
II
III
IV
V
X
Chromosome
Type
C>A
C>G
C>T
D
DI
DNV
I
T>A
T>C
T>G
Clustering
single
clustered
SV
COMPLEX
FOLDBACK
TRSL
Class
Substitutions
Indels
DNVs

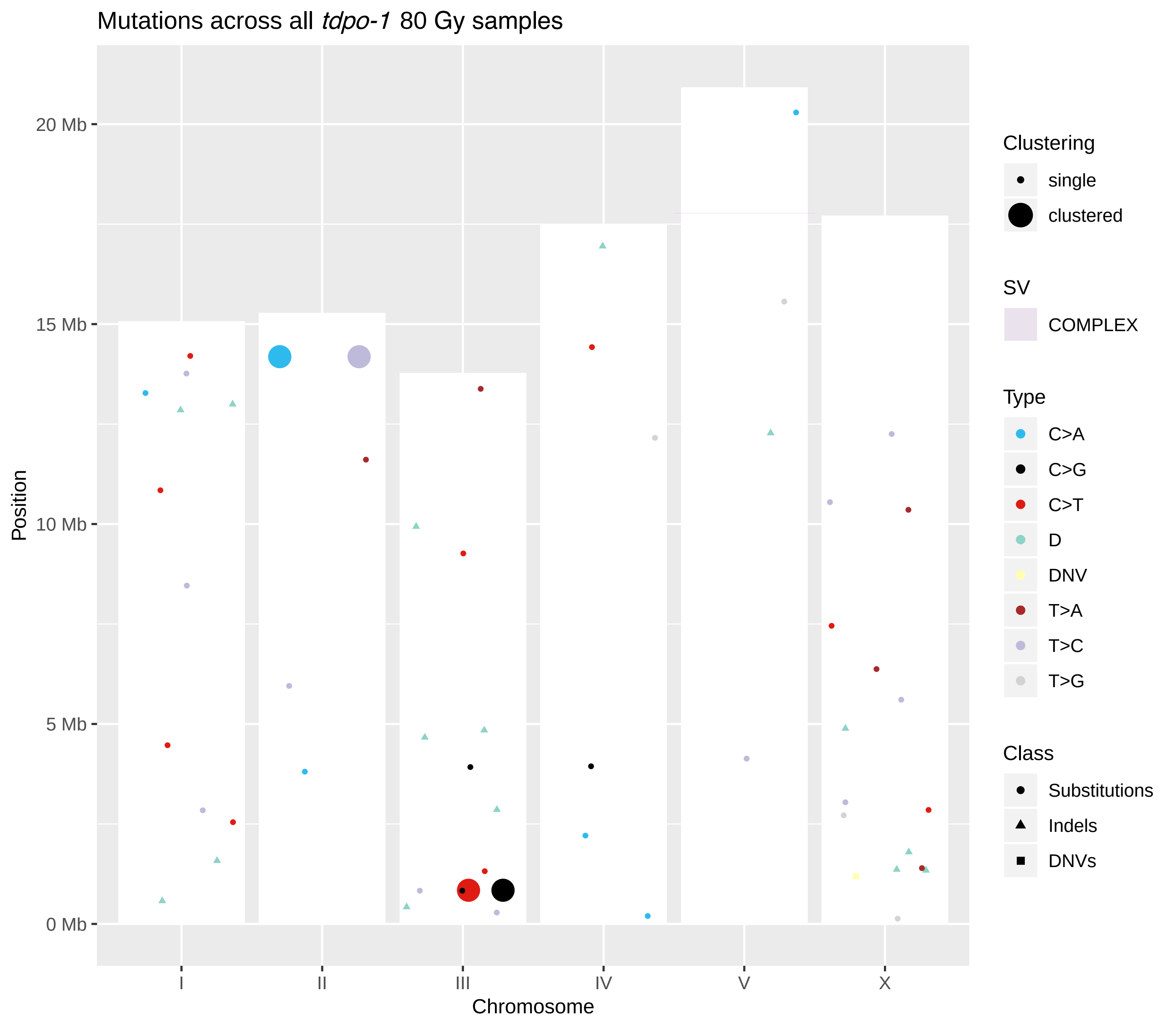

Mutations across all tdpo-1 80 Gy samples
20 Mb
15 Mb
10 Mb
5 Mb
0 Mb
Position
I
II
III
IV
V
X
Chromosome
Clustering
single
clustered
SV
COMPLEX
Type
C>A
C>G
C>T
D
DNV
T>A
T>C
T>G
Class
Substitutions
Indels
DNVs

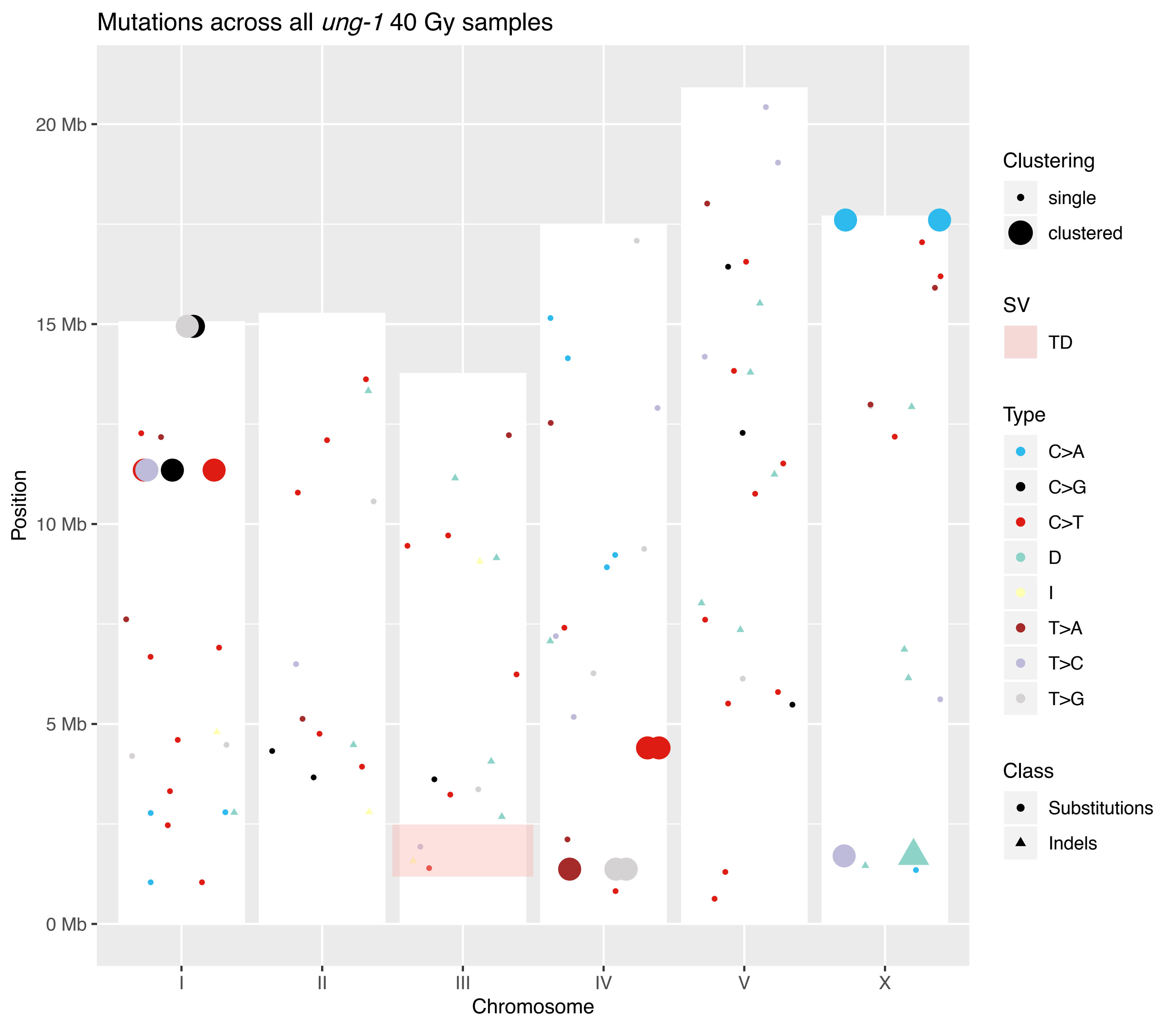
Mutations across all *ung-1* 40 Gy samples
Position
20 Mb
15 Mb
10 Mb
5 Mb
0 Mb
I
II
III
IV
V
X
Chromosome
Clustering
single
clustered
SV
TD
Type
C>A
C>G
C>T
D
I
T>A
T>C
T>G
Class
Substitutions
Indels

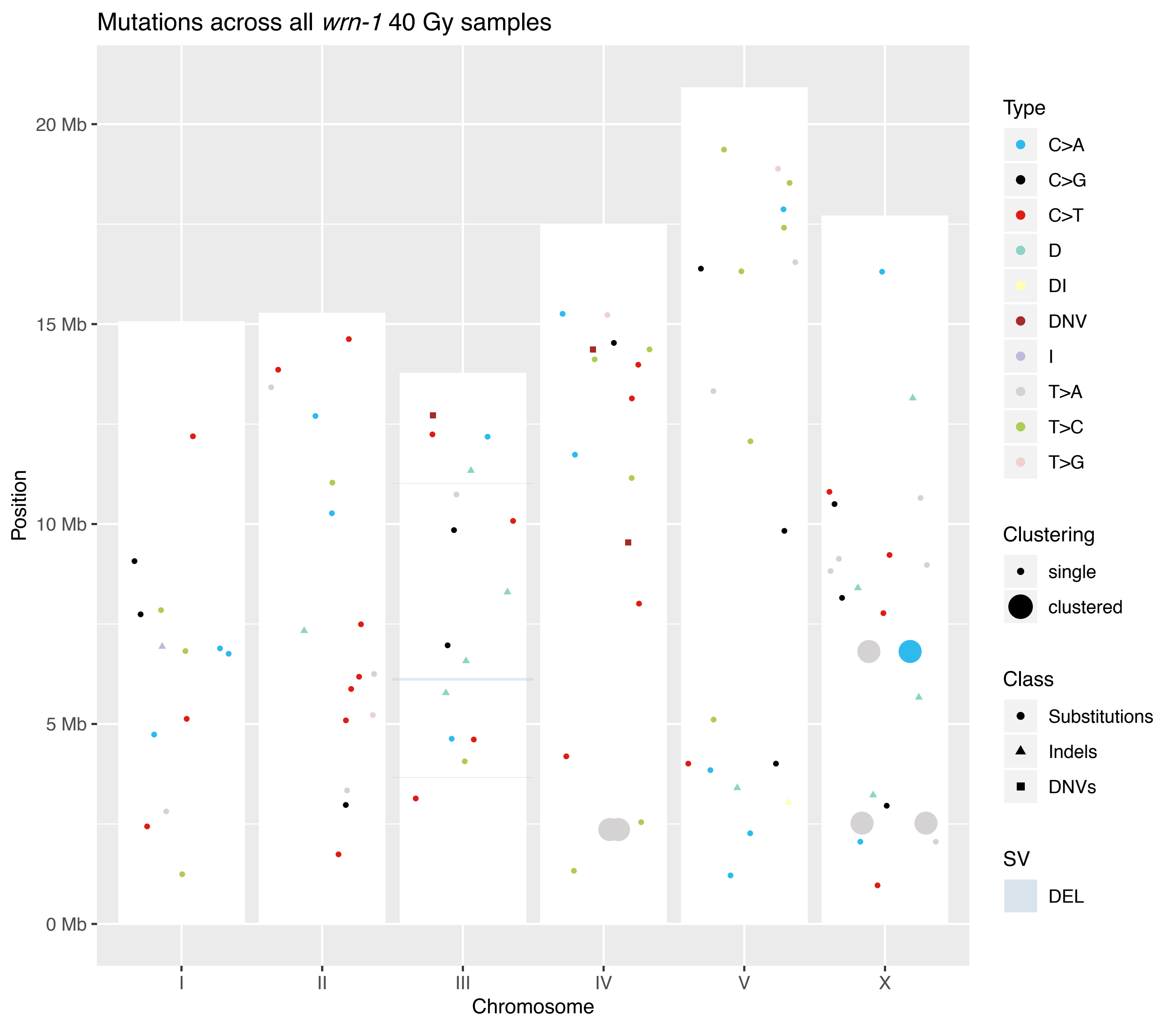

Mutations across all *wrn-1* 40 Gy samples
Position
20 Mb
15 Mb
10 Mb
5 Mb
0 Mb
I
II
III
IV
V
X
Chromosome
Type
C>A
C>G
C>T
D
DI
DNV
I
T>A
T>C
T>G
Clustering
single
clustered
Class
Substitutions
Indels
DNVs
SV
DEL

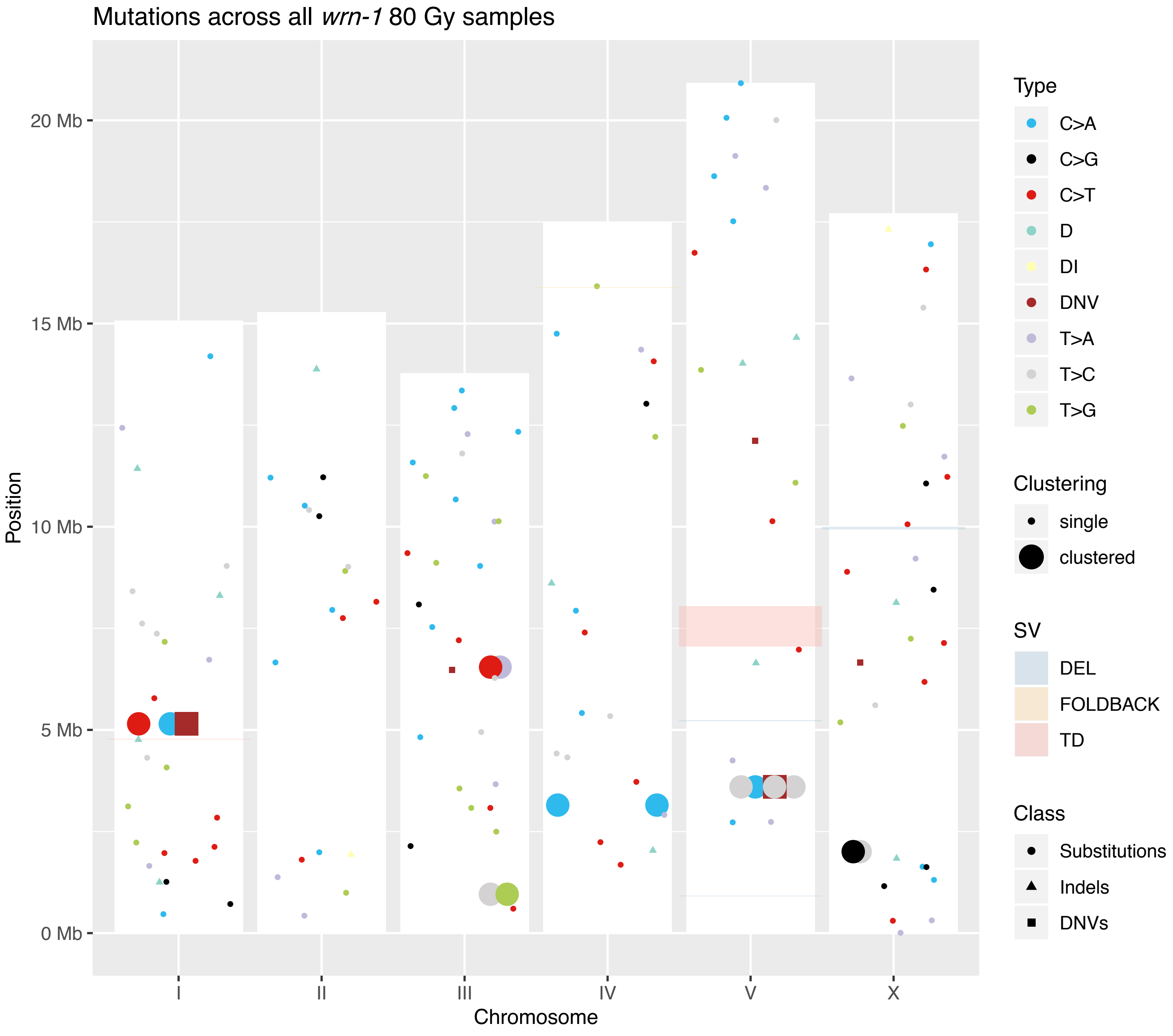

Mutations across all *wrn-1* 80 Gy samples
Position
20 Mb
15 Mb
10 Mb
5 Mb
0 Mb
I
II
III
IV
V
X
Chromosome
Type
C>A
C>G
C>T
D
DI
DNV
T>A
T>C
T>G
Clustering
single
clustered
SV
DEL
FOLDBACK
TD
Class
Substitutions
Indels
DNVs

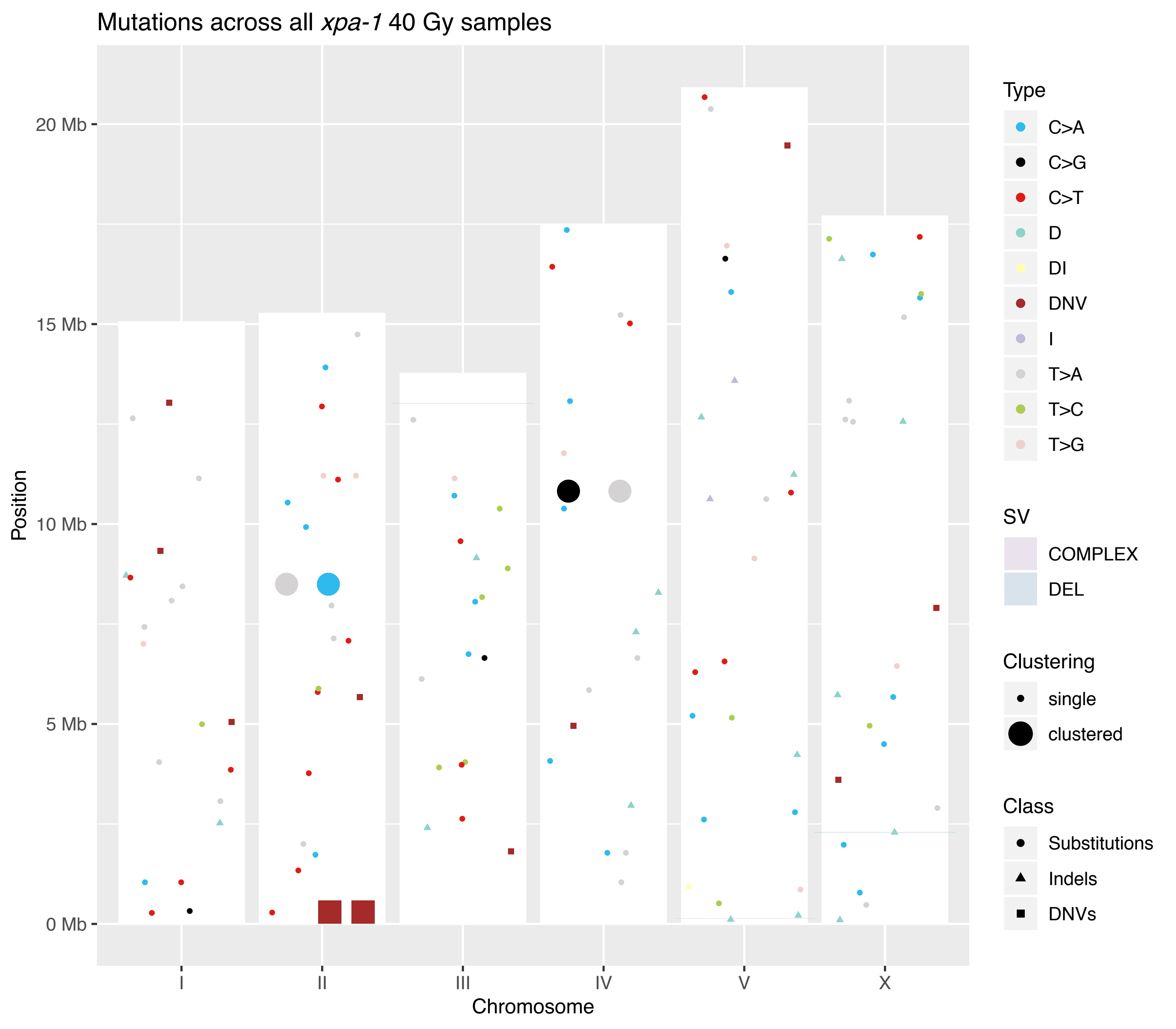

Mutations across all *xpa-1* 40 Gy samples
Position
20 Mb
15 Mb
10 Mb
5 Mb
0 Mb
I
II
III
IV
V
X
Chromosome
Type
C>A
C>G
C>T
D
DI
DNV
I
T>A
T>C
T>G
SV
COMPLEX
DEL
Clustering
single
clustered
Class
Substitutions
Indels
DNVs

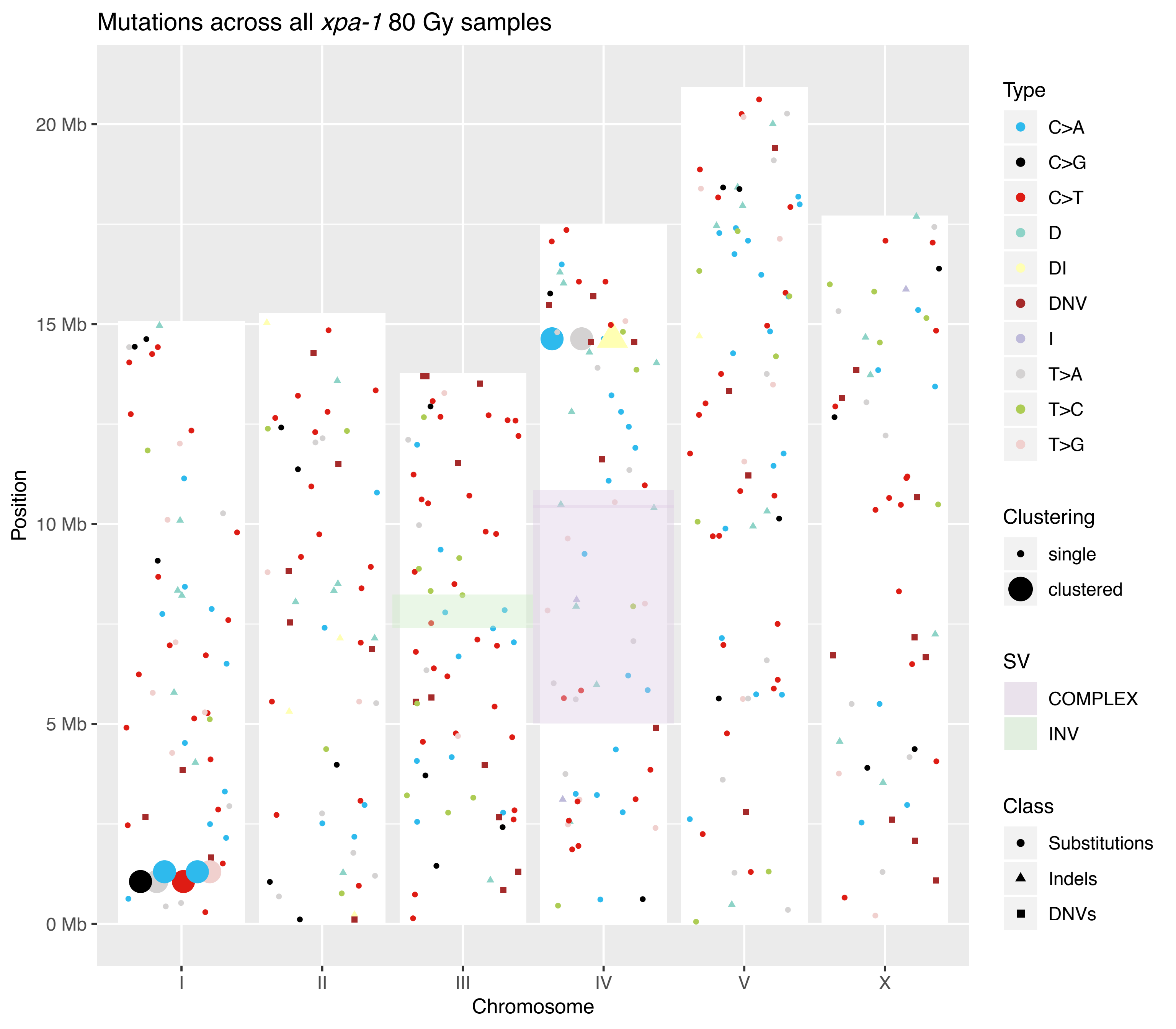
Mutations across all *xpa-1* 80 Gy samples
Position
20 Mb
15 Mb
10 Mb
5 Mb
0 Mb
Chromosome
I
II
III
IV
V
X
Type
C>A
C>G
C>T
D
DI
DNV
I
T>A
T>C
T>G
Clustering
single
clustered
SV
COMPLEX
INV
Class
Substitutions
Indels
DNVs

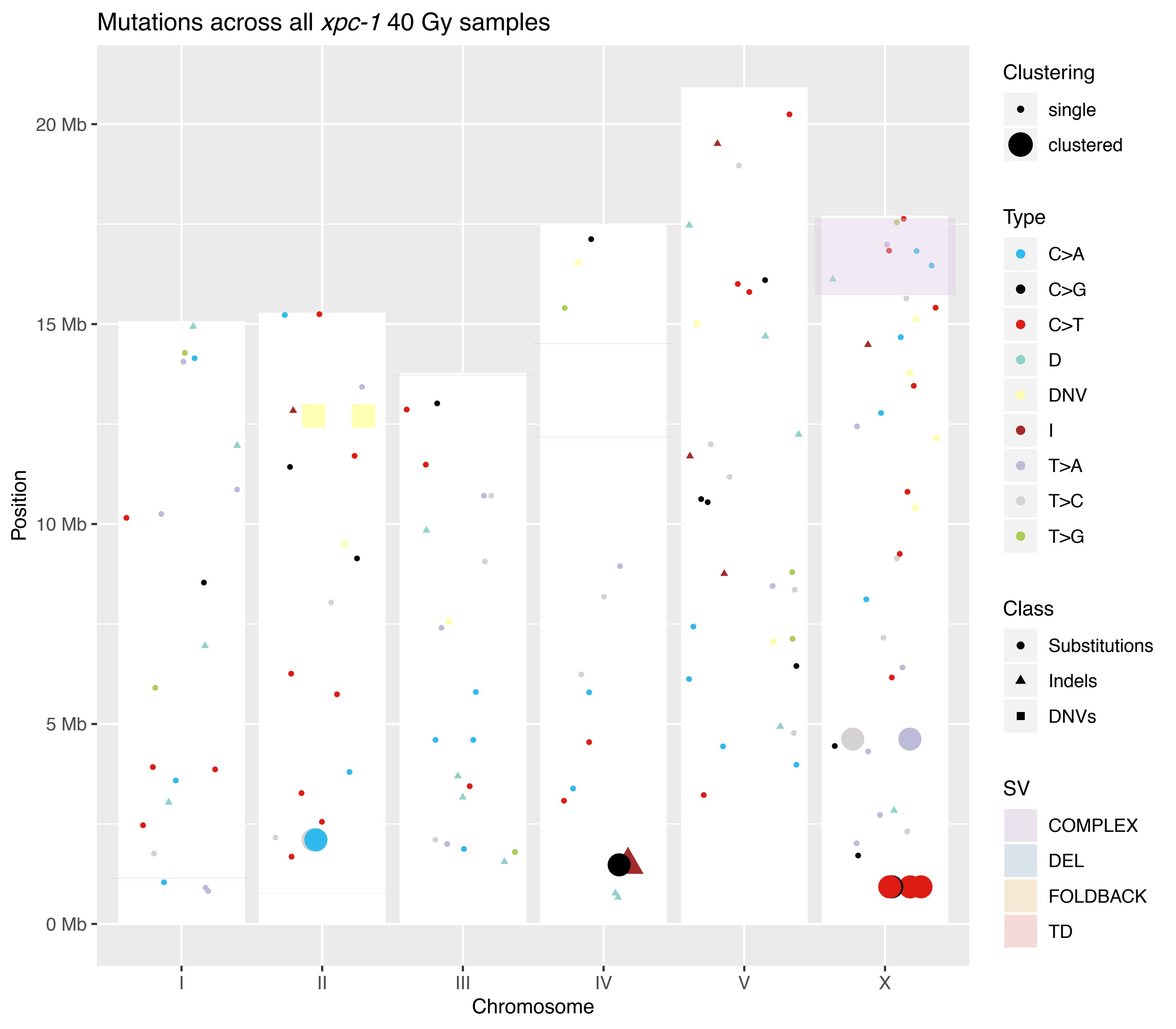
Mutations across all xpc-1 40 Gy samples
Position
20 Mb
15 Mb
10 Mb
5 Mb
0 Mb
I
II
III
IV
V
X
Chromosome
Clustering
single
clustered
Type
C>A
C>G
C>T
D
DNV
I
T>A
T>C
T>G
Class
Substitutions
Indels
DNVs
SV
COMPLEX
DEL
FOLDBACK
TD

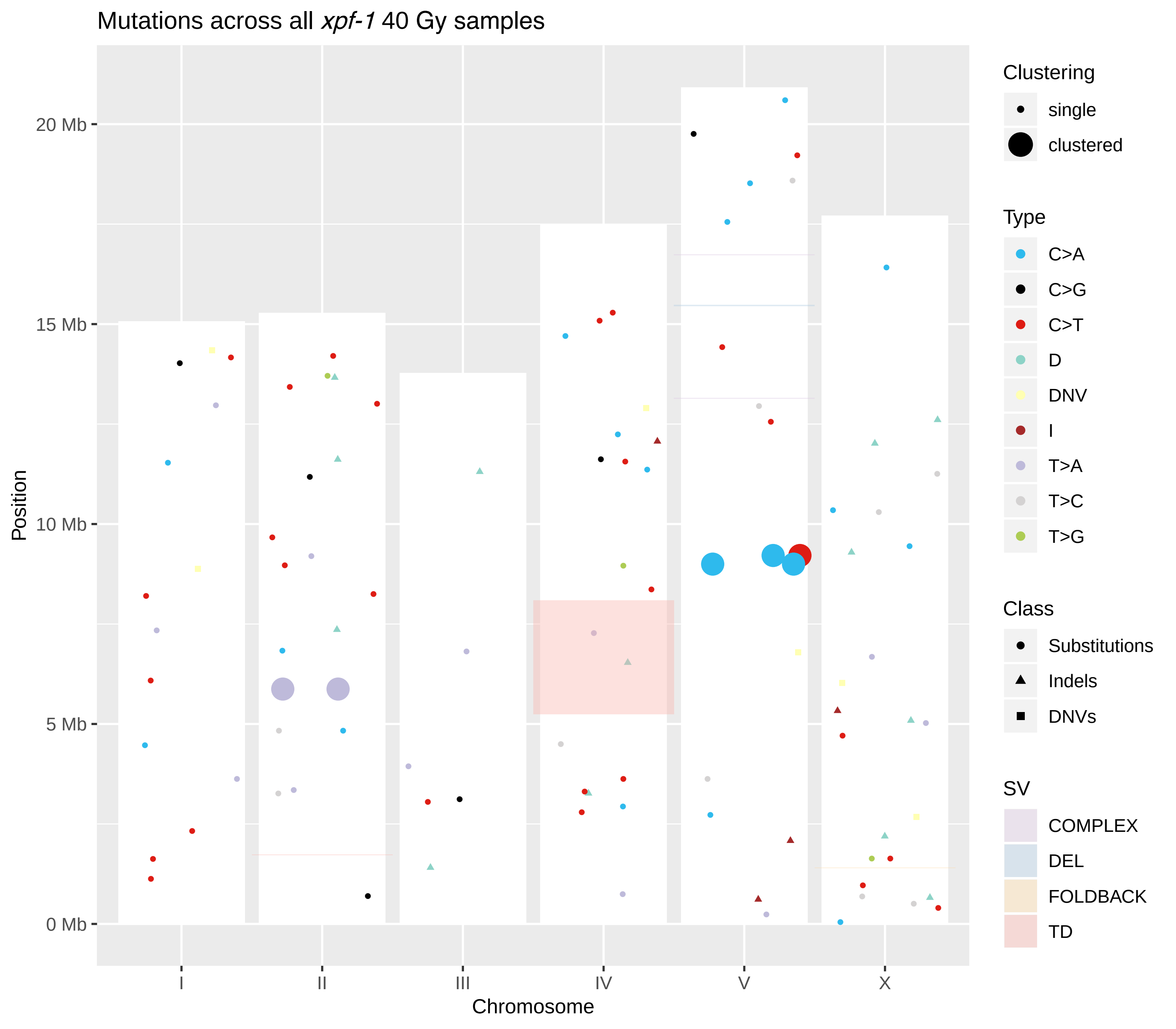
Mutations across all xpf-1 40 Gy samples
Position
20 Mb
15 Mb
10 Mb
5 Mb
0 Mb
I
II
III
IV
V
X
Chromosome
Clustering
single
clustered
Type
C>A
C>G
C>T
D
DNV
I
T>A
T>C
T>G
Class
Substitutions
Indels
DNVs
SV
COMPLEX
DEL
FOLDBACK
TD

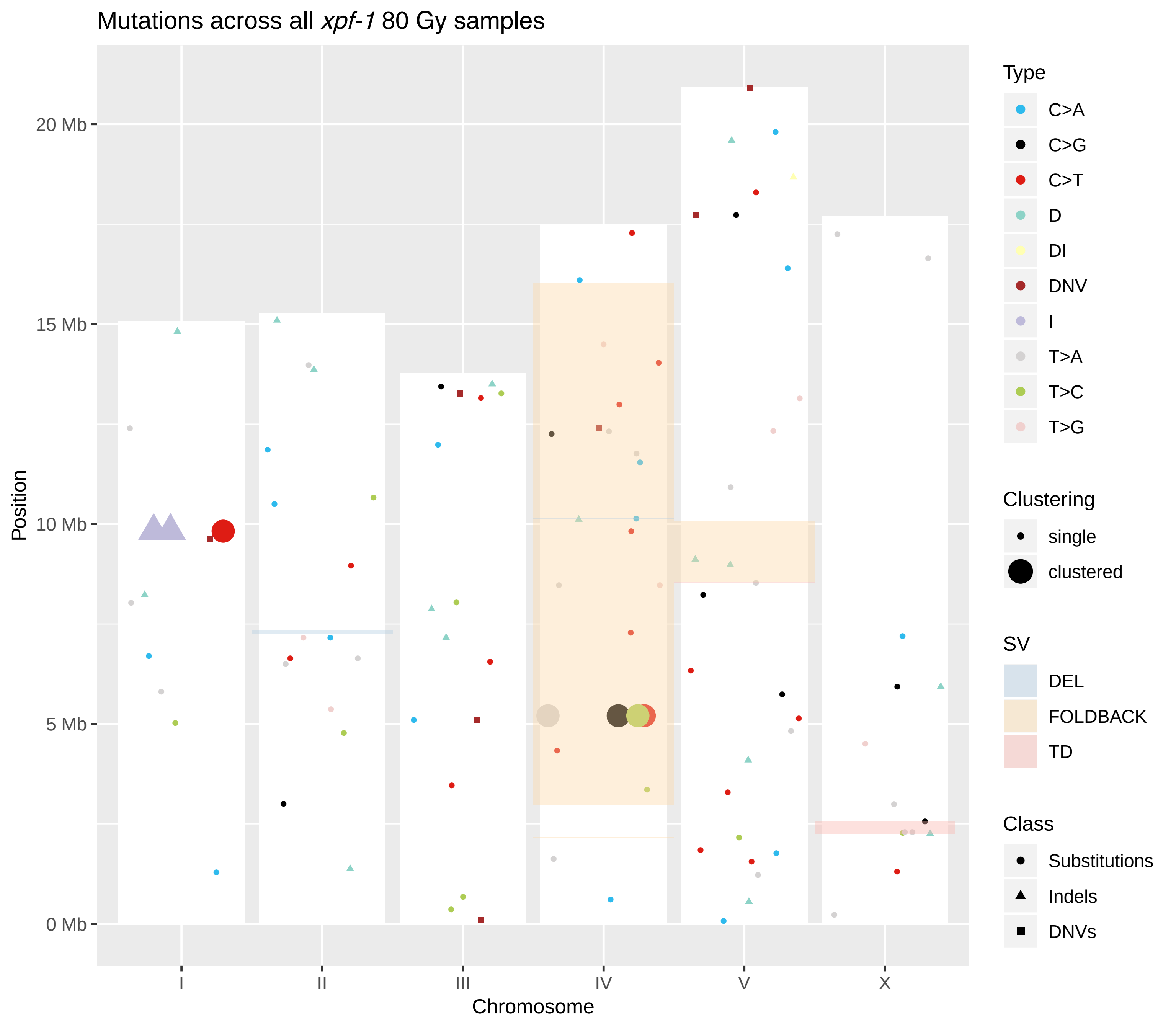

Mutations across all *xpf-1* 80 Gy samples
Position
20 Mb
15 Mb
10 Mb
5 Mb
0 Mb
I
II
III
IV
V
X
Chromosome
Type
C>A
C>G
C>T
D
DI
DNV
I
T>A
T>C
T>G
Clustering
single
clustered
SV
DEL
FOLDBACK
TD
Class
Substitutions
Indels
DNVs
