# Supplementary material for: C. elegans genome-wide analysis reveals DNA repair pathways that act cooperatively to preserve genome integrity upon ionizing radiation
Source: PLoS One. 2021 Oct 6;16(10):e0258269. doi: 10.1371/journal.pone.0258269 (PMC8494335; doi:10.1371/journal.pone.0258269)
Supplement: S4 File — (DOCX) [file pone.0258269.s014.docx]

**Supplementary Information: *C. elegans* genome-wide analysis reveals DNA repair pathways that act cooperatively to preserve genome integrity upon ionizing radiation**

Bettina Meier^1^*, Nadezda V. Volkova^2^*, Bin Wang^1,3^, Victor Gonzalez-Huici^1, 4^, Simone Bertolini^1^, Peter J. Campbell^5-7^, Moritz Gerstung^2, 8^, Anton Gartner^1,9,10^

Experimental details related to gDNA preparations, the exposure of nematodes to UV irradiation, cisplatin and Gamma-rays are described in Volkova et al. 2020 [[1]](https://paperpile.com/c/2Xy9s1/rBP2) publication (link: <https://www.nature.com/articles/s41467-020-15912-7#Sec11> ).

Filtered VCF files can be downloaded from the Supplementary Data 6 file collection - of Volkova et al. 2020 [[1]](https://paperpile.com/c/2Xy9s1/rBP2) using the list of samples described in Supplementary Table S1 (below).

**Data acquisition and filtering**

Procedures for Data acquisition and filtering are described below, following the corresponding Methods section of Volkova et al. 2020 [[1]](https://paperpile.com/c/2Xy9s1/rBP2)**.**

Filtering of samples was performed using the same criteria as in [[2]](https://paperpile.com/c/2Xy9s1/B7cW). Base substitutions were called using CAVEMAN [[3]](https://paperpile.com/c/2Xy9s1/j2h2) and subjected to the following filtering criteria:

1. Coverage of the variant site in both the sample of interest and reference should not exceed 150 reads or recede below 15 reads;
2. There should be no reads reporting the variant in the reference sample;
3. There should be at least 20% of reads and at least 5 reads reporting the variants in test sample;
4. There should be at least one read in the test sample reporting the variant in each direction;
5. There should be no indel called at the same position (relevant for homopolymer junctions);
6. All the variants are filtered against the normal panel of 6 wild-type samples; samples of the same genotype and generation higher than 1 are not compared to each other to avoid overfiltering of related samples.

Multiple substitutions which were found at adjacent sites in the same sample are classified as dinucleotide or multi-nucleotide variants, if their VAF is similar (difference less than 5%).

Small size insertions and deletions were called using PINDEL [[4]](https://paperpile.com/c/2Xy9s1/Yds0) and subjected to the following filtering criteria:

1. Coverage of the variant site in both the sample of interest and reference should not exceed 150 reads or recede below 10 reads;
2. There should be no more than 1 read reporting the variant in the reference sample;
3. There should be more than 20% of reads and at least 5 reads reporting the variants in test sample;
4. There should be at least one read in test sample reporting the variant in each direction;
5. If the variant falls into a repetitive region, the regions should not be longer than 18 repeats;
6. All the variants are filtered against the normal panel of 6 wild-type samples; samples of the same genotype and generation higher than 1 are not compared to each other to avoid overfiltering of related samples.
7. Indels above 400 bp in length were filtered out due to a high amount of false positives.

Indels are further classified based on the type (deletions, insertions, and complex indels - or deletions-insertions (DI)) and size (1 bp, 2-5 bp, 5-50 bp, 50-400 bp). Small insertions and deletions are further classified based on the local context: if the indel happened in repetitive sequence or not.

The structural variants were called using DELLY [[5]](https://paperpile.com/c/2Xy9s1/bR36) with the following filters:

1. Each variant should be supported by more than 10 high-quality reads in test sample and no reads in the reference sample;
2. Each variant should pass the default DELLY quality filter (which relates to mapping quality around the breakpoints);
3. Stand-alone deletions and tandem duplications were tested for the drop or increase in coverage, respectively, by comparing the ratio of the coverage in the test and control worms outside the variant breakpoints and between them;
4. Variants in telomeric regions were removed;
5. Duplicated SVs across unrelated samples were removed; samples of the same genotype and generation higher than 1 are not compared to each other to avoid overfiltering of related samples.
6. SV calls smaller than 450 bp in length were considered unreliable and filtered out.

The resulting sets of breakpoints were further classified in line with [[6]](https://paperpile.com/c/2Xy9s1/V2tq) using clustering by proximity and a simplified set of variants:

- Tandem duplications;
- Deletions;
- Inversions;
- Intrachromosomal translocations;
- Interchromosomal events;
- Foldbacks (change of the orientation of sequence without a second breakpoint in close proximity);
- Complex events (everything which was not possible to classify any other way).

**Supplementary References**

1. [Volkova NV, Meier B, González-Huici V, Bertolini S, Gonzalez S, Vöhringer H, et al. Mutational signatures are jointly shaped by DNA damage and repair. Nat Commun. 2020;11: 2169.](http://paperpile.com/b/2Xy9s1/rBP2)

2. [Meier B, Volkova NV, Hong Y, Schofield P, Campbell PJ, Gerstung M, et al. Mutational signatures of DNA mismatch repair deficiency in C. elegans and human cancers. Genome Res. 2018;28: 666–675.](http://paperpile.com/b/2Xy9s1/B7cW)

3. [Nik-Zainal S, Alexandrov LB, Wedge DC, Van Loo P, Greenman CD, Raine K, et al. Mutational Processes Molding the Genomes of 21 Breast Cancers. Cell. 2012. pp. 979–993. doi:](http://paperpile.com/b/2Xy9s1/j2h2)[10.1016/j.cell.2012.04.024](http://dx.doi.org/10.1016/j.cell.2012.04.024)

4. [Ye K, Schulz MH, Long Q, Apweiler R, Ning Z. Pindel: a pattern growth approach to detect break points of large deletions and medium sized insertions from paired-end short reads. Bioinformatics. 2009;25: 2865–2871.](http://paperpile.com/b/2Xy9s1/Yds0)

5. [Rausch T, Zichner T, Schlattl A, Stütz AM, Benes V, Korbel JO. DELLY: structural variant discovery by integrated paired-end and split-read analysis. Bioinformatics. 2012;28: i333–i339.](http://paperpile.com/b/2Xy9s1/bR36)

6. [Li Y, PCAWG Structural Variation Working Group, Roberts ND, Wala JA, Shapira O, Schumacher SE, et al. Patterns of somatic structural variation in human cancer genomes. Nature. 2020. pp. 112–121. doi:](http://paperpile.com/b/2Xy9s1/V2tq)[10.1038/s41586-019-1913-9](http://dx.doi.org/10.1038/s41586-019-1913-9)
